# Species‐level repertoire size predicts a correlation between individual song elaboration and reproductive success

**DOI:** 10.1002/ece3.5418

**Published:** 2019-07-02

**Authors:** Cristina M. Robinson, Nicole Creanza

**Affiliations:** ^1^ Department of Biological Sciences Vanderbilt University Nashville Tennessee

**Keywords:** Bayesian analysis, closed‐ended leaning, elaborate song, learned vocalization, meta‐analysis, open‐ended learning, passeriformes, reproductive success, sexual selection, songbirds, syllable repertoire

## Abstract

Birdsong has long been considered a sexually selected trait that relays honest information about male quality, and laboratory studies generally suggest that female songbirds prefer larger repertoires. However, analysis of field studies across species surprisingly revealed a weak correlation between song elaboration and reproductive success, and it remains unknown why only certain species show this correlation in nature. Taken together, these studies suggest that females in numerous species can detect and prefer larger repertoires in a laboratory setting, but larger individual repertoires correlate with reproductive success only in a subset of these species. This prompts the question: Do the species that show a stronger correlation between reproductive success and larger individual repertoires in nature have anything in common? In this study, we test whether between‐species differences in two song‐related variables—species average syllable repertoire size and adult song stability over time—can be used to predict the importance of individual song elaboration in reproductive success within a species. Our cross‐species meta‐analysis of field studies revealed that species with larger average syllable repertoire sizes exhibited a stronger correlation between individual elaboration and reproductive success than species with smaller syllable repertoires. Song stability versus plasticity in adulthood provided little predictive power on its own, suggesting that the putative correlation between repertoire size and age in open‐ended learners does not explain the association between song elaboration and reproductive success.

## 1 INTRODUCTION

Birdsong is among the most elaborate forms of vocal communication in nature. In addition, song plays a critical role in reproduction: Songbirds that cannot sing a species‐typical song have an impaired ability to attract mates (Catchpole & Slater, 2003). In many different species, song elaboration (e.g., the size of the song repertoire and syllable repertoire), the accuracy of learning, or performance characteristics (e.g., trill rate or stereotypy) of an individual male's song have been shown to be influenced by variables such as developmental stress or age (Nowicki, Peters, & Podos, 1998; Nowicki & Searcy, 2004; Slater, 2003). These variables have been linked to both song quality and reproductive success, which is measured as mating success (female choice) or reproductive output (the number of offspring a male produces). Therefore, song has the potential to act as an honest and reliable indicator of individual male quality (Gil & Gahr, 2002).

Songbird repertoire sizes have been associated with genetic, environmental, and cultural factors (Lachlan & Slater, 2003; Nowicki, Searcy, & Peters, 2002; Reid et al., 2005; Roper & Zann, 2006; Spencer, Buchanan, Goldsmith, & Catchpole, 2003; Tchernichovski, Mitra, Lints, & Nottebohm, 2001). In addition, an individual male's repertoire size could signal his song‐learning ability to potential mates, as song learning can be anatomically constrained by brain nucleus size or neuron number in birds (Devoogd, Krebs, Healy, & Purvis, 1993; Gil & Gahr, 2002). In other words, while song is culturally transmitted in songbirds, a high‐quality song might also be associated with a better genetic background, increased learning ability, a higher quality cultural model, a less stressful environment, or some combination, all of which could correlate with fitness. Thus, individual song elaboration could be indicative of males that can achieve greater reproductive success (a) by having greater reproductive output and leaving more surviving offspring, and/or (b) by having increased mating success if females prefer males with larger repertoires. We call attention to this distinction since song has been linked to both of these measures of reproductive success in previous studies, though these measurements reflect aspects of sexual selection that are difficult to disentangle from one another. Repertoire size has been linked to paternal effort in feeding nestlings (Buchanan & Catchpole, 2000) and with the number of eggs laid by a female (Kroodsma, 1976), both of which would potentially increase reproductive output. In addition, laboratory studies in numerous species have suggested that female birds tend to prefer more elaborate songs, which might link song and syllable repertoire size to mate choice (Baker, Bjerke, Lampe, & Espmark, 1986; Catchpole, Dittami, & Leisler, 1984; Catchpole, Leisler, & Dittami, 2010; Lampe & Saetre, 1995; Searcy, 1984, 1992; Verheyen, Eens, & Pinxten, 1991). Thus, song elaboration, as measured by an individual's number of unique song types (song repertoire size) or unique syllable types (syllable repertoire size), has long been hypothesized to be important in sexual selection (Catchpole, 1980, 1987; Howard, 1974; Kroodsma, 1976; Macdougall‐Shackleton, 1997; Nowicki et al., 1998; Pfaff, Zanette, MacDougall‐Shackleton, & MacDougall‐Shackleton, 2007; Reid et al., 2004; Searcy, 1984, 1992; Searcy & Andersson, 1986; Yasukawa, Blank, & Patterson, 1980), and studies of both reproductive output and mate choice might be expected to show positive associations with individual song elaboration.

However, analysis of the literature seeking to correlate individual song elaboration with reproductive success across species has not revealed a strong relationship between the two. One review (Byers & Kroodsma, 2009) found that whereas many females show increased copulation responses to songs with larger repertoires in laboratory experiments (~80% of studies), field studies were much less likely to find a relationship between repertoire size and mate choice (~35% of studies). These results led to the conclusion that although females of many species may have a preference for elaborate songs, this preference does not necessarily play a significant role in mate choice. Instead, the quality of other factors, such as territory, plumage, or other song variables including performance and stereotypy, may be more influential in female choice than repertoire size in some bird species (Ballentine, 2004; Gontard‐Danek, 1999; Logue & Forstmeier, 2008; Nowicki, Searcy, & Peters, 2002; Treisman, 1978; Williams & Slater, 1990). A subsequent quantitative meta‐analysis consisting of only field studies (Soma & Garamszegi, 2011) found a significant effect of song elaboration on reproductive success—as measured by both mate choice and reproductive output—but the association was weak, with the average effect size (*r*) ranging between 0.1 and 0.3, depending on how the data were incorporated into the meta‐analysis and whether publication bias was controlled for in the final dataset.

Thus, there was a marked difference between the results of these reviews (Byers & Kroodsma, 2009; Soma & Garamszegi, 2011) and the long‐standing expectation that song elaboration plays a prominent role in sexual selection (Catchpole, 1980, 1987; Howard, 1974; Kroodsma, 1976; Macdougall‐Shackleton, 1997; Nowicki et al., 1998; Pfaff et al., 2007; Reid et al., 2004; Searcy, 1984, 1992; Searcy & Andersson, 1986; Yasukawa et al., 1980). Field studies that do not find a relationship between song elaboration and reproductive success often use territory quality as a putative explanatory variable, implying that, in certain species, environmental factors make it advantageous for females to pick territories on which to raise young based on the abundance or stability of resources instead of making decisions based on the resident male's song quality (Catchpole, 1986; Hiebert, Stoddard, & Arcese, 1989). Indeed, both of the aforementioned reviews (Byers & Kroodsma, 2009; Soma & Garamszegi, 2011) also proposed that song elaboration may not be under universal selective pressure across species. If song elaboration is not under a universal selection pressure across all bird species, it raises the question: Is there a subset of bird species for which song elaboration correlates with reproductive success, and do those species have anything in common?

**Figure 1:**
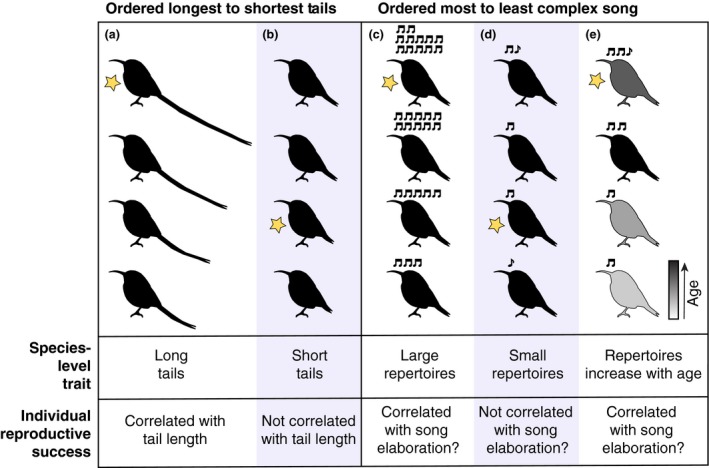
Schematic of traits that may predict the presence of sexual selection. (a–b) Males are ranked by tail length (longest tails at the top). (c–e) Males are ranked by song elaboration (most elaborate songs at the top). In all cases, the male with the highest reproductive success is marked by a star. If males with more exaggerated tails are more reproductively successful, due to female preference or genetic superiority, and if tail length is heritable, we would expect the species distribution to shift toward more exaggerated phenotypes. In this case, one would predict that sexual selection for exaggerated tails is more likely occurring in species with a long average tail length (a) than in species with a short average tail length (b). In this same line of thinking, if males with more elaborate songs are more reproductively successful and song elaboration is heritable, then one would predict that sexual selection for more elaborate song is more likely occurring in species with a large average repertoire size (c) than in species with a small average repertoire size (d). Alternatively, if males can learn more syllables as they age (open‐ended learners), syllable repertoire size could act as a signal for male age (e). Females might hypothetically prefer older males, because they have more breeding experience than their younger counterparts and have proven their survival capability. Thus, females would prefer mates with larger repertoires if repertoire size correlates with age. In contrast, in closed‐ended learners, where song cannot signal age, females would not prefer more elaborate songs

Here, we propose two potential species‐level traits that might help predict the strength of the correlation between individual song elaboration and reproductive success. The first hypothesis we test is whether an aspect of song—species‐level average syllable repertoire size—may help predict the strength of this correlation. This would be comparable to tail length in birds, which represents a particularly well‐studied case of sexual selection. Numerous studies have linked within‐species variation in tail length to fitness: Individuals with longer tails generally have greater reproductive success. However, there is a between‐species component to these observations as well; tail length has primarily been shown to correlate with mating success in species that have elongated tails, whereas tail length has *not* been correlated to reproductive success in studied species with shorter tails. (Andersson, 1982, 1992; Griggio, Valera, Casas‐Crivillé, Hoi, & Barbosa, 2010; Pryke, Andersson, & Lawes, 2001; Thusius, Peterson, Dunn, & Whittingham, 2001; Westneat, 2006). Thus, it is likely that a species with sexual selection for tail length could, on average, have longer tails than a species that does not (Figure 1). In this sense, between‐species differences in tail length might have predictive power: In species that have longer tails on average, we might hypothesize that individuals with longer tails will have higher reproductive success. Here, we propose that this same line of reasoning might apply to between‐species differences in syllable repertoire size. For the remainder of the paper, we use “individual song elaboration” to refer to within‐species differences and “species average syllable repertoire size” to refer to between‐species differences.

If males with greater individual song elaboration are more reproductively successful and these males tend to produce offspring with larger‐than‐average repertoires, then the average syllable repertoire size of the entire species could gradually increase under this sexual selection pressure (Figure 1). Thus, larger average species syllable repertoires may be a signature of bird species in which individual song elaboration correlates with reproductive success. An alternative explanation that relies primarily on reproductive output could hinge on the difficulty of learning the species syllable repertoire; it may be that fairly small average species syllable repertoires can be learned even by less fit males. Thus, larger individual song elaboration would only correlate with reproductive output in species with average syllable repertoire sizes that are large enough that less fit males cannot easily learn the full repertoire.

Alternatively, birds with small average species syllable repertoires might attend more closely to repertoire size, because it is easier to discern which males have more elaborate songs when each male only produces a handful of syllables. In birds with larger average species syllable repertoires, it would take more listening time and be more difficult for a female to discern which potential mate shows greater song elaboration (Krebs, 1977; Krebs & Kroodsma, 1980). Thus, small increases in individual song elaboration may be more meaningful in birds with smaller average species syllable repertoires, where differences can be quickly perceived. If this correlation is not driven by female preferences, it is more difficult to postulate a reason why a link between individual song elaboration and reproductive output would exist only in species with smaller average species syllable repertoires.

We also propose a second hypothesis: That the association between individual song elaboration and reproductive success could differ between species based on the length of the song‐learning window when measured as the stability of the species’ songs over time. The length of the song‐learning window varies greatly between species, but most can be roughly divided into two categories: (a) “age‐limited” or “closed‐ended” learners and (b) “open‐ended” learners (Beecher & Brenowitz, 2005; Brenowitz & Beecher, 2005; Williams, 2004). Closed‐ended learners do not modify their songs in adulthood (Beecher & Brenowitz, 2005). They have a set developmental window, or sensitive period, within which they must learn to produce their adult song. Once this time window closes, the adult song is crystallized, and the bird will not change its syllable or song repertoire. In contrast, open‐ended song learning has been defined as a bird's ability to modify its song after its first calendar year (Beecher & Brenowitz, 2005). Open‐ended learners do not have a set developmental window within which they learn their adult song and can continuously or seasonally add, subtract, and/or alter syllables or songs in their adult repertoire. Of note, some open‐ended learners are known to increase the overall size of their repertoires as they age (Searcy & Andersson, 1986). This means that in open‐ended species, song elaboration can potentially indicate the age of a male in addition to his song‐learning capacity (Figure 1). Furthermore, in some species, older males are preferred over younger males (Dickinson, 2001; Sundberg & Dixon, 1996), and, more generally, longevity might be a signal of high genetic quality or greater foraging experience (Brooks & Kemp, 2001; Kipper & Kiefer, 2010; Kokko, 1998; Martin, 1995; however, see Schroeder, Nakagawa, Rees, Mannarelli, & Burke, 2015). Therefore, our first hypothesis is that females from species with open‐ended learning would prefer greater song elaboration moreso than closed‐ended learners, in which song elaboration cannot signal age. Since the true length of the song‐learning window (whether a bird *can* change its repertoire) is incredibly difficult to measure in the field, we use song stability (whether a bird *does* change its repertoire) as a proxy. If a longitudinal study of a species showed that individual's songs remained the same from year to year, we classified that species as having a stable song, but if a study showed that individual's syllable repertoire changed between seasons (exchanging old syllables for new ones) or their syllable repertoire sizes increased with age, we classified that species as having a plastic song.

In this study, we attempt to determine whether species average syllable repertoire size or song stability can predict the strength of the correlation between individual song elaboration and reproductive success. Using a Bayesian multilevel phylogenetic meta‐analysis of available field data, we observed that larger species average syllable repertoire sizes predict for a stronger correlation between individual song elaboration and reproductive success, whereas song stability versus plasticity did not predict the strength of this correlation.

## 2 METHODS

### 2.1 Data collection

**Figure 2:**
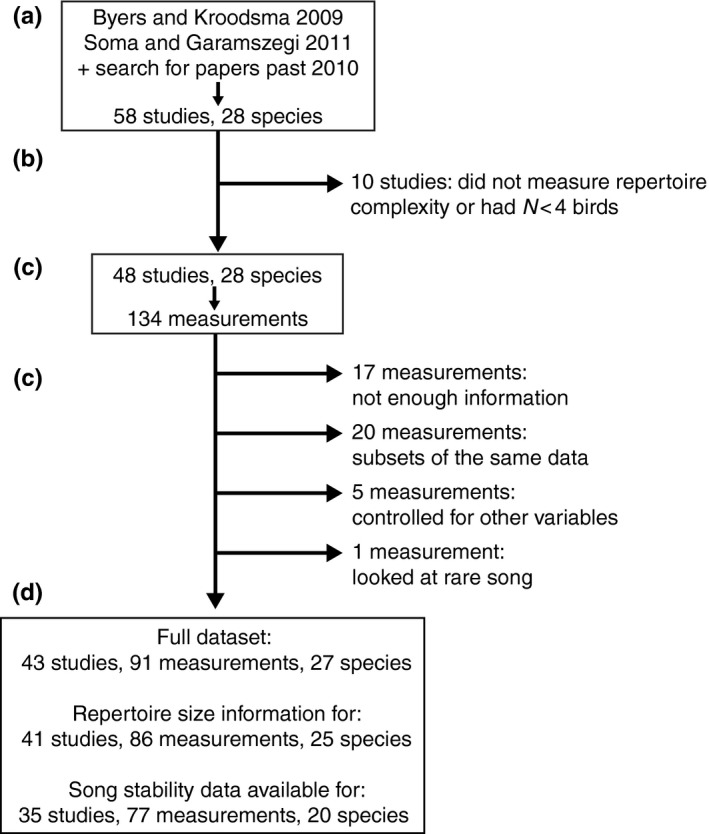
Schematic of dataset formation. (a) Fifty‐seven studies and one thesis were compiled from the references in Byers and Kroodsma 2009, Soma and Garamszegi 2011, and a search for studies and theses published since those reviews. (b) Ten studies were discarded because they either did not measure repertoire complexity or studied fewer than 4 individuals. (c) We derived 134 measurements from the remaining 48 studies. (d) We discarded 43 measurements for the reasons listed. (e) The full dataset contained 43 studies (42 papers plus one thesis), 91 measurements, and 27 species

We compiled our list of references in three stages (Workflow in Figure 2):

Stage 1) We obtained field studies that examined the link between individual song elaboration (number of songs or syllables) and reproductive success (reproductive output or mating success) from the references included in the reviews by Byers and Kroodsma (2009) and Soma and Garamszegi (2011). Additionally, we searched for relevant studies published since these reviews using the terms “bird” and “song complexity,” “song versatility,” or “repertoire” in combination with “mating success,” “reproductive success,” or “mate choice” in Google Scholar, Web of Science, and ProQuest Dissertation and Theses Global database, which yielded eleven more field studies and one thesis with data that was unpublished at the time of data acquisition. This led to a total of 57 studies and 1 thesis. However, 10 studies were discarded, because they correlated reproductive success with aspects of song other than elaboration as we defined it. In the studies that remained, individual song elaboration was measured by either song repertoire size (unique number of songs per individual) or syllable repertoire size (unique number of syllables per individual). We included studies that measured the association between reproductive success and either of these song elaboration metrics, because syllable repertoire size and song repertoire size are correlated between species (Figure S1 in Appendix S3) and are likely also correlated within species. For information regarding which studies correlated what form of song elaboration with reproductive success in which species, see columns 1–3 of Table S1 in Appendix S3.
Stage 2) We performed a literature search using PubMed, Web of Science, and Google Scholar to gather information on the average syllable repertoire size for each species identified in Stage 1. Species syllable repertoire was defined as the average number of distinct syllables produced across individuals (Snyder & Creanza, 2019). Although many of the studies gathered in Stage 1 examined the correlation between syllable repertoire size and reproductive success, they often did not report the average species syllable repertoire size, so we searched for other sources (source per species noted in brackets in the supplementary data; list of bracketed references present in Appendix S3). Studies with average species syllable repertoires were found using the following search terms: Passeriformes or [species name] in combination with “song syllables,” “song complexity,” and “syllable repertoire.” For four species in the full dataset, information on the average species syllable repertoire size could not be found or was ambiguous, so we manually counted the unique syllables sung by individual birds using sonograms of song recordings from xeno-canto.org, and we were able to calculate the average number of syllables across individuals for two of these species (see methods, xeno‐canto citations, Tables S2–S3, and Figures S2–S3 in Appendix S3).
Stage 3) We performed another literature search to gather information on the length of the song‐learning window, using song stability over time as a proxy. Species that sang new syllables after sexual maturity—either by exchanging an old syllable for a novel one or by incorporating a new addition—were considered to have plastic songs. Studies with information about song stability were found using the following search terms: [species name] or [common name] in combination with “open‐ended,” “close‐ended,” “closed‐ended,” “age‐limited,” “crystal*,” “adult learning,” and “song changes.” Information on the song stability of several species was not available (Table S4 in Appendix S3). Information on the species average syllable repertoire size existed for all species for which we found information regarding song stability.

### 2.2 Dataset formation

From the 48 field studies that remained, we compiled 134 relevant measurements (Table S1 in Appendix S3). These commonly used measurements of reproductive success were categorized as follows:

Number of females: how many social mates a male attracts, where males who attract more females are assumed to be more successful.
Latency to pairing date or laying date: these two measures are traditionally used as a measure of reproductive success, because attractive males should pair first, and those who produce offspring sooner have a better chance of parenting a larger brood (Verhulst & Nilsson, 2008) or more than one brood of offspring in a single breeding season (Middleton, 1979; Murphy, 1983, 1986; Newton & Marquiss, 1984; Pinkowski, 1977). Chicks born earlier in the season also tend to be more viable (Catchpole & Slater, 2003; Murphy, 1986).
Extrapair paternity: this is often considered a metric of reproductive success because males that sire offspring in extrapair matings are assumed to be more attractive to females. However, it has been suggested to be an unreliable metric; see (Garamszegi, 2004; Petrie & Kempenaers, 1998; Soma & Garamszegi, 2011) and Table S5 and Supporting Information for results in Appendix S3.
Clutch size or number of offspring/recruits: these three measures are affected by both male and female genetic quality; however, it has been shown that females exposed to more elaborate songs can respond by producing larger clutches (Kroodsma, 1976), so male song quality can also potentially affect this metric. The number of offspring or number of recruits (offspring that return to the parental territory) is related to the genetic fitness of males and females, but also to parental investment.

The meta‐analytic mean for each of these metrics of reproductive success can be viewed in Table S5 in Appendix S3. When we group these metrics into different aspects of reproductive success, we use number of females and latency to pairing/laying date as proxies for social mate choice, extrapair paternity as a proxy for genetic mate choice, and clutch size or number of offspring/recruits as proxies for male reproductive output (see Appendix S3 for results).

Measurements of the correlation between individual song elaboration and reproductive success (as defined above) that were not Pearson's correlation coefficient (*r)* values were converted into *r* values using standard methods (Friedman, 1968; Nachar, 2008; Wolf, 1986); see also Data S1 for our exact calculations. Because negative correlations indicate a stronger relationship between individual song elaboration and reproductive success in latency to reproduction measurements, all latency measurements were multiplied by − 1 (as in (Scordato, 2018)). We converted *r* values into Fisher's *Z* values via Fisher's *r*‐to‐*Z* transformation, because Fisher's *Z* values have normally distributed variance—a criterion for variance estimation in the meta‐analysis. This transformation leads to a slight positive bias, so we applied the recommended transformation prior to conversion to correct for that bias (Field & Gillett, 2010). In total, 43 measurements were removed from the analysis for the reasons covered in Figure 2 and are labeled by their reason for exclusion in Table S1. We created three primary datasets:

Full dataset: includes all species for which we found measurements that correlated individual song elaboration with reproductive success even if we could not obtain information on the species average syllable repertoire size or song stability (91 correlation measurements, 27 species).
Species average syllable repertoire dataset: subset of the species for which we found or counted species average syllable repertoire sizes (86 correlation measurements, 25 species).
Song stability dataset: subset of the species for which we found information on song stability (77 correlation measurements, 20 species).

See Table S4 in Appendix S3 for the list of species in each dataset. We tested for funnel plot asymmetry in all three datasets using Egger's regression test (regtest; R package metafor) (Viechtbauer, 2010) and ranked correlation test (ranktest; R package metafor) (Song, Khan, Dinnes, & Sutton, 2002). Some studies included measurements of the correlation between individual song elaboration and reproductive success before and after controlling for territory quality or other factors. In these cases, we used the measurements that did not control for other factors in the primary datasets. We created and tested secondary datasets which included the territory‐controlled values in place of the noncontrolled values.

### 2.3 Random effects meta‐analysis

For our initial meta‐analytic assessment of the data, we performed a series of random effects meta‐analyses, as has been done in this field in the past (Soma & Garamszegi, 2011). We report these analyses in Appendix S1: Random Effects Meta‐analysis for full disclosure of all statistical tests used. However, the major caveats of this analysis were (a) that it required discarding a significant portion of the data, (b) that the remaining data needed to be analyzed separately by measurement type, and (c) that this style of meta‐analysis does not control for factors such as phylogenetic relatedness or nonindependence of multiple measures from the same species.

### 2.4 Bayesian multilevel phylogenetic meta‐analysis

We next analyzed the data as a multilevel phylogenetic meta‐analysis using the R package MCMCglmm (Hadfield, 2010). In this package, group differences are assessed by adding them as fixed effects. Random effects can be added to account for heterogeneity and nonindependence of data as sources of variance as well as phylogenetic relatedness. These complex models can be assembled because the program uses a Markov chain Monte Carlo simulation to estimate the amount of variance each of these parameters explains. We chose to include four random effects in our meta‐analytic model based on the structure of our data and the best practices suggested in the literature (Nakagawa & Santos, 2012; Pastor & Lazowski, 2017; Wilson et al., 2010):

MType: effects due to the metrics of reproductive success (measurement type) used in a given study
Phylo: effects due to the phylogenetic differences of species studied
Species: effects due to nonphylogenetic differences between the species studied
Study: effects due to nonindependence of measurements coming from a given study

Because no study investigated more than one population, differences caused by assessing different populations of the same species would be captured in the Study random effect. We also included a term for the standard error, which was calculated using the standard equation for Fisher's *Z*. In this model, the user must hypothesize the amount of variance that each random effect accounts for (prior) and assign a confidence (*nu*) to this hypothesis before running the simulation. The priors for MType, Species, and Study were set to:

$$\frac{\text{Variance}\;(\text{correlations between individual song elaboration and reproductive success})}{(\text{number of random effects + 1)}}$$

We used a low confidence *nu* set to one (see, e.g., Supplement file 5 of Wilson et al., 2010). The prior for standard error was fixed at the values we calculated from the Fisher's *Z*s. The prior for the Phylo was set by passing a species relatedness matrix—which was calculated as described in the next section—to the ginverse argument of MCMCglmm. To examine the amount of variance each random effect accounted for, we tested a series of models wherein each random effect was included alone or in combination with the others for each of our fixed effects and calculated the heterogeneity as described previously (Nakagawa & Santos, 2012). Ultimately, we included all variance terms, because inclusion of all terms led to a markedly lower deviance information criterion (DIC) for all meta‐analytic models (Table 1 and Tables S6–S25 in Appendix S3).

**Table 1:** Population variance in the song stability dataset

Random	*I* ^2^ (%)	DIC
Species	53.52	17.03
MType	39.35	33.82
Study	62.89	−7.63
Species	11.78	12.05
Phylo	20.28	
MType	21.24	−27
Study	43.5	
MType	33.17	−9.74
Species	6.19	
Phylo	12.16	
Study	42	−24.5
Species	4.78	
Phylo	8.39	
MType	22.7	−44.37
Species	4.05	
Phylo	7.61	
Study	19.32	

Note

Different sources of variance and nonindependence in the data were added to the model as random effects terms alone and in combination with the others. MType encodes the variance due to the metric of reproductive success used to generate each measurement. Study indicates variance accounted for by studies that reported multiple measurements. Phylo accounts for the effects of phylogeny, while Species encompasses all remaining species‐related effects. Percent variance (heterogeneity, *I* ^2^) was calculated by dividing the mean estimated variance by the total variance in the data. (Nakagawa & Santos, 2012). DIC stands for deviance information criterion.

We set the fixed effect in the models as either the full population (i.e., Fisher's *Z* ~ 1), the natural log of species average syllable repertoire size (as a continuous variable), or song stability (subpopulations that were song‐stable or song‐plastic). All models were run for 200,000 iterations, with a burn‐in of 30,000 iterations and a thinning interval of 10 iterations. All tested models appeared to reach convergence, because they were well mixed with peaks separated from zero (Appendix S2: Caterpillar Plots), and values for Gelman's $\hat{R}$ were < 1.1 (gelman.diag from R package coda) (Brooks & Gelman, 1998; Gelman & Rubin, 1992; Plummer, Best, Cowles, & Vines, 2006). The *p_MCMC_* is a measure of the fraction of runs that estimated a posterior mean greater than zero; the definition of significance evidence of an effect size above zero in these models is that > 95% of the MCMC runs estimate a posterior mean greater than zero (*p*_*MCMC*_ < 0.05). We performed an additional posterior predictive test on the continuous species average repertoire model to determine whether the model accurately predicted the correlation between song elaboration and reproductive success for the species tested.

### 2.5 BEST analysis

We tested for between‐group differences using “Bayesian Estimation Supersedes the *t*‐Test” (BEST) from the R package BEST. (Kruschke, 2013) This test returns the likelihood that the difference between the true means of two groups is greater than zero and gives a 95% credibility interval for the magnitude of this difference. We considered groups to be different if there was < 5% chance that there was no real difference, or this difference was in the opposite direction of what the meta‐analysis suggested.

### 2.6 Controlling for phylogenetic relationships

To control for phylogenetic effects, we performed our Bayesian meta‐analysis with a phylogeny that we generated from publicly available data. (Jetz, Thomas, Joy, Hartmann, & Mooers, 2012) Using a list of all species in this study, we extracted a set of 1,000 trees via the phylogeny subsets tool on birdtree.org. (Jetz et al., 2012) We included *Sayornis phoebe* as an outgroup to root the tree. We then created a consensus tree in R using the mean edge length method via the consensus.edges function (phytools package) (Revell, 2011) and converted it into a relatedness matrix for use in the Bayesian meta‐analysis with the inverseA function (MCMCglmm package). (Hadfield, 2010) Species not present in a dataset were dropped from the relatedness matrix before being passed to the MCMCglmm function.

## 3 RESULTS

### 3.1 Testing for publication bias and examining heterogeneity

**Figure 3:**
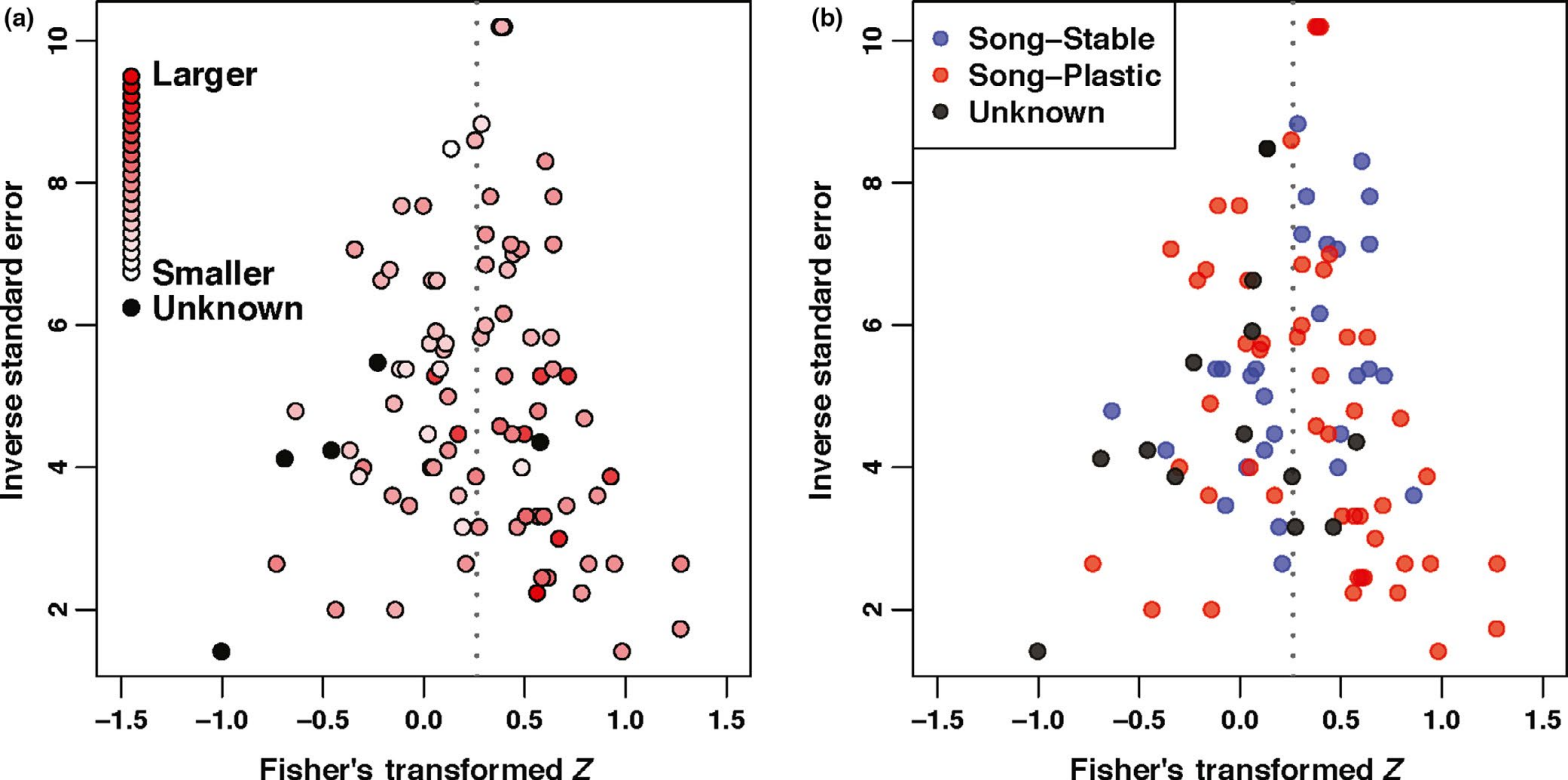
Funnel plot asymmetry. Funnel plots show the 91 measurements of the correlation between song elaboration and reproductive success from the full dataset. The gray dotted line represents the mean Fisher's transformed *Z*. (a) Circle color becomes more red as the repertoire size of the species increases. Black circles show measurements from species for which the syllable repertoire size is unknown. (b) Blue circles indicate measures from song‐stable species, while red circles indicate measurements from song‐plastic species. Black circles denote species for which no song stability information was available. Egger's regression testing on the full dataset (*z* = 0.9109, *p* = 0.3624), species average syllable repertoire dataset (*z* = 1.5555, *p* = 0.1198), or song stability dataset (*z* = 1.4782, *p* = 0.1394) revealed no significant funnel plot asymmetry. Ranked correlation testing on the full dataset (*τ* = 0.0227, *p* = 0.7523), species average syllable repertoire dataset (*τ* = 0.0539, *p* = 0.4667), or song stability dataset (*τ* = 0.0414, *p* = 0.5974) also revealed no significant funnel plot asymmetry

To assess the effect of publication bias on our datasets, we tested for funnel plot asymmetry using Egger's regression test and the ranked correlation test (Song et al., 2002) in all three datasets. These tests revealed no significant asymmetry (Figure 3), suggesting that publication bias did not significantly affect our data. We also found no evidence for publication bias when we used territory‐controlled values in place of noncontrolled values (Figure S4 in Appendix S3). We also examined the amount of variance that each of our random effect terms accounted for in our models for the full population. We found that the way that reproductive success was measured accounted for the most variance, while phylogeny and other species differences accounted for little variance (Table 1, also see Supporting Information Results and Tables S6–S25 in Appendix S3).

### 3.2 Assessing the relationship between song elaboration and reproductive success across all studies

We first estimated the meta‐analytic mean for correlation between individual song elaboration and reproductive success for the entire population (all species) using all three datasets (full, species average syllable repertoire, and song stability dataset). The posterior means of the models using the full dataset and the syllable repertoire dataset were not significantly separated from 0, whereas the model using the song stability dataset was weakly significantly separated from 0 (Full Dataset: Posterior Mean = 0.213, 95% CredInt = [−0.163;0.607], *p*_*MCMC*_ = 0.193; Species Average Syllable Repertoire Dataset: Posterior Mean = 0.242, 95% CredInt=[−0.021;0.521], *p*_*MCMC*_ = 0.067; Song Stability Dataset: Posterior Mean = 0.264, 95% CredInt=[0.021;0.53], *p*_*MCMC*_ = 0.042). We obtained similar results when we used our datasets that included territory‐controlled values in place of noncontrolled values (Table S26 in Appendix S3) or when we accounted for phylogenetic uncertainty (Table S27 in Appendix S3). While our results across all studies were largely not significant, we note that the magnitude of the estimated posterior mean of the effect sizes was similar to what was reported previously by Soma and Garamszegi (Soma & Garamszegi, 2011).

### 3.3 Testing the effect of species average repertoire size on the strength of the correlation between individual song elaboration and reproductive success

**Figure 4:**
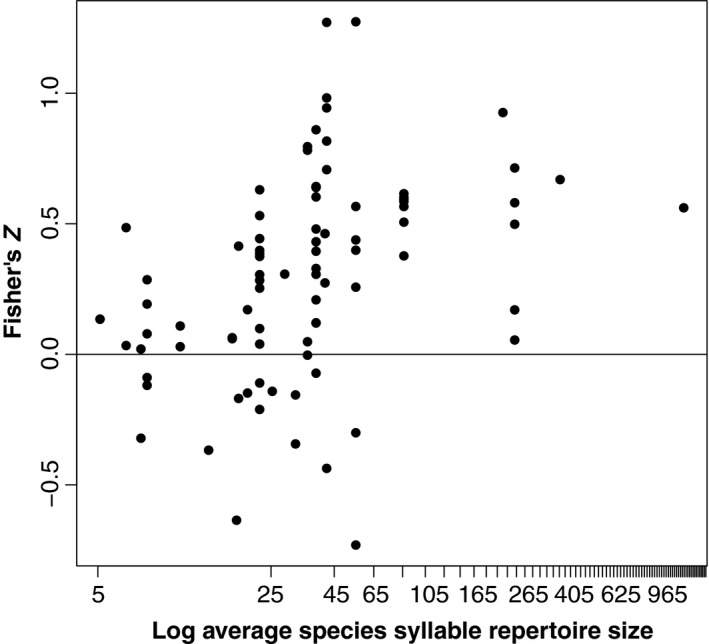
Effect sizes as repertoire size increases. We plot the effect sizes for studies used in our meta‐analysis as a function of the average syllable repertoire size of the species studied. For species with large repertoires, the effect sizes were generally positive, indicating a positive relationship between individual repertoire size and reproductive success; however, for species with small‐ to medium‐sized repertoires, studies found a wide range of effects, both positive and negative

**Figure 5:**
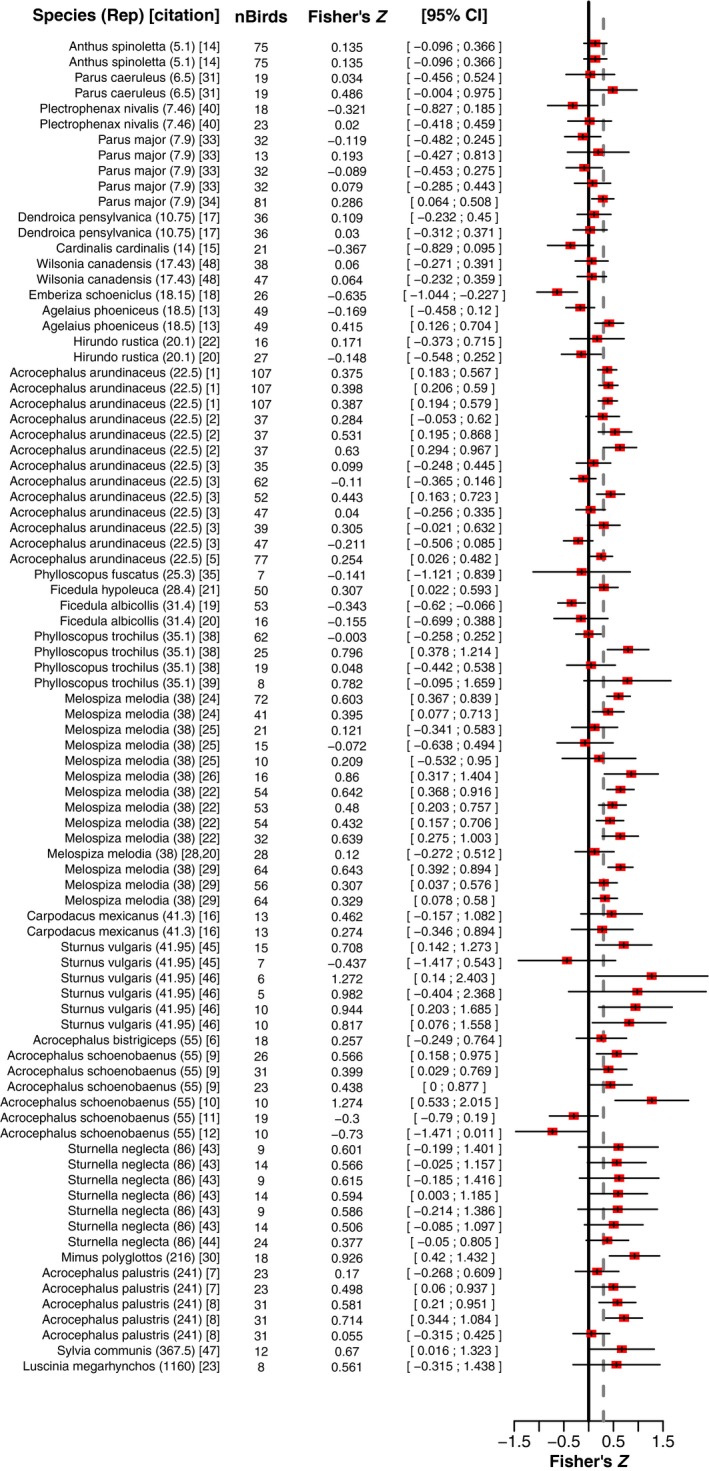
Forest plot of the species average syllable repertoire dataset. Columns show the individual studies and species studied, the number of birds used to generate a measurement, the Fisher's *Z* form of the estimate, and its 95% confidence intervals. Ticks in the boxes mark the Fisher's *Z*, and black horizontal lines show the confidence interval. The gray, dashed vertical line shows the population mean. When the same study is listed in more than one row on the plot, multiple different metrics of reproductive success were obtained from that study

To examine whether there was a linear relationship between average species repertoire size and the correlation between individual song elaboration and reproductive success, we tested the natural log of the species average syllable repertoire size as a continuous variable. The slope of this relationship was significantly greater than 0 (Intercept: Posterior Mean = −0.356, 95% CredInt = [−0.753;0.056], *p*_*MCMC*_ = 0.081; Slope: Posterior Mean = 0.167, 95% CredInt = [0.071;0.262], *p*_*MCMC*_ = 0.001) (Figures 4 and 5). Thus, for the 25 species studied here, this model predicts that the strength of the correlation between individual song elaboration and reproductive success grows modestly with every natural‐log increase in species average syllable repertoire size. The average syllable repertoires of the bird species studied here range from 5.1 syllables (ln(5.1) = 1.63) to over 1,160 (ln(1,160) = 7.06), so the model predicts that, when all other things are equal, the species with the smallest average syllable repertoires will show a very weak correlation, while species with very large average syllable repertoires will show moderate‐to‐strong correlations. However, we caution readers that this linear model also requires information for the random effects we included (e.g., phylogeny and the metric of reproductive success used) to predict values for new species; the slope and intercept presented here should not be used in isolation to make predictions about other species (see Figure 4 for the distribution of effect sizes when not accounting for the random effects). We next tested how well the data fit the model using a posterior predictive check. This analysis has the model predict the real correlations between individual song elaboration and reproductive success using all the predictor variables (random and fixed effects) for each measurement. We found that the model predicted the real correlations accurately, and no species or measurement appeared to deviate significantly from the model predictions (Figure S5 in Appendix S3).

To test the robustness of these results, we performed a jackknife analysis where each species was removed in turn. This did not significantly affect our results (Table S28 in Appendix S3): If any individual species was removed, we still observed a significant association. Furthermore, we tested whether these results were driven by the species with the smallest or largest average repertoires by excluding those species. The three species with the largest average syllable repertoires could be excluded and the model still predicted a significant relationship between species average syllable repertoire size and the correlation between individual song elaboration and reproductive success (Table S29 in Appendix S3). In addition, up to nine species with the smallest average syllable repertoires could be excluded, and the model still predicted a significant relationship (Table S30 in Appendix S3). For many species, there were multiple measures of species average syllable repertoire size reported in the literature, and we took the median value for the main analysis. It did not significantly affect our results when we used the maximum or minimum literature‐reported values instead of the median values (Table S31 in Appendix S3), used territory‐controlled values in place of noncontrolled values (Table S32 and Figure S6 in Appendix S3), or accounted for phylogenetic uncertainty (Table S27 in Appendix S3). This finding was also robust to methodological changes in which repertoire size was broken into discrete groups (see Supporting Information for results, Tables S33–S44 and Figures S6–S9 in Appendix S3).

### 3.4 Probing the differences between species with stable or plastic songs

**Figure 6:**
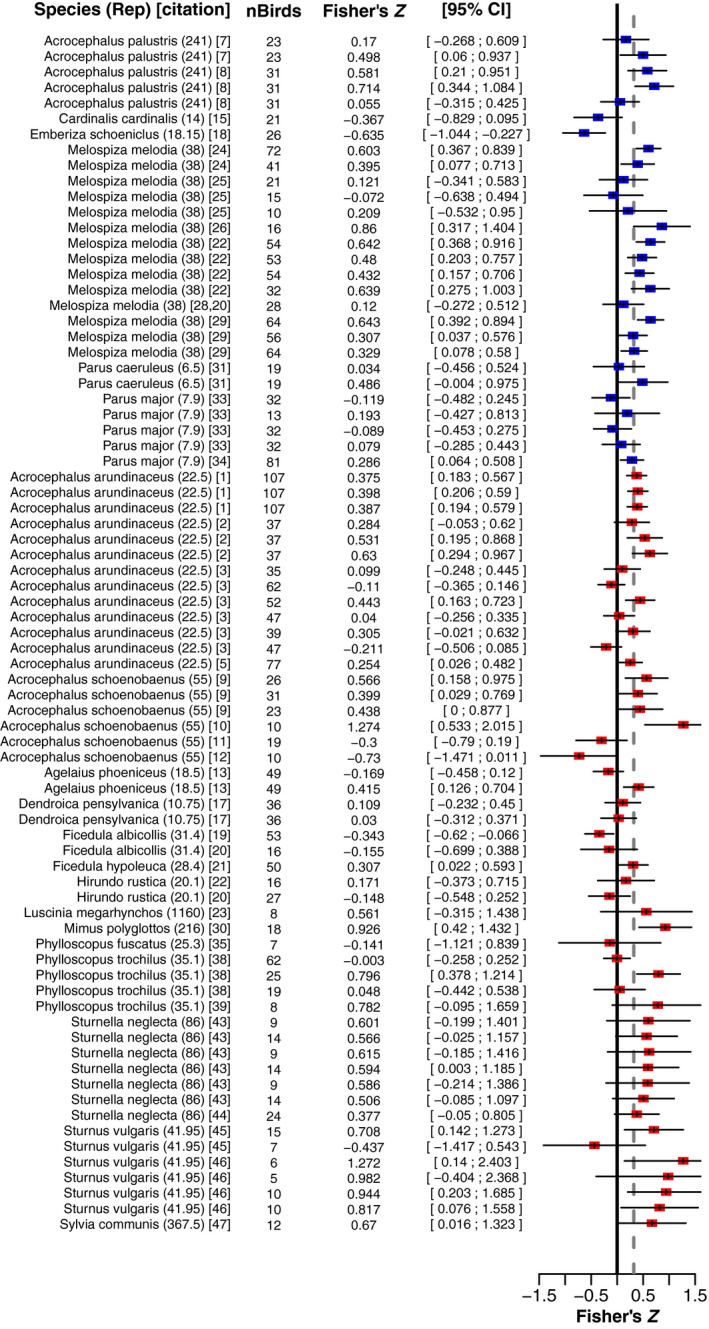
Song stability does not predict the presence or absence of a correlation between individual song elaboration and reproductive success. Meta‐analysis performed in the song stability dataset. Forest plot shows the individual studies and species studied, the number of birds used to generate a measurement, the Fisher's *Z* form of the estimate, and its 95% confidence intervals. Blue boxes mark measurements in the song‐stable group, while red boxes mark measurements in the song‐plastic group. Ticks in the boxes mark the Fisher's *Z*, and black horizontal lines show the confidence interval for each box. The gray, dashed vertical line shows the population mean. When the same study is listed in more than one row on the plot, multiple different metrics of reproductive success were obtained from that study

To examine whether song stability could predict the strength of the correlation between individual male repertoire size and reproductive success in a given species, we tested song stability as a fixed effect using the song stability dataset (Figure 6). The effect size for song‐stable species was not significantly separated from zero (Posterior Mean = 0.149, 95% CredInt = [−0.226;0.511], *p*_*MCMC*_ = 0.39). The effect size for song‐plastic species was predicted to be positive (Posterior Mean = 0.31, 95% CredInt = [0.034;0.594], *p*_*MCMC*_ = 0.028), but song plasticity did not appear to be a reliable predictor of the correlation between individual song elaboration and reproductive success. First, the song‐plasticity estimate was not strongly significant (*p*_*MCMC*_ = 0.028), particularly when we consider that we tested two independent hypotheses on our dataset. Second, the song‐plastic group's posterior mean and 95% credibility interval were qualitatively similar to those seen for the entire population, and its 95% credibility interval overlapped substantially with that for song‐stable species. Finally, we examined whether there was a difference between the song‐stable and song‐plastic groups using “Bayesian Estimation Supersedes the *t*‐Test” (BEST) analysis, and we did not find evidence for a significant difference between song‐stable and song‐plastic species (BEST_%<0_ = 21.7%, Mean Difference = 0.075, 95% CredInt = [−0.109;0.259]). In other words, song‐stable and song‐plastic species did not show a significant difference in their distribution of effect sizes. Thus, our results suggest that song stability may not be a species trait that can reliably predict the strength of the correlation between individual song elaboration and reproductive success. However, we note that we have few song‐stable species in our dataset (6), so this hypothesis should be re‐evaluated as more data become available. This discrete analysis of adult song plasticity may be difficult to directly compare to the continuous analysis of species average syllable repertoire, so we provide a discrete analysis of repertoire size in Appendix S3, which is concordant with our results from the continuous analysis.

We classified a species as song‐plastic if individual birds changed their repertoires over time. This included both species that increase their repertoire size with age, which have the potential to signal their age via their repertoire size, as well as species that replace old syllables with new ones and maintain a constant repertoire size, which does not signal their age. To address this, we repeated this analysis, reclassifying all studied species as those which do increase their repertoire size with age and those that do not. The reclassification scheme did not significantly affect our results (Table S45 and S46 in Appendix S3). Field studies examining song stability often examine a small number of birds, so it is possible that one small‐scale study might conclude that a species does change its repertoire when another study might conclude that the species does not (Rivera‐Gutierrez, Pinxten, & Eens, 2011). Therefore, we re‐assigned each species in turn to the opposite song‐stability classification. This did not significantly affect our results (Table S47 in Appendix S3). Using territory‐controlled measurements in place of noncontrolled measurements also did not significantly affect our results (Table S48, S49 and Figure S10 in Appendix S3). Finally, accounting for phylogenetic uncertainty also did not significantly affect our results (Table S27 in Appendix S3). Taken together, these results do not support the hypothesis that song stability can be used to predict the strength of the between individual song elaboration and reproductive success.

## 4 DISCUSSION

The relationship between reproductive success and song elaboration has long been proposed to exist (Catchpole, 1980, 1987; Howard, 1974; Kroodsma, 1976; Macdougall‐Shackleton, 1997; Nowicki et al., 1998; Pfaff et al., 2007; Reid et al., 2004; Searcy, 1984, 1992; Searcy & Andersson, 1986; Yasukawa et al., 1980), and, in laboratory settings, females have been observed to prefer large repertoires in mate‐choice tests (Baker et al., 1986; Byers & Kroodsma, 2009; Catchpole et al., 1984, 2010; Lampe & Saetre, 1995; Searcy, 1984, 1992; Soma & Garamszegi, 2011; Verheyen et al., 1991) and to lay more eggs in response to playbacks of larger repertoires (Kroodsma, 1976). However, analysis of available field data has not provided significant evidence for a strong correlation between reproductive success and song elaboration in nature (Byers & Kroodsma, 2009; Soma & Garamszegi, 2011), implying that sexual selection for more elaborate songs might only occur in a subset of species. Thus, there has been a long‐standing controversy over the putative link between birdsong elaboration, especially individual male repertoire size, and reproductive success, with some research claiming “elaborate songs […] are the acoustic equivalent of the peacock's tail” (Catchpole, 1987) and other research stating “it is unlikely that sexual selection for more elaborate songs is widespread among songbirds” (Byers, 2011). Here, we reconcile the contradictory interpretations in the literature by showing that the correlation between individual repertoire size and reproductive success is stronger in species with elaborate syllable repertoires than in species with simple repertoires. Unexpectedly, song stability did not provide predictive power regarding the correlation between song elaboration and reproductive success.

Our findings regarding repertoire size may initially appear circular: If we are testing studies that attempt to link larger individual song elaboration to increased reproductive success within a species, can larger species‐level syllable repertoires really be an independent signal of the strength of this link? We return to the parallel with sexual selection for tail length in birds (Figure 1), where species‐level exaggerated tail length could suggest that sexual selection is operating on this heritable trait within a species, predicting an individual‐level correlation between tail length and reproductive success. There are little data on the cultural and genetic heritability of repertoire size, but existing analyses indicate that repertoire size has strong phylogenetic signal (Crouch & Mason‐Gamer, 2019; Snyder & Creanza, 2019; Tietze et al., 2015). Sexual selection theory predicts that if females consistently choose males with more elaborate repertoires or if males with larger repertoires are more fecund, the distribution of repertoire sizes in a species may shift toward larger values over time.

Our results and the above prediction stand in contrast to the past hypothesis that species with smaller average repertoires might be more likely to exhibit a correlation between individual song elaboration and reproductive success, because it would be easier to discern differences in song elaboration between males when the species produces fewer syllables (Krebs, 1977; Krebs & Kroodsma, 1980). Why would these species not exhibit this preference in nature when they do in laboratory studies (Baker et al., 1986; Catchpole et al., 1984, 2010; Lampe & Saetre, 1995; Searcy, 1984, 1992; Verheyen et al., 1991)? Small average species repertoires are suggested to be more advantageous in contexts where dialects or song‐matching are an important facet of male–male competition (Beecher, Campbell, & Nordby, 2000; Catchpole, 1983; Konishi, 1985; Naguib, 1999; O'Loghlen & Rothstein, 1995). If males with larger repertoires tend to lose these competitions and are thus low in dominance hierarchies or cannot hold a territory, this would counteract female preferences for larger repertoires (Scordato, 2018). Our findings would not rule out a model wherein each bird species balances its average repertoire size between more elaborate songs optimized for attracting females and less elaborate songs optimized for other functions, such as individual recognition, territory defense, or other aggressive interactions, but further investigation would be required. Alternatively, if females have a stronger preference for traits reflecting song‐learning accuracy (Nowicki et al., 2002; Yasukawa, 2002), song performance (Ballentine, 2004; Hofstad, Espmark, Moksnes, Haugan, & Ingebrigtsen, 2002; Lyons, Beaulieu, & Sockman, 2014; Nowicki & Searcy, 2004) (trill length, note frequency, etc), or other exaggerated physical or behavioral traits (ornate plumage (Badyaev, Hill, & Weckworth, 2002; Soma & Garamszegi, 2018; Hill, 1991), lekking (Fiske, Rintamäki, & Karvonen, 1998; Maynard, 2012), dance (Byers, Hebets, & Podos, 2010)), these characteristics may be more indicative of male quality, and would thus have greater influence on mate choice in nature (Buchanan & Catchpole, 1997).

Alternatively, the measured association between individual song elaboration and reproductive success could hinge on the ability of repertoire size to act as an honest signal of male fitness (Nowicki & Searcy, 2005). Therefore, the species average repertoire would need to be large enough that inferior males cannot learn all of it. Large repertoires have been proposed to be costly to learn (Gil & Gahr, 2002) due to the metabolic costs of the neural underpinnings of song learning (Airey & DeVoogd, 2000; Devoogd et al., 1993; Pfaff et al., 2007) and because of the time and energy that must be dedicated to learning, practicing, and displaying the repertoire (Nowicki et al., 1998). Theoretically, small species average syllable repertoires would not lead to large resource requirements and would be less costly to learn. Thus, inferior males would be able to produce all species‐typical syllables. In this case, performance characteristics may be more indicative of male quality and eventual reproductive output than repertoire size in species with small species average syllable repertoires, as performance would likely still be affected by male quality (Hoi‐Leitner, Nechtelberger, & Hoi, 1995; Nowicki & Searcy, 2005).

We hypothesized that open‐ended learners would be more likely to show a correlation between individual song elaboration and reproductive success than closed‐ended learners, because open‐ended learners could potentially signal their age with their song. Extending the song‐learning window is expected to be metabolically costly (Beecher & Brenowitz, 2005; Creanza, Fogarty, & Feldman, 2016), so longer learning windows should be present only in species where there is selection for song traits that could benefit from extended learning windows, such as sexual selection for larger individual repertoires. Indeed, it has been suggested that adult song learning is associated with the evolution of larger repertoires (Creanza et al., 2016). However, we found that the strength of this correlation in species with plastic songs was not significantly different than in song‐stable species. It may be that open‐ended learning is beneficial in multiple contexts; in some species, males may increase their repertoire size over time to signal their age, whereas in other species song plasticity may assist in song‐matching and counter singing if male–male interactions are critical to reproductive success. Thus, song plasticity overall would not be predictive of a correlation between individual song elaboration and reproductive success. Narrowing our definition of song‐plastic species to those that increase their repertoire size with age did not yield significant results. However, our results from analyzing the interaction between species repertoire size and song stability allow us to cautiously propose that there may be an interaction between larger species average syllable repertoire size and song plasticity (see Supporting Information for results and Tables S50–S54 in Appendix S3). Further research will be required before we can conclude whether adult song plasticity can predict the strength of the correlation between individual song elaboration and reproductive success.

While these findings take an important step in elucidating the link between song elaboration and reproductive success, we note that this meta‐analysis was done with the goal of generating testable predictions for future field studies, which is by definition limited by the number of existing studies. Our meta‐analysis was performed on the relatively small number of species for which the correlation between individual elaboration and reproductive success was measured in the field. It remains to be seen whether these results will apply across all bird species, and we caution against making songbird‐wide generalizations from a meta‐analysis of relatively few species (Guolo & Varin, 2017). As more data are collected, it will also be important to investigate other factors that have been proposed to affect the strength of sexual selection in a species, such as polygyny, extrapair paternity, breeding synchrony, and migration behaviors (Birkhead & Biggins, 2010; Catchpole & Slater, 2003; Collins, Kort, Pérez‐Tris, & Tellería, 2009; Emlen & Oring, 1977; Freeman‐Gallant, Wheelwright, Meiklejohn, States, & Sollecito, 2005; Irwin, 2000; Mountjoy, James Mountjoy, & Leger, 2001; Read & Weary, 1992; Snyder & Creanza, 2019; Soma & Garamszegi, 2011; Vedder, Komdeur, Velde, Schut, & Magrath, 2011; Yezerinac & Weatherhead, 1997; see Appendix S3 for extended discussion).

To date, most research on the relationship between individual repertoire size and reproductive success has been conducted in species with small‐ to moderate‐sized syllable repertoires (see Figure 4). Currently, we have data from four species with average repertoires larger than 100 syllables; our observation of a significant relationship between species average repertoire size and the correlation between individual song elaboration and reproductive success persists if three of these four species are removed, but not if all four are removed. With more species surveyed at this higher end of species average repertoire size, we could better evaluate our observed trend. Thus, our results suggest that the field would particularly benefit from surveying more species with very large average repertoires to assess the relationship between individual song elaboration and reproductive success.

Here, we re‐evaluated the link between song elaboration and reproductive success through a Bayesian meta‐analysis of decades of field studies that integrates additional between‐species variables that may interact with sexual selection on individual song elaboration. Our meta‐analysis brings the results of these studies into sharper focus and proposes new hypotheses for future research to explore the origins and long‐term effects of sexual selection on elaboration in learned mating signals. We find that individual male song elaboration appears to be most correlated to reproductive success in species that have evolved unusually large syllable repertoires, potentially implying both past and ongoing sexual selection for larger individual repertoires in these species. If so, it will be important to consider the factors that initially drive the evolution of this elaboration and whether this trend is driven by mate choice and/or an association between individual song elaboration and reproductive output. These factors could include (a) the species‐specific importance of male traits for which song acts as an honest signal (e.g., health, developmental stresses, and song‐learning capacity), (b) species lifestyle and ecological niche traits (e.g., migratory status), and (c) tension between the importance of different uses of song. As more relevant variables are revealed, it will be possible to build better models to explain the different forces influencing sexual selection in song. Such models would be powerful tools not only for understanding bird species, but also for gaining insight into the behavioral and ecological forces that mediate the expression of sexually selected traits in different species.

## CONFLICT OF INTEREST

The authors declare no competing interests.

## AUTHOR CONTRIBUTIONS

C.R. and N.C. developed the ideas and designed the experiments. C.R. compiled the dataset, wrote the code, and analyzed data. C.R. and N.C. wrote the manuscript and approved it for publication.

## Data Availability

All data and associated code are available in Data S1 provided and at github.com/CreanzaLab/RepertoireSizeReproductiveSuccess.
