# Supplementary material for: Species‐level repertoire size predicts a correlation between individual song elaboration and reproductive success
Source: Ecol Evol. 2019 Jul 2;9(14):8362–77. doi: 10.1002/ece3.5418 (PMC6662282; doi:10.1002/ece3.5418)
Supplement: Supplementary file 1 [file ECE3-9-8362-s001.pdf]

Appendix A: Random Effects Meta-analysis for "Species-level repertoire size predicts a correlation between individual song elaboration and reproductive success"

Cristina Robinson and Nicole Creanza

Department of Biological Sciences, Vanderbilt University

<u>Contents</u>

**Random Effects Meta-analysis Methods**	**2**
**Random Effects Meta-analysis Results**	**5**
**References for Supplemental Methods and Results**	**17**
**Supplemental Figures**	**18**
**Supplemental Tables**	**28**

See "Supplemental References" in "Supplementary Information" for the list of studies referenced by [#] in various figures.

Random Effects Meta-analysis Methods

Dataset formation for the traditional meta-analysis

We gathered data as described in the main text for the Bayesian meta-analysis, but this traditional type of meta-analysis could not accommodate multiple measurement types from the same study, so we sorted the measurements into three categories depending on reproductive variable type as detailed below. In studies where *r* values were obtained for multiple variables belonging to one category, only one of these measures was included in the primary dataset, so that each study could contribute only one *r* value per category. Measurements were chosen for each category in descending order of the number of available measurements for each variable, setting the variable priority as follows: Latency to Pairing Date: 1) latency to pairing date, 2) latency to laying date, and 3) latency to hatching date (21 studies, 14 bird species); Number of Offspring: 1) number of fledglings, 2) clutch size, and 3) recruits (16 studies, 9 bird species); and Number of Females: 1) number of females and 2) measurements of extra-pair paternity (17 studies, 13 bird species).

Traditional meta-analysis

In addition to the Bayesian meta-analysis described in the main text, we conducted a more traditional meta-analysis. With the dataset containing species for which we had (a) studies of information on repertoire size and reproductive success, (b) data on repertoire stability (as a proxy for open vs. closed learning status), and (c) species-level repertoire size estimates, we performed a meta-analyses using the R packages `meta` and `metafor`, which use Hedges and

colleagues' method[1,2]. Additionally, we used the information in Birds of North America (birdsna.org) and Handbook of Birds of the World (hbw.com) to classify the migratory status of each bird species as sedentary, migratory, or mixed. Species that contained both sedentary and migratory individuals or populations (mixed) were labeled as migratory species in the final analysis to dichotomize the data. The literature on the role of song complexity in reproductive success argues against the possibility that there is a common effect size across bird species; therefore, a fixed effect model would be inappropriate for our dataset. Instead, and in accordance with Soma and Garamszegi[3], we used a random-effects model, which would not assume that there is a common effect size across species. We calculated heterogeneity using the restricted maximum likelihood method (REML), as this has been suggested to be an appropriate method for meta-analysis of both continuous and dichotomous data[4,5]. Six meta-analyses were performed, two for each of the three dataset categories, where the species studied in each category were divided into subpopulations: 1) species with larger syllable repertoires and species with smaller syllable repertoires based on a threshold of 38 syllables as described in the main text or 2) song-stable species and song-plastic species as a proxy for the length of the song-learning window. Because these data were tested twice to examine these two subpopulation variables, we used a Bonferroni-corrected significance threshold (α=0.025).

Threshold Testing

To determine the effect of the threshold value used to dichotomize species based on their average syllable repertoire size, we repeated the meta-analysis within each dataset category using each species' repertoire size as the threshold value in turn. The smallest repertoire threshold used was

the value at which at least two studies were present in the smaller repertoire subpopulation. The largest threshold was the value at which there were at least two studies in the larger repertoire subpopulation. For each threshold value, we calculated whether a smaller versus larger syllable repertoire predicted an association between song complexity and reproductive success. We used the same Bonferroni-corrected significance threshold for this procedure as in the meta-analyses (α=0.025).

Controlling for phylogenetic relationships

It was possible that our results would be confounded with phylogenetic relationships, since closely related species might have related behaviors and thus may not be independent samples. To control for phylogenetic effects, we performed a phylogenetically controlled ANOVA (phylANOVA) using R packages `maps`, `ape`, and `phytools`. Using a list of each species in a given dataset category, we extracted a set of 1000 trees via the phylogeny subsets tool on birdtree.org[40]. We then created a consensus tree in R using the mean edge length method via the consensus.edges function. Use of a species-level phylogeny required each species to be present only once in a dataset category. Therefore, we averaged the literature *r* values (the correlation between a given reproductive success category and individual male repertoire size) for each species that was represented by multiple studies in a dataset category. Literature *r* values were plotted as a continuous trait. The phylANOVA function was used to test for differences between song stability, species syllable repertoire size, and migratory status while controlling for phylogeny. A Bonferroni-corrected significance threshold was used (α=0.017 for 3 ANOVAs).

Interacting Variables

It was possible there would be an interaction between species syllable repertoire size and song stability. To examine this, we stratified the data in the latency to pairing date or number of offspring categories by song stability and compared the average literature r values between species with larger and smaller syllable repertoire sizes in song-stable versus song-plastic species via Welch's two sample t-test. We repeated this process with the subpopulation variables reversed. Four T-tests were performed, so we used a Bonferroni-corrected significance threshold (α=0.0125).

Jackknife Analysis

We performed a jackknife analysis to determine whether the potential miscategorization of a species' song stability could influence our results. Jackknife testing was performed first by changing the song stability grouping of one study at a time and repeating the meta-analysis to examine whether this regrouping changed the significance of the results. However, there were several cases where a single bird species was represented by multiple studies. Logically, if one of these species' song stability was miscategorized, this would change the grouping of all studies about that species. Therefore we ran a second jackknife, wherein regrouping was performed at the level of the bird species. We used the same Bonferroni-corrected significance threshold for this procedure as in the main meta-analyses (α=0.025).

Random Effects Meta-analysis Results

Traditional meta-analysis

To examine whether a male's individual repertoire size was correlated with only some indicators of reproductive success, we split the dataset into three categories: latency to pairing date (14 species, 21 studies), number of offspring (9 species, 16 studies), and number of females (13 species, 17 studies). We tested for a significant association between individual male repertoire size and reproductive success in each reproductive success category. The mean effect size for pairing date was significant (r=–0.37, I^2=74.7%, τ^2=0.1082, z=–4.47, p<0.0001) (Supplemental Figure SA1 A–B), as was the effect size for the number of offspring (r=0.35, I^2=74.6%, τ^2=0.1024, z=3.99, p=0.0001) (Supplemental Figure SA1 C-D). In contrast, number of females did not have a significant effect size (r=0.12, I^2=68.3%, τ^2=0.0968, z=1.22, p=0.22) (Supplemental Figure SA1 E-F).

However, the results of small, random-effects meta-analyses can be easily swayed by one or a few studies that are weighted more heavily in the analysis due to large sample sizes[6]. To examine whether any study in our analysis had this capability, we compared the range of weights in each category (W_{range}) to the weight value all studies would have if they were weighted equally (W_{equal}). W_{range} did not substantially extend W_{equal} for any category (pair date: W_{equal}=4.76, W_{range}=[1.8; 6.3]; offspring: W_{equal}=6.25, W_{range}=[3.6; 7.9]; females: W_{equal}=5.88, W_{range}=[2.6; 8.5]). Therefore, we concluded that the data would not be skewed by any individual study due to large sample size. Furthermore, some of the studies reported the correlation between reproductive success and repertoire size both before and after controlling for territory quality in the individual birds studied. We repeated these meta-analyses using a secondary dataset that exchanged non-territory-controlled measures with territory-controlled measures (only possible

for three measures), which only marginally affected the results (pair date: r=–0.34; offspring: r=0.36; females: r=0.10) (Supplemental Figure SA2).

Our results showed that while the effect size for each bird species is assumed to be different, the average effect size across the bird species in our study is small to moderate and negative for latency to pairing date, small to moderate and positive for number of offspring, and there is no correlation between repertoire size and the number of females a male acquires. It is important to note that for latency to pairing date a *negative* correlation indicates higher reproductive success (individual males with larger repertoires acquire mates faster), whereas for number of offspring a *positive* correlation indicates higher reproductive success (individual males with larger repertoires produce more offspring). These results are consistent with previous studies: there is a weak correlation between repertoire size and reproductive success across species. However, this overall weak correlation may mask a phenomenon in which repertoire size is correlated with reproductive success in some species, but not in others. Thus, we sought to discover whether aspects of species song-learning behavior could partition the full set of species into subsets with different patterns of sexual selection pressure on repertoire size. We propose two variables as candidates for creating these subsets: species syllable repertoire size and learning style (closed-ended vs. open-ended learning) proxied by song stability (stable songs vs. plastic songs).

Species Repertoire Size Meta-Analysis

Larger individual male repertoires may signal higher male intelligence, health, or fitness, decreased parasite load, increased access to resources, or other factors. Female preference for

larger repertoires could in turn create a selection pressure that increases the average syllable repertoire size of the species over time. Therefore, we first split the full population into two subpopulations based on each species' average syllable repertoire size, using less than 38 syllables as the threshold for smaller repertoire. Other possible thresholds were tested as explained below (Supplemental Figure SA5). We reran the meta-analysis on all three dataset categories with the bird species split into larger and smaller syllable repertoire groups, despite the fact that on a full population level there was not a significant average effect size for the correlation between an individual male's repertoire size and the number of females attracted. There was a significant difference in the effect sizes between species with larger versus smaller syllable repertoires for pairing date ($r_{smaller}$=–0.09, $I^2_{smaller}$=64.9%, $\tau^2_{smaller}$=0.0592, r_{larger}=–0.51, I^2_{larger}=41.9%, τ^2_{larger}=0.0262, χ^2=23.33, p=0.0004) (Supplemental Figure SA1A). While the average effect size for species with larger syllable repertoires was significant (z=–7.23, p<0.0001), the average effect size for species with smaller syllable repertoires was not (z=–0.77, p=0.44) (Supplemental Figure SA1A). There was also a significant difference in the effect sizes between species with larger versus smaller syllable repertoires for number of offspring ($r_{smaller}$=0.16, $I^2_{smaller}$=72.5%, $\tau^2_{smaller}$=0.0777, r_{larger}=0.59, I^2_{larger}<0.1%, τ^2_{larger}<0.0001, χ^2=15.26, p=0.0001) (Supplemental Figure SA1C). Once again, the effect size was only significant for species with larger syllable repertoires (z=9.29, p<0.0001) and not for species with smaller syllable repertoires (z=1.49, p=0.14) (Supplemental Figure SA1C). There was no significant difference between the effect sizes of species with larger versus smaller syllable repertoire for number of females ($r_{smaller}$=0.08, $I^2_{smaller}$=75.9%, $\tau^2_{smaller}$=0.1064, r_{larger}=0.12, I^2_{larger}=59%, τ^2_{larger}=0.1167, χ^2=0.16, p=0.69) (Supplemental Figure SA1E).

These data on our smaller set of species reveal that species syllable repertoire size may be able to be used as a metric to predict the importance of an individual male's repertoire size in reproductive success. The data predict that on average, species with larger syllable repertoire sizes sill show significant correlations between individual male repertoire size and reproductive success (pair $date_{confidInter}$=[-0.61; -0.39], $offspring_{confidInter}$=[0.49; 0.67]), while species with smaller syllable repertoires will not (pair $date_{confidInter}$=[-0.29; 0.13], $offspring_{confidInter}$=[-0.05; 0.36]) (Supplemental Figure SA1A, C and E). This is not to say that bird species with smaller repertoires would never show a correlation between individual male repertoire size and reproductive success, but rather the data suggest that that smaller average syllable repertoires are not predictive of such a correlation, while larger syllable average repertoires are.

It cannot be ignored, however, that our selection of a species repertoire threshold at 38 syllables might not be meaningful for the species in this study or species in general. Therefore, we reran the meta-analysis for all three categories with a range of threshold values, and compiled the *p*-values. If we had chosen a realistic threshold value, we would expect to see that repertoire threshold values near 38 syllables would also lead to significant group differences, while those that were further away from 38 syllables would not be significant, creating a U-shaped curve of *p*-values with a trough near 38 syllables.

Indeed, our analysis revealed this U-shaped *p*-value pattern for latency to pairing date and number of offspring (Supplemental Figure SA3A-B). On the other hand, for number of females

there had been no significant difference between species with larger and smaller syllable repertoires when using a threshold of 38 syllables (ln(38)=~3.6), and we did not expect any other threshold value to lead to significant results. Our expectations were confirmed; when the number of females dataset category was used to test alternative threshold values, no threshold value led to significant results (Supplemental Figure SA3C). The overall plot did not form a U-shaped distribution around 38 syllables, but rather the points were scattered randomly. To determine whether the threshold values for the latency to pairing date and number of offspring dataset categories were driven by the most positive or most negative correlations in a given dataset category, we repeated the analysis with either the two most positive or two most negative values omitted. These truncated category datasets created a similar U-shaped pattern to the full category datasets, which were centered around 38 syllables (Supplemental Figure SA3D-G). Therefore, it is unlikely that our threshold was shifted one way or another by unusually strong or weak correlations. These data suggest that 38 syllables is near the implied, real threshold value where a bird species has a large enough syllable repertoire that one can predict that there will be a correlation between individual male repertoire size and reproductive success.

Song Stability Meta-Analysis

In addition to species syllable repertoire size, we also tested whether length of the song-learning window had an association between song elaboration and reproductive success by using song stability as a proxy. For open-ended learners, repertoire size can be an indicator of age[25–27], and open-ended learning is correlated with larger species repertoire sizes[8]. Therefore, it is plausible that, in song-plastic species, individual male repertoire size is more important in female choice

than in song-stable species. Therefore, we hypothesized that elaboration would correlate with reproductive success more in species which show song plasticity in adulthood than in song-stable species. To address this, we next split our dataset into two groups based on song stability and reran the meta-analysis for all three categories of reproductive success. There were no significant differences between song-plastic species and song-stable species for any of the variables of reproductive success (pairing date: $r_{plastic}=-0.41$, $I^2_{plastic}=78.8\%$, $\tau^2_{plastic}=0.1935$, $r_{stable}=-0.39$, $I^2_{stable}=48.7\%$, $\tau^2_{stable}=0.0303$, $\chi^2=0.02$, $p=0.89$; offspring: $r_{plastic}=0.36$, $I^2_{plastic}=73.2\%$, $\tau^2_{plastic}=0.0968$, $r_{stable}=0.35$, $I^2_{stable}=77.1\%$, $\tau^2_{stable}=0.1276$, $\chi^2<0.01$, $p=0.95$; females: $r_{plastic}=0.12$, $I^2_{plastic}=60.6\%$, $\tau^2_{plastic}=0.0685$, $r_{stable}=0.18$, $I^2_{stable}=77.1\%$, $\tau^2_{stable}=0.1282$, $\chi^2=1.67$, $p=0.20$) (Supplemental Figure SA1B, D, F). These unexpected findings show that, in the species sampled here, song stability is not a meaningful metric in predicting the bird species for which individual male repertoire size is correlated with reproductive success.

Prediction Intervals

Our study was completed using a random-effects model; therefore, the population and subpopulation effect sizes, which are averages of the individual effect sizes in each species, are less meaningful than the prediction intervals around these averages, wherein the individual effect sizes of each constituent species would be expected to fall. For each category, the prediction interval for the full set of studies is very wide, encompassing predictions of positive, zero, and negative correlation coefficients. As expected, the prediction intervals for the song-plastic and song-stable subpopulations are similar to one another and similar to the full population for all categories (Supplemental Figure SA4A-C). For number of females, the prediction intervals for

the species with smaller and larger syllable repertoire subpopulations were similar to one another and to the full population (Supplemental Figure SA4C). In contrast, while the species with smaller syllable repertoire subpopulation prediction intervals are similar to the full population prediction intervals for latency to pairing date and number of offspring, the species with larger syllable repertoires subpopulation has a tighter prediction interval that indicates an association with reproductive success: a negative correlation for latency to pairing date and a positive correlation for number of offspring (Supplemental Figure SA4A–B). Together, these data predict that in any given species with a larger syllable repertoire size, there will be a small to large correlation between individual male repertoire size and reproductive success when measured by latency to pairing date or number of offspring produced. On the other hand, within the subpopulation of birds with smaller syllable repertoire sizes, larger individual repertoire size does not reliably correlate with these measures of success. However, it is important to note that prediction intervals can be calculated to be erroneously small when datasets sets are small[6]; the real prediction intervals for species with larger repertoires are likely wider than those presented here. It will be interesting to see whether the hypotheses suggested by this analysis will be supported or refuted as additional data becomes available.

Jackknife Analysis

It is possible that longitudinal study of repertoire size in a limited number of birds is not sufficient to accurately determine the song stability for that species, and thus we may have miscategorized a species' song stability. To account for this, we performed two regrouping jackknife studies. In one, we switched the song stability grouping for each individual study in

turn and reran the meta-analysis. In the other, we switched the grouping for all studies examining a given species, as each species was represented by 1 to 3 studies in each category. Neither the study jackknife for latency to pairing date nor the study jackknife number of offspring yielded any significant results (Supplemental Table SA1). For number of females, reclassification of two studies (Bucannon, and Catchpole 2000) [11] and (Marshal, Bucannon, and Catchpole 2007) [12] on *Acrocephalus schoenobaenus* to song-stable led to significant differences between groups pre-Bonferroni correction, but not post-Bonferroni correction (p=0.0432 and p=0.0419) (Note: bracketed references from the dataset can found in the **References for field studies** section in "Supplementary Information." In the species jackknife, re-grouping of any individual species did not consistently change our results, though regrouping of *Acrocephalus arundinaceus* led to significant results in number of offspring when reclassified as song-stable learners (p=0.0101), and regrouping of *A. schoenobaenus* in the number of females category led to significant results (p=0.006) (Supplemental Table SA24). It should be noted that there is unambiguous information available for the two mentioned *Acrocephalus* species: for *A. arundinaceus,* 56 individual males were studied for up to 5 years, and repertoires were found to change over time [1]. For *A. schoenobaenus,* 8 individuals followed within a single season increased repertoire sharing with their neighbors over time, and 6 individuals followed for up to 4 years showed changes in syllable repertoire size across years with a trend towards increasing repertoire size [40]. Furthermore, re-categorizing any single species did not change the results in more than one indicator of reproductive success. This makes us confident that these species were correctly grouped as song-plastic species. Together, these results suggest that it is unlikely that incorrect categorization of any one species is responsible for the lack of significance in the

original analysis of the song stability subpopulations. These data support our result that song stability by itself is likely not a metric that can be used to predict whether individual male repertoire size is correlated with reproductive success in a given bird species.

Possible Interactions

In this dataset, both song-stable and song-plastic species could have either smaller or larger species syllable repertoire sizes. Therefore, we wanted to examine whether there was an interaction between these two variables. We stratified the data in latency to pairing date and number of offspring by one of these subpopulations (e.g. song stability) and then tested for differences in the literature *r* values between the other subpopulation variable (repertoire size). Four Welch's two sample t-tests were performed on these data, so we used a Bonferroni-corrected significance threshold (α=0.0125). The only significant interaction was revealed in latency to pairing date, where song-plastic species with larger species syllable repertoires were significantly different from song-plastic species with smaller species syllable repertoires ($N_{Plasticlarger}$=7, $r_{Plasticlarger}$=–0.66; $N_{Plasticsmaller}$=6, $r_{Plasticsmaller}$=–0.11; t=–3.37, p=0.027) (Supplemental Table SA2). There was also an interaction in number of offspring that was trending significant, where song-stable species with larger species syllable repertoires were significantly different from song-stable species with smaller species syllable repertoires ($N_{Stablelarger}$=5, $r_{Stablelarger}$=0.59; $N_{Stablesmaller}$=4, $r_{Stablesmaller}$=–0.04; t=4.51, p=0.015) (Supplemental Table SA3). These data leave open the possibility that there is some interaction between song stability and repertoire size that yields greater predictive power, but larger sample sizes would be needed to make any definite conclusions.

Controlling for phylogenetic relationships

We used a phylogenetic ANOVA to test whether our results remained significant even when accounting for phylogeny. Soma and Garamszegi[3] had previously shown that controlling for phylogeny led to a poorer fit model when the data was restricted in this manner. However, their dataset was not broken into three separate reproductive success categories. Our results thus far indicated that song stability was not a meaningful predictor of the importance of individual male repertoire size in reproductive success. Interestingly, some research suggests that migratory birds and subspecies have larger average repertoires than their sedentary counterparts. Therefore, we performed three phylogenetic ANOVAs on our datasets: one for species syllable repertoire size, a second for song stability, and a third for migratory status. For latency to pairing date, species repertoire size produced significantly different subpopulations after correcting for phylogeny ($\text{Mean}_{\text{smaller}}=-0.123$, $\text{Mean}_{\text{larger}}=-0.577$, *p*-value=0.0068), while song stability did not ($\text{Mean}_{\text{plastic}}=-0.322$, $\text{Mean}_{\text{stable}}=-0.358$, *p*-value=0.88) (Supplemental Figure SA5A). On the other hand, for number of offspring, migratory status did lead to significantly different subpopulations ($\text{Mean}_{\text{mig}}=0.551$, $\text{Mean}_{\text{sed}}=-0.082$, *p*-value=0.007), species syllable repertoire was trending significant post-Bonferroni-correction ($\text{Mean}_{\text{smaller}}=0.118$, $\text{Mean}_{\text{larger}}=0.619$, *p*-value=0.039) and song stability was not significant ($\text{Mean}_{\text{Plastic}}=0.184$, $\text{Mean}_{\text{Stable}}=0.536$, *p*-value=0.18) (Supplemental Figure SA5B). Conclusions from these phylANOVAs need to be interpreted with caution, because the sample sizes are small, but the data suggest that species with larger syllable repertoires have larger correlations between individual male repertoire size and latency to pairing date than species with smaller syllable repertoires even after controlling for phylogenetic

relatedness. The correlations between individual male repertoire size and number of offspring were significantly different between species with larger syllable repertoires and smaller syllable repertoires after correcting for phylogenetic relatedness, but these differences did not remain significant after Bonferroni correction. Studies in more species will be required to definitively answer this question.

References for Supplemental Methods and Results

1. Hedges, L. V. How hard is hard science, how soft is soft science? The empirical cumulativeness of research. *Am. Psychol.* **42**, 443–455 (1987).
2. Field, A. P. & Gillett, R. How to do a meta-analysis. *Br. J. Math. Stat. Psychol.* **63**, 665–694 (2010).
3. Soma, M. & Garamszegi, L. Z. Rethinking birdsong evolution: meta-analysis of the relationship between song complexity and reproductive success. *Behav. Ecol.* **22**, 363–371 (2011).
4. Veroniki, A. A. *et al.* Methods to estimate the between-study variance and its uncertainty in meta-analysis. *Res Synth Methods* **7**, 55–79 (2016).
5. Nakagawa, S. & Santos, E. S. A. Methodological issues and advances in biological meta-analysis. *Evol. Ecol.* **26**, 1253–1274 (2012).
6. Guolo, A. & Varin, C. Random-effects meta-analysis: the number of studies matters. *Stat. Methods Med. Res.* **26**, 1500–1518 (2017).

Supplemental Figures

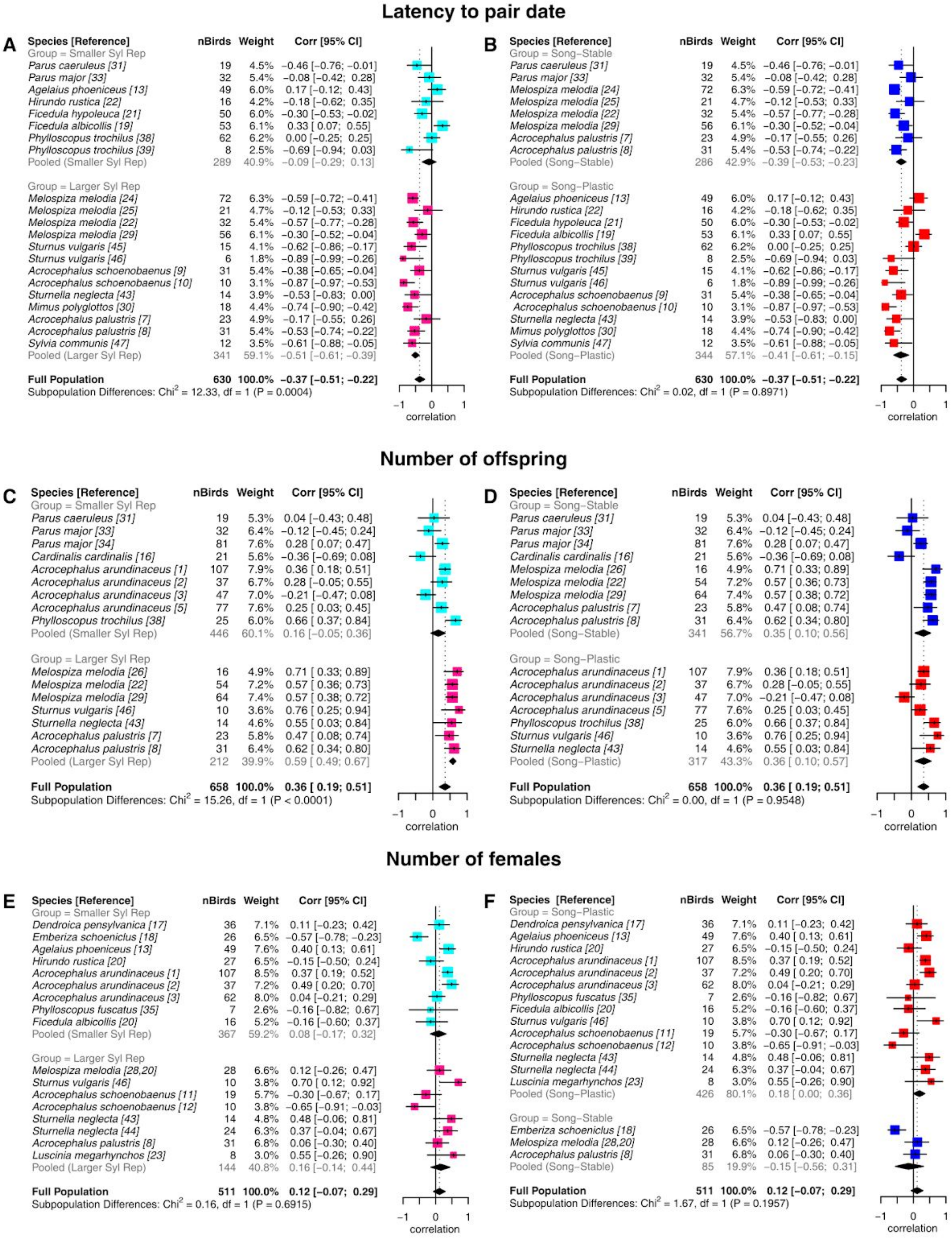

Supplemental Figure SA1: Large species repertoires are predictive of moderate to strong correlations between individual male repertoire size and reproductive success. Random effects model meta-analysis of the correlation between individual male syllable repertoire size and reproductive success with full model data present at the bottom of each analysis. The full population was split in half based on species syllable repertoire size or song stability. Because all datasets were tested twice, a Bonferroni-corrected significance threshold was used in determining significance (α=0.025). When latency to pairing date was used as the metric for reproductive success (A and B), more negative correlations indicate higher reproductive success. The full population had a significant effect size ($N_{pairdate}$=21, $r_{pairdate}$=–0.37, $I^2_{pairdate}$=74.7%, $\tau^2_{pairdate}$=0.1082, z=–4.47, p<0.0001). (A) There was a significant difference in effect size between species with smaller syllable repertoires and larger syllable repertoires ($N_{smaller}$=8, N_{larger}=13, $r_{smaller}$=–0.09, $I^2_{smaller}$=64.9%, $\tau^2_{smaller}$=0.0592, r_{larger}=–0.51, I^2_{larger}=41.9%, τ^2_{larger}=0.0262, χ^2=23.33, p=0.0004). The effect size was not significant for species with smaller repertoires ($r_{smaller}$=–0.09, z=–0.77, p=0.44). The effect size was significant for species with larger repertoires (r_{larger}=–0.54, z=–7.23, p<0.0001). (B) There was no significant difference in effect size between song-stable and song-plastic species (N_{stable}=8, $N_{plastic}$=13, r_{stable}=–0.39, I^2_{stable}=48.7%, τ^2_{stable}=0.0303, $r_{plastic}$=–0.41, $I^2_{plastic}$=78.8%, $\tau^2_{plastic}$=0.1935, χ^2=0.02, p=0.89). When number of offspring was used as the metric for reproductive success (C and D), more positive correlations indicate higher reproductive success. The full population had a significant effect size ($N_{offspring}$=16, $r_{offspring}$=0.35, $I^2_{offspring}$=74.6%, $\tau^2_{offspring}$=0.1024, z=3.99, p=0.0001). (C) There was a significant difference in effect size between species with species with smaller syllable repertoires and species with large syllable repertoires ($N_{smaller}$=8, N_{larger}=8, $r_{smaller}$=0.16, $I^2_{smaller}$=72.5%, $\tau^2_{smaller}$=0.0777, r_{larger}=0.59, I^2_{larger}<0.1%, τ^2_{larger}<0.0001, χ^2=15.26, p=0.0001). The effect size was not significant for species with smaller repertoires ($r_{smaller}$=–0.16, z=1.49, p=0.14). The effect size was significant for species with larger repertoires (r_{larger}=0.59, z=9.29, p<0.0001). (D) There was no significant difference in effect size between song-stable and song-plastic species (N_{stable}=9, $N_{plastic}$=7, r_{stable}=0.35, I^2_{stable}=77.1%, τ^2_{stable}=0.1276, $r_{plastic}$=0.36, $I^2_{plastic}$=73.2%, $\tau^2_{plastic}$=0.0968, χ^2<0.01, p=0.95). When number of females, was used as the metric for reproductive success (E and F), more positive correlations indicate higher reproductive success. The full population did not have a significant effect size ($N_{females=}$17, r=0.12, I^2=68.3%, τ^2=0.0968, z=1.22, p=0.22). (E) There was no significant difference in effect size between species with smaller syllable repertoires and larger syllable repertoires ($N_{smaller}$=7, N_{larger}=10, $r_{smaller}$=–0.09, $I^2_{smaller}$=64.9%, $\tau^2_{smaller}$=0.0592, r_{larger}=–0.51, I^2_{larger}=41.9%, τ^2_{larger}=0.0262, χ^2=23.33, p=0.0004). (F) There was no significant difference in effect size between song-stable and song-plastic species (N_{stable}=13, $N_{plastic}$=4, $r_{plastic}$=0.12, $I^2_{plastic}$=60.6%, $\tau^2_{plastic}$=0.0685, r_{stable}=0.18, I^2_{stable}=77.1%, τ^2_{stable}=0.1282, χ^2=1.67, p=0.20).

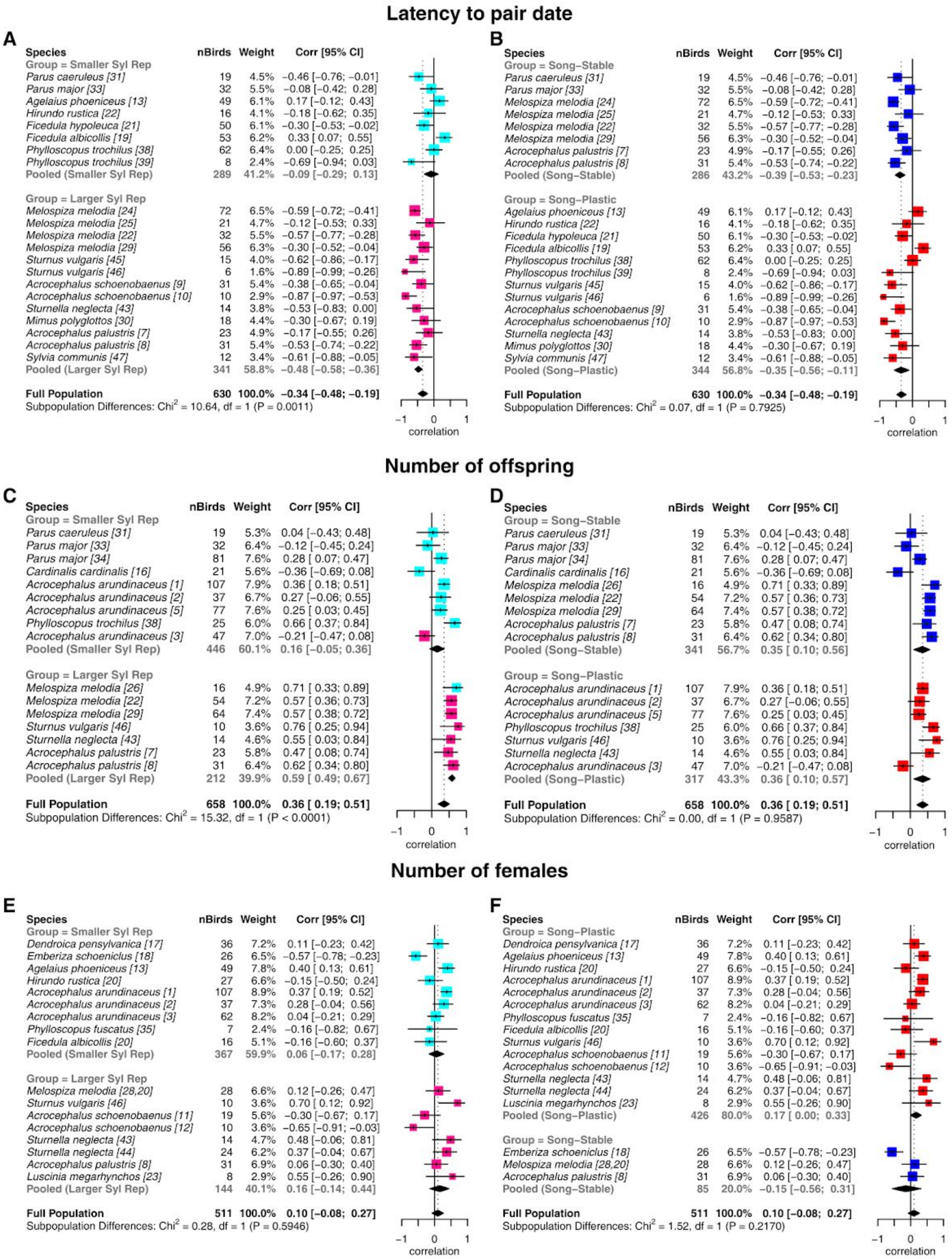

Supplemental Figure SA2: There are minimal differences when territory-quality controlled measurements are used. These meta-analyses were conducted identically to those explained in

Figure SA1, however three measurements were exchanged with territory controlled counterparts. When latency to pairing date was used as the metric for reproductive success (A and B), more negative correlations indicate higher reproductive success. The full population had a significant effect size ($N_{pairdate}$=21, $r_{pairdate}$=-0.34, $I^2_{pairdate}$=72.5%, $\tau^2_{pairdate}$=0.092, z=-4.32, p<0.0001). (A) There was a significant difference in effect size between species with smaller syllable repertoires and larger syllable repertoires ($N_{smaller}$=8, $N_{largerr}$=13, $r_{smaller}$=-0.09, I^2_{small}=64.9%, $\tau^2_{smaller}$=0.059, r_{larger}=-0.48, I^2_{larger}<35.9%, τ^2_{larger}<0.0.17, χ^2=10.64, p=0.0011). The effect size was not significant for species with smaller repertoires ($r_{smaller}$=-0.09, z=-0.77, p=0.44). The effect size was significant for species with larger repertoires (r_{larger}=-0.48, z=-7.23, p<0.0001). (B) There was no significant difference in effect size between song-stable and song-plastic species (N_{stable}=8, $N_{plastic}$=13, r_{stable}=-0.39, I^2_{stable}=48.7%, τ^2_{stable}=0.030, $r_{plastic}$=-0.35, $I^2_{plastic}$=74.9%, $\tau^2_{plastic}$=0.16, χ^2=0.07, p=0.79). When number of offspring was used as the metric for reproductive success (C and D), more positive correlations indicate higher reproductive success. The full population had a significant effect size ($N_{offspring}$=16, $r_{offspring}$=0.36, $I^2_{offspring}$=74.6%, $\tau^2_{offspring}$=0.10, z=3.38, p<0.0001). © There was a significant difference in effect size between species with smaller syllable repertoires and larger syllable repertoires ($N_{smaller}$=8, $N_{largerr}$=8, $r_{smaller}$=0.16, $I^2_{smaller}$=72.5%, $\tau^2_{smaller}$=0.078, $r_{largerr}$=0.59, I^2_{larger}<0.01%, τ^2_{larger}<0.01, χ^2=15.32, p<0.0001). The effect size was trending significant for species with smaller repertoires ($r_{smaller}$=0.16, z=2.03, p=0.0443). The effect size was significant for species with larger repertoires (r_{larger}=0.59, z=9.29, p<0.0001). (D) There was no significant difference in effect size between song-stable and song-plastic species (N_{stable}=9, $N_{plastic}$=7, r_{stable}=0.35, I^2_{stable}=77.1%, τ^2_{stable}=0.13, $r_{plastic}$=-0.35, $I^2_{plastic}$=73.3%, $\tau^2_{plastic}$=0.10, χ^2<.01, p=0.96). (E-F) When number of females was used as the metric for reproductive success, more positive correlations indicate higher reproductive success. The full population did not have a significant effect size ($N_{females}$=17, $r_{females}$=0.10, $I^2_{females}$=65.4%, $\tau^2_{females}$=0.082, z=1.11, p=0.27). (E) There was no significant difference in effect size between species with smaller syllable repertoires and larger syllable repertoires ($N_{smaller}$=9, N_{larger}=10, $r_{smaller}$=0.06, $I^2_{smaller}$=72.5%, $\tau^2_{smaller}$=0.085, r_{larger}=0.16, I^2_{larger}=59%, τ^2_{larger}=0.12, χ^2=0.10, p=0.75). (F) There was no significant difference in effect size between song-stable and song-plastic species (N_{stable}=13, $N_{plastic}$=4, r_{stable}=0.17, I^2_{stable}=77.1%, τ^2_{stable}=0.14, $r_{plastic}$=-0.15, $I^2_{plastic}$=56.4%, $\tau^2_{plastic}$=0.0505, χ^2=1.38, p=0.24).

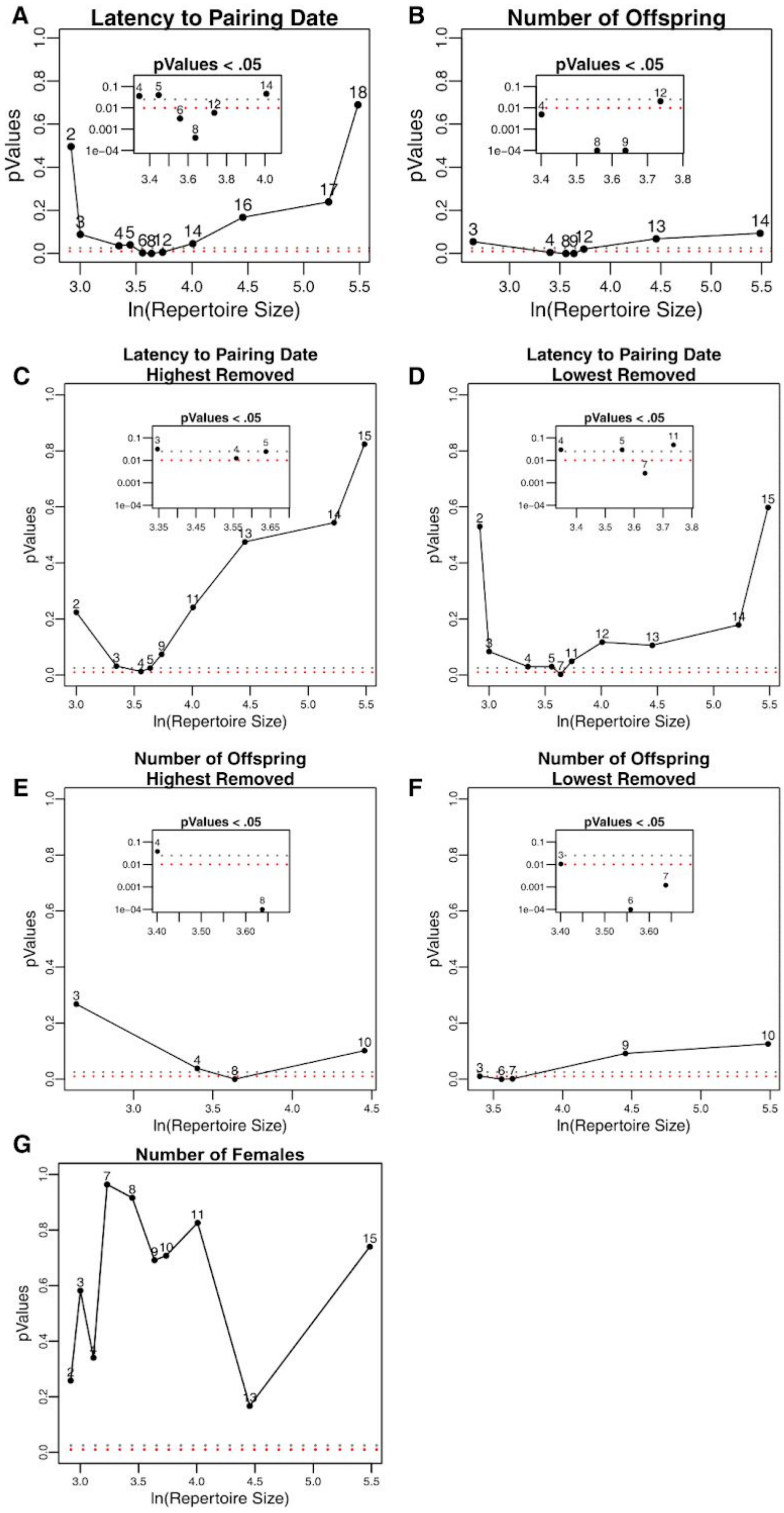

Supplemental Figure SA3: Plots of the *p*-values when different repertoire sizes are used as the threshold value. Insets show *p*-values that were less than 0.05 plotted on a $\log_{10}$ scale. In both the

main plot and the inset, the grey dotted line marks a significance threshold of 0.025, and the red dotted line marks a significance threshold of 0.01. Numbers above points are the number of studies/measurements present in the smaller repertoire group at that threshold value. Data were plotted from the latency to pairing date (A, C, D), number of offspring (B, E, F), or number of females (G) categories. (A, B, G) show the full data set. Other panels show the *p*-values when two most positive (C, E) or two most negative (D, F) correlations are removed from the dataset. The most significant repertoire size thresholds are around 38 syllables for pairing date and number of offspring, even when the most positive or most negative correlations are removed (A-F). Number of females instead show a random scattering of *p*-values (G).

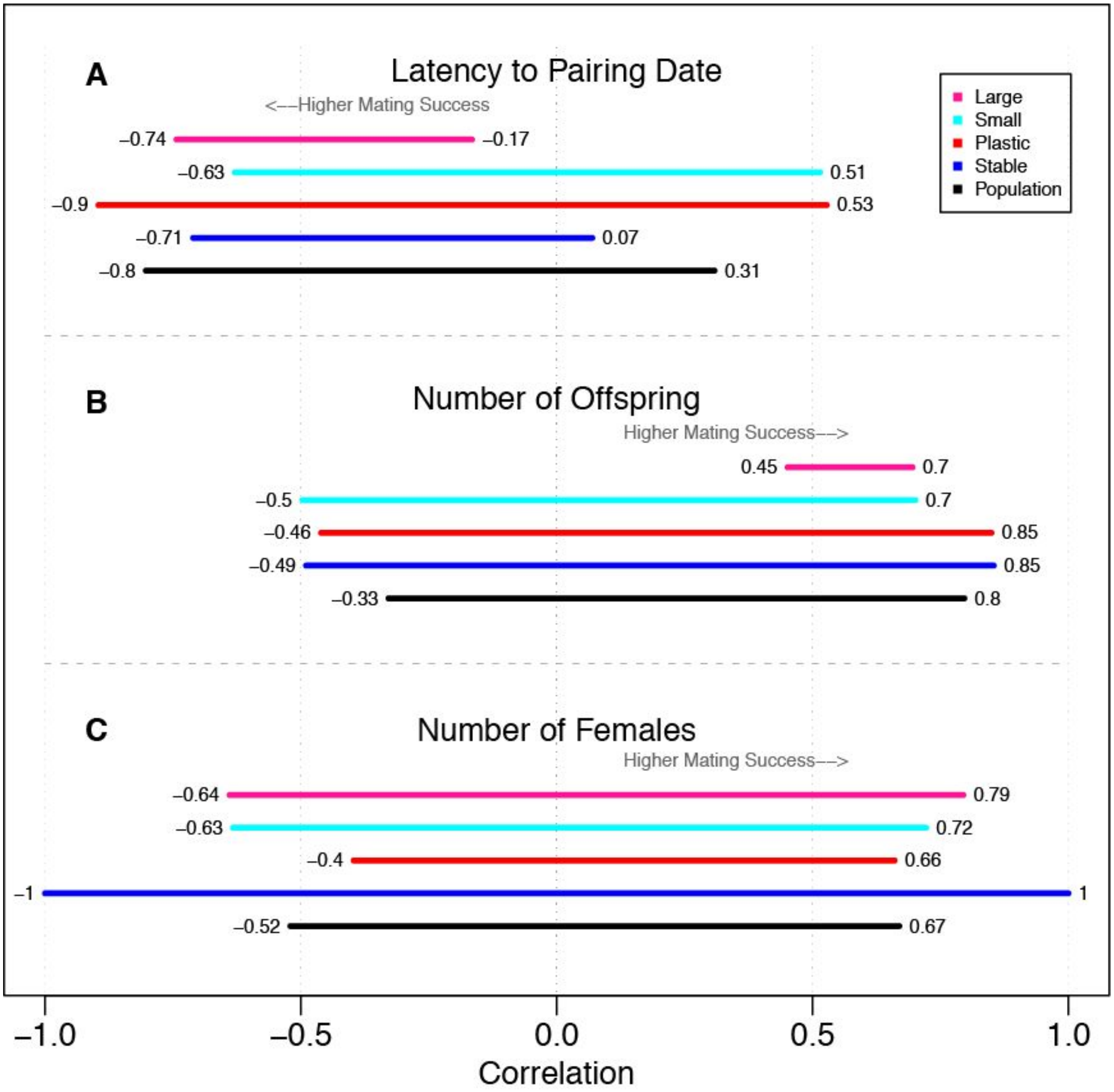

Supplemental Figure SA4: Only the group of species with larger repertoires has prediction intervals that are smaller than the full population prediction intervals. (A-C) Lines show the prediction intervals for the full population, the song stability groups, and species syllable repertoire groups for each of the reproductive success categories. (A) For latency to pairing date, all subpopulations display similar, wide prediction intervals to the full population except the species with larger repertoires group. This subpopulation comprises only the negative half of the correlation range (earlier pairing, higher reproductive success), with the expected correlations between individual male repertoire size and reproductive success for all members of this group ranging from small to larger (PI=[-0.17, -0.74]). (B) The same pattern exists for number of offspring, where only the species with larger syllable repertoires subpopulation is smaller than

the full population. This subpopulation comprises only the positive half of the population range, with the expected correlations between individual male repertoire size and reproductive success for all members of this group ranging from moderate to larger (PI=[0.45, 0.72]). (C) For number of females, there were no meaningful differences between the full population and any subpopulation.

A

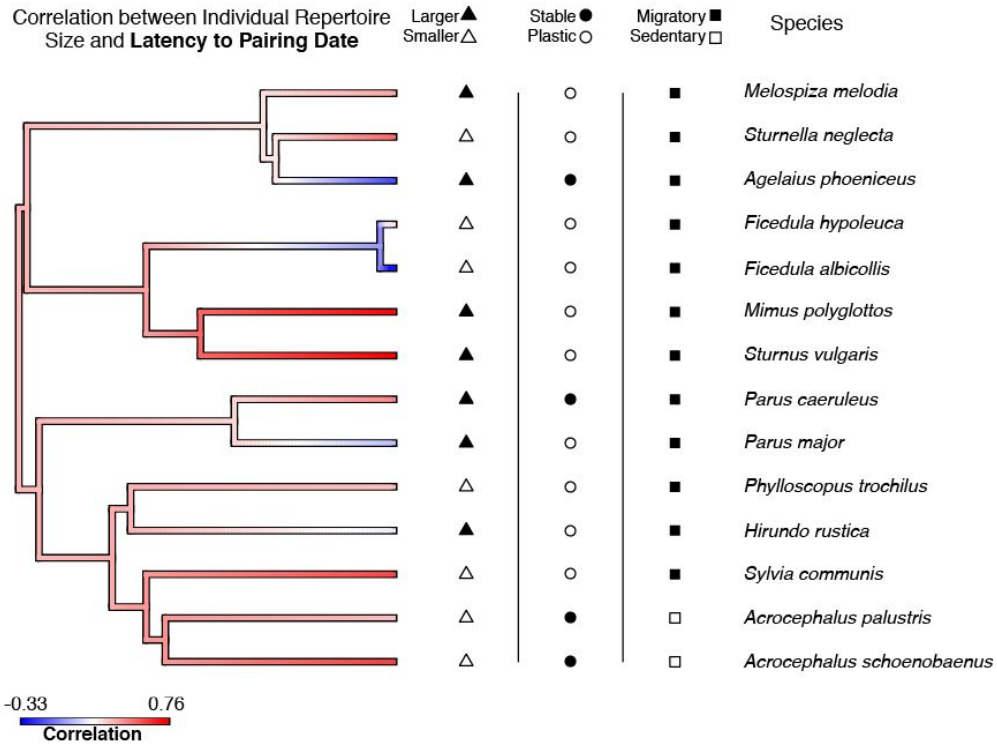

Phylogenically–Controlled ANOVA Results:
Bonferroni–Corrected Alpha = 0.017

Repertoire Size F–Value = 13.244 p–value = 0.0068 Means: Smaller: 0.123 Larger: 0.572
Learning Style F–Value = 0.032 p–value = 0.8818 Means: Stable: 0.358 Plastic: 0.322
Migratory Style F–Value = 0.123 p–value = 0.8042 Means: Sedentary: 0.271 Migratory: 0.36

B

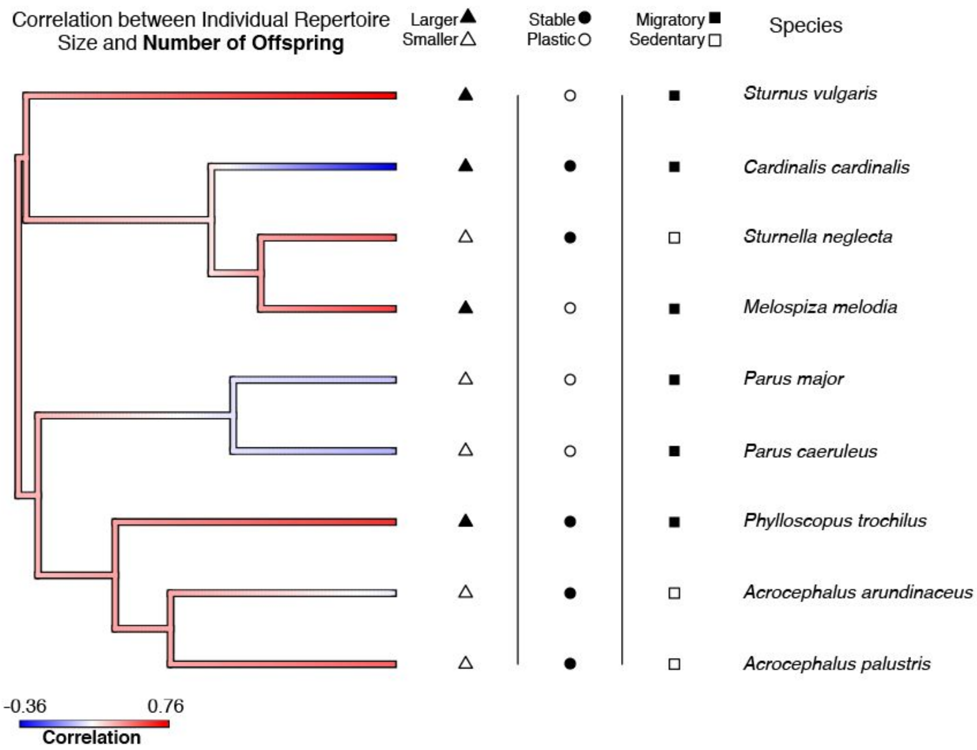

Phylogenically–Controlled ANOVA Results:
Bonferroni–Corrected Alpha = 0.017

Repertoire Size F–Value = 6.859 p–value = 0.0388 Means: Smaller: 0.118 Larger: 0.619
Learning Style F–Value = 2.276 p–value = 0.1688 Means: Stable: 0.536 Plastic: 0.184
Migratory Style F–Value = 17.289 p–value = 0.0066 Means: Sedentary: –0.082 Migratory: 0.551

Supplemental Figure SA5: Repertoire size subpopulation differences persist after controlling for phylogeny in a traditional meta-analysis. (A–B) Trees show the phylogeny between bird species and the continuous character map of the correlation between individual male repertoire size and either the latency to pairing date (A) or number of offspring (B). Triangles represent syllable repertoire size, circles represent song stability, and squares represent migratory status. Only for latency to pairing date (A), the correlations were multiplied by -1, so that redder colors indicate greater reproductive success on both trees. A Bonferroni-corrected significance threshold (α=0.017) was used for the following phylogenetic ANOVAs. The degrees of freedom for each test are the number of species minus one. (A) For latency to pairing date, there was a significant difference between the reproductive success correlation of smaller and larger species syllable repertoires after phylogenetic control ($Mean_{smaller}$=0.123, $Mean_{larger}$=0.572, F=13.2, p=0.0068). There was still no significant difference between song-stable and song-plastic after phylogenetic control ($Mean_{stable}$=0.358, $Mean_{plastic}$=0.33, F=0.032, p=0.88). There was no significant difference between sedentary and migratory birds after phylogenetic control ($Mean_{sed}$=0.271, $Mean_{mig}$=0.36, F=0.123, p=0.80). (B) For number of offspring, there was no significant difference after Bonferroni-correction between the reproductive success correlation of smaller and larger species syllable repertoires after phylogenetic control ($Mean_{smaller}$=0.118, $Mean_{larger}$=0.619, F=6.86, p=0.039). There was still no significant difference between song-stable and song-plastic species after phylogentic control ($Mean_{stable}$=0.54, $Mean_{plastic}$=0.184, F=2.28, p=0.17). There was a significant difference between sedentary and migratory birds after phylogenetic control ($Mean_{sed}$=-0.082, $Mean_{mig}$=0.551, F=17.23, p=0.007).

Supplemental Table SA1: Possible miscategorization of song stability for one study would not change the outcomes of the meta-analyses. The Bonferroni-corrected significance threshold (α=0.025) from the main meta-analyses was used to determine significance. The song stability (Song-plastic or song-stable) associated with each study was individually switched, and the meta-analysis was repeated to obtain a new p-value. Re-categorization of no one study led to significant results.

Latency to Pairing Date		Number of Offspring		Number of Females	
Ref	pval	Ref	pval	Ref	pval
[7]	0.8501	[1]	0.9131	[1]	0.5131
[8]	0.7322	[2]	0.8169	[2]	0.6326
[9]	0.8598	[3]	0.4206	[3]	0.1404
[10]	0.5815	[5]	0.7702	[8]	0.2665
[13]	0.371	[7]	0.8405	[11]	0.0432
[19]	0.29	[8]	0.6052	[12]	0.0419
[21]	0.7554	[16]	0.4333	[13]	0.5192
[22]	0.7124	[26]	0.551	[17]	0.1937
[24]	0.5371	[22]	0.6536	[18]	0.8605
[25]	0.8175	[29]	0.6462	[20]	0.0815
[22]	0.6704	[31]	0.7352	[20]	0.0621
[29]	0.9372	[33]	0.5448	[28;20]	0.1902
[30]	0.6921	[34]	0.905	[23]	0.4287
[31]	0.8463	[38]	0.5588	[35]	0.1249
[33]	0.6814	[43]	0.8691	[43]	0.498
[38]	0.461	[46]	0.6462	[44]	0.4319
[39]	0.9317			[46]	0.7164
[43]	0.9857				
[45]	0.9056				
[46]	0.7962				
[47]	0.9533				

Supplemental Table SA2: Possible miscategorization of song stability for one species would not change the outcomes of the meta-analyses. The Bonferroni-corrected significance threshold (α=0.025) from the main meta-analyses was used to determine significance. The song stability associated with each species was individually switched, and the meta-analysis was rerun to obtain a new p-value. Miscategorization of *Acrocephalus arudinaceus* (3 studies) would have led to significant results for number of offspring (p=0.0101). Miscategorization of *A. schoenobaenus* (2 studies) would have led to significant results for number of females (p=0.006). References numbers refer to supplemental references. Significant differences marked by asterisks.

Latency to Pairing Date		Number of Offspring		Number of Females	
Species	pval	Species	pval	Species	pval
Parus major	0.6814	*Parus major*	0.4444	*Emberiza schoeniclus*	0.8605
Agelaius phoeniceus	0.371	*Cardinalis cardinalis*	0.4333	*Agelaius phoeniceus*	0.5192
Hirundo rustica	0.7124	*Acrocephalus arundinaceus*	0.0101*	*Hirundo rustica*	0.0621
Ficedula hypoleuca	0.7554	*Phylloscopus trochilus*	0.5588	*Phylloscopus fuscatus*	0.1249
Ficedula albicollis	0.29	*Melospiza melodia*	0.1454	*Acrocephalus arundinaceus*	0.8945
Phylloscopus trochilus	0.6088	*Sturnus vulgaris*	0.6462	*Ficedula albicollis*	0.0815
Melospiza melodia	0.5903	*Sturnella neglecta*	0.8691	*Melospiza melodia*	0.1902
Sturnus vulgaris	0.6022	*Acrocephalus palustris*	0.5115	*Sturnus vulgaris*	0.7164
Acrocephalus schoenobaenus	0.6363			*Acrocephalus schoenobaenus*	0.006*
Sturnella neglecta	0.9857			*Sturnella neglecta*	0.7491
Mimus polyglottos	0.6921			*Acrocephalus palustris*	0.5773
Acrocephalus palustris	0.8502			*Luscinia megarhynchos*	0.1957
Sylvia communis	0.8971				

Supplemental Table SA3: Song stability may add some predictive value in combination with species syllable repertoire size. Data from the latency to pairing date or number of offspring categories were stratified by either song stability or species syllable repertoire size and then a Welch's two sample t-test was used to examine whether there was a difference in the other subpopulation variable. Because four t-tests were performed, a Bonferroni-corrected significance threshold was used (α=0.0125). The degrees of freedom for latency to pairing date were 11, 6, 11, and 6 in descending order. The degrees of freedom for number of offspring were 5, 7, 5, and 7 in descending order. Significant differences marked by asterisks.

Latency to Pairing Date						Number of Offspring					
Class	Group	N	Mean	t	pval	Class	Group	N	Mean	t	pval
Plastic	Large	7	-0.663	-3.37	0.012*	Plastic	Large	2	0.656	2.2	0.0874
	Small	6	-0.11				Small	5	0.268		
Stable	Large	6	-0.3812	-0.583	0.63	Stable	Large	5	0.5886	4.51	0.015
	Small	2	-0.271				Small	4	-0.041		
Large	Plastic	7	-0.663	2.58	0.027	Large	Plastic	2	0.6555	-0.05965	0.64
	Stable	6	-0.381				Stable	5	0.5886		
Small	Plastic	6	-0.271	-0.664	0.56	Small	Plastic	5	0.268	-1.59	0.156
	Stable	2	-0.11				Stable	4	-0.0412		
