# Supplementary material for: Species‐level repertoire size predicts a correlation between individual song elaboration and reproductive success
Source: Ecol Evol. 2019 Jul 2;9(14):8362–77. doi: 10.1002/ece3.5418 (PMC6662282; doi:10.1002/ece3.5418)
Supplement: Supplementary file 2 [file ECE3-9-8362-s002.pdf]

Appendix B: Caterpillar Plots for "Species-level repertoire size predicts a correlation between individual song elaboration and reproductive success"

Cristina Robinson and Nicole Creanza

Department of Biological Sciences, Vanderbilt University

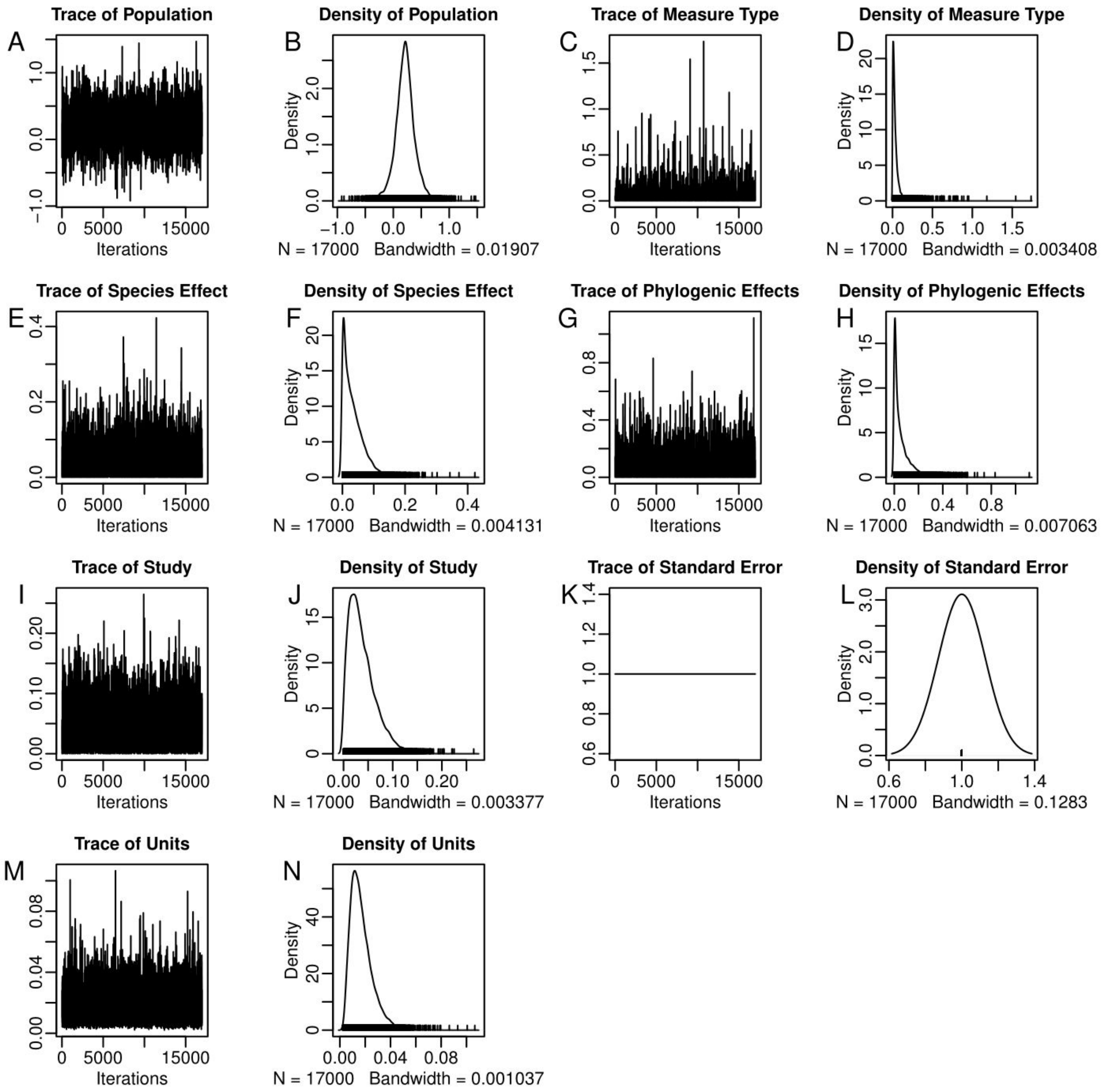

Supplemental Figure SB1: Convergence of parameters in the full population model for the full dataset. (A), (C), (E), (G), (I), (K), (M) show the trace of the estimated value in order of earliest model iteration to latest model iteration. (B), (D), (F), (H), (J), (L), (N) show the posterior distributions of (A), (C), (E), (G), (I), (K), (M). Tallies mark individual estimates. The first pair of plots is for the population fixed effect, while subsequent pairs show random effects. Standard error was estimated from the data itself rather than via the MCMC simulation, so its distribution was fixed at 1. Units represents the estimation of the residual variance. There is no visible trend in the traces that would suggest autocorrelation affected the simulation, and the traces for all terms appear well mixed. The density maps for all random effects terms peak above zero.

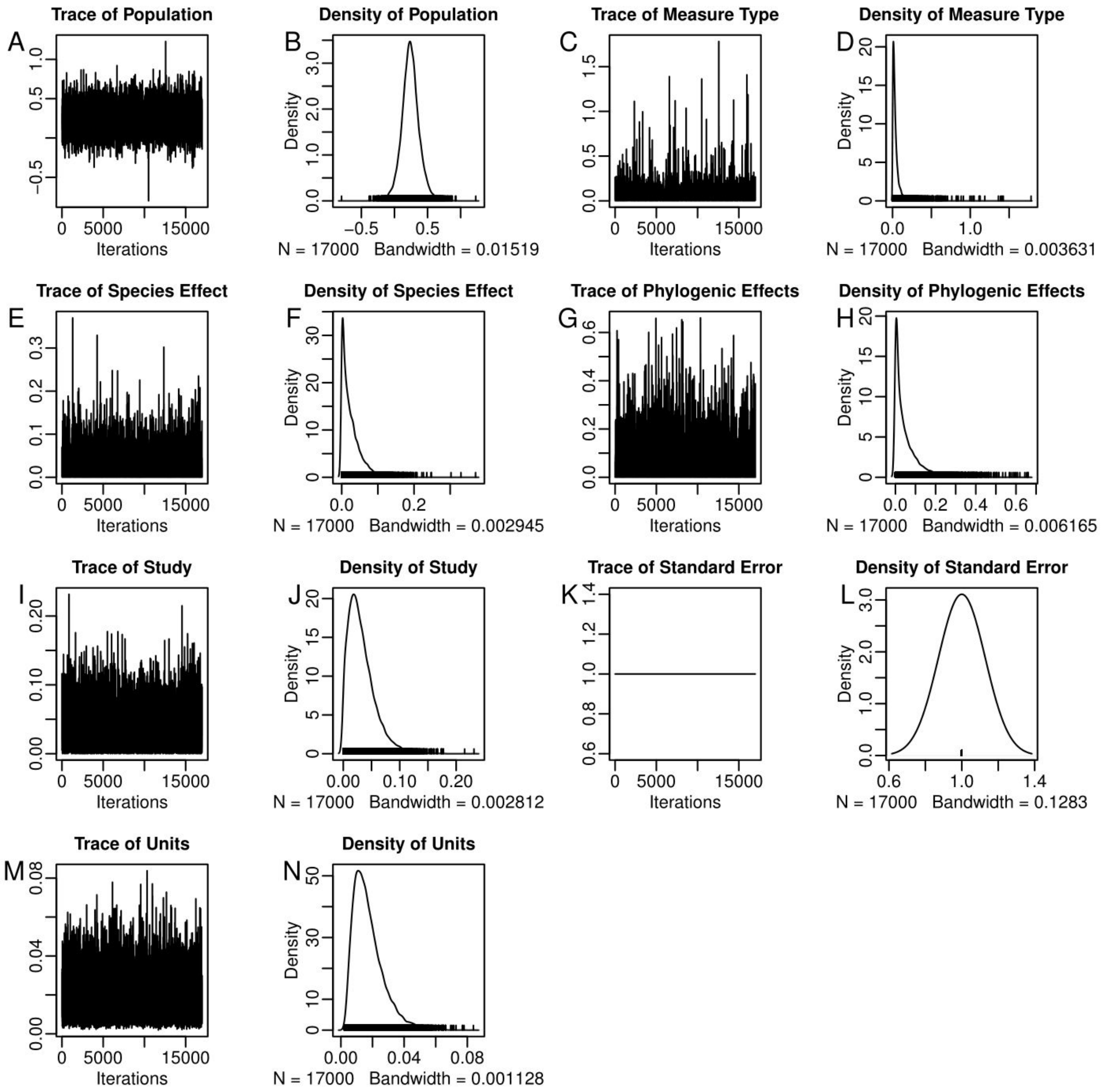

Supplemental Figure SB2: Convergence of parameters in the full population model for the syllable repertoire data set. Annotation is the same as in Supplemental Figure S13. There is no visible trend in the traces that would suggest autocorrelation affected the simulation, and the traces for all terms appear well mixed. The density maps for all random effects terms peak above zero.

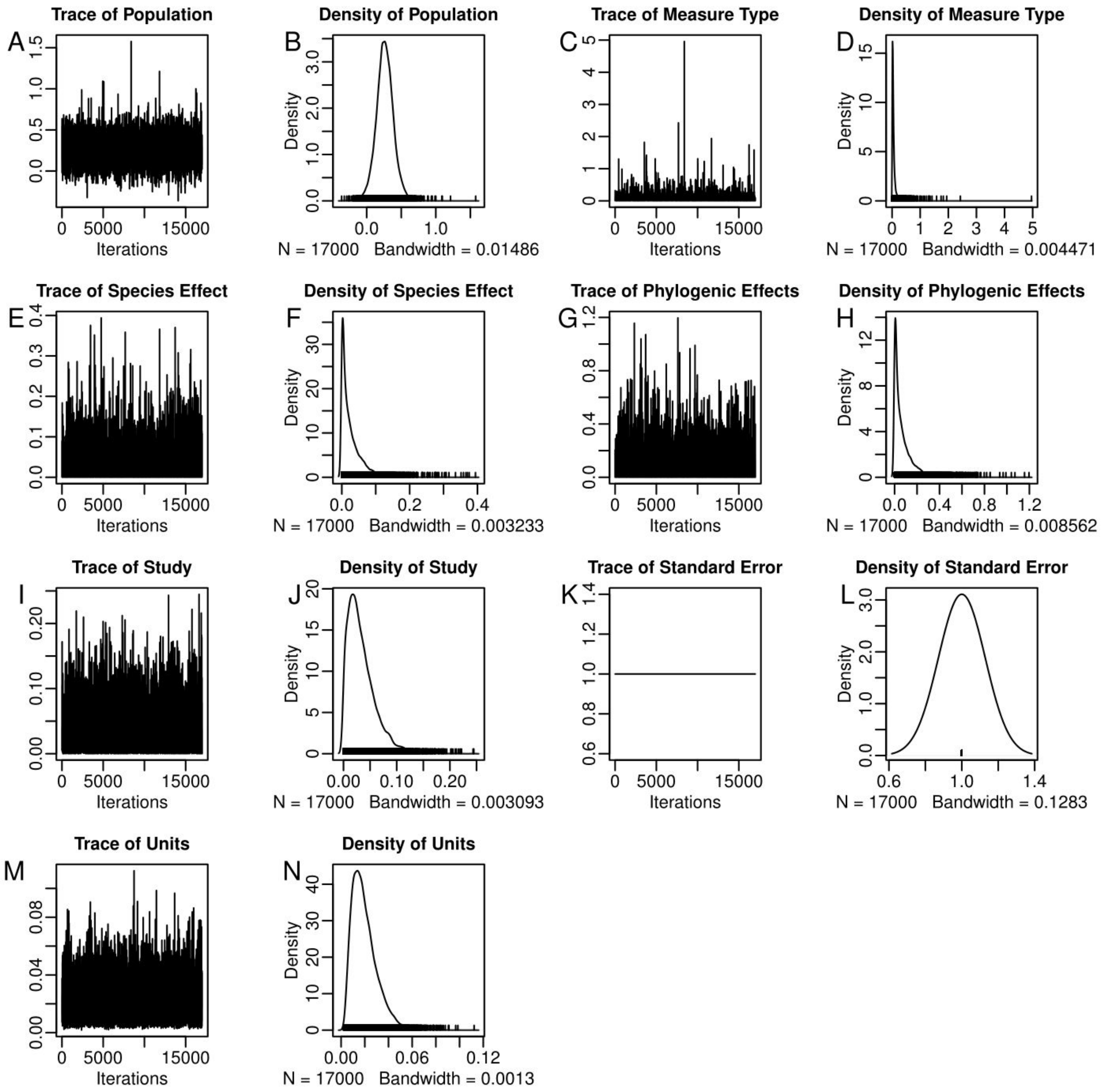

Supplemental Figure SB3: Convergence of parameters in the repertoire size model for the song stability data set. Annotation is the same as in Supplemental Figure S13, except that the first two pairs of graphs are fixed effects for smaller and larger syllable repertoires. There is no visible trend in the traces that would suggest autocorrelation affected the simulation, and the traces for all terms appear well mixed. The density maps for all random effects terms peak above zero.

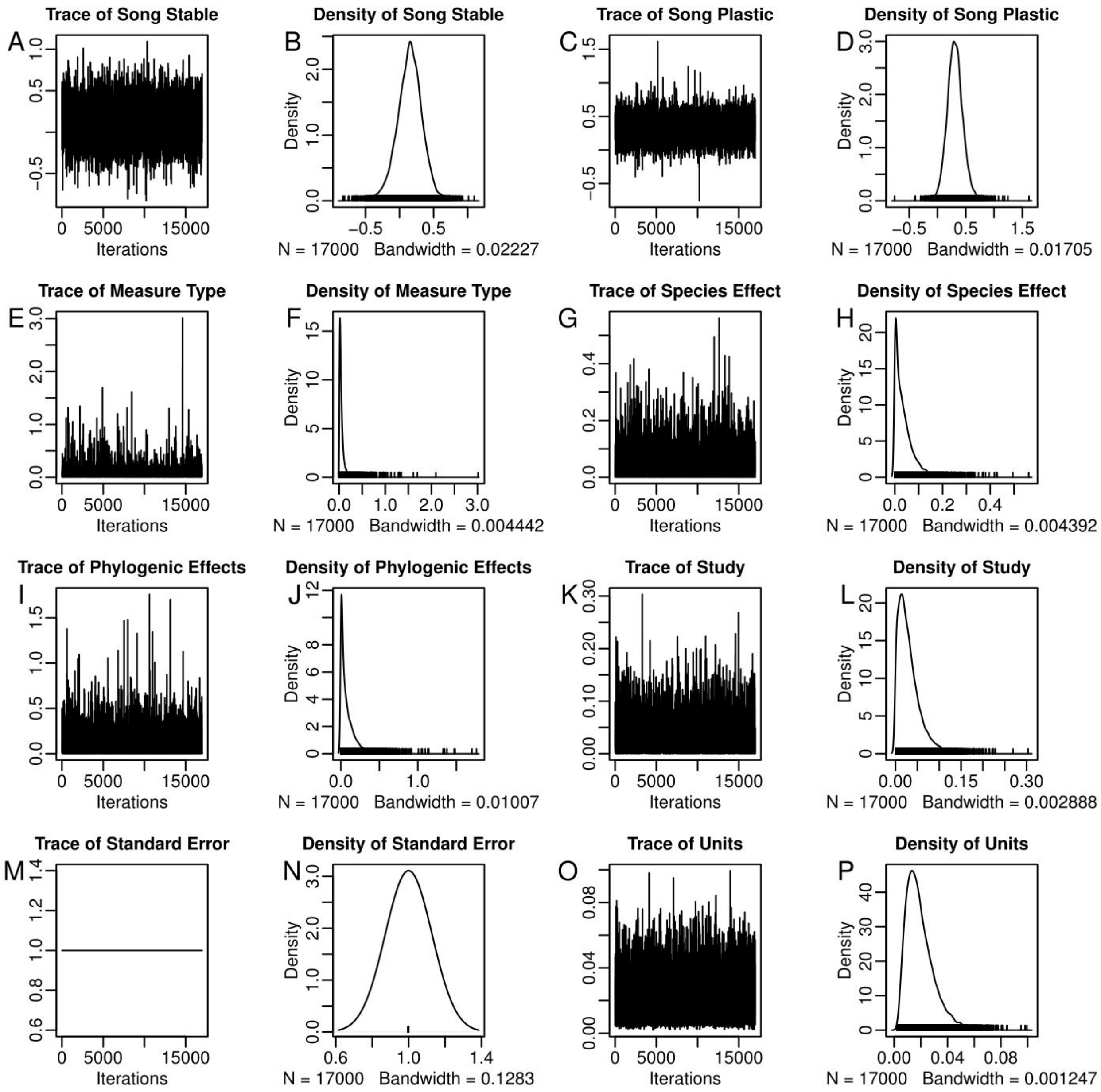

Supplemental Figure SB4: Convergence of parameters in the song stability model for the song stability data set. Annotation is the same as in Supplemental Figure S13, except that the first two pairs of graphs are fixed effects for song-stable and song-plastic species. There is no visible trend in the traces that would suggest autocorrelation affected the simulation, and the traces for all terms appear well mixed. The density maps for all random effects terms peak above zero.

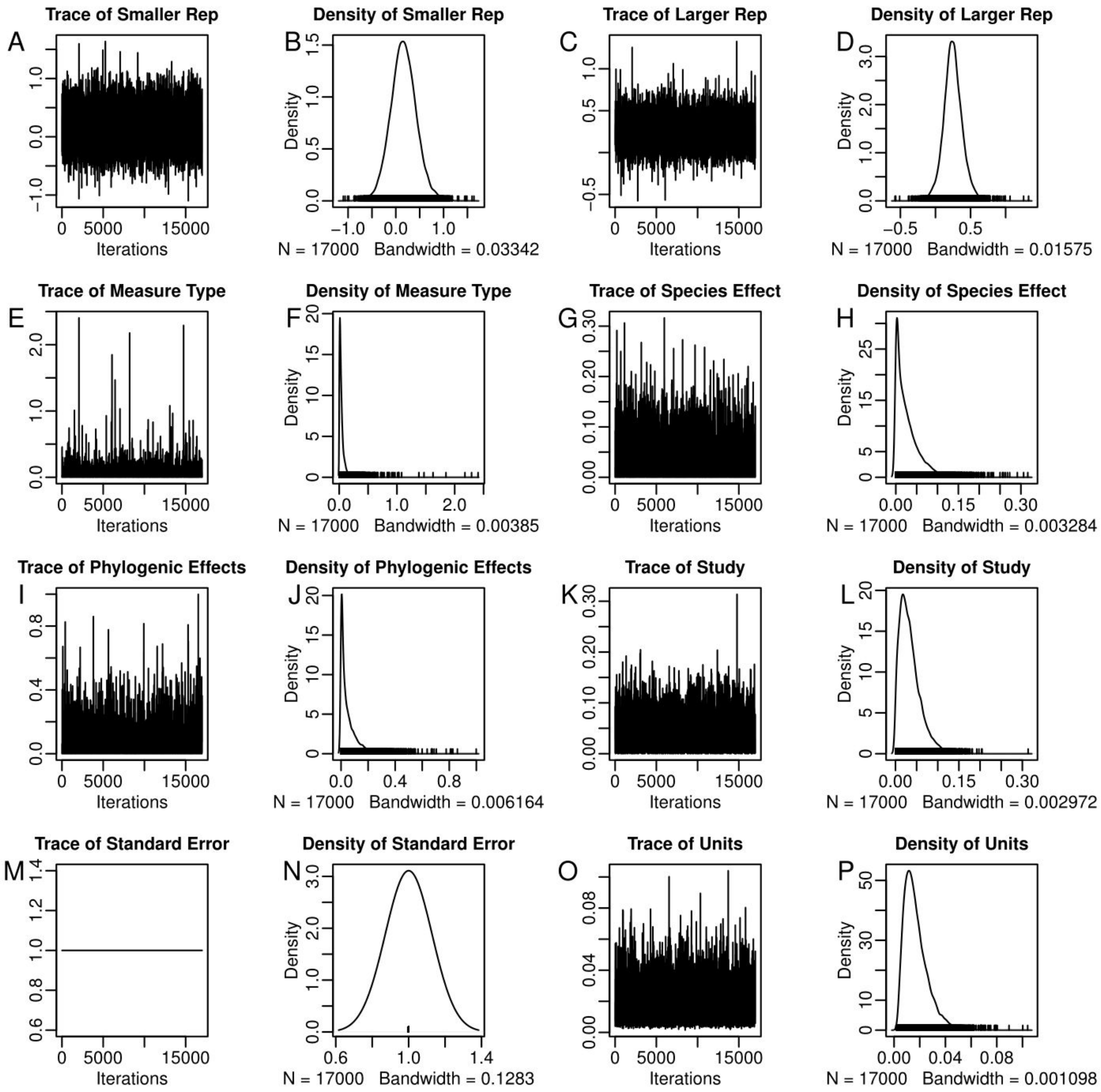

Supplemental Figure SB5: Convergence of parameters in the syllable repertoires model for the syllable repertoire data set, with the threshold for 'larger' repertoires set to >= 7.46. Annotation is the same as in Supplemental Figure S13, except that the first two pairs of graphs are fixed effects for relatively smaller and larger syllable repertoires. There is no visible trend in the traces that would suggest autocorrelation affected the simulation, and the traces for all terms appear well mixed. The density maps for all random effects terms peak above zero.

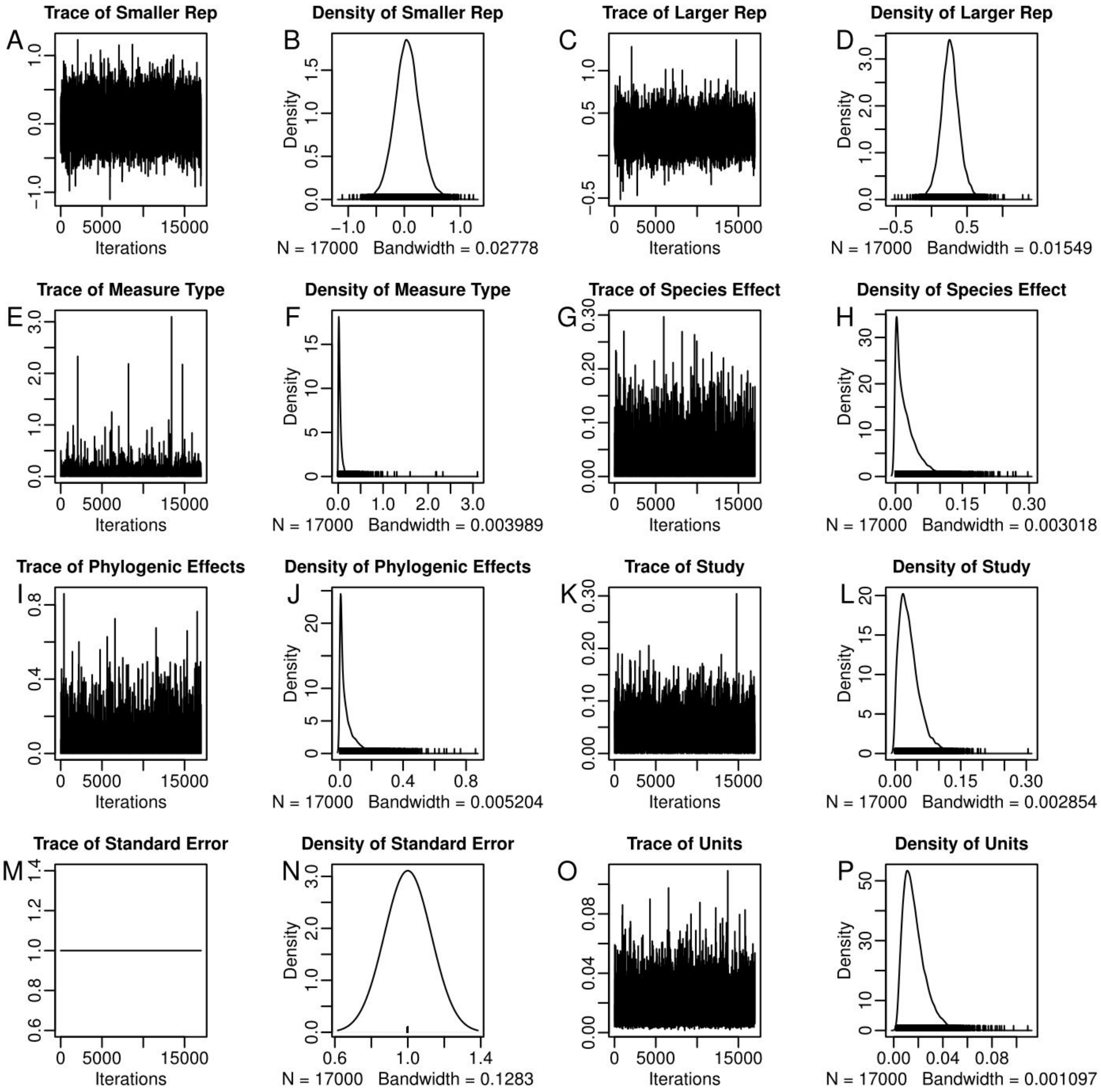

Supplemental Figure SB6: Convergence of parameters in the syllable repertoires model for the syllable repertoire data set, with the threshold for 'larger' repertoires set to >= 7.9. Annotation is the same as in Supplemental Figure S13, except that the first two pairs of graphs are fixed effects for relatively smaller and larger syllable repertoires. There is no visible trend in the traces that would suggest autocorrelation affected the simulation, and the traces for all terms appear well mixed. The density maps for all random effects terms peak above zero.

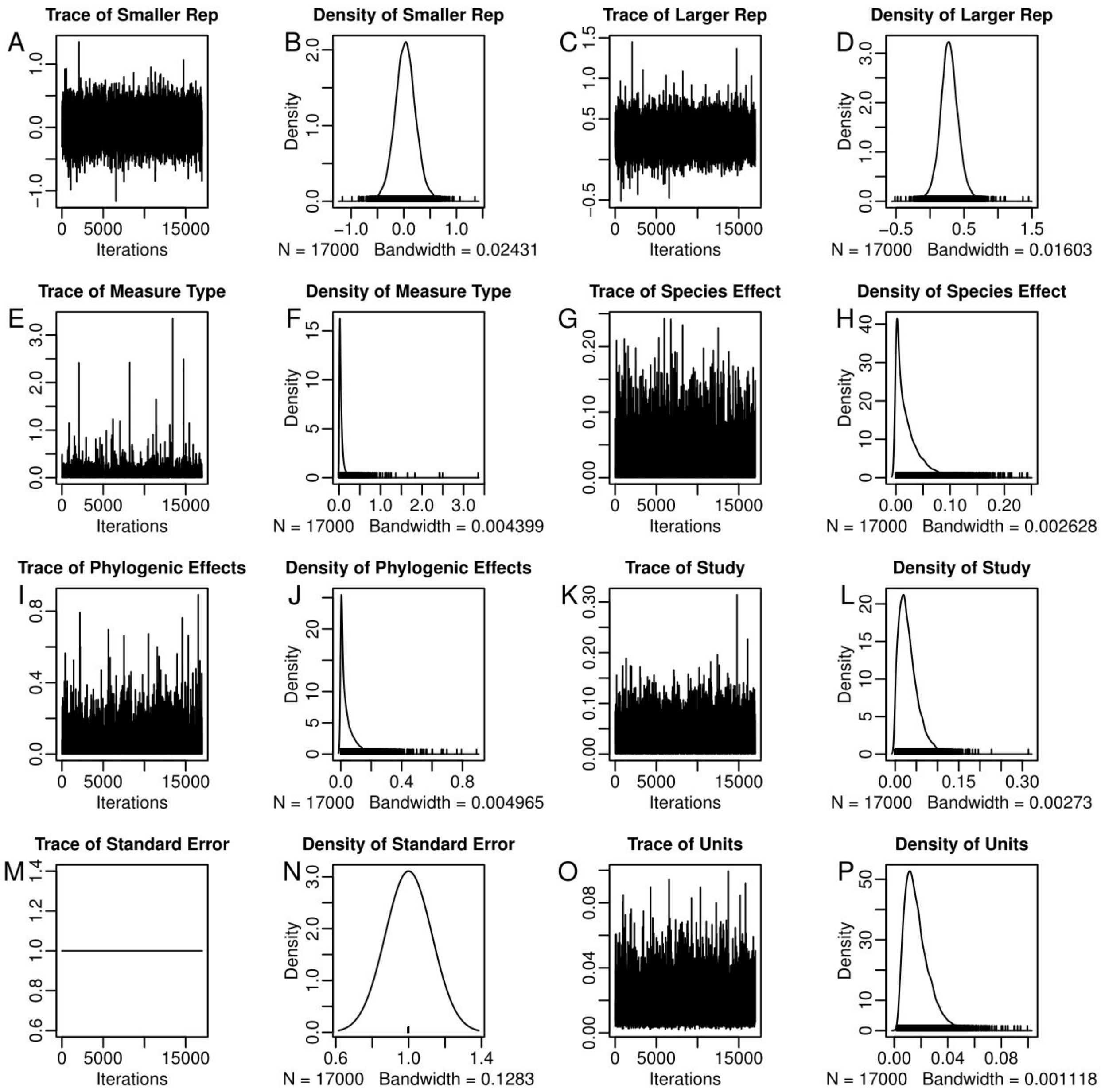

Supplemental Figure SB79: Convergence of parameters in the syllable repertoires model for the syllable repertoire data set, with the threshold for 'larger' repertoires set to >= 10.75. Annotation is the same as in Supplemental Figure S13, except that the first two pairs of graphs are fixed effects for relatively smaller and larger syllable repertoires. There is no visible trend in the traces that would suggest autocorrelation affected the simulation, and the traces for all terms appear well mixed. The density maps for all random effects terms peak above zero.

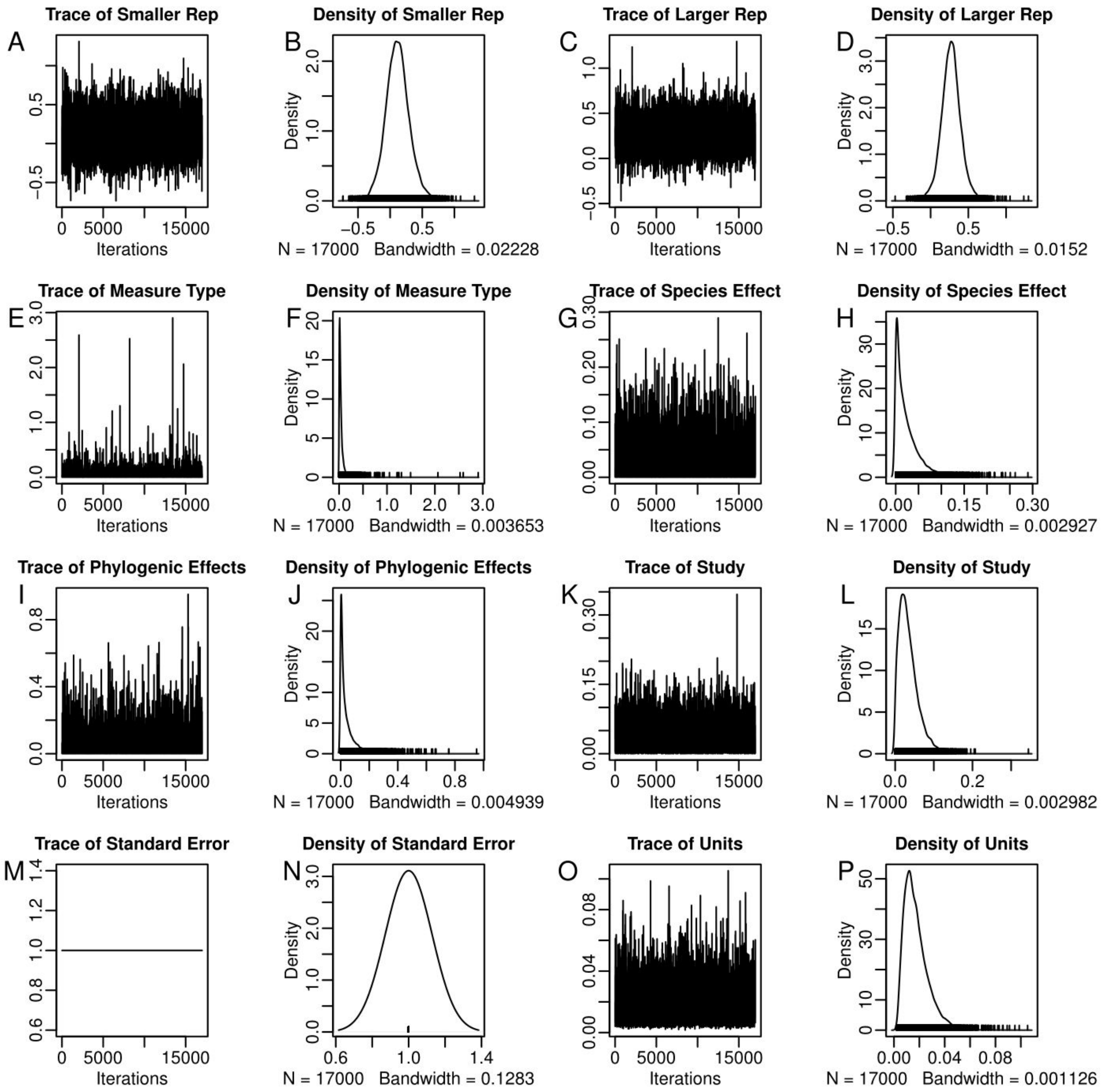

Supplemental Figure SB8: Convergence of parameters in the syllable repertoires model for the syllable repertoire data set, with the threshold for 'larger' repertoires set to >= 14. Annotation is the same as in Supplemental Figure S13, except that the first two pairs of graphs are fixed effects for relatively smaller and larger syllable repertoires. There is no visible trend in the traces that would suggest autocorrelation affected the simulation, and the traces for all terms appear well mixed. The density maps for all random effects terms peak above zero.

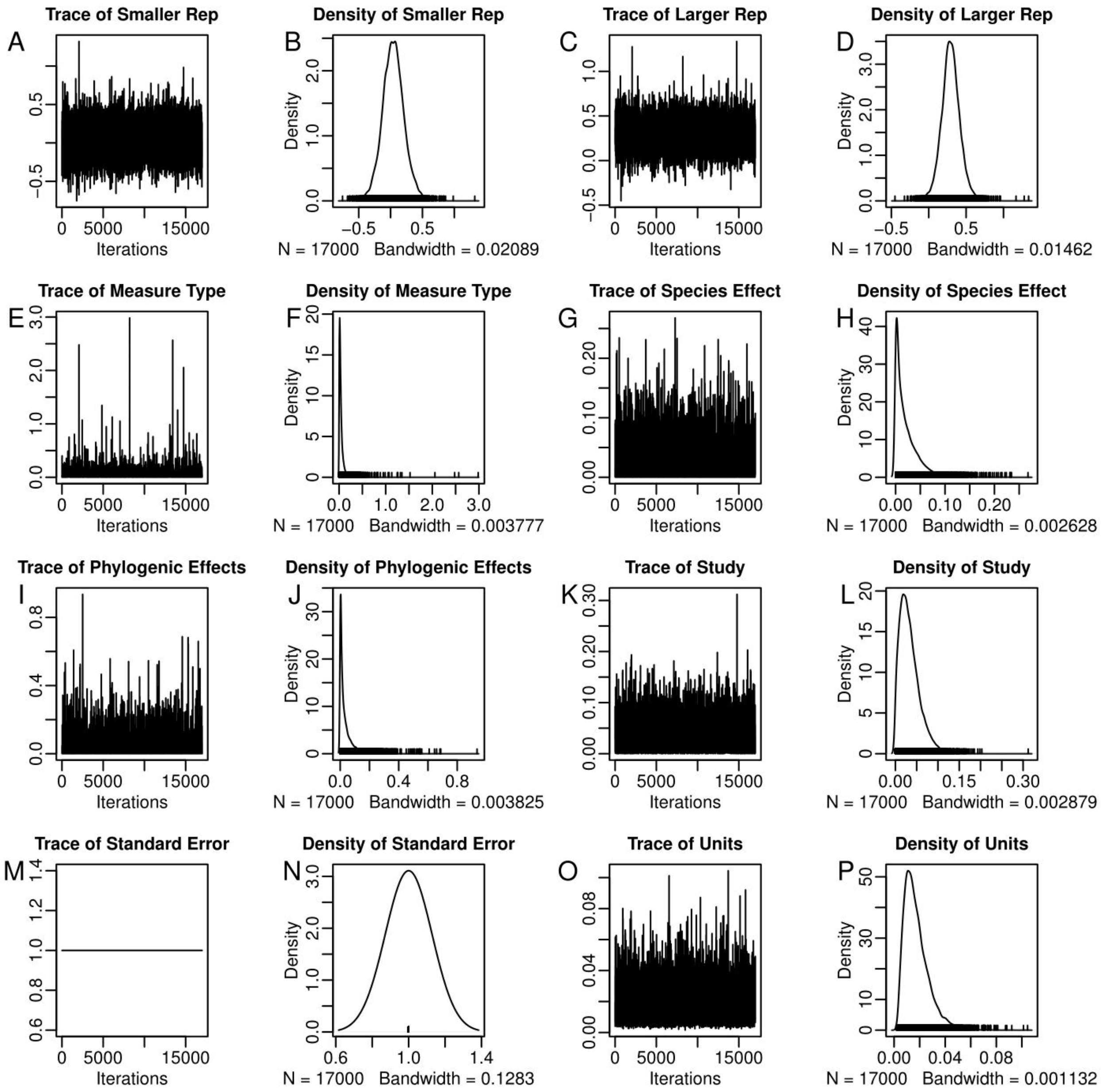

Supplemental Figure SB9: Convergence of parameters in the syllable repertoires model for the syllable repertoire data set, with the threshold for 'larger' repertoires set to >= 17.43. Annotation is the same as in Supplemental Figure S13, except that the first two pairs of graphs are fixed effects for relatively smaller and larger syllable repertoires. There is no visible trend in the traces that would suggest autocorrelation affected the simulation, and the traces for all terms appear well mixed. The density maps for all random effects terms peak above zero.

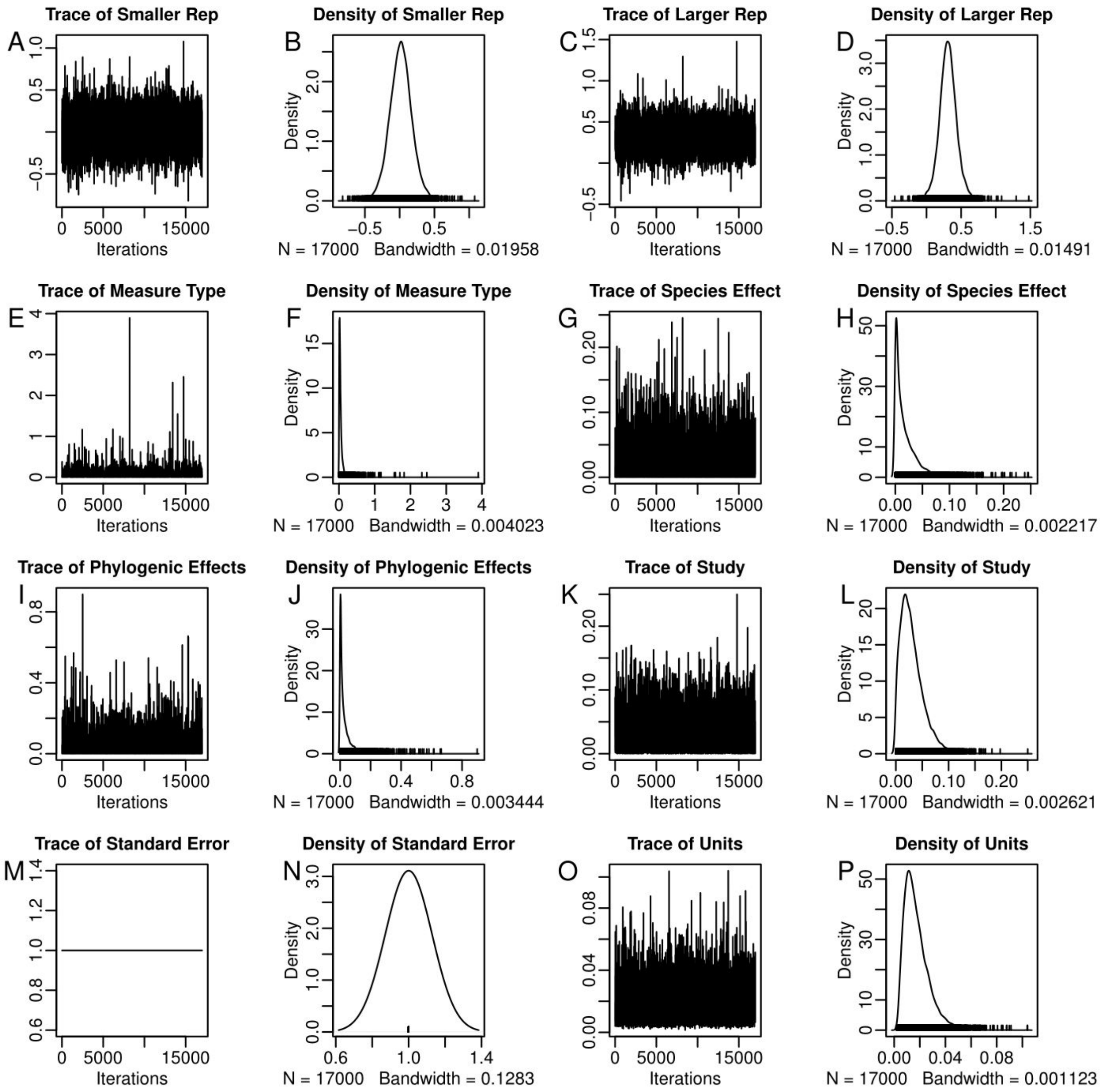

Supplemental Figure SB10: Convergence of parameters in the syllable repertoires model for the syllable repertoire data set, with the threshold for 'larger' repertoires set to >= 18.15. Annotation is the same as in Supplemental Figure S13, except that the first two pairs of graphs are fixed effects for relatively smaller and larger syllable repertoires. There is no visible trend in the traces that would suggest autocorrelation affected the simulation, and the traces for all terms appear well mixed. The density maps for all random effects terms peak above zero.

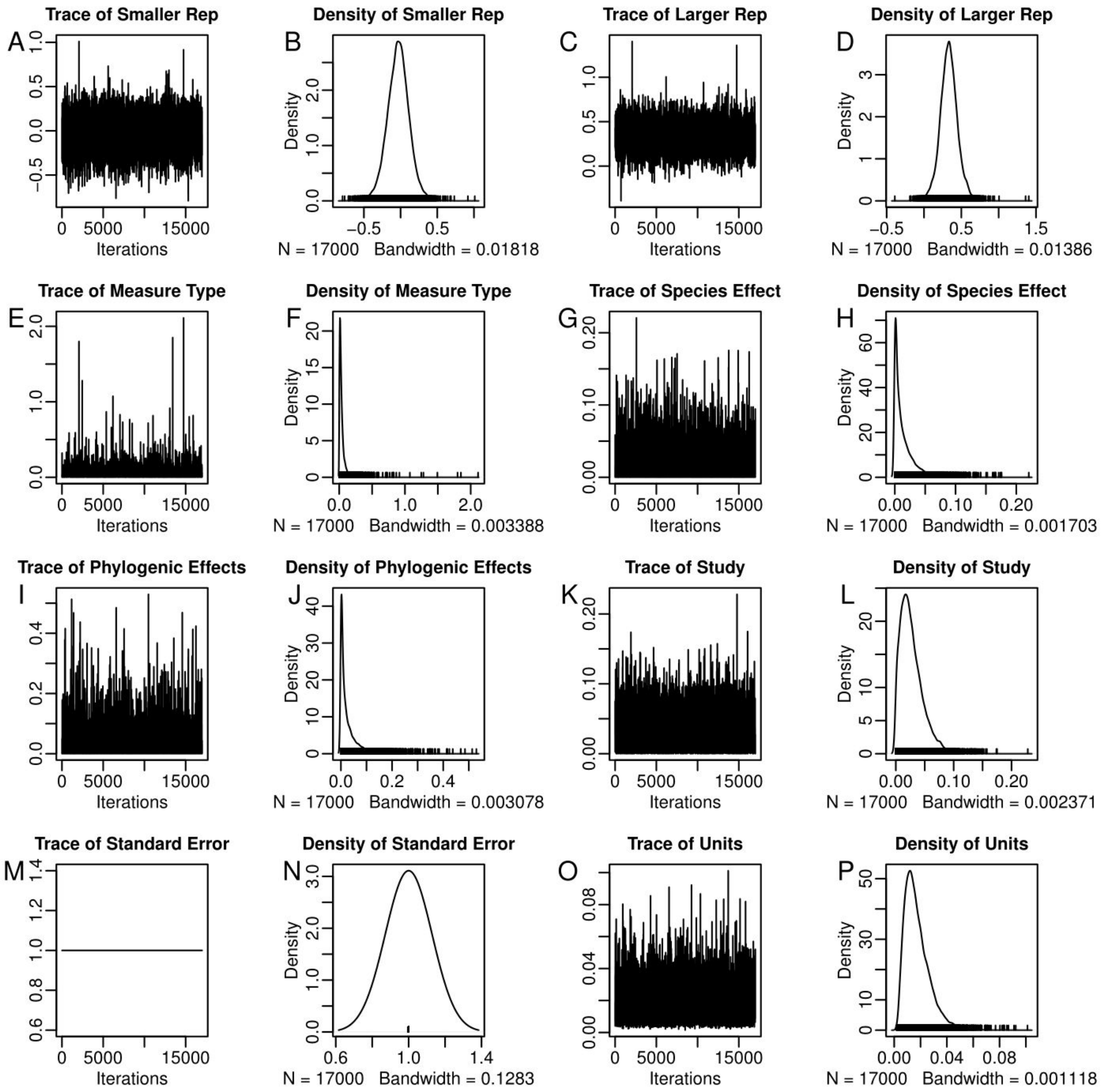

Supplemental Figure SB11: Convergence of parameters in the syllable repertoires model for the syllable repertoire data set, with the threshold for 'larger' repertoires set to >= 18.5. Annotation is the same as in Supplemental Figure S13, except that the first two pairs of graphs are fixed effects for relatively smaller and larger syllable repertoires. There is no visible trend in the traces that would suggest autocorrelation affected the simulation, and the traces for all terms appear well mixed. The density maps for all random effects terms peak above zero.

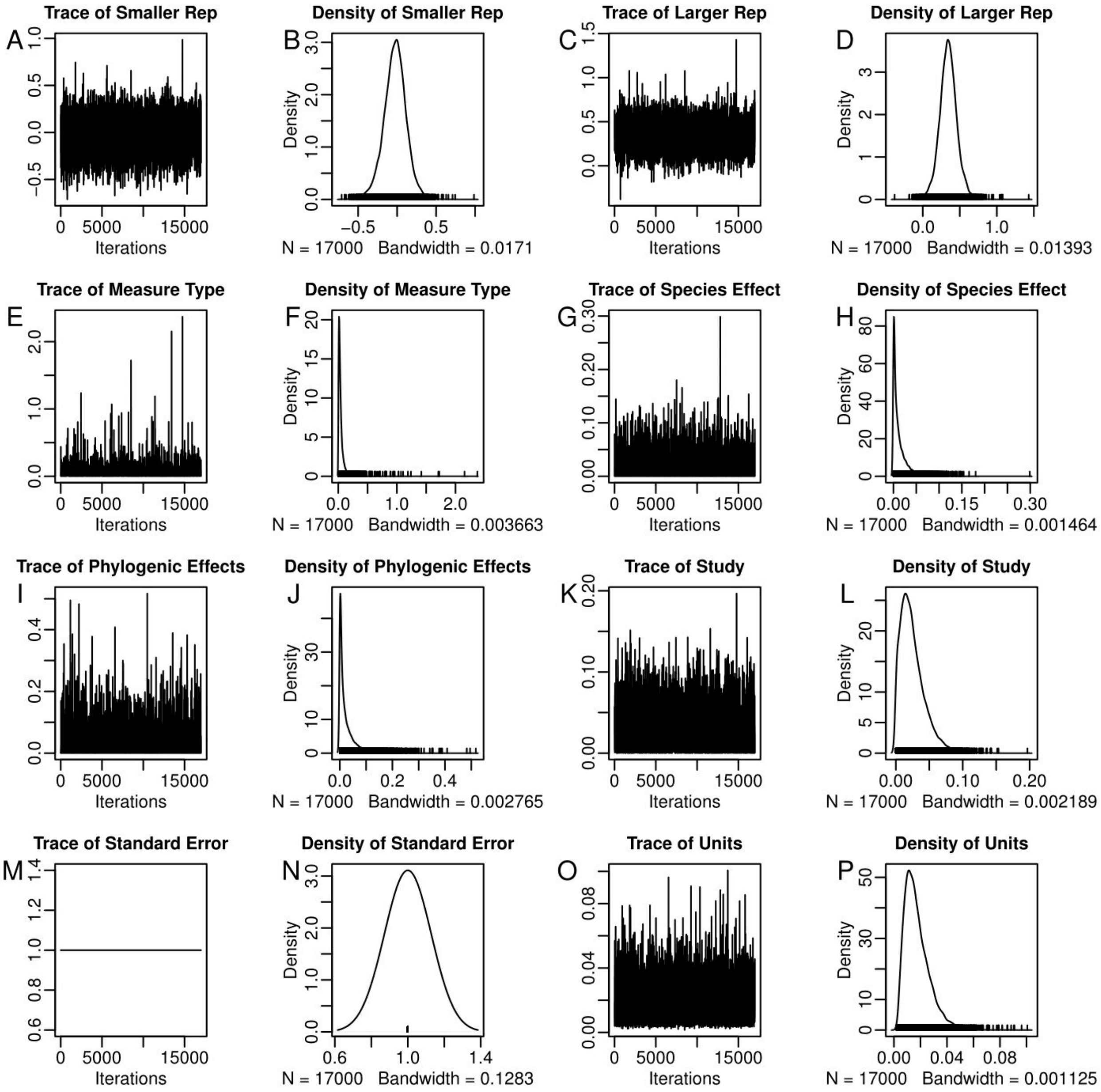

Supplemental Figure SB12: Convergence of parameters in the syllable repertoires model for the syllable repertoire data set, with the threshold for 'larger' repertoires set to >= 20.1. Annotation is the same as in Supplemental Figure S13, except that the first two pairs of graphs are fixed effects for relatively smaller and larger syllable repertoires. There is no visible trend in the traces that would suggest autocorrelation affected the simulation, and the traces for all terms appear well mixed. The density maps for all random effects terms peak above zero.

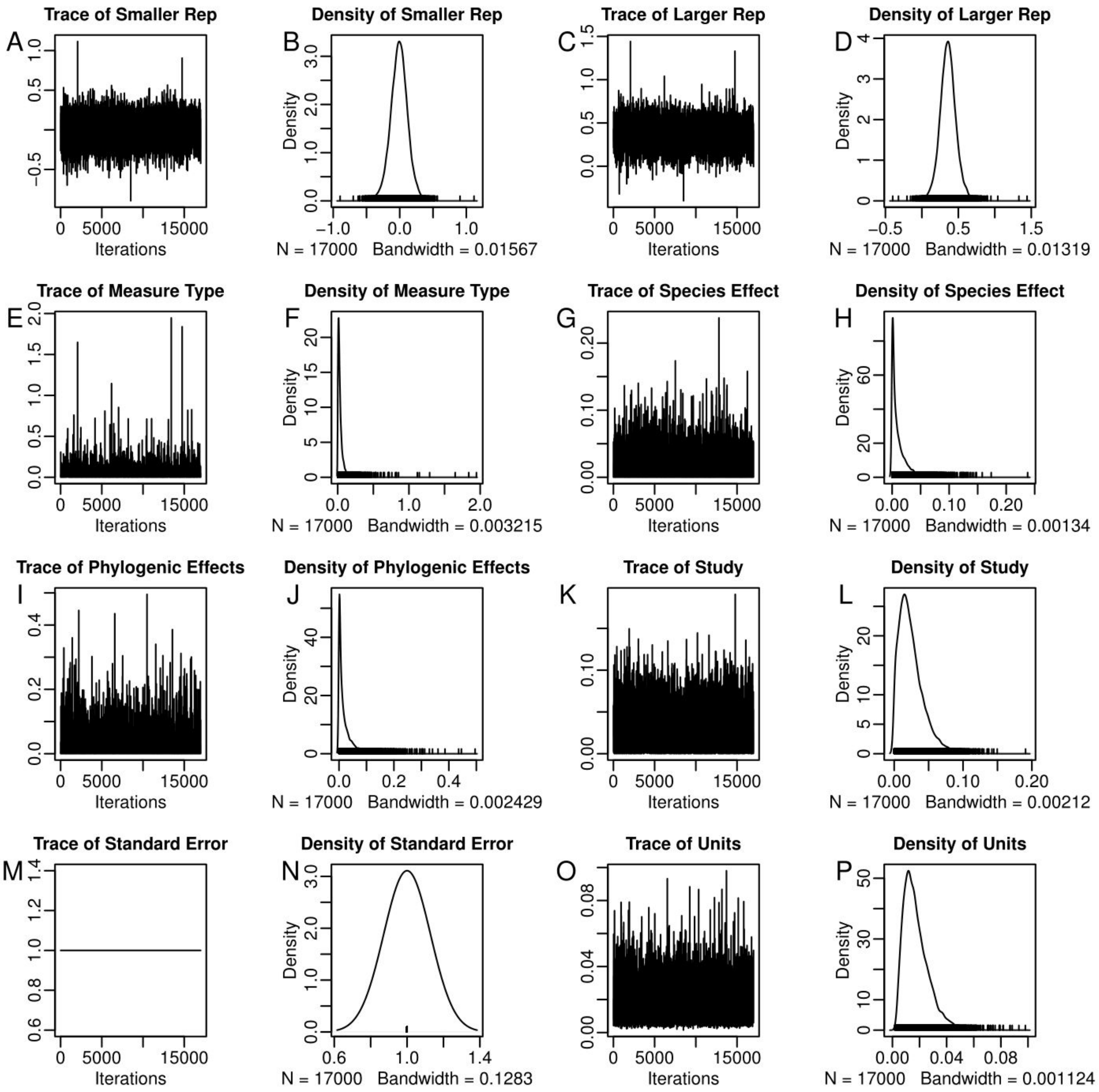

Supplemental Figure SB13: Convergence of parameters in the syllable repertoires model for the syllable repertoire data set, with the threshold for 'larger' repertoires set to >= 22.5. Annotation is the same as in Supplemental Figure S13, except that the first two pairs of graphs are fixed effects for relatively smaller and larger syllable repertoires. There is no visible trend in the traces that would suggest autocorrelation affected the simulation, and the traces for all terms appear well mixed. The density maps for all random effects terms peak above zero.

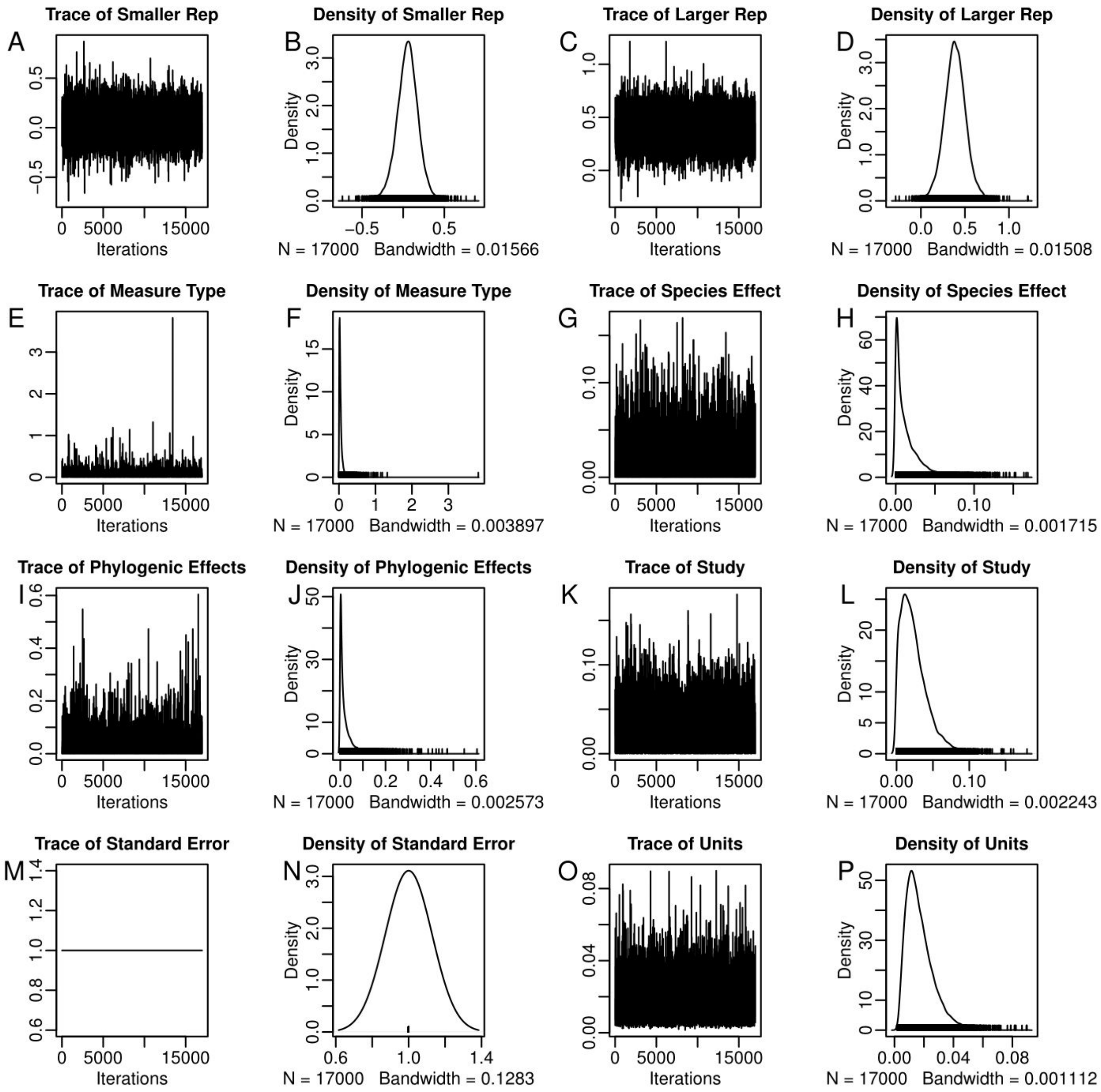

Supplemental Figure SB14: Convergence of parameters in the syllable repertoires model for the syllable repertoire data set, with the threshold for 'larger' repertoires set to >= 25.3. Annotation is the same as in Supplemental Figure S13, except that the first two pairs of graphs are fixed effects for relatively smaller and larger syllable repertoires. There is no visible trend in the traces that would suggest autocorrelation affected the simulation, and the traces for all terms appear well mixed. The density maps for all random effects terms peak above zero.

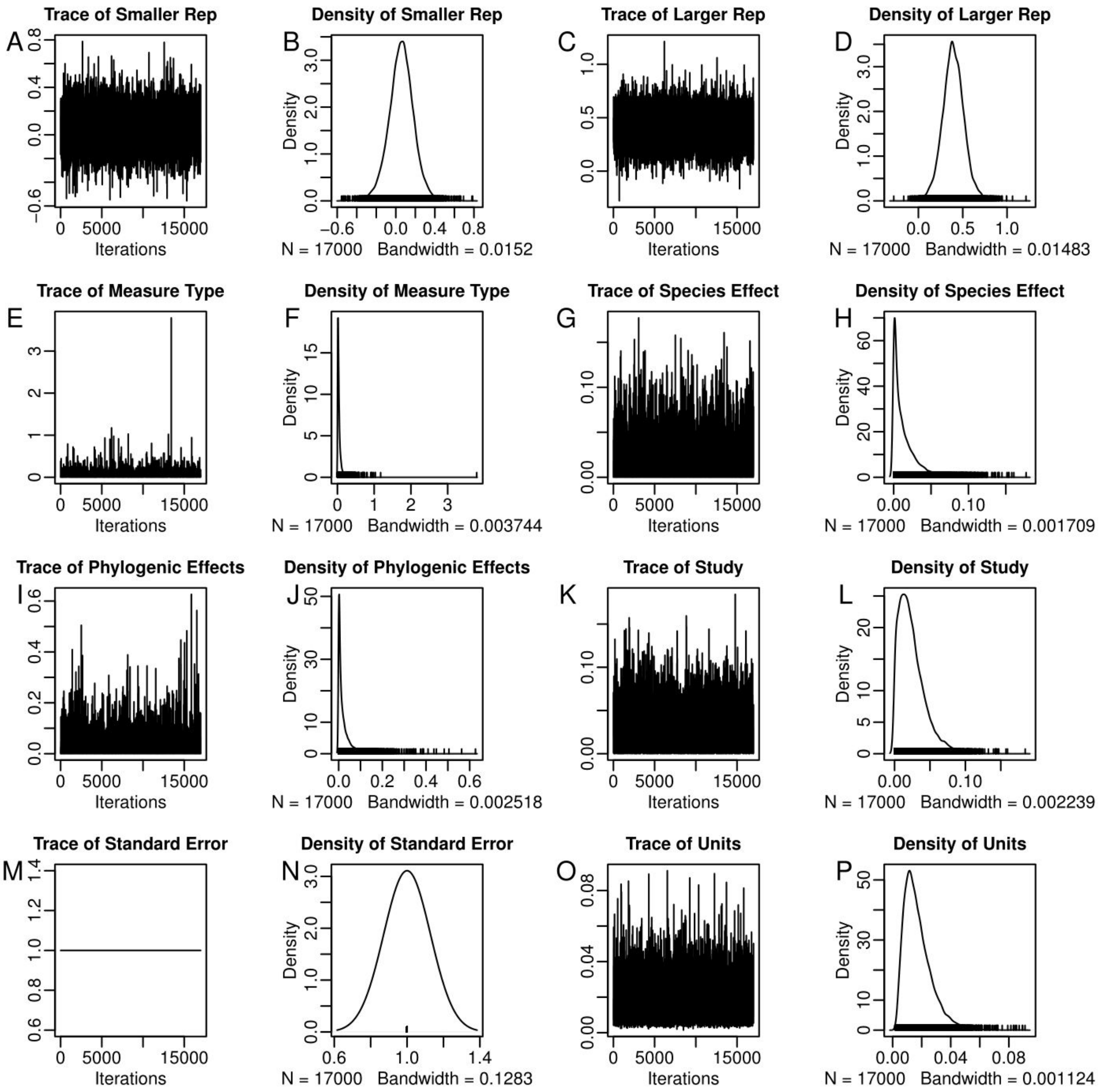

Supplemental Figure SB15: Convergence of parameters in the syllable repertoires model for the syllable repertoire data set, with the threshold for 'larger' repertoires set to >= 28.4. Annotation is the same as in Supplemental Figure S13, except that the first two pairs of graphs are fixed effects for relatively smaller and larger syllable repertoires. There is no visible trend in the traces that would suggest autocorrelation affected the simulation, and the traces for all terms appear well mixed. The density maps for all random effects terms peak above zero.

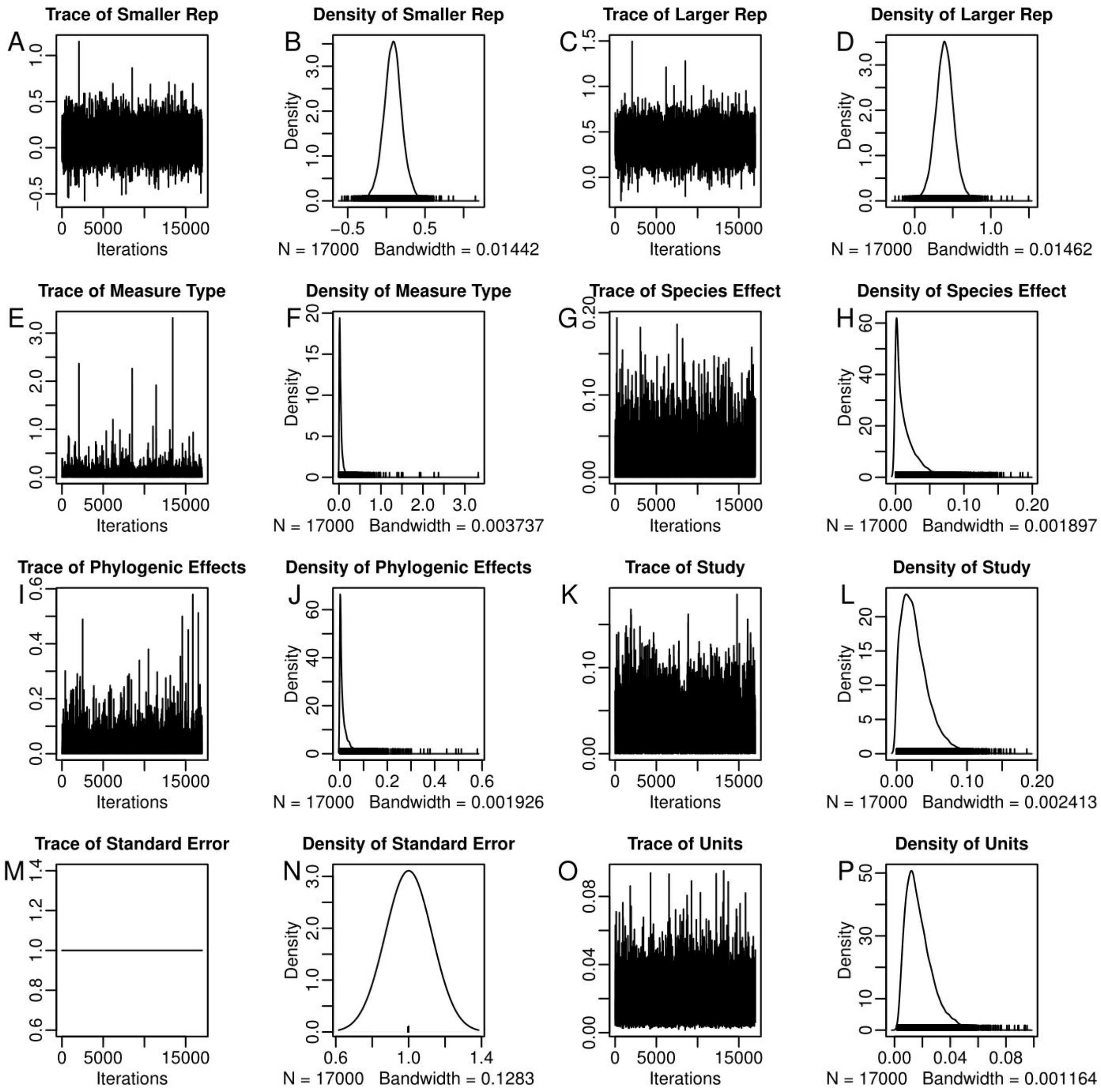

Supplemental Figure SB16: Convergence of parameters in the syllable repertoires model for the syllable repertoire data set, with the threshold for 'larger' repertoires set to >= 31.4. Annotation is the same as in Supplemental Figure S13, except that the first two pairs of graphs are fixed effects for relatively smaller and larger syllable repertoires. There is no visible trend in the traces that would suggest autocorrelation affected the simulation, and the traces for all terms appear well mixed. The density maps for all random effects terms peak above zero.

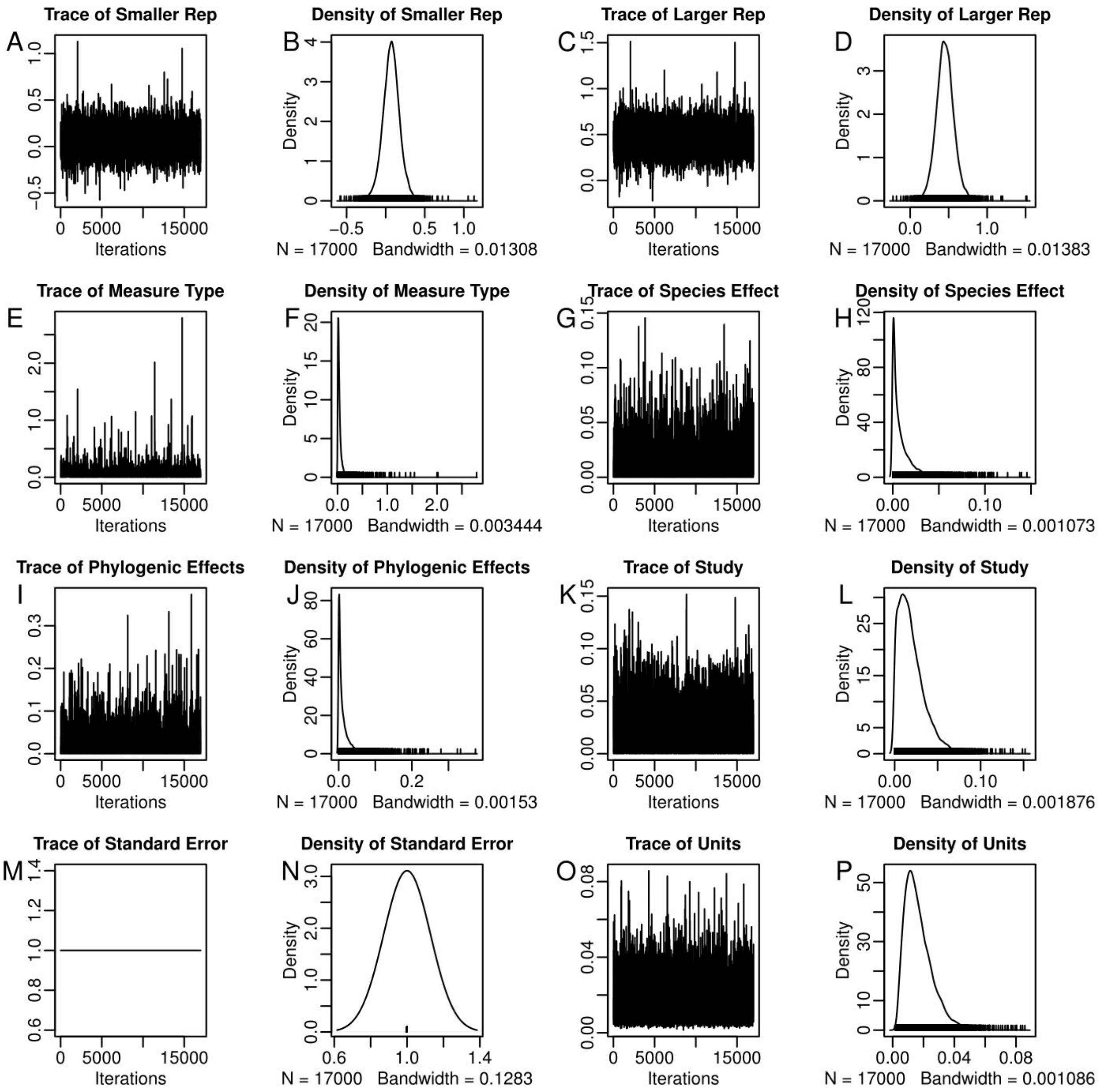

Supplemental Figure SB17: Convergence of parameters in the syllable repertoires model for the syllable repertoire data set, with the threshold for 'larger' repertoires set to >= 35.1. Annotation is the same as in Supplemental Figure S13, except that the first two pairs of graphs are fixed effects for relatively smaller and larger syllable repertoires. There is no visible trend in the traces that would suggest autocorrelation affected the simulation, and the traces for all terms appear well mixed. The density maps for all random effects terms peak above zero.

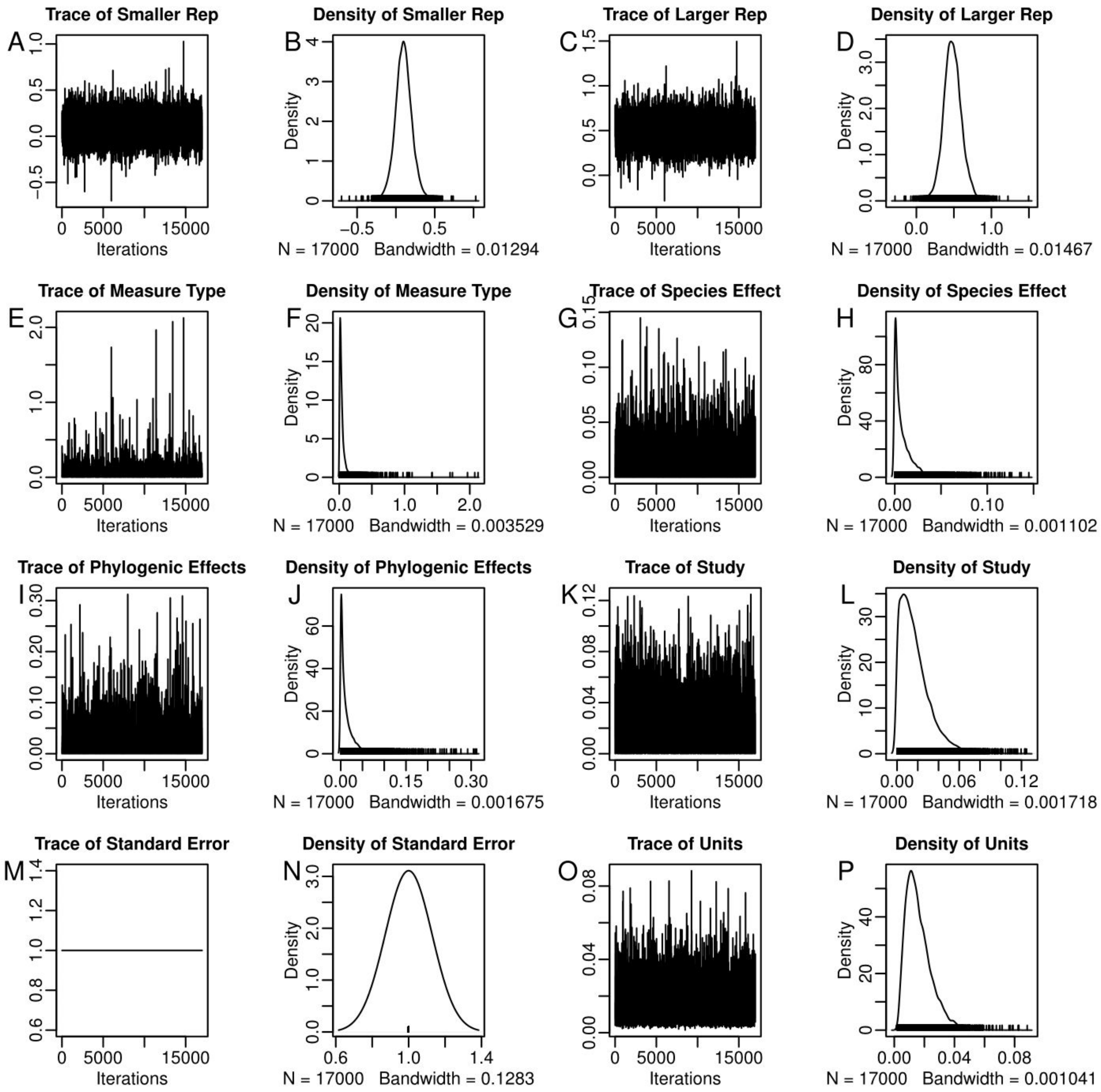

Supplemental Figure SB18: Convergence of parameters in the syllable repertoires model for the syllable repertoire data set, with the threshold for 'larger' repertoires set to >= 38. Annotation is the same as in Supplemental Figure S13, except that the first two pairs of graphs are fixed effects for relatively smaller and larger syllable repertoires. There is no visible trend in the traces that would suggest autocorrelation affected the simulation, and the traces for all terms appear well mixed. The density maps for all random effects terms peak above zero.

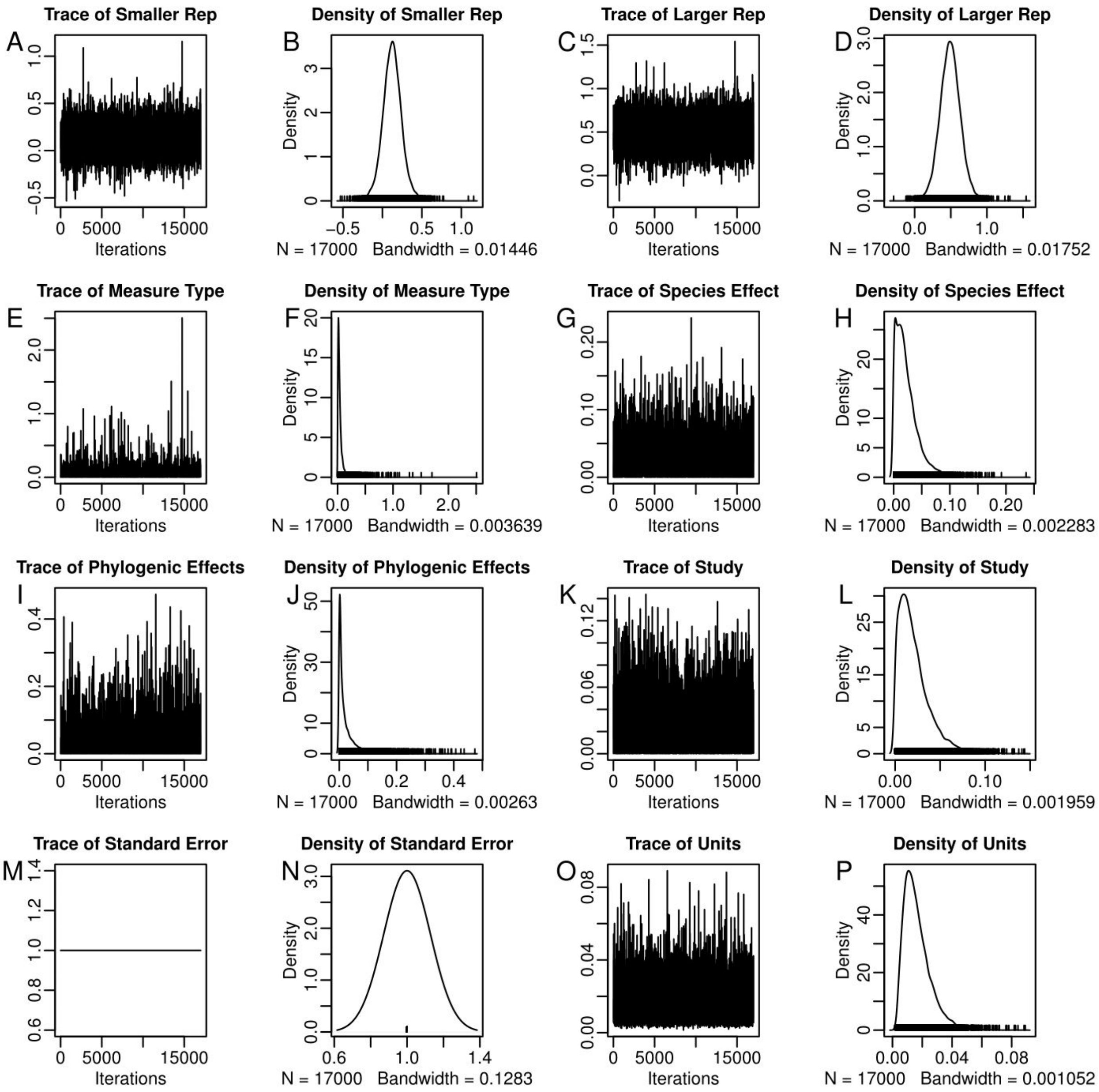

Supplemental Figure SB19: Convergence of parameters in the syllable repertoires model for the syllable repertoire data set, with the threshold for 'larger' repertoires set to >= 41.3. Annotation is the same as in Supplemental Figure S13, except that the first two pairs of graphs are fixed effects for relatively smaller and larger syllable repertoires. There is no visible trend in the traces that would suggest autocorrelation affected the simulation, and the traces for all terms appear well mixed. The density maps for all random effects terms peak above zero.

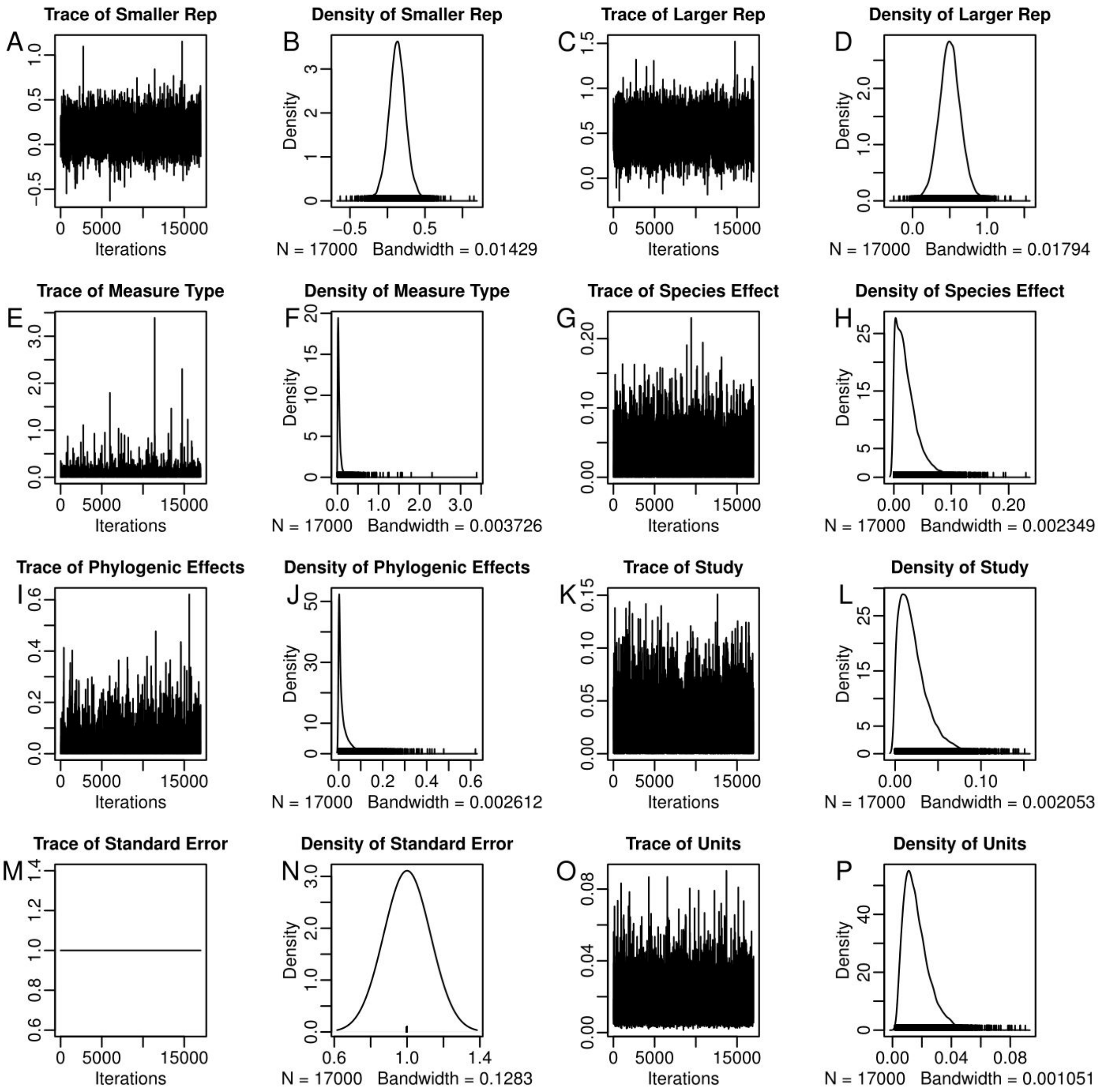

Supplemental Figure SB20: Convergence of parameters in the syllable repertoires model for the syllable repertoire data set, with the threshold for 'larger' repertoires set to >= 41.95. Annotation is the same as in Supplemental Figure S13, except that the first two pairs of graphs are fixed effects for relatively smaller and larger syllable repertoires. There is no visible trend in the traces that would suggest autocorrelation affected the simulation, and the traces for all terms appear well mixed. The density maps for all random effects terms peak above zero.

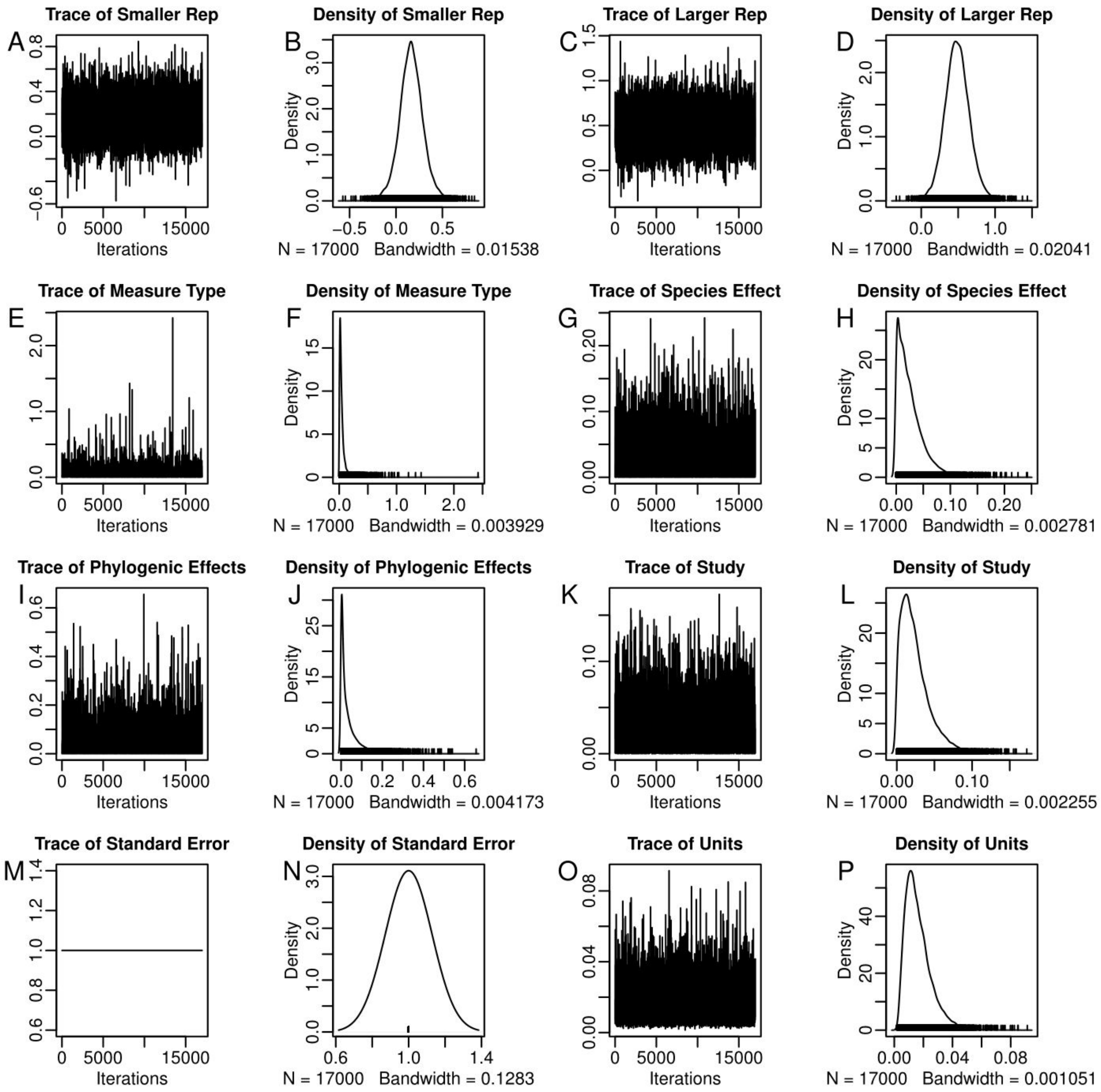

Supplemental Figure SB21: Convergence of parameters in the syllable repertoires model for the syllable repertoire data set, with the threshold for 'larger' repertoires set to >= 55. Annotation is the same as in Supplemental Figure S13, except that the first two pairs of graphs are fixed effects for relatively smaller and larger syllable repertoires. There is no visible trend in the traces that would suggest autocorrelation affected the simulation, and the traces for all terms appear well mixed. The density maps for all random effects terms peak above zero.

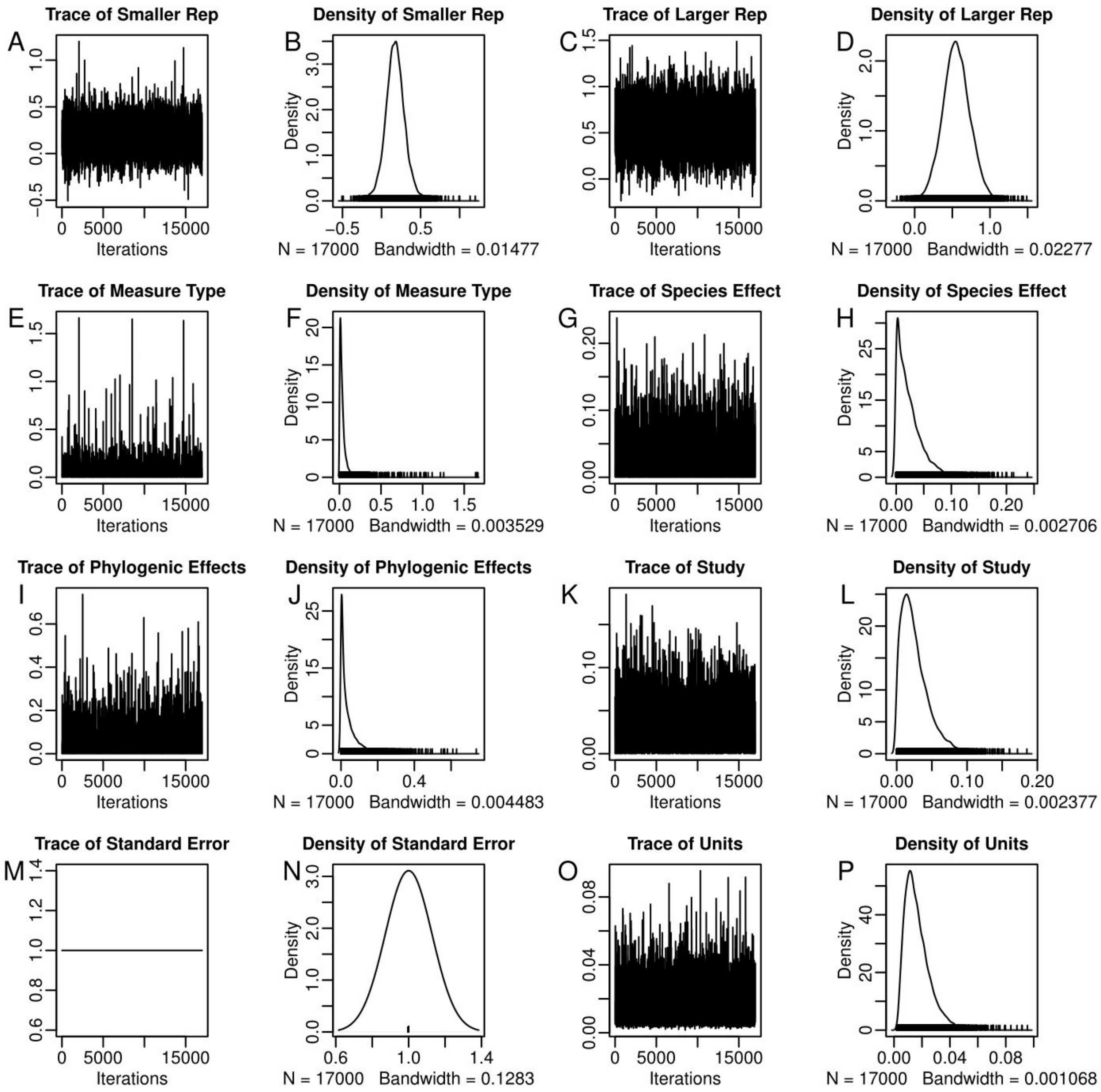

Supplemental Figure SB22: Convergence of parameters in the syllable repertoires model for the syllable repertoire data set, with the threshold for 'larger' repertoires set to >= 86. Annotation is the same as in Supplemental Figure S13, except that the first two pairs of graphs are fixed effects for relatively smaller and larger syllable repertoires. There is no visible trend in the traces that would suggest autocorrelation affected the simulation, and the traces for all terms appear well mixed. The density maps for all random effects terms peak above zero.

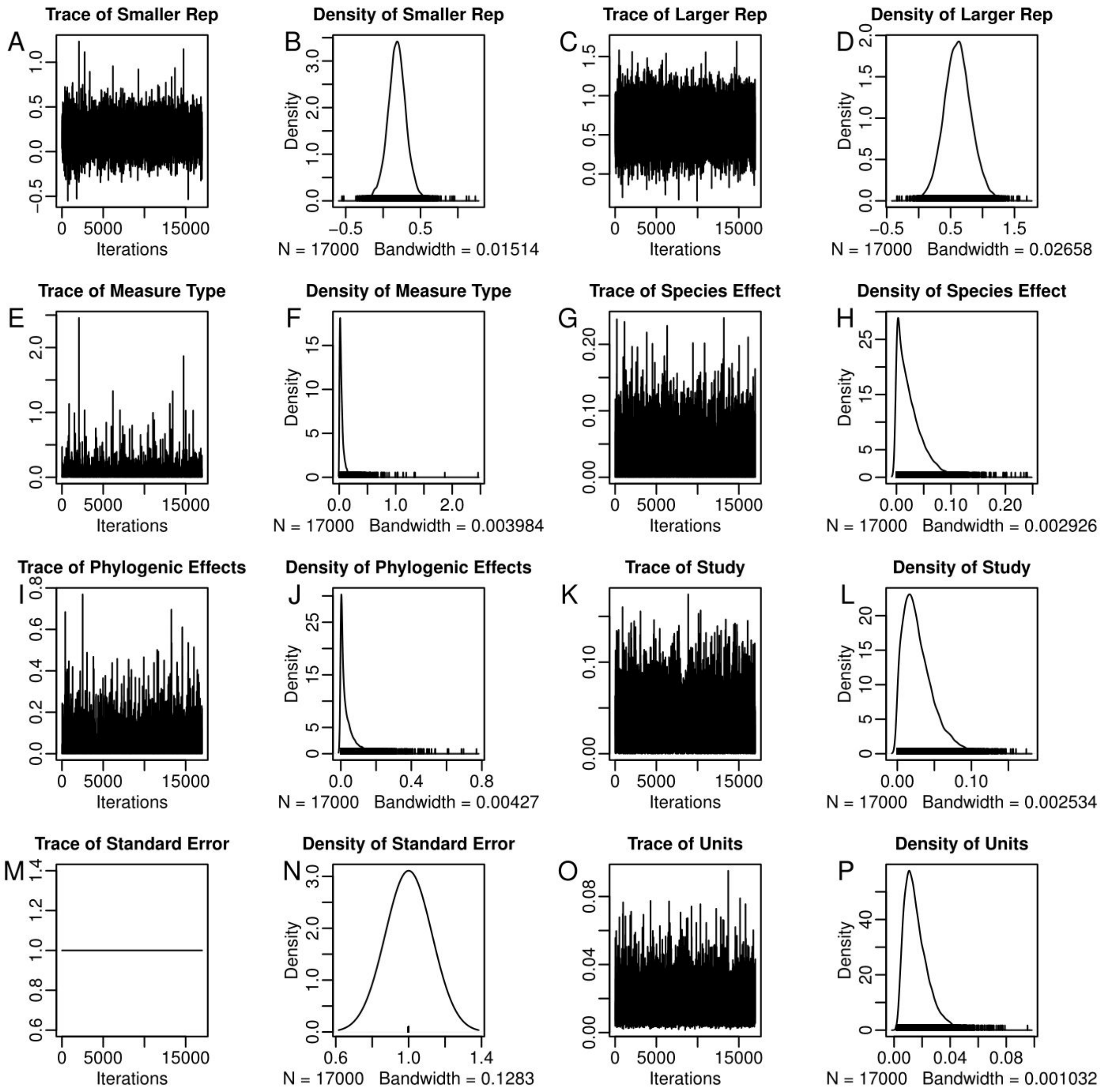

Supplemental Figure SB23: Convergence of parameters in the syllable repertoires model for the syllable repertoire data set, with the threshold for 'larger' repertoires set to >= 216. Annotation is the same as in Supplemental Figure S13, except that the first two pairs of graphs are fixed effects for relatively smaller and larger syllable repertoires. There is no visible trend in the traces that would suggest autocorrelation affected the simulation, and the traces for all terms appear well mixed. The density maps for all random effects terms peak above zero.

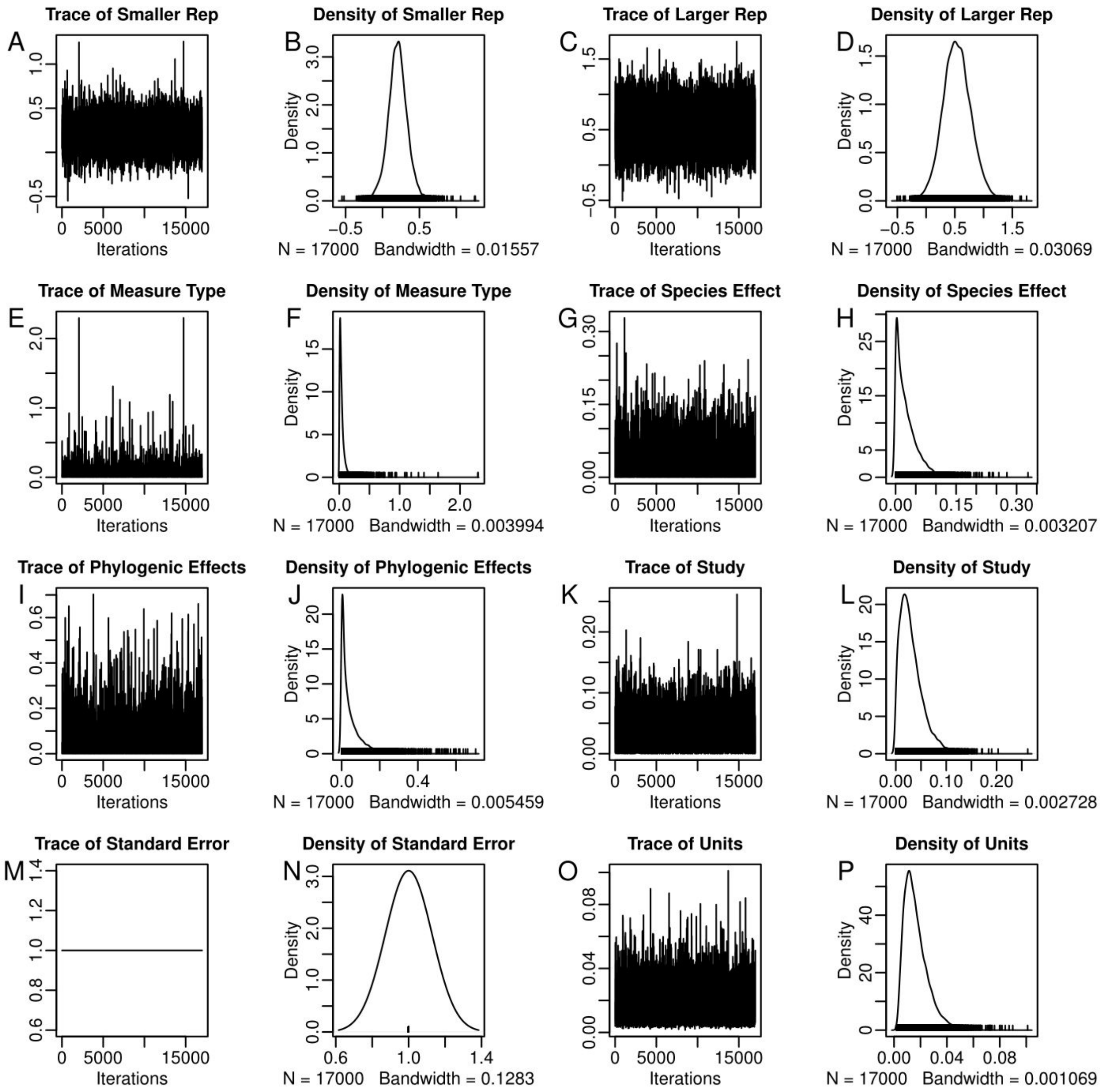

Supplemental Figure SB24: Convergence of parameters in the syllable repertoires model for the syllable repertoire data set, with the threshold for 'larger' repertoires set to >= 241. Annotation is the same as in Supplemental Figure S13, except that the first two pairs of graphs are fixed effects for relatively smaller and larger syllable repertoires. There is no visible trend in the traces that would suggest autocorrelation affected the simulation, and the traces for all terms appear well mixed. The density maps for all random effects terms peak above zero.

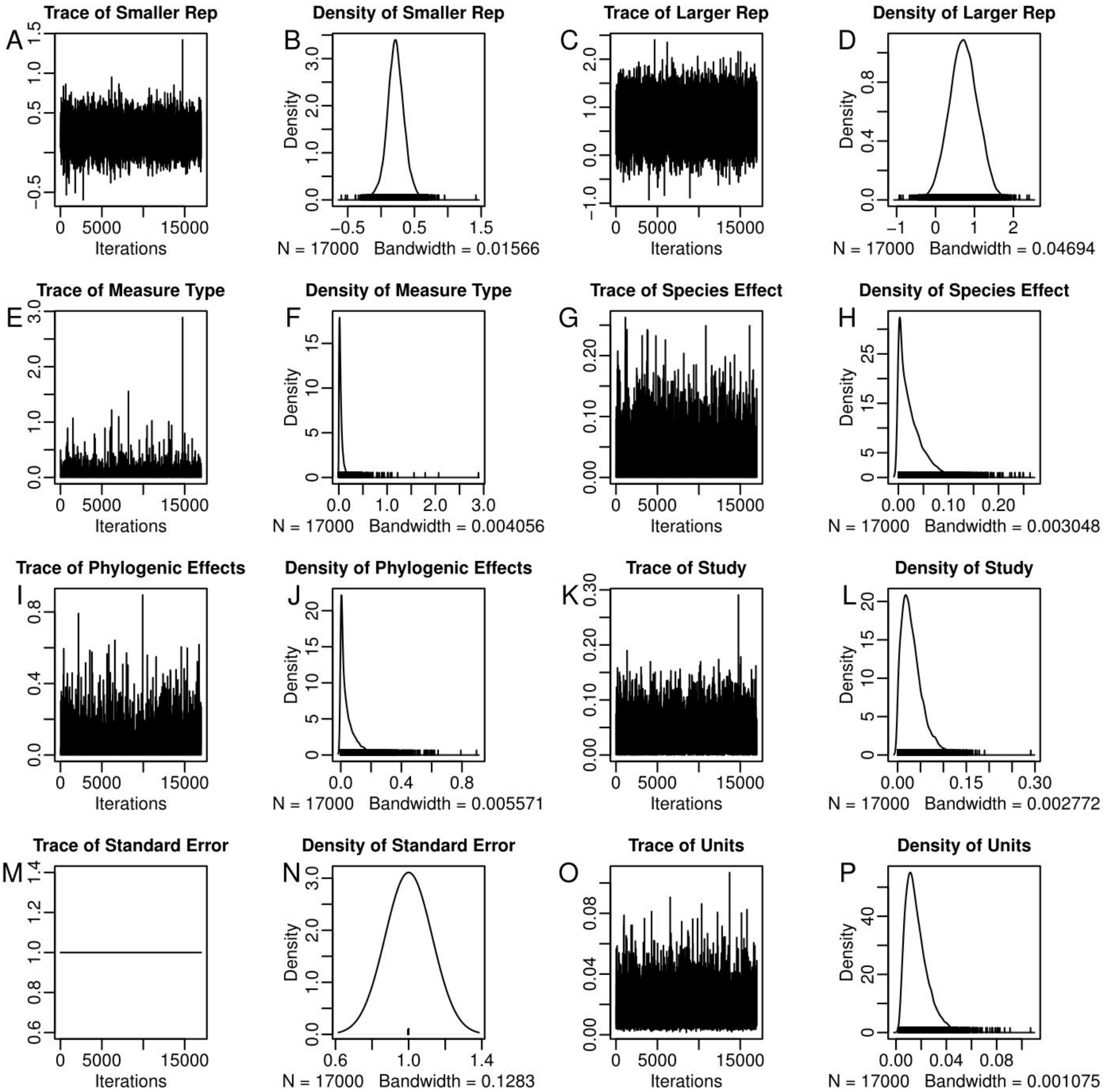

Supplemental Figure SB25: Convergence of parameters in the syllable repertoires model for the syllable repertoire data set, with the threshold for 'larger' repertoires set to >= 367.5. Annotation is the same as in Supplemental Figure S13, except that the first two pairs of graphs are fixed effects for relatively smaller and larger syllable repertoires. There is no visible trend in the traces that would suggest autocorrelation affected the simulation, and the traces for all terms appear well mixed. The density maps for all random effects terms peak above zero.

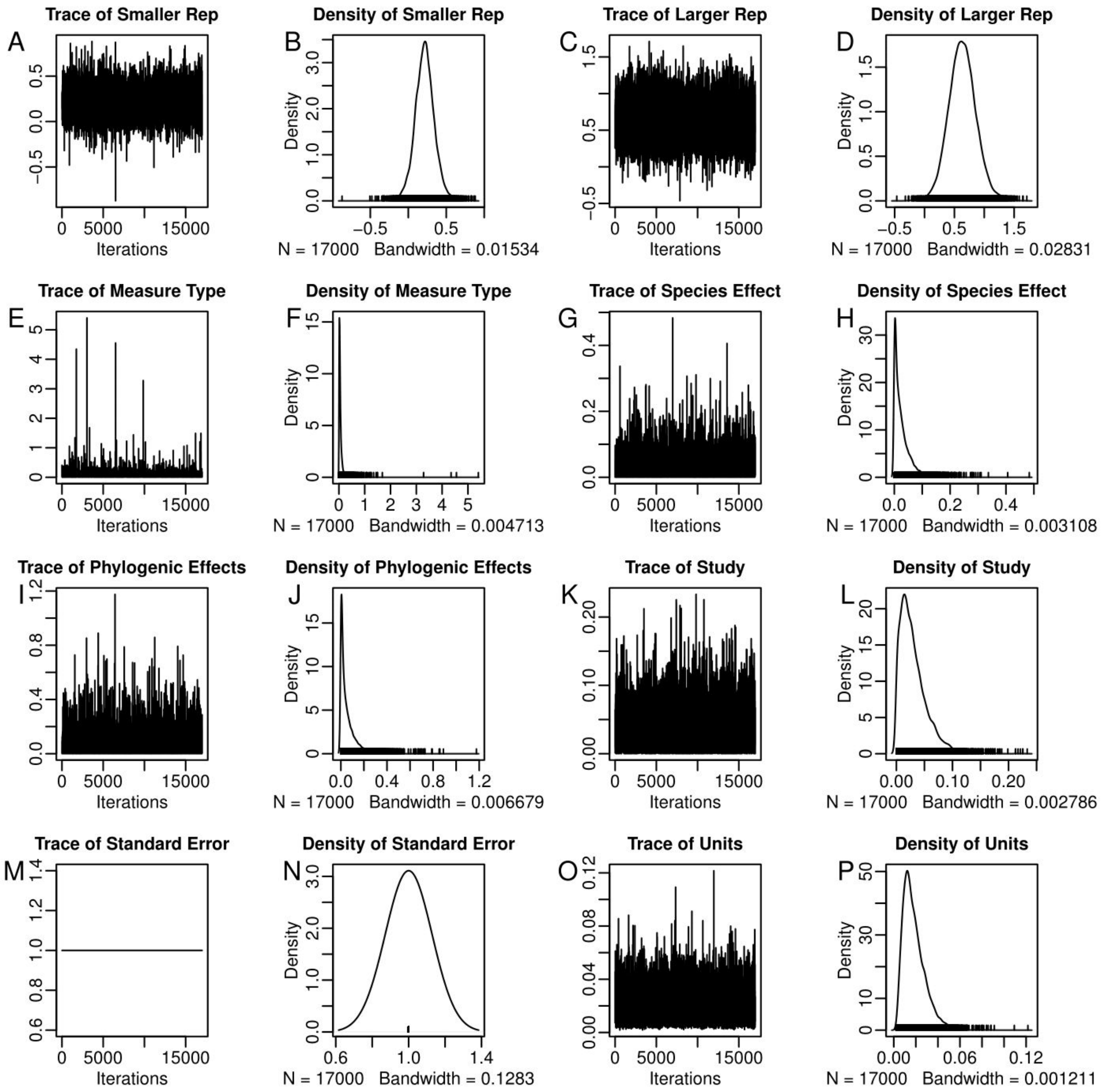

Supplemental Figure SB26: Convergence of parameters in the syllable repertoires model for the song stability data set. Threshold >= 18.5. Annotation is the same as in Supplemental Figure S13, except that the first two pairs of graphs are fixed effects for relatively smaller and larger syllable repertoires. There is no visible trend in the traces that would suggest autocorrelation affected the simulation, and the traces for all terms appear well mixed. The density maps for all random effects terms peak above zero.

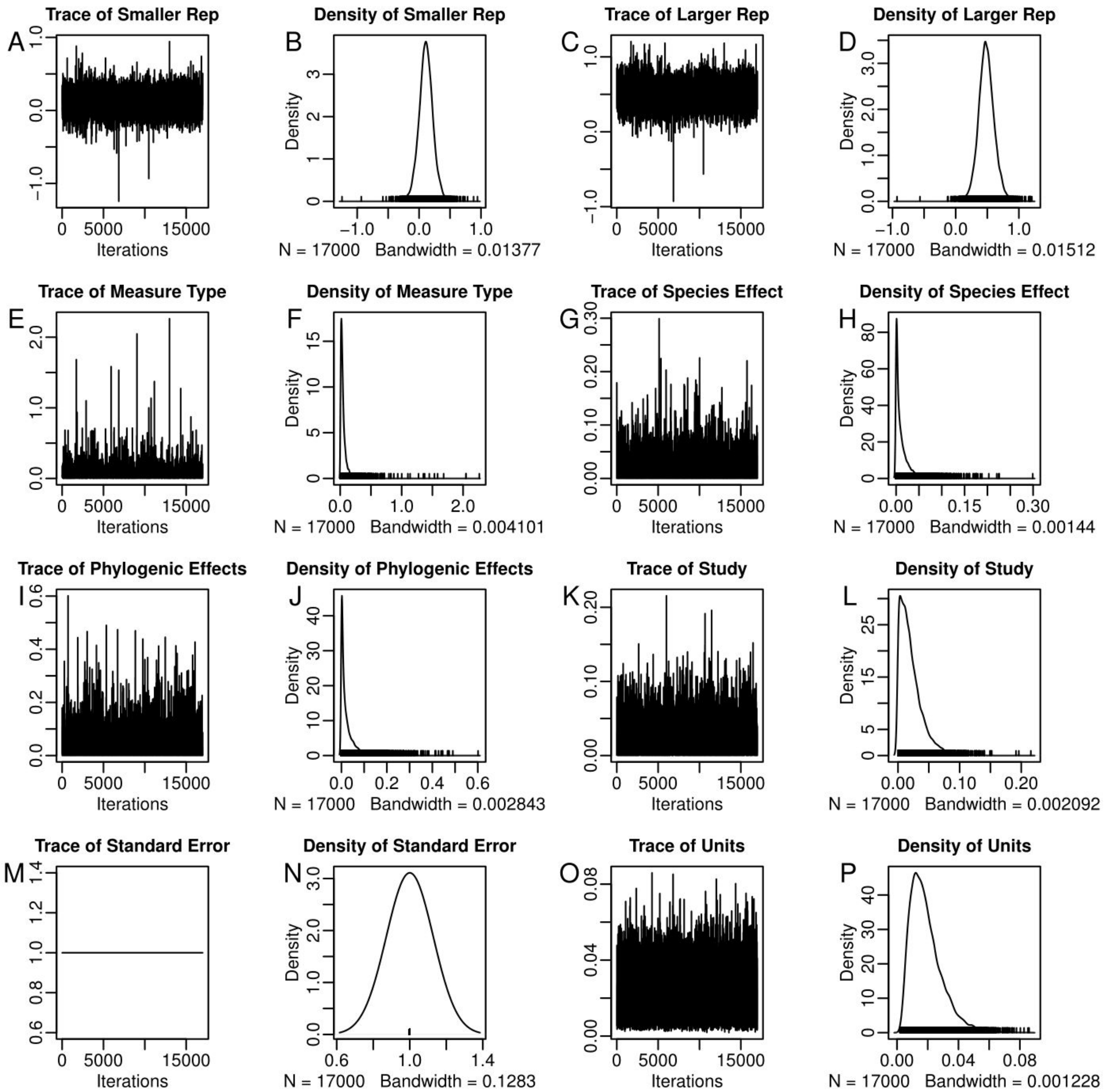

Supplemental Figure SB27: Convergence of parameters in the syllable repertoires model for the song stability data set. Threshold >= 38. Annotation is the same as in Supplemental Figure S13, except that the first two pairs of graphs are fixed effects for relatively smaller and larger syllable repertoires. There is no visible trend in the traces that would suggest autocorrelation affected the simulation, and the traces for all terms appear well mixed. The density maps for all random effects terms peak above zero.

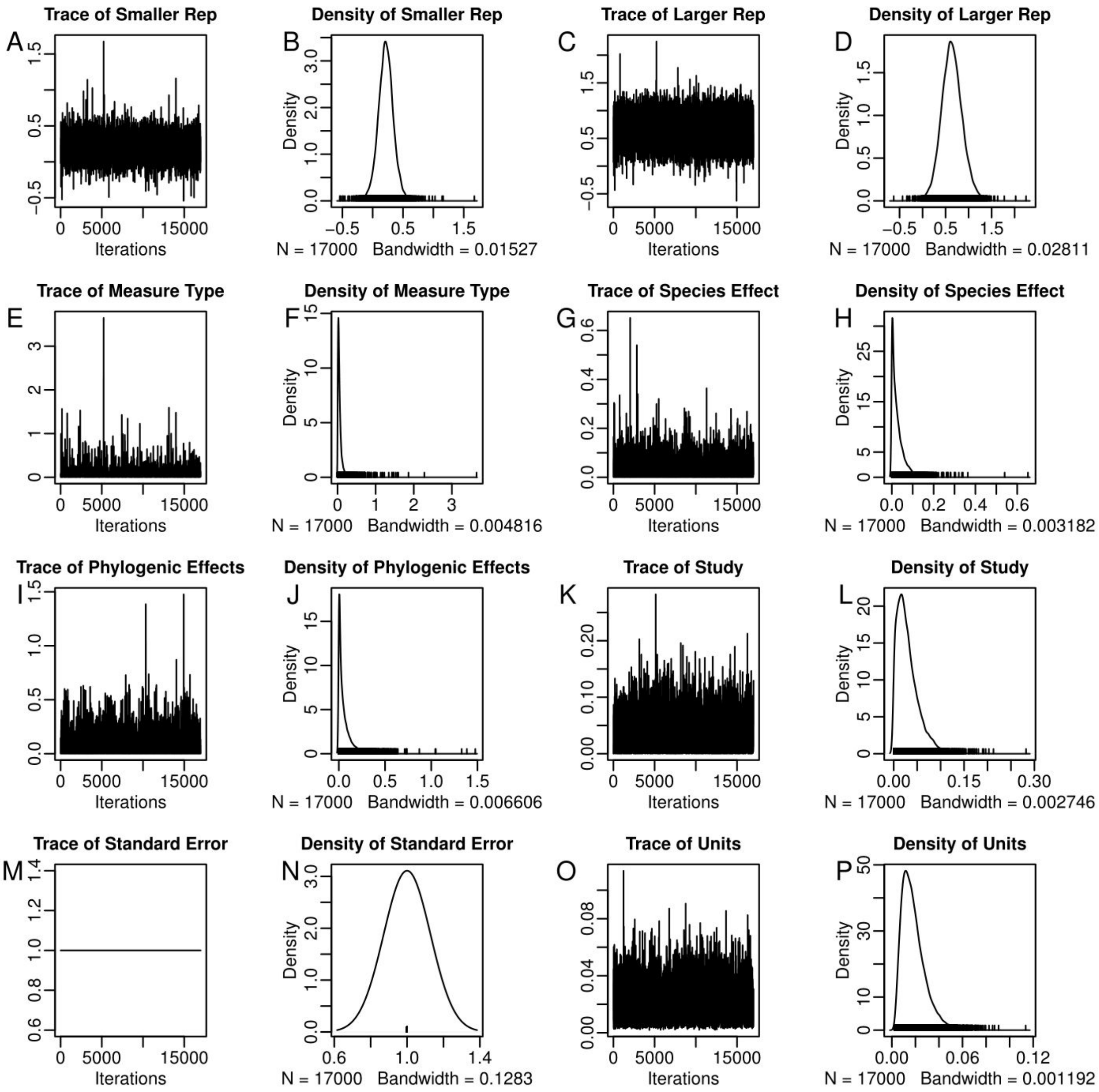

Supplemental Figure SB28: Convergence of parameters in the syllable repertoires model for the song stability data set. Threshold >= 216. Annotation is the same as in Supplemental Figure S13, except that the first two pairs of graphs are fixed effects for relatively smaller and larger syllable repertoires. There is no visible trend in the traces that would suggest autocorrelation affected the simulation, and the traces for all terms appear well mixed. The density maps for all random effects terms peak above zero.

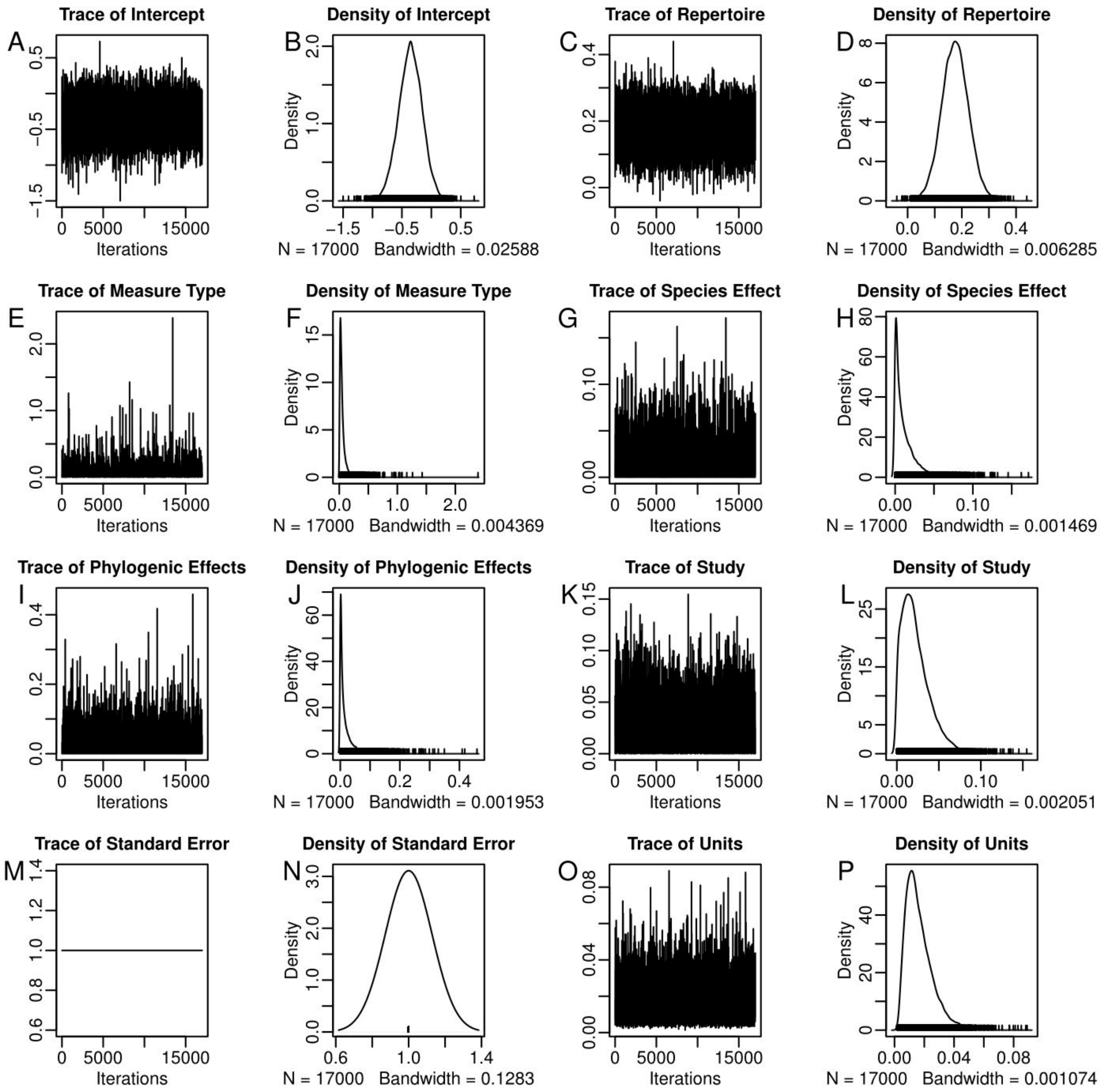

Supplemental Figure SB29: Convergence of parameters in the continuous repertoire size model for the syllable repertoire data set. Annotation is the same as in Supplemental Figure S13, except that the first two pairs of graphs are fixed effects for the intercept and slope (Repertoire). There is no visible trend in the traces that would suggest autocorrelation affected the simulation, and the traces for all terms appear well mixed. The density maps for all random effects terms peak above zero.

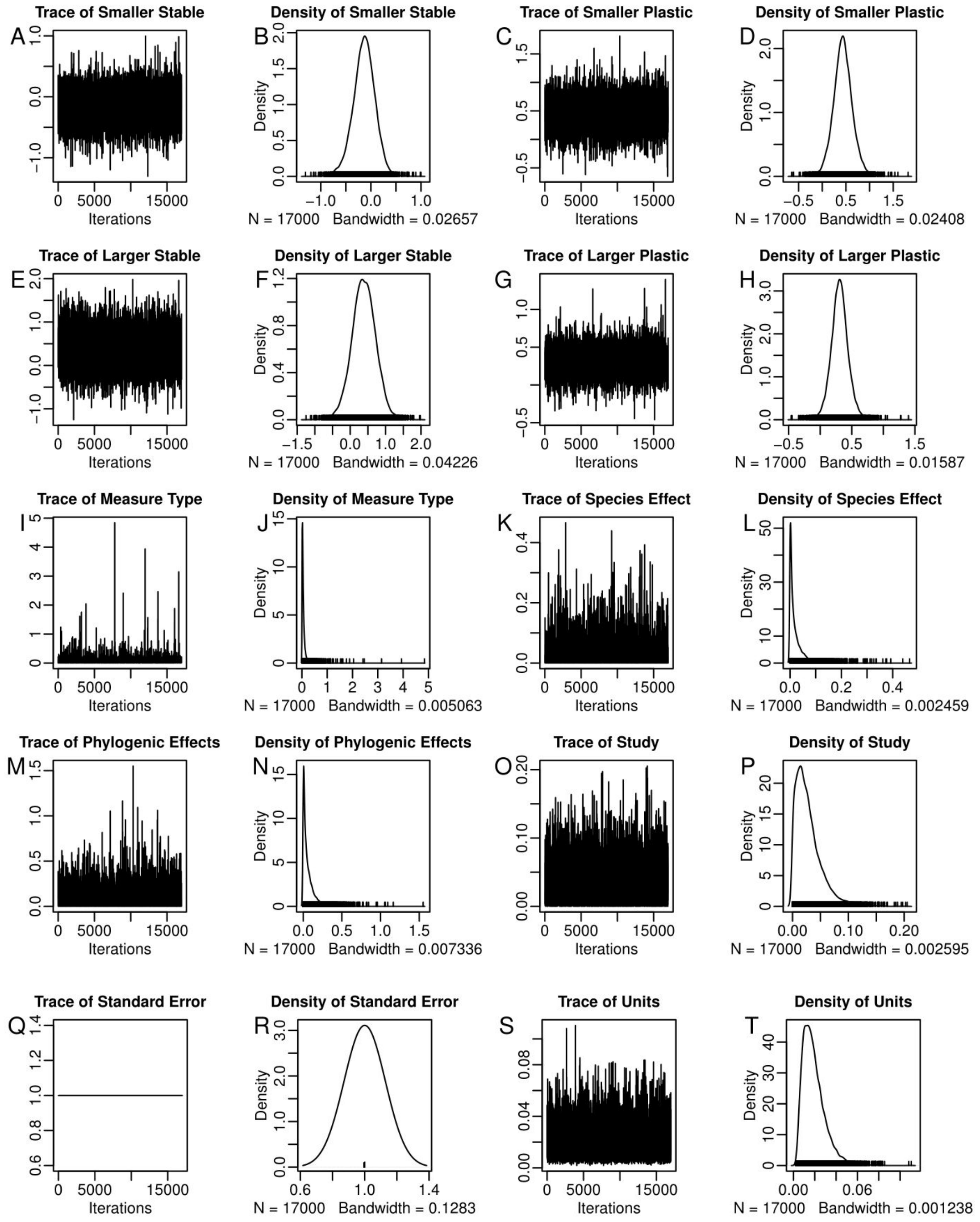

Supplemental Figure SB30: Convergence of parameters in the repertoire size model for the syllable repertoire data set. Threshold >= 18.5. Annotation is the same as in Supplemental Figure S13, except that the first four pairs of graphs are fixed effects for species with smaller syllable repertoires and stable songs, smaller repertoires and plastic songs, larger repertoires and stable songs, and larger repertoires and plastic songs. There is no visible trend in the traces that would suggest autocorrelation affected the simulation, and the traces for all terms appear well mixed. The density maps for all random effects terms peak above zero.

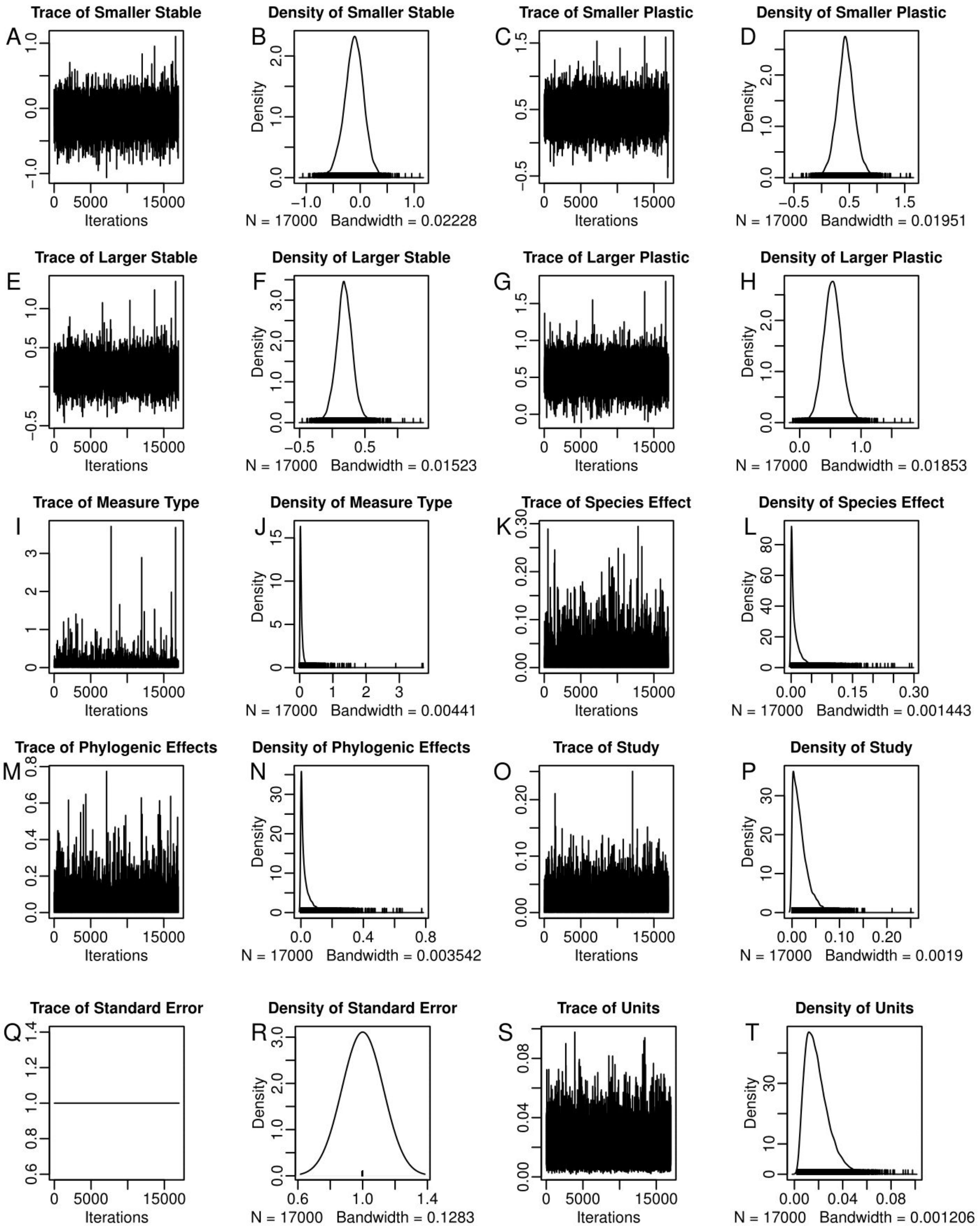

Supplemental Figure SB31: Convergence of parameters in the repertoire size model for the syllable repertoire data set. Threshold >= 38. Annotation is the same as in Supplemental Figure S13, except that the first four pairs of graphs are fixed effects for species with smaller syllable repertoires and stable songs, smaller repertoires and plastic songs, larger repertoires and stable songs, and larger repertoires and plastic songs. There is no visible trend in the traces that would suggest autocorrelation affected the simulation, and the traces for all terms appear well mixed. The density maps for all random effects terms peak above zero.

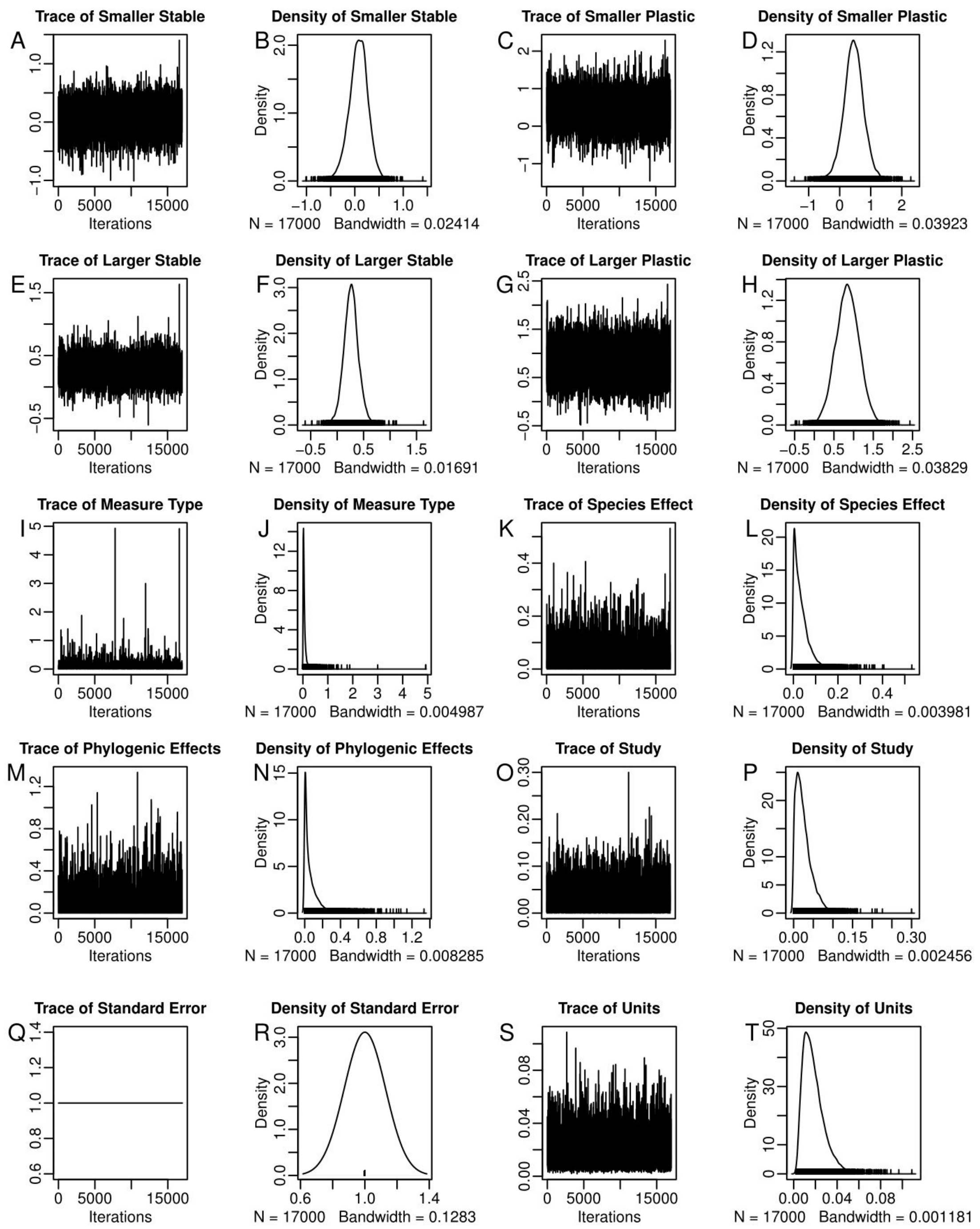

Supplemental Figure SB32: Convergence of parameters in the repertoire size model for the syllable repertoire data set. Threshold >= 216. Annotation is the same as in Supplemental Figure S13, except that the first four pairs of graphs are fixed effects for species with smaller syllable repertoires and stable songs, smaller repertoires and plastic songs, larger repertoires and stable songs, and larger repertoires and plastic songs. There is no visible trend in the traces that would suggest autocorrelation affected the simulation, and the traces for all terms appear well mixed. The density maps for all random effects terms peak above zero.

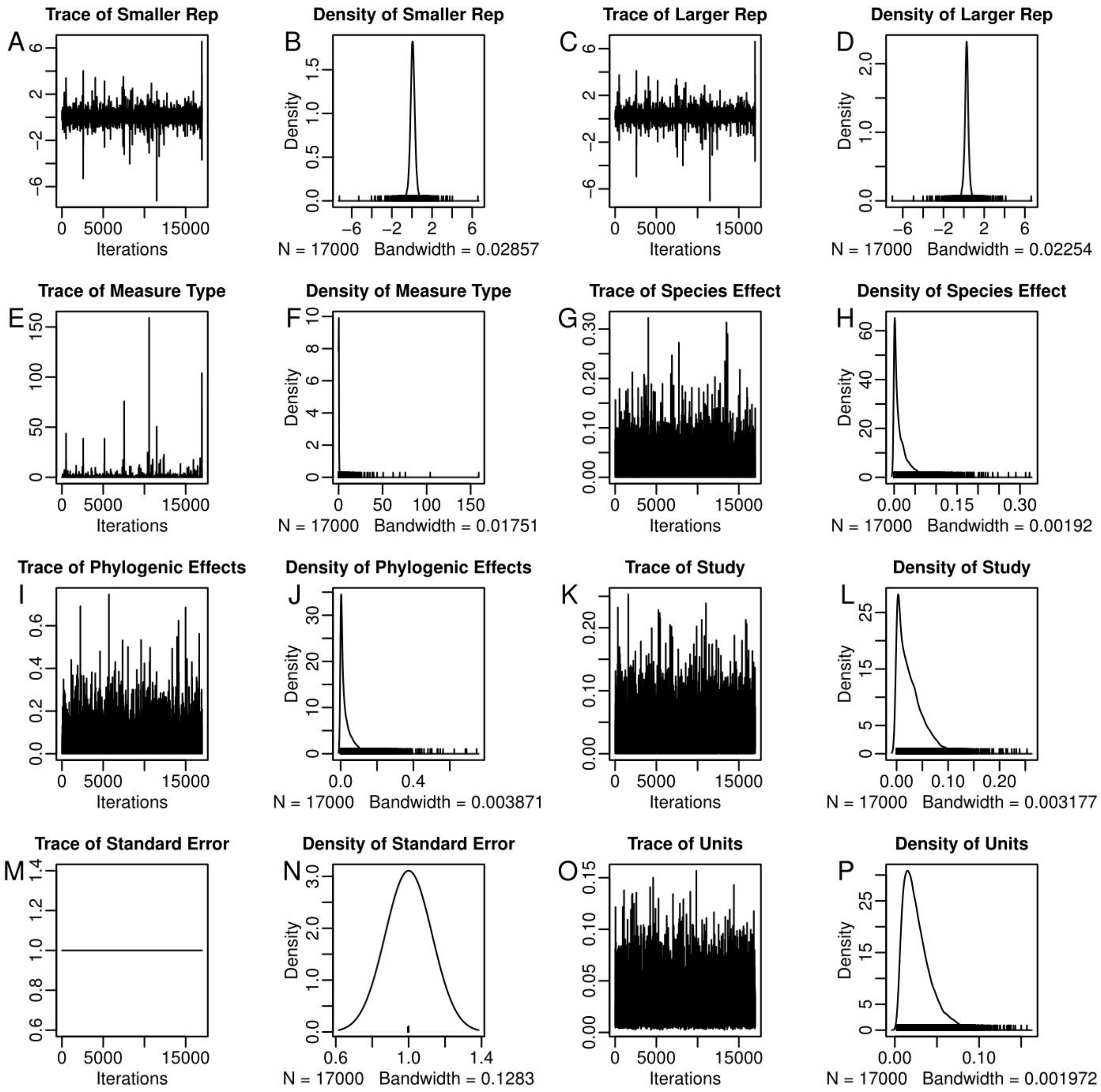

Supplemental Figure SB33: Convergence of parameters in the syllable repertoires model for the no offspring data set. Threshold >= 18.5. Annotation is the same as in Supplemental Figure S13, except that the first two pairs of graphs are fixed effects for relatively smaller and larger syllable repertoires. There is no visible trend in the traces that would suggest autocorrelation affected the simulation, and the traces for all terms appear well mixed. The density maps for all random effects terms peak above zero.

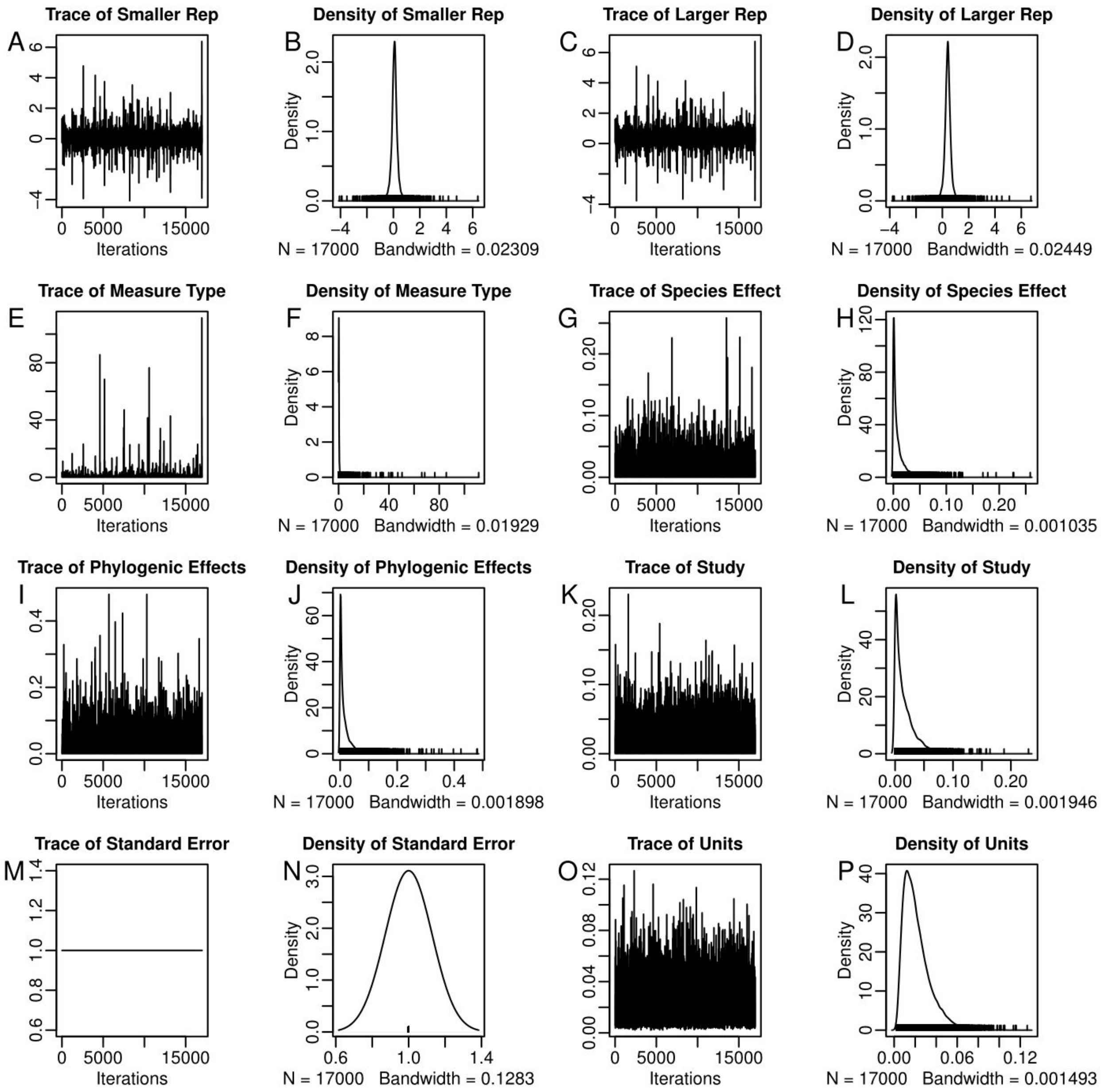

Supplemental Figure SB34: Convergence of parameters in the syllable repertoires model for the no offspring data set. Threshold >= 38. Annotation is the same as in Supplemental Figure S13, except that the first two pairs of graphs are fixed effects for relatively smaller and larger syllable repertoires. There is no visible trend in the traces that would suggest autocorrelation affected the simulation, and the traces for all terms appear well mixed. The density maps for all random effects terms peak above zero.

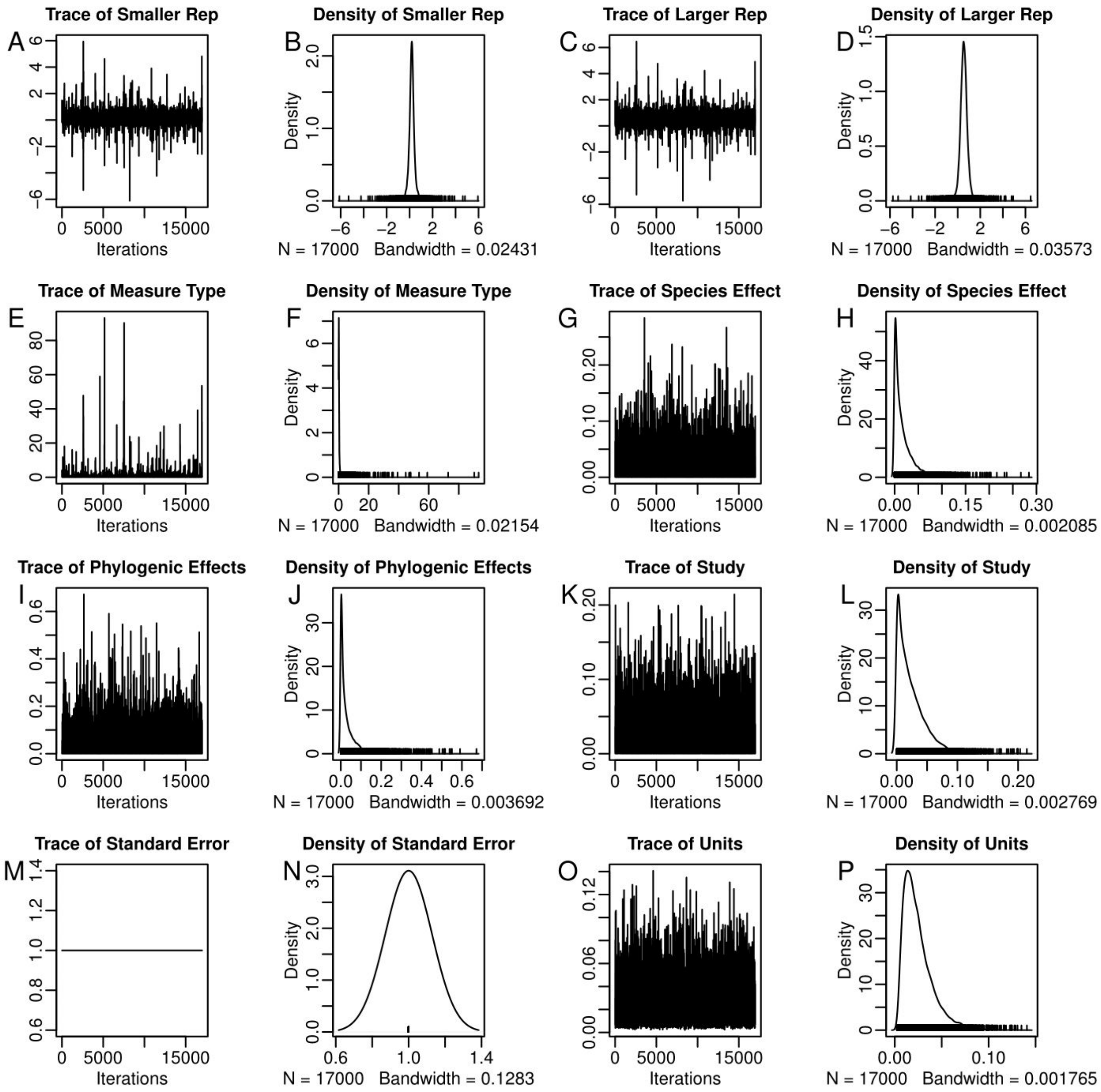

Supplemental Figure SB35: Convergence of parameters in the syllable repertoires model for the no offspring data set. Threshold >= 216. Annotation is the same as in Supplemental Figure S13, except that the first two pairs of graphs are fixed effects for relatively smaller and larger syllable repertoires. There is no visible trend in the traces that would suggest autocorrelation affected the simulation, and the traces for all terms appear well mixed. The density maps for all random effects terms peak above zero.

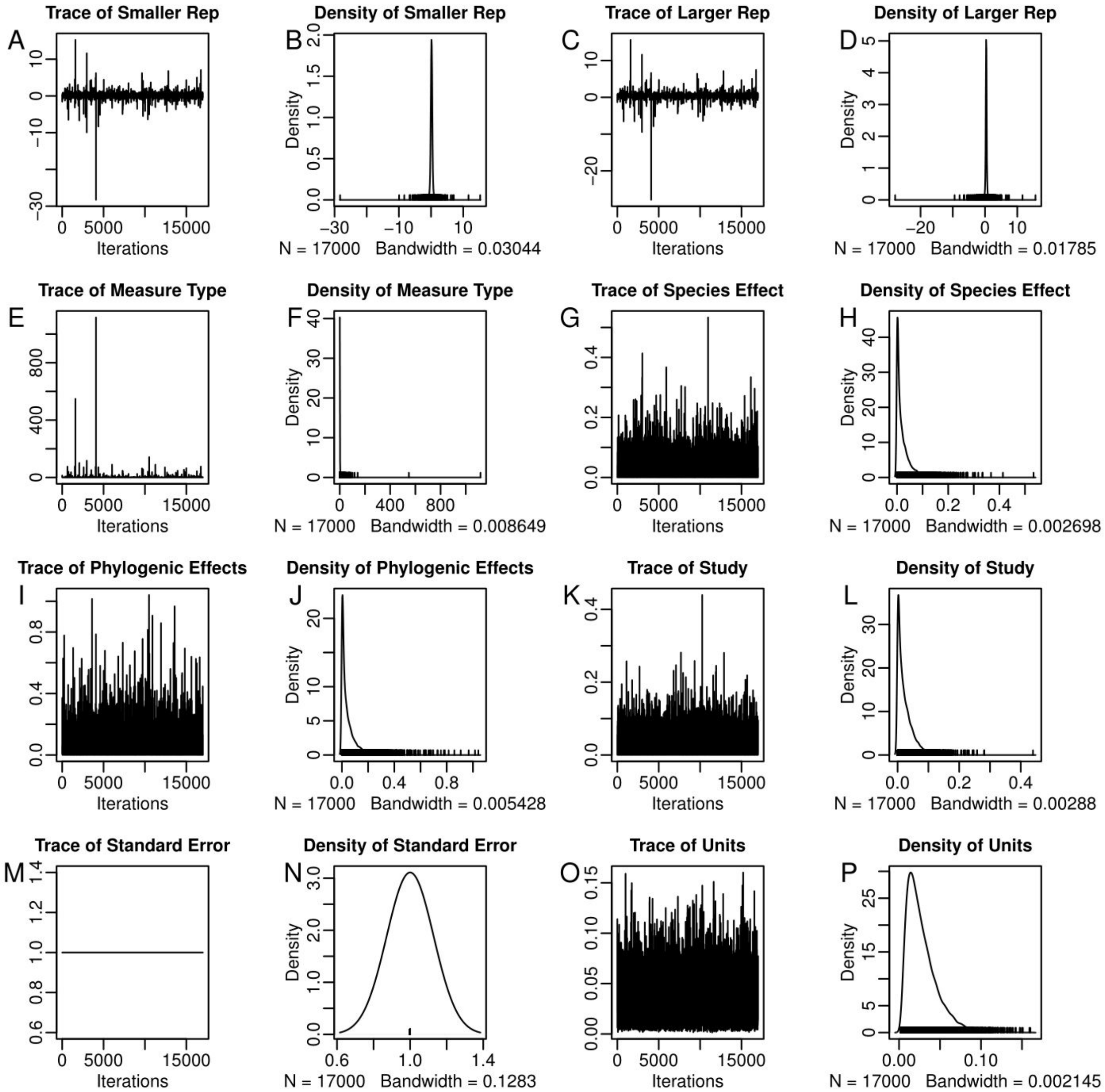

Supplemental Figure SB36: Convergence of parameters in the syllable repertoires model for the no offspring or extra-pair paternity data set. Threshold >= 18.5. Annotation is the same as in Supplemental Figure S13, except that the first two pairs of graphs are fixed effects for relatively smaller and larger syllable repertoires. There is no visible trend in the traces that would suggest autocorrelation affected the simulation, and the traces for all terms appear well mixed. The density maps for all random effects terms peak above zero.

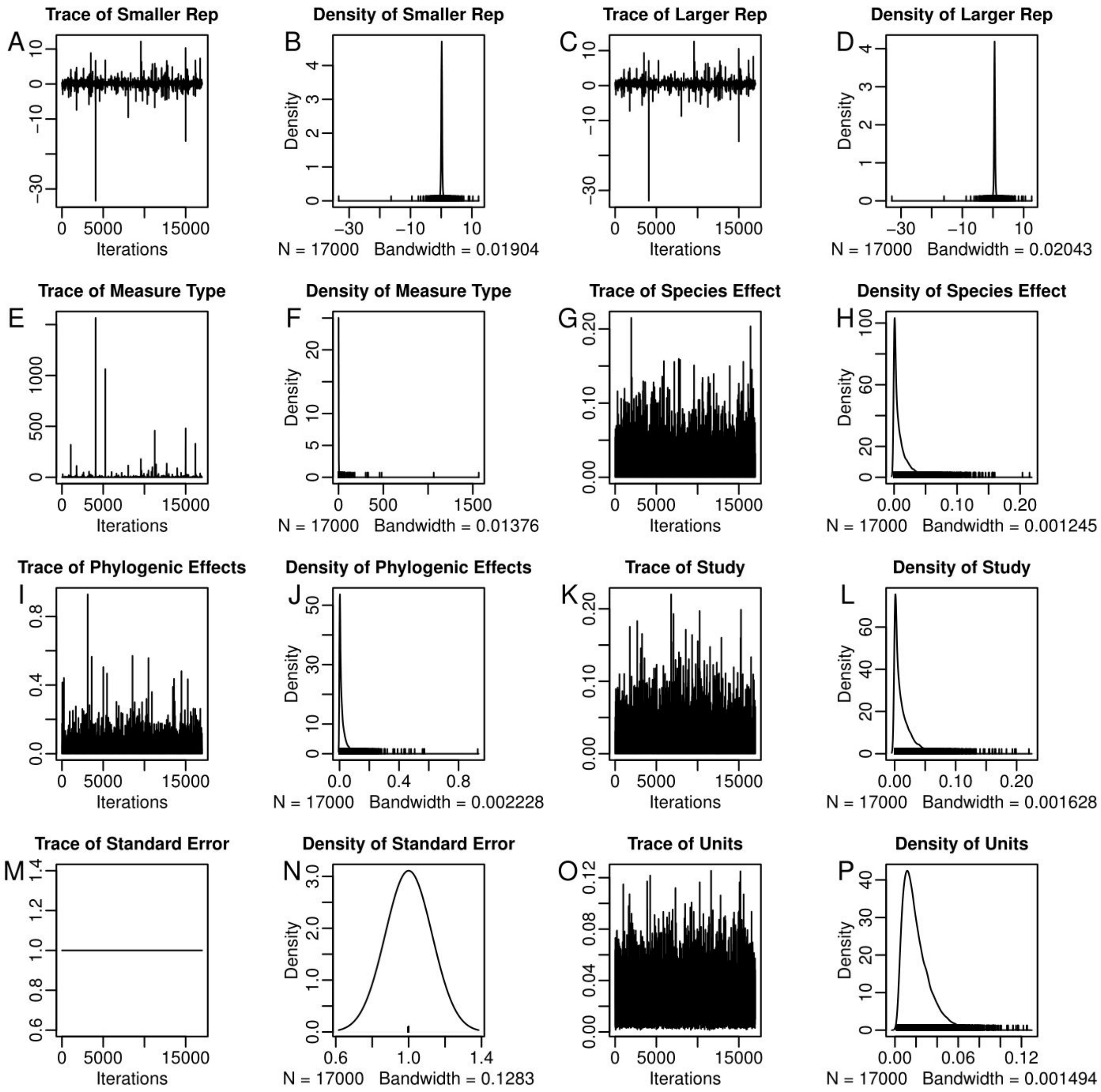

Supplemental Figure SB37: Convergence of parameters in the syllable repertoires model for the no offspring or extra-pair paternity data set. Threshold >= 38. Annotation is the same as in Supplemental Figure S13, except that the first two pairs of graphs are fixed effects for relatively smaller and larger syllable repertoires. There is no visible trend in the traces that would suggest autocorrelation affected the simulation, and the traces for all terms appear well mixed. The density maps for all random effects terms peak above zero.

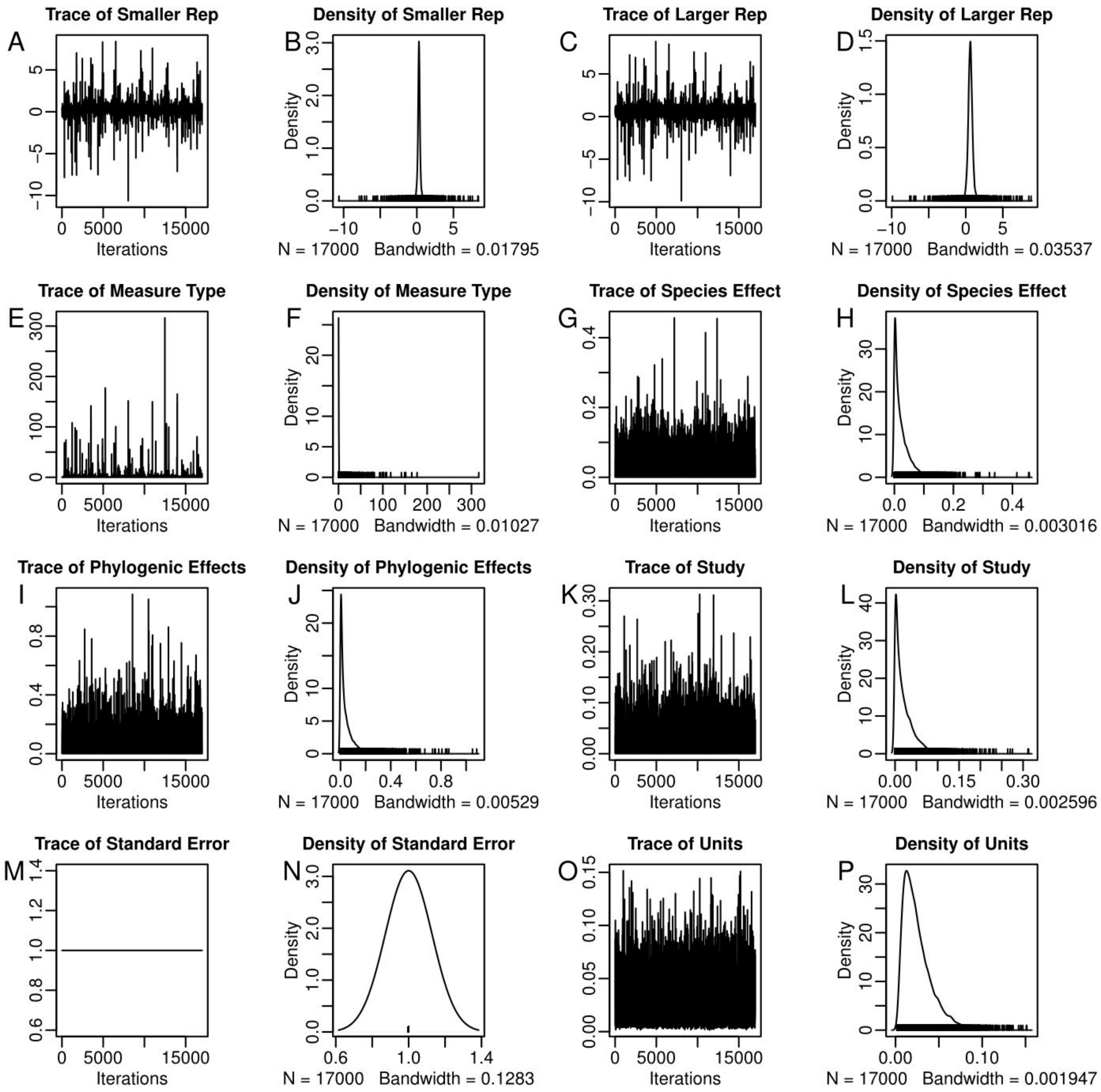

Supplemental Figure SB38: Convergence of parameters in the syllable repertoires model for the no offspring or extra-pair paternity data set. Threshold >= 216. Annotation is the same as in Supplemental Figure S13, except that the first two pairs of graphs are fixed effects for relatively smaller and larger syllable repertoires. There is no visible trend in the traces that would suggest autocorrelation affected the simulation, and the traces for all terms appear well mixed. The density maps for all random effects terms peak above zero.

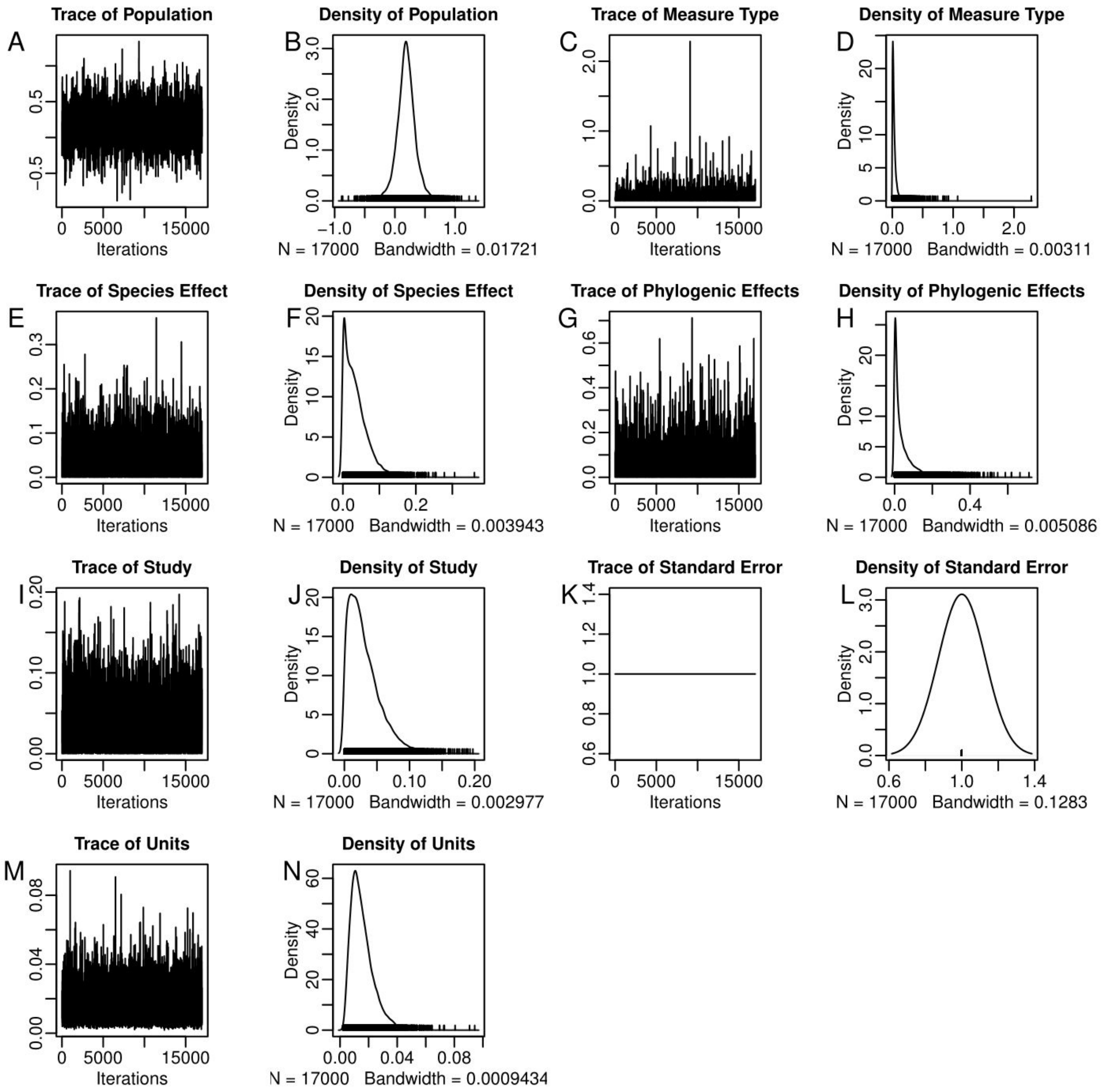

Supplemental Figure SB39: Convergence of parameters in the full population model for the full data set with territory-controlled measurements. Annotation is the same as in Supplemental Figure S13. There is no visible trend in the traces that would suggest autocorrelation affected the simulation, and the traces for all terms appear well mixed. The density maps for all random effects terms peak above zero.

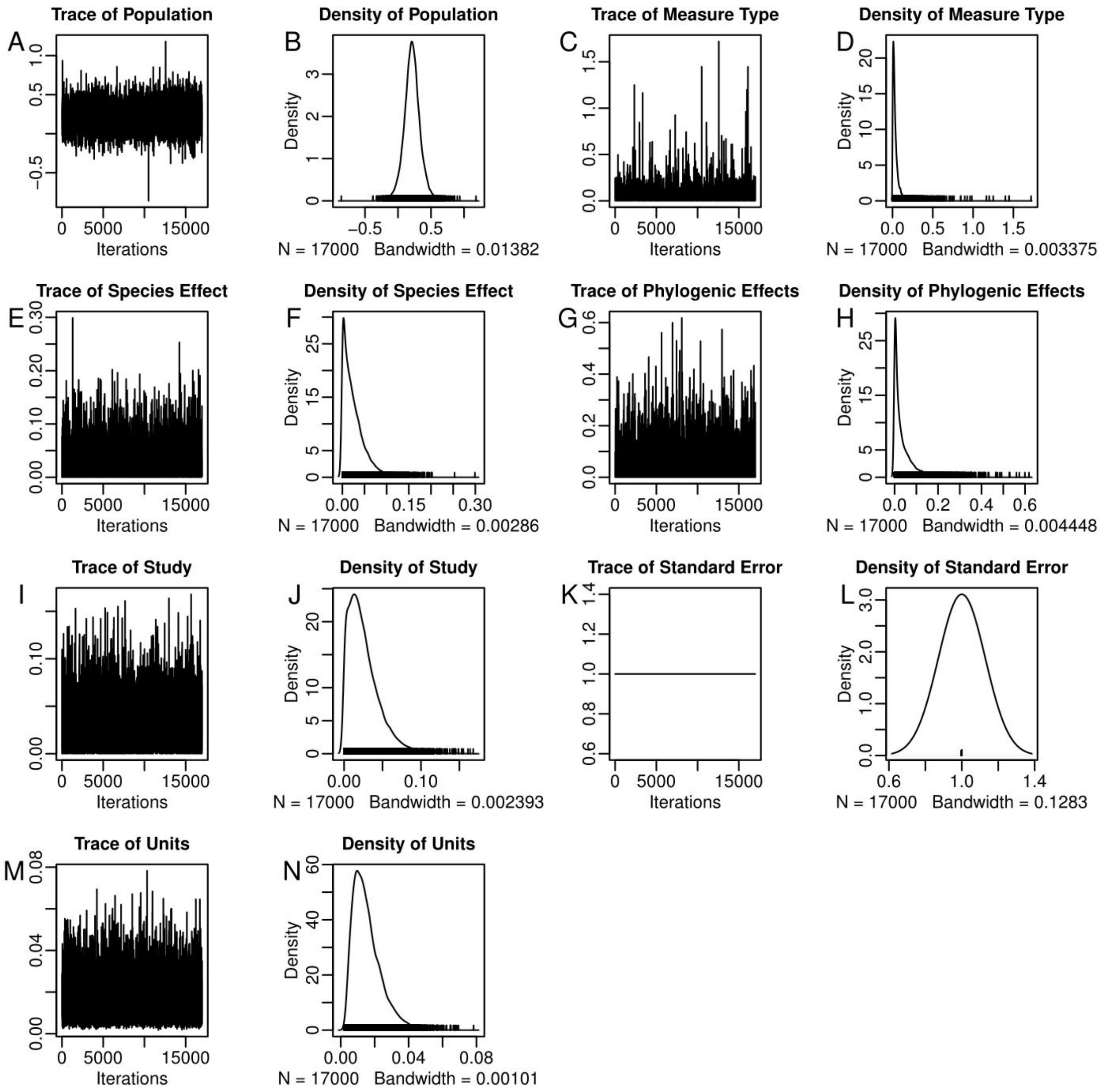

Supplemental Figure SB40: Convergence of parameters in the full population model for the syllable repertoire data set with territory-controlled measurements. Annotation is the same as in Supplemental Figure S13. There is no visible trend in the traces that would suggest autocorrelation affected the simulation, and the traces for all terms appear well mixed. The density maps for all random effects terms peak above zero.

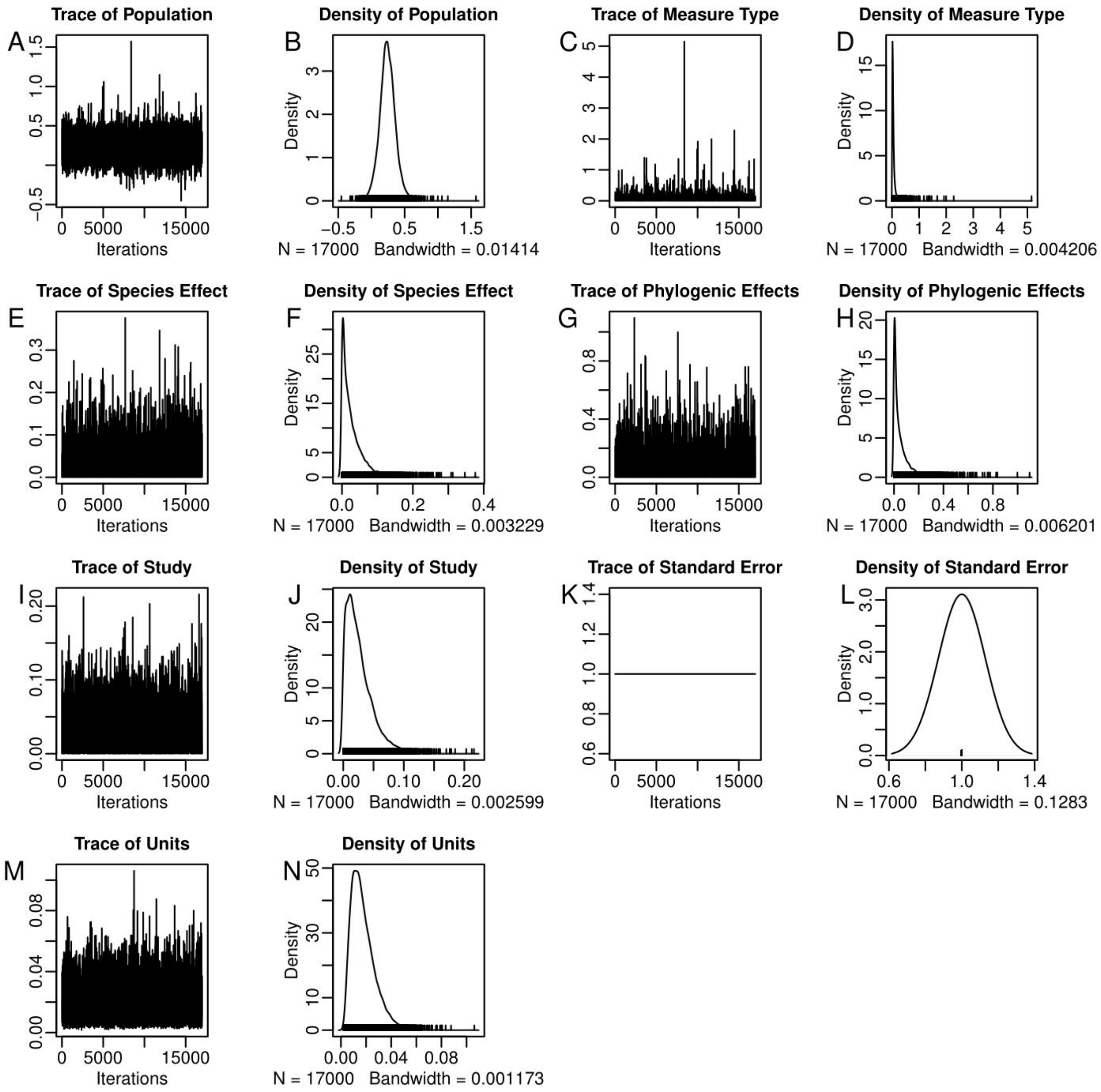

Supplemental Figure SB41: Convergence of parameters in the repertoire size model for the song stability data set. Annotation is the same as in Supplemental Figure S13, except that the first two pairs of graphs are fixed effects for smaller and larger syllable repertoires. There is no visible trend in the traces that would suggest autocorrelation affected the simulation, and the traces for all terms appear well mixed. The density maps for all random effects terms peak above zero.

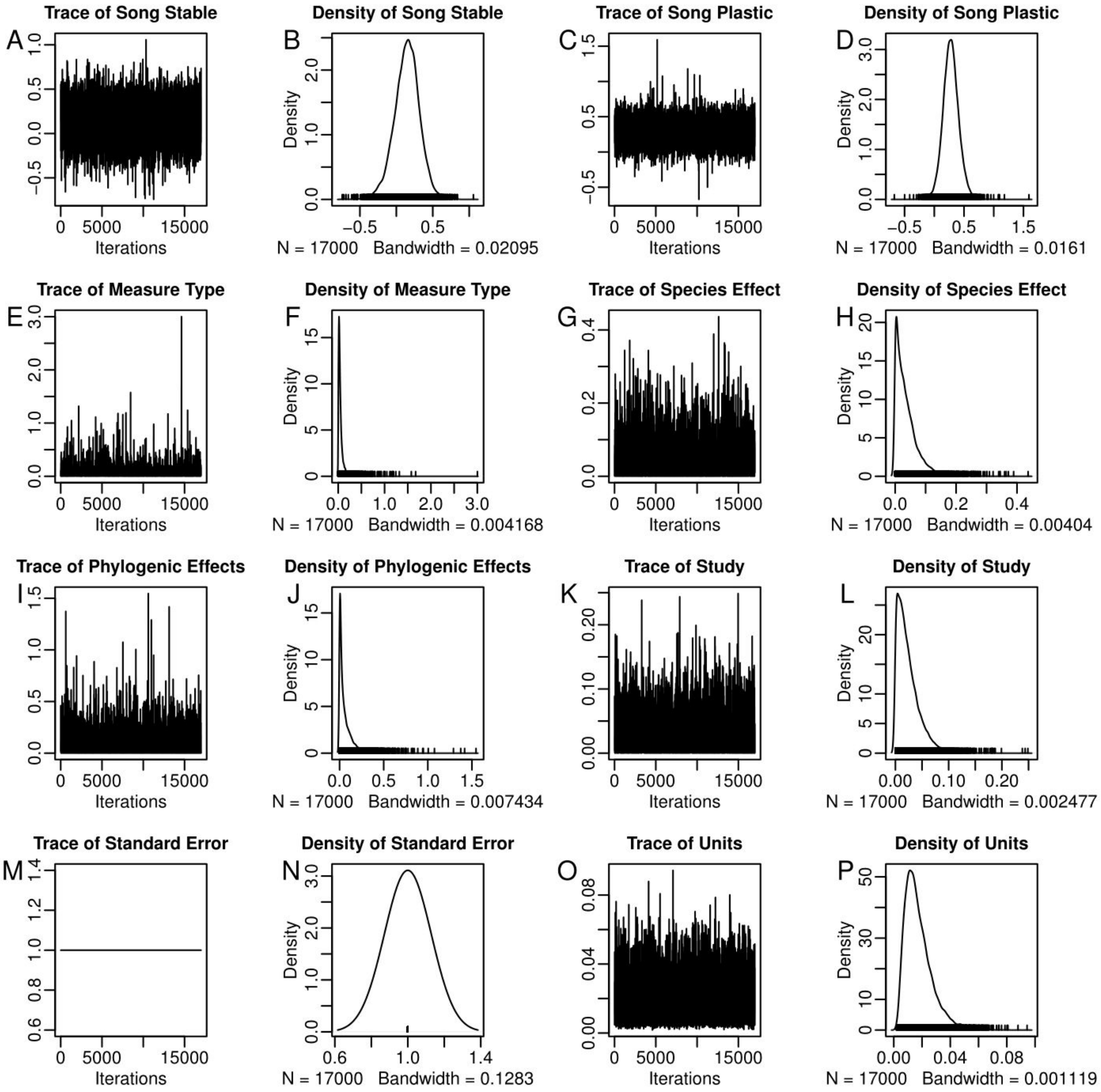

Supplemental Figure SB42: Convergence of parameters in the song stability model for the song stability data set with territory-controlled measurements. Annotation is the same as in Supplemental Figure S13, except that the first two pairs of graphs are fixed effects for song-stable and song-plastic species. There is no visible trend in the traces that would suggest autocorrelation affected the simulation, and the traces for all terms appear well mixed. The density maps for all random effects terms peak above zero.

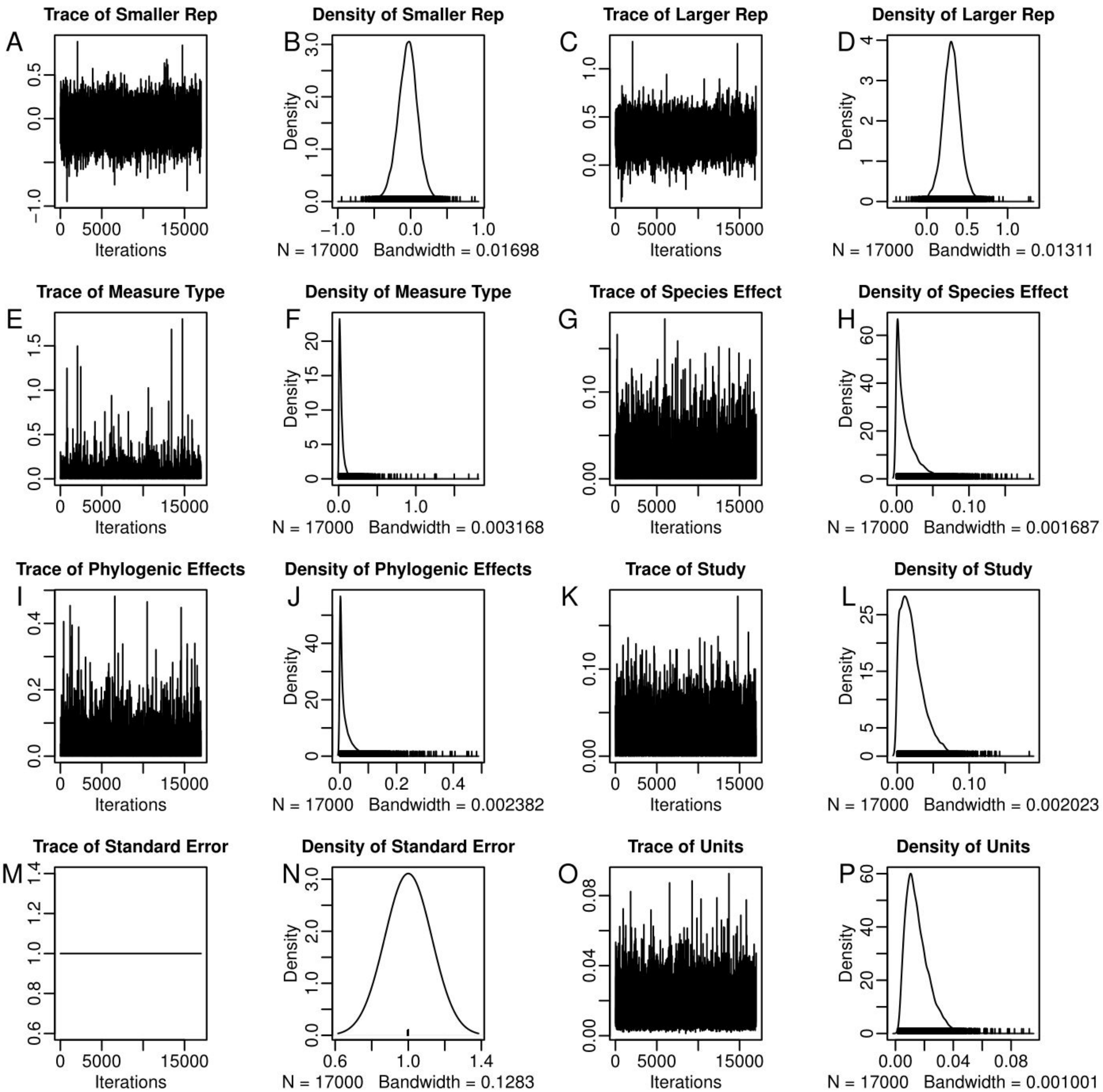

Supplemental Figure SB43: Convergence of parameters in the syllable repertoires model for the syllable repertoire data set with territory-controlled measurements. Threshold >= 18.5. Annotation is the same as in Supplemental Figure S13, except that the first two pairs of graphs are fixed effects for relatively smaller and larger syllable repertoires. There is no visible trend in the traces that would suggest autocorrelation affected the simulation, and the traces for all terms appear well mixed. The density maps for all random effects terms peak above zero.

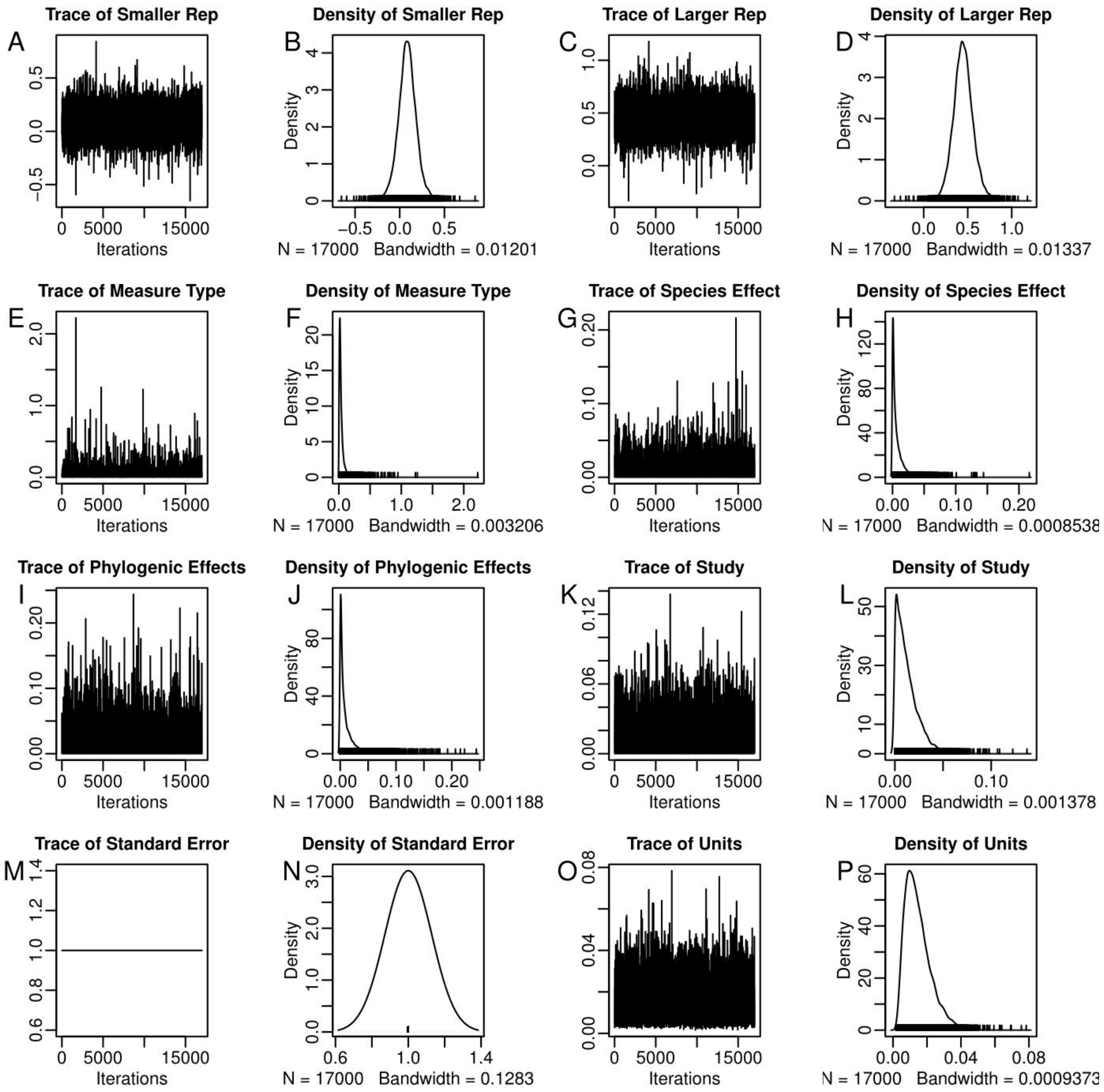

Supplemental Figure SB44: Convergence of parameters in the syllable repertoires model for the syllable repertoire data set with territory-controlled measurements. Threshold >= 38. Annotation is the same as in Supplemental Figure S13, except that the first two pairs of graphs are fixed effects for relatively smaller and larger syllable repertoires. There is no visible trend in the traces that would suggest autocorrelation affected the simulation, and the traces for all terms appear well mixed. The density maps for all random effects terms peak above zero.

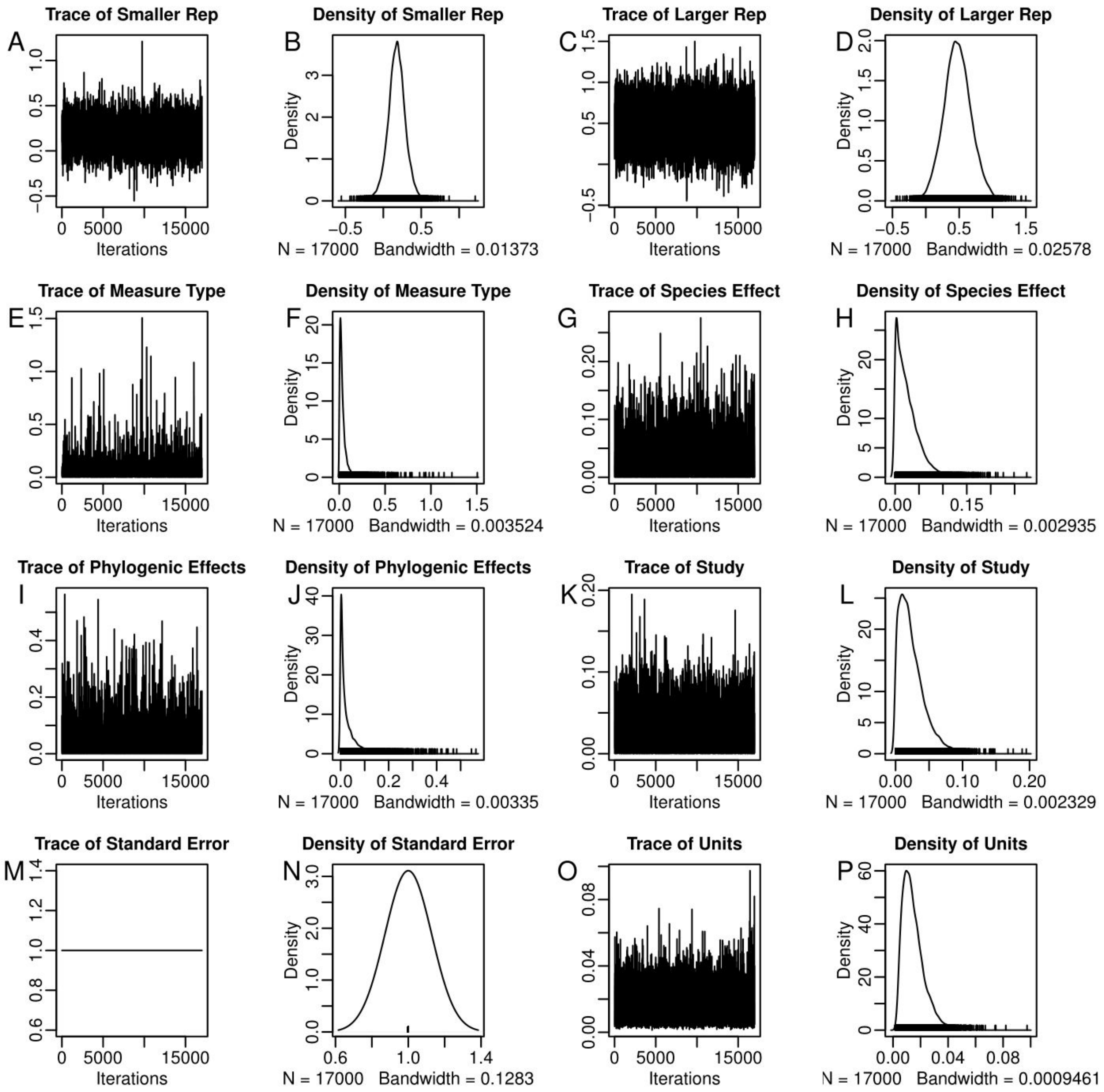

Supplemental Figure SB45: Convergence of parameters in the syllable repertoires model for the syllable repertoire data set with territory-controlled measurements. Threshold >= 216. Annotation is the same as in Supplemental Figure SS13, except that the first two pairs of graphs are fixed effects for relatively smaller and larger syllable repertoires. There is no visible trend in the traces that would suggest autocorrelation affected the simulation, and the traces for all terms appear well mixed. The density maps for all random effects terms peak above zero.
