# Supplementary material for: Species‐level repertoire size predicts a correlation between individual song elaboration and reproductive success
Source: Ecol Evol. 2019 Jul 2;9(14):8362–77. doi: 10.1002/ece3.5418 (PMC6662282; doi:10.1002/ece3.5418)
Supplement: Supplementary file 3 [file ECE3-9-8362-s003.pdf]

Supporting Information for "Species-level repertoire size predicts a correlation between individual song elaboration and reproductive success"

Cristina Robinson and Nicole Creanza

Department of Biological Sciences, Vanderbilt University

__Contents__

**Bayesian Multilevel Phylogenetic Meta-Analysis Methods**	**2**
**Bayesian Multilevel Phylogenetic Meta-Analysis Results**	**4**
**Bayesian Multilevel Phylogenetic Meta-Analysis Discussion**	**12**
**References for Supplemental Results and Discussion**	**14**
**Supplemental Figures**	**16**
**Supplemental Tables**	**30**
**References for studies included in meta-analyses and tables**	**62**
**References for xeno-canto files used for quantifying syllable repertoire size**	**69**

See "Appendix A: Random Effects Meta-Analysis" for the methods, results, tables and figures associated with our original non-Bayesian analysis.

See "Appendix B: Caterpillar Plots" for the plots that assess the convergence of our Bayesian meta-analytic models.

Bayesian Multilevel Phylogenetic Meta-Analysis Methods

Data Collection

We determined estimates for *Wilsonia canadensis* and *Carpodacus mexicanus* (Supplemental Tables S2-3). Average species syllable repertoire size could not be estimated for *Saxicola caprata* or *Phylloscopus trochiloides* (Supplemental Figures S2 and S3), so these species were not included in the species syllable repertoire dataset (see Supplemental Table S4). In the case of *Saxicola caprata*, only a small number of short recordings were available, and many new syllables were still being discovered by the end of the recording. We searched the Macaulay Library[1] database for additional songs, but they were not longer than the recordings on xeno-canto.org. *Phylloscopus trochiloides* is a proposed ring species with very different syllable and song types between regions[2]. We did know the region that each measurement was associated with (India, Siberia, or Kyrgyzstan), but only one or two usable recordings were available for each region, and new syllables were still being discovered at the end of these short recordings. We searched for additional recordings in the Macaulay Library, but did not find longer recordings in the geographic regions of interest.

Bayesian Multi-level Phylogenetic Meta-analysis

We tested meta-analysis models not included in the main text: average species syllable repertoire size (subpopulations with relatively smaller or larger average species repertoires) and a model where both average species syllable repertoire (as subpopulations) and song stability were included as fixed effects to examine potential interactions between these traits. To dichotomize species average syllable repertoire size, we tested all possible thresholds that resulted in at least two species in each group. As stated above, song stability was dichotomized such that species that showed no repertoire changes between their first and second breeding season were defined as being song-stable. These models were designed the same way as described for models in the main analysis.

Controlling for phylogenetic relationships

The main method we used to account for phylogenetic relationships in the main analysis used a consensus tree. This removes any phylogenetic uncertainty. To account for phylogenetic uncertainty, we used the R package mulTree to generate a series of MCMCglmm models based on 100 randomly chosen trees (two chains per tree) from our set of 1000 trees for the following four models: 1) the entire population model in the full dataset, 2) the song stability model in the song stability dataset, and 3) the continuous and 4) discrete species average syllable repertoire size in the species average syllable repertoire dataset. Models were otherwise identical to those above, except that they were run for 400,000 iterations.

Bayesian Multilevel Phylogenetic Meta-Analysis Results

Assessing the variance with a Bayesian meta-analysis

We tested our hypotheses again using a Bayesian meta-analysis. This style of meta-analysis allowed us to pool all of the data into one analysis, because we could control for several aspects of variance and non-independence in the data, including (1) phylogenetic relatedness of species ('Phylo'), (2) non-independence of species that was not linked to phylogeny ('Species'), (3) the non-independence of measures coming from a single study ('Study'), (4) and the variance caused by combining measurements that used different metrics of reproductive success ('MType'). The amount of variance in the data accounted for by each of these random effects terms is estimated using a Markov Chain Monte Carlo simulation. To assess this variance, we ran a series of meta-analytic models, which included each random effects variable alone or in combination with the others using the song stability dataset. When the full population was examined, each random effects variable tested alone accounted for a meaningful amount of variance, but when all random effects variables were included, only the MType and Study terms accounted for a large amount of variance (Table 1). We obtained similar results for this variance assessment when the population was broken into groups by song stability (Supplemental Table S6) or average species syllable repertoire size (Supplemental Tables continuous S7, discrete S8-25). One notable exception to this was that the Species and Phylo terms accounted less variance for moderate syllable repertoire size thresholds (22.5 to 41.95, Supplemental Tables S14-20). Furthermore, the Study term accounted for much less variance when the threshold was set between 38 to 55 (Supplemental Tables S19-21). We kept all four random effects terms in the models, because inclusion of all terms led to markedly lower Deviance Information Criteria (DIC) for all models.

To better understand how measurement type (MType) affected the variance, we tested MType as a fixed effect (Supplemental Table S5). Notably, there was a trend towards significant evidence for a relationship between reproductive success and individual male repertoire size only when the number of females (Posterior Mean=0.407, 95% CreInt=[0.026;0.787], p_{MCMC}=0.038) or number of fledglings (Posterior Mean=0.346, 95% CredInt=[-0.007;0.718], p_{MCMC}=0.053) was measured. Furthermore, while there was no significant evidence for a relationship between reproductive success and individual male repertoire size when extra-pair paternity was measured, the predicted range of correlations for this measure are negatively skewed (Posterior Mean=-0.178, 95% CredInt=[-0.589;0.219], p_{MCMC}=0.322), which is in line with what Soma and Garamszegi[3] had previously reported in their meta-analysis.

Probing the differences between species when species average syllable repertoire is a discrete variable

Species average syllable repertoire size should be cautiously treated as a continuous variable, because each researcher could define syllables slightly differently. In addition, there is a precedent for conducting this type of meta-analysis by separating birds into discrete groups based on song characteristics. In their analysis, Soma and Garamszegi[3] separated species into either 5 bins based on species average song repertoire size, or 3 bins based on species average unique syllables per song, to see if these measures of average species elaboration could predict which species show a correlation between individual song elaboration and reproductive success.

Binning by species average song repertoire size did lead to significant differences, but this was largely discounted, because species average song repertoire size did not account for a significant proportion of the variability in their dataset. Species average syllables per song did not lead to significant results or account for a significant amount of variability.

Therefore, we retested our hypothesis by dividing species in the species average repertoire size dataset into 2 bins, "smaller" and "larger," based on the species syllable repertoire size. There was no clear value at which to divide species into larger and smaller repertoire sizes, so we tested all species average syllable repertoire sizes as the threshold between the bins. We found the posterior mean for the larger species average repertoire bin was significantly separated from 0 for the majority of the repertoire size thresholds, predicting weak correlations when the threshold value was small (e.g. threshold≥18.5, z=0.333) and strong correlations when the threshold was large (threshold≥216, z=0.611) (Figure 5, Supplemental Table 33). The smaller species average repertoire size bin was never significantly separated from 0 (Figure 5, Supplemental Table 33). BEST analysis confirmed it was highly likely that there was a real difference between the larger and smaller bins for all thresholds between 10.75 and 216 syllables and that this difference was substantial (differences in z ranging from 0.238 to 0.376 depending on which significantly different threshold was examined) (Supplemental Table 34). Three species average syllable

repertoire thresholds were of particular interest: 1) at 18.5 syllables the meta-analytic mean for the smaller bin was the most negative, 2) 38 syllables led to the model with the most significant evidence of a strong correlation in the larger repertoire bin, and 3) 216 syllables led to the greatest difference between the meta-analytic means of the smaller and larger species average syllable repertoire bins. We used only these three thresholds for the analyses below.

Jackknife resampling and territory-controlled measurements

Our dataset contained a small number of species, so it was possible that one species with more extreme correlations could skew our results. To account for this, we performed a jackknife analysis where we removed each species from the repertoire size dataset in turn and repeated analysis in the continuous and discrete models. This is did not significantly alter the results of the meta-analysis (Supplemental Tables S28 (continuous), S36-38 (discrete, thresholds 18.5, 28, and 216)). However, when the threshold was set at 216 and either *Emberiza schoeniclus* or *Ficedula albicollis* was removed, the smaller group was trending towards significant evidence of a real effect. Furthermore, there is limited research on song stability, so it was possible that we have miscategorized the song stability of a species. To account for this, we performed a pseudo-jackknife, where the song stability categorization of each species was changed in turn, from stable to plastic or vice versa. This did not significantly alter our results (Supplemental Table S47). Miscategorization of the song stability in *Agelaius phoeniceus*, *Ficedula albicollis*, or *Hirundo rustica* would have led to posterior means that were significantly different from zero

in the plastic group, however the posterior means for the song-plastic and song-stable species post recategorization of each of these species were similar to those from the main analysis.

Finally, in the formation of our dataset, a few measures of the correlation between individual repertoire size and reproductive success were accompanied with a territory-controlled counterpart. We omitted the territory-control measurements in our main datasets, so that all measures would be more comparable. However, in some species, territory quality appears more important than repertoire size in reproductive success (e.g.[4]). Therefore, we repeated all main analyses in datasets where the territory-controlled measurements were included in place of the non-territory-controlled measurements. This did not significantly alter our results (Supplemental Figures S4, 6, 10 and Supplemental Tables S26 (full population), S32 (repertoire size continuous), S43-44 (discrete, thresholds 18.5, 28, and 216)), S48-49 (song stability).

Testing sensitivity to published repertoire size estimates

Measurements of average repertoire size are inherently noisy, because different investigators define syllables differently, and syllables are markedly different between species. Therefore, we repeated the threshold analysis using the highest and lowest values that we encountered for each species in the literature. While switching the repertoire size values did change the overall order of species, it did not have a major effect on the position of individual species, with most birds changing their ranking order by 2 positions or fewer (Supplemental Figure S7-8). Of note, the position of *Emberiza schoeniclus* was noticeably affected by the repertoire size estimate used

(Original position=8, Min position=5, Max position=13), as was the position of *Phylloscopus trochilus* (Original position=15, Min position=15, Max position=20). The meta-analytic results and trends for the dataset using the highest and lowest repertoire size values found in the literature were similar to those seen in our main analysis (Supplemental Tables S31 (continuous), S39-42 (discrete, all thresholds)), suggesting that our results do not depend on the precise repertoire size measurements used or require species to be in the exact order they were arranged.

Permutation test

We next wanted to assess the probability of getting significant results when the data was split into two groups arbitrarily instead of based on published syllable repertoire size. We generated 500 permutations of our data, randomly assigning each species to one of two groups, and performed a meta-analysis with each permutation. We found that 4.4% of the randomized groups (8.8% of the two group models) showed significant (p_{MCMC}<0.025) evidence of a real correlation between song elaboration and reproductive success which was also significantly different from the other group in that same model ($BEST_{\%<0}$<2.5%). In contrast, 14 out of 21 models from the real data were significant (thresholds 18.15 to 216 syllables); in the real data, the middle range of thresholds was significant while the largest and smallest thresholds were not. (Supplemental Figure S9A). Interestingly, the arbitrary significant groups were enriched with species with larger repertoires and had fewer species with smaller repertoires relative to the arbitrary nonsignificant groups (Supplemental Figures S9B). If we reduce our p_{MCMC} threshold for significant results to 0.013—allowing only a 2.5% chance of type I error based on the arbitrary

grouping results—the main analysis results are still significant for 13 out of 21 syllable repertoire thresholds (18.5 to 216 syllables).

Testing for interactions between syllable repertoire size and song stability

Although song stability did not provide predictive value alone, it was possible that in combination with average syllable repertoire size it would provide additional predictive information. Therefore, we next tested for the existence of this interaction by including an interaction term between syllable repertoire size and song stability in the Bayesian meta-analysis; however, breaking the species into four groups led to very small sample sizes that make the results of the model difficult to interpret (Supplemental Table S50). The results tentatively suggest that there may be some additional predictive power of these two variables in combination; species with intermediate to large syllable repertoires combined with plastic songs were most likely to show a correlation between individual repertoire size and reproductive success, while species with smaller syllable repertoires and stable songs were least likely to show this correlation. A larger number of species will need to be studied to make any conclusive statements.

Disentangling mate choice and fecundity as measures of reproductive success

Some of the metrics of reproductive success in our dataset are indicative of mate choice, such as pairing date, and others are more indicative of fecundity, such as number of offspring. In a previous meta-analysis, Byers and Kroodsma[5] specifically addressed mate choice and song

elaboration. Thus, we wanted to test how our results would be affected if we analyzed only proxies of mate choice and omitted proxies of fecundity (number of offspring). This reduced the syllable repertoire dataset to 53 measurements, but all 25 species were still represented. In this model, there was not significant evidence for a correlation between individual repertoire size and success in obtaining status as a social or genetic mate, though it was trending in this direction with the largest threshold of 216 (Supplemental Table S51). Our previous analysis of the metrics of mating success had suggested that the metric for genetic mate choice (extra-pair paternity) actually showed a negative relationship between individual male repertoire size and reproductive success, which hints at the possibility that females choose social mates and extra-pair mates based on different criteria. We ran an additional model that excluded measures related to number of offspring and extra-pair paternity, so that it only included proxies of social mate choice. This reduced the dataset to 42 measurements in 19 species. With this model, there was a trend towards significant evidence of a correlation between individual male repertoire size and success in becoming a social mate when the threshold was set at 38 or 216 (Supplemental Table S52). Interestingly, for both the dataset with social and genetic mate metrics and the dataset with social mate metrics only, the smaller repertoire size group had a smaller meta-analytic mean for all three thresholds, and BEST analysis still predicted that it was highly likely that there was a difference between the real means of the larger and smaller syllable repertoire groups only when the threshold was set at 38 (Supplemental Table S53-54). While we did not observe a significant correlation between song elaboration and mate choice, the trends in our BEST results leave open the possibility that, given a larger dataset, there would be an association between song elaboration and mate choice.

Bayesian Multilevel Phylogenetic Meta-Analysis Discussion

Larger species average syllable repertoires have been proposed to be associated with other life history traits, which suggests the possibility that these other traits may also be predictive of a correlation between individual song elaboration and reproductive success. It is predicted that sexual selection is more intense in polygynous species, where one male is socially mated to multiple females, or in species with high rates of extra-pair paternity (EPP)[6–10]. However, Soma and Garamszegi[3] had shown previously that there were not significant differences between species based on mating system or rate of EPP. This argues against the possibility that selection for larger repertoire size is universally more intense in these species. Alternatively, some species engage in reproductive synchrony, where all females are receptive to mating at the same time to prevent a single male from monopolizing mating attempts with all females[11,12]. These species may be more likely to exhibit a preference for larger repertoires, perhaps because sexual selection is intensified during periods of breeding synchrony[11,12] (but see[13]) and song might be a salient signal for females in these mate choice decisions. It has also been shown in some species that migratory groups have larger syllable average repertoire sizes than sedentary counterparts[2,14–16] (but see[17]). In our original random effects meta-analysis, we found that migratory behavior was not predictive for the strength of the correlation between individual song elaboration and reproductive success (Appendix A: Figure SA5), but our dataset contains

relatively few sedentary species. Data from more sedentary species will be required to conclusively test this hypothesis.

While testing our model, we found that the different metrics of reproductive success were predicted to have different meta-analytic means, perhaps because some of these metrics indicate fecundity while others assess mate choice. Furthermore, we noted that only EPP showed a negative, though nonsignificant, meta-analytic mean, suggesting that females may use different criteria to choose social and extra-pair mates. We investigated this possibility using a data set that only contained measures of mate choice (excluding measures of fecundity), and another that contained only measures of social mate choice (excluding measures of fecundity and EPP). Both of these new meta-analytic models were similar to one another, but, in contrast to the main model, led to non-significant results. It is not clear whether fecundity measurements drove the effect detected in original model, or if there were simply not enough measurements remaining in the reduced mate choice datasets to achieve significant results. More data will be required to determine whether the correlation between reproductive success and individual song elaboration is linked to fecundity, mate choice, or both. Nonetheless, it seems unlikely that fecundity alone would drive the correlation between individual song elaboration and reproductive success, because if males with larger repertoires are more fecund, then there would be a reproductive

benefit to be gained by females that prefer larger repertoires, thus predicting a correlation with mate choice as well.

References for Supplemental Results and Discussion

1. Cornell University Cornell Lab of Ornithology. *Macaulay Library: Archive of Animal Sounds and Video*. (2009).
2. Irwin, D. E. Song variation in an avian ring species. *Evolution* **54**, 998 (2000).
3. Soma, M. & Garamszegi, L. Z. Rethinking birdsong evolution: meta-analysis of the relationship between song complexity and reproductive success. *Behav. Ecol.* **22**, 363–371 (2011).
4. Howard, R. D. The influence of sexual selection and interspecific competition on mockingbird song (Mimus polyglottos). *Evolution* **28**, 428–438 (1974).
5. Byers, B. E. & Kroodsma, D. E. Female mate choice and songbird song repertoires. *Anim. Behav.* **77**, 13–22 (2009).
6. Catchpole, C. K. & Slater, P. J. B. *Bird Song: Biological Themes and Variations*. (Cambridge University Press, 2003).
7. Emlen, S. & Oring, L. Ecology, sexual selection, and the evolution of mating systems. *Science* **197**, 215–223 (1977).
8. Vedder, O., Komdeur, J., van der Velde, M., Schut, E. & Magrath, M. J. L. Polygyny and extra-pair paternity enhance the opportunity for sexual selection in blue tits. *Behav. Ecol. Sociobiol.* **65**, 741–752 (2011).
9. Freeman-Gallant, C. R., Wheelwright, N. T., Meiklejohn, K. E., States, S. L. & Sollecito, S. V. Little effect of extrapair paternity on the opportunity for sexual selection in Savannah sparrows (Passerculus sandwichensis). *Evolution* **59**, 422–430 (2005).

10. Snyder, K. T. & Creanza, N. Polygyny is linked to accelerated birdsong evolution but not to larger song repertoires. *Nat. Commun.* **10**, 884 (2019).

11. Birkhead, T. R. & Biggins, J. D. Reproductive Synchrony and Extra-pair Copulation in Birds. *Ethology* **74**, 320–334 (2010).

12. Yezerinac, S. M. & Weatherhead, P. J. Reproductive synchrony and extra-pair mating strategy in a socially monogamous bird,Dendroica petechia. *Anim. Behav.* **54**, 1393–1403 (1997).

13. Ims, R. A. The potential for sexual selection in males: Effect of sex ratio and spatiotemporal distribution of receptive females. *Evol. Ecol.* **2**, 338–352 (1988).

14. Read, A. F. & Weary, D. M. The evolution of bird song: comparative analyses. *Philos. Trans. R. Soc. Lond. B Biol. Sci.* **338**, 165–187 (1992).

15. Mountjoy, D. J., James Mountjoy, D. & Leger, D. W. Vireo song repertoires and migratory distance: three sexual selection hypotheses fail to explain the correlation. *Behav. Ecol.* **12**, 98–102 (2001).

16. Collins, S. A., de Kort, S. R., Pérez-Tris, J. & Tellería, J. L. Migration strategy and divergent sexual selection on bird song. *Proc. Biol. Sci.* **276**, 585–590 (2009).

17. Catchpole, C. K. & McGregor, P. K. Sexual selection, song complexity and plumage dimorphism in European buntings of the genus Emberiza. *Anim. Behav.* **33**, 1378–1380 (1985).

Supplemental Figures

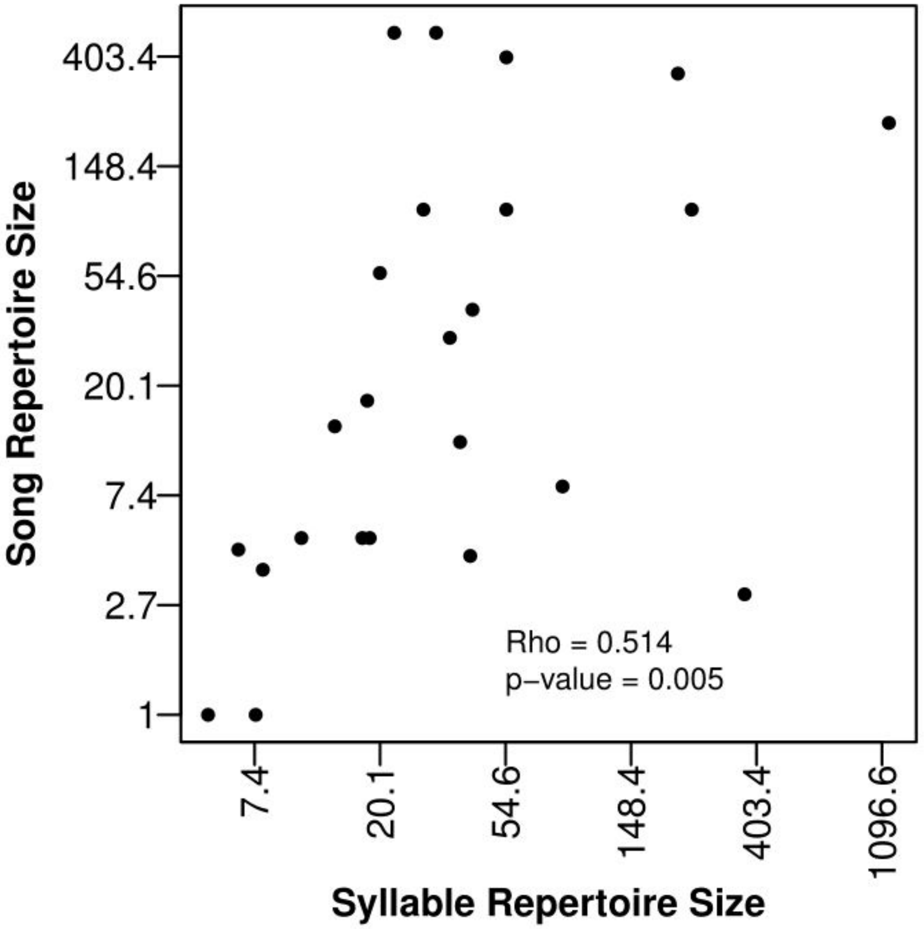

Supplemental Figure S1: Song and syllable repertoire size are correlated between species. Each dot represents a species from this study. All species for which we had syllable repertoire measurements were included except *Phylloscopus fuscatus*, for which we did not have a measurement of song repertoire size. Spearman's rho and associated p-value included on plot. Song repertoire data obtained from Snyder and Creanza[10]. Specific references for each species are found in the supplemental datafile for the analysis, where numbering matches the supplemental references at the end of this document.

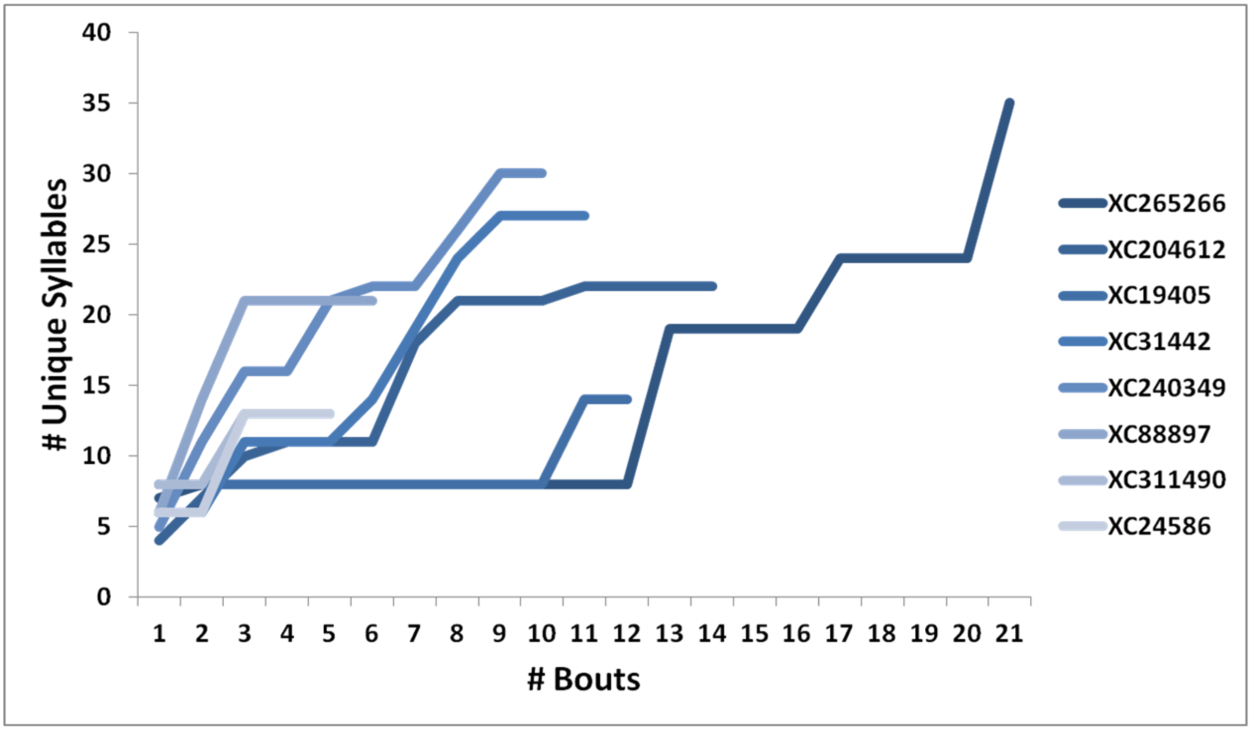

Supplemental Figure S2: Syllable repertoire estimates of Pied Bush Chat (*Saxicola caprata*) for rarefaction curves. Recordings ranged from were only 40 seconds to 2.5 minutes, however even in the longer recordings, new syllables were still being encountered in the last 30 seconds of recording. Based on this evidence, we were not convinced that we had complete repertoire data for this species and thus did not include it in our syllable repertoire size dataset.

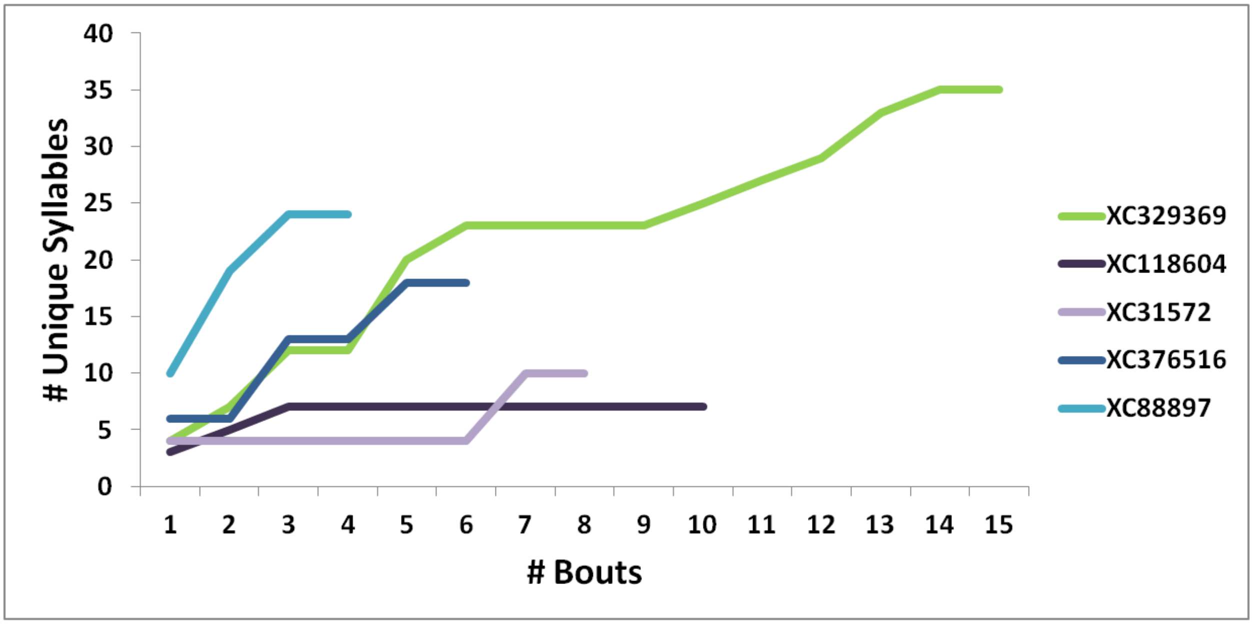

Supplemental Figure S3: Syllable repertoire estimates of Greenish Warbler (*Phylloscopus trochiloides*) for rarefaction curves. Green line is the recording for Kyrgyzstan, blue lines are Russian recordings, and purple lines are Indian recordings. Curves suggest that the recordings were not long enough to capture the full repertoire. While the longest recording (Kyrgyzstan) was ~2.5 minutes, the remaining recordings were only 40-70 seconds, and the intervals between songs in these recordings was often between 5-8 seconds. Based on this evidence, we were not convinced that we had complete repertoire data for this species and thus did not include it in our syllable repertoire size dataset.

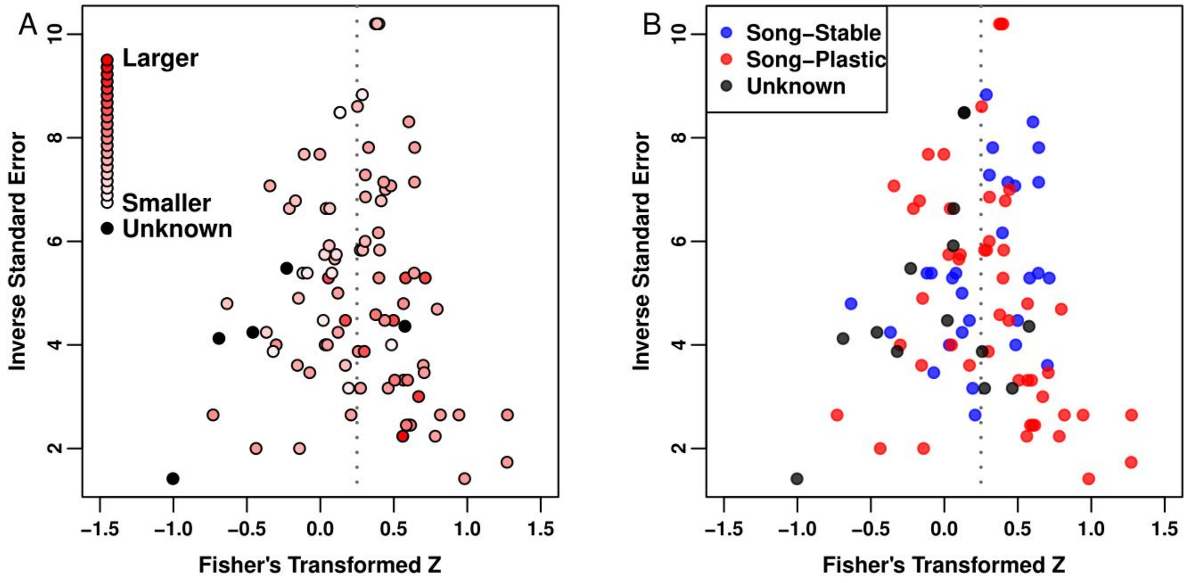

Supplemental Figure S4: Funnel plots show the 91 measurements of the correlation between song elaboration and reproductive success from the full dataset used in the Bayesian meta-analysis presented in the main text, here performed with territory controlled-measurements. The grey dotted line represents the mean Fisher's transformed *Z*. (A) Circle color becomes more red as the repertoire size of the species increases. Black circles show measurements from species for which the syllable repertoire size is unknown. (B) Blue circles indicate measures from song-stable species, while red circles indicate measurements from song-plastic species. Black circles denote species for which no song stability information was available. Regression testing on the full data set (z=0.8393, p=0.4013), syllable repertoire dataset (z=1.4602, p=0.1442), or song stability dataset (z=1.3952, p=0.1630) revealed no significant funnel plot asymmetry. Ranked correlation testing on the full data set (τ=0.0168, p=0.8156), syllable repertoire dataset (τ=0.0472, p=0.5233), or song stability dataset (τ=0.0345, p=0.6598) also revealed no significant funnel plot asymmetry.

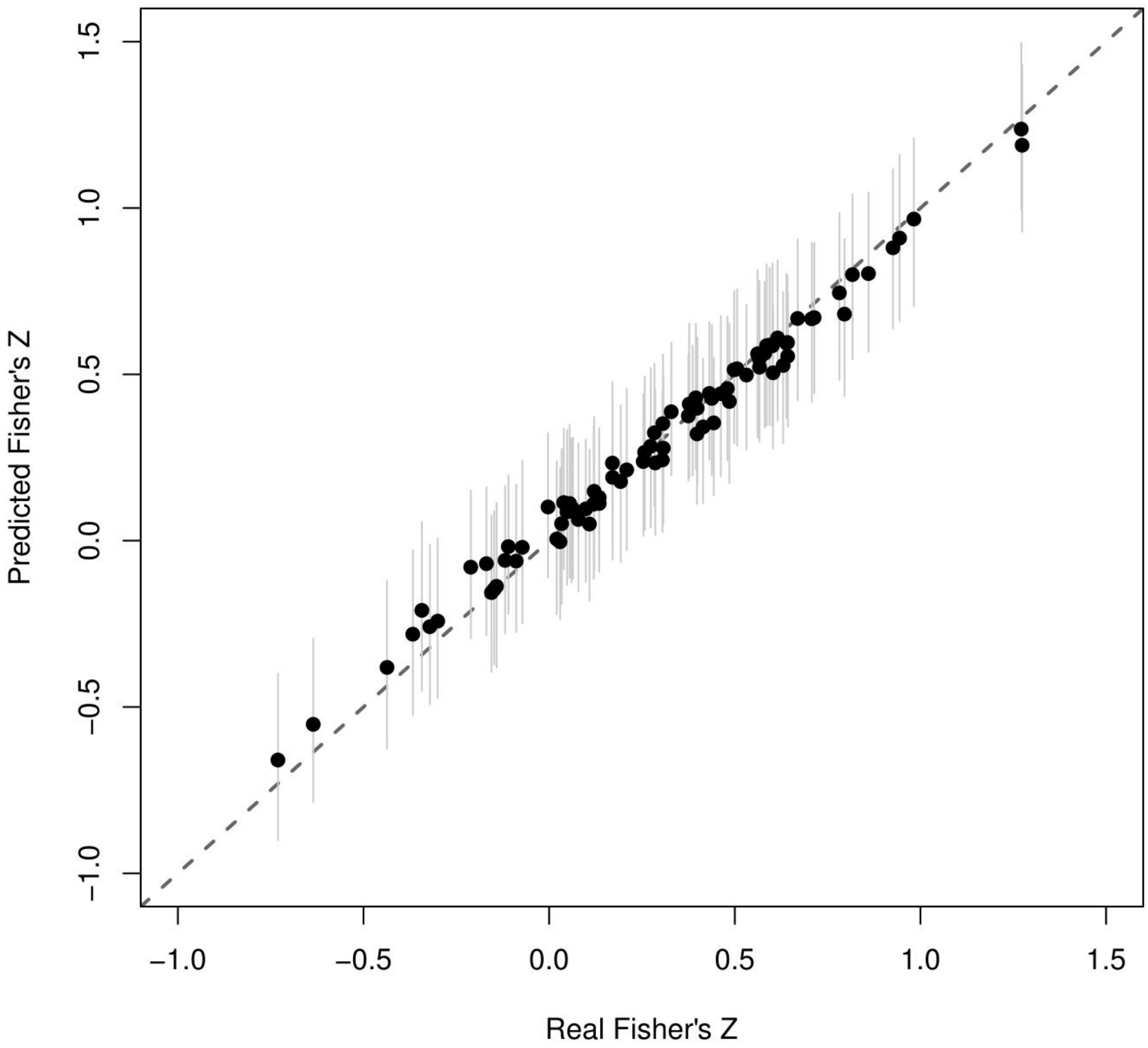

Supplemental Figure S5: Posterior predictive check of continuous syllable repertoire size model. Each black dot compares the actual correlation in Fisher's *Z* between individual song elaboration and reproductive success measured in the field (*x*-axis) to the value predicted for that correlation (*y*-axis) when the model given the repertoire size of the studied species and information about the random effects (i.e. MType, Study, Phylo and Species). The dark grey dashed line shows the line of unity, where the predicted correlation is identical to the real correlation. Light grey solid lines show the 95% confidence interval around each predicted correlation. All dots are very close to or overlapping with the line of unity, showing that the model-predicted correlations were very similar to the real correlations.

Species (Rep) [citation]	nBirds	Fisher's Z	[95% CI]
Anthus spinoletta (5.1) [14]	75	0.135	[−0.096 ; 0.366]
Anthus spinoletta (5.1) [14]	75	0.135	[−0.096 ; 0.366]
Parus caeruleus (6.5) [31]	19	0.034	[−0.456 ; 0.524]
Parus caeruleus (6.5) [31]	19	0.486	[−0.004 ; 0.975]
Plectrophenax nivalis (7.46) [40]	18	−0.321	[−0.827 ; 0.185]
Plectrophenax nivalis (7.46) [40]	23	0.02	[−0.418 ; 0.459]
Parus major (7.9) [33]	32	−0.119	[−0.482 ; 0.245]
Parus major (7.9) [33]	13	0.193	[−0.427 ; 0.813]
Parus major (7.9) [33]	32	−0.089	[−0.453 ; 0.275]
Parus major (7.9) [33]	32	0.079	[−0.285 ; 0.443]
Parus major (7.9) [34]	81	0.286	[0.064 ; 0.508]
Dendroica pensylvanica (10.75) [17]	36	0.109	[−0.232 ; 0.45]
Dendroica pensylvanica (10.75) [17]	36	0.03	[−0.312 ; 0.371]
Cardinalis cardinalis (14) [15]	21	−0.367	[−0.829 ; 0.095]
Wilsonia canadensis (17.43) [48]	38	0.06	[−0.271 ; 0.391]
Wilsonia canadensis (17.43) [48]	47	0.064	[−0.232 ; 0.359]
Emberiza schoeniclus (18.15) [18]	26	−0.635	[−1.044 ; −0.227]
Agelaius phoeniceus (18.5) [13]	49	−0.169	[−0.458 ; 0.12]
Agelaius phoeniceus (18.5) [13]	49	0.415	[0.126 ; 0.704]
Hirundo rustica (20.1) [22]	16	0.171	[−0.373 ; 0.715]
Hirundo rustica (20.1) [20]	27	−0.148	[−0.548 ; 0.252]
Acrocephalus arundinaceus (22.5) [1]	107	0.375	[0.183 ; 0.567]
Acrocephalus arundinaceus (22.5) [1]	107	0.398	[0.206 ; 0.59]
Acrocephalus arundinaceus (22.5) [1]	107	0.387	[0.194 ; 0.579]
Acrocephalus arundinaceus (22.5) [2]	37	0.273	[−0.063 ; 0.609]
Acrocephalus arundinaceus (22.5) [2]	37	0.288	[−0.048 ; 0.624]
Acrocephalus arundinaceus (22.5) [2]	37	0.404	[0.068 ; 0.74]
Acrocephalus arundinaceus (22.5) [3]	35	0.099	[−0.248 ; 0.445]
Acrocephalus arundinaceus (22.5) [3]	62	−0.11	[−0.365 ; 0.146]
Acrocephalus arundinaceus (22.5) [3]	52	0.443	[0.163 ; 0.723]
Acrocephalus arundinaceus (22.5) [3]	47	0.04	[−0.256 ; 0.335]
Acrocephalus arundinaceus (22.5) [3]	39	0.305	[−0.021 ; 0.632]
Acrocephalus arundinaceus (22.5) [3]	47	−0.211	[−0.506 ; 0.085]
Acrocephalus arundinaceus (22.5) [5]	77	0.254	[0.026 ; 0.482]
Phylloscopus fuscatus (25.3) [35]	7	−0.141	[−1.121 ; 0.839]
Ficedula hypoleuca (28.4) [21]	50	0.307	[0.022 ; 0.593]
Ficedula albicollis (31.4) [19]	53	−0.343	[−0.62 ; −0.066]
Ficedula albicollis (31.4) [20]	16	−0.155	[−0.699 ; 0.388]
Phylloscopus trochilus (35.1) [38]	62	−0.003	[−0.258 ; 0.252]
Phylloscopus trochilus (35.1) [38]	25	0.796	[0.378 ; 1.214]
Phylloscopus trochilus (35.1) [38]	19	0.048	[−0.442 ; 0.538]
Phylloscopus trochilus (35.1) [39]	8	0.782	[−0.095 ; 1.659]
Melospiza melodia (38) [24]	72	0.603	[0.367 ; 0.839]
Melospiza melodia (38) [24]	41	0.395	[0.077 ; 0.713]
Melospiza melodia (38) [25]	21	0.121	[−0.341 ; 0.583]
Melospiza melodia (38) [25]	15	−0.072	[−0.638 ; 0.494]
Melospiza melodia (38) [25]	10	0.209	[−0.532 ; 0.95]
Melospiza melodia (38) [26]	16	0.702	[0.158 ; 1.245]
Melospiza melodia (38) [22]	54	0.642	[0.368 ; 0.916]
Melospiza melodia (38) [22]	53	0.48	[0.203 ; 0.757]
Melospiza melodia (38) [22]	54	0.432	[0.157 ; 0.706]
Melospiza melodia (38) [22]	32	0.639	[0.275 ; 1.003]
Melospiza melodia (38) [28,20]	28	0.12	[−0.272 ; 0.512]
Melospiza melodia (38) [29]	64	0.643	[0.392 ; 0.894]
Melospiza melodia (38) [29]	56	0.307	[0.037 ; 0.576]
Melospiza melodia (38) [29]	64	0.329	[0.078 ; 0.58]
Carpodacus mexicanus (41.3) [16]	13	0.462	[−0.157 ; 1.082]
Carpodacus mexicanus (41.3) [16]	13	0.274	[−0.346 ; 0.894]
Sturnus vulgaris (41.95) [45]	15	0.708	[0.142 ; 1.273]
Sturnus vulgaris (41.95) [45]	7	−0.437	[−1.417 ; 0.543]
Sturnus vulgaris (41.95) [46]	6	1.272	[0.14 ; 2.403]
Sturnus vulgaris (41.95) [46]	5	0.982	[−0.404 ; 2.368]
Sturnus vulgaris (41.95) [46]	10	0.944	[0.203 ; 1.685]
Sturnus vulgaris (41.95) [46]	10	0.817	[0.076 ; 1.558]
Acrocephalus bistrigiceps (55) [6]	18	0.257	[−0.249 ; 0.764]
Acrocephalus schoenobaenus (55) [9]	26	0.566	[0.158 ; 0.975]
Acrocephalus schoenobaenus (55) [9]	31	0.399	[0.029 ; 0.769]
Acrocephalus schoenobaenus (55) [9]	23	0.438	[0 ; 0.877]
Acrocephalus schoenobaenus (55) [10]	10	1.274	[0.533 ; 2.015]
Acrocephalus schoenobaenus (55) [11]	19	−0.3	[−0.79 ; 0.19]
Acrocephalus schoenobaenus (55) [12]	10	−0.73	[−1.471 ; 0.011]
Sturnella neglecta (86) [43]	9	0.601	[−0.199 ; 1.401]
Sturnella neglecta (86) [43]	14	0.566	[−0.025 ; 1.157]
Sturnella neglecta (86) [43]	9	0.615	[−0.185 ; 1.416]
Sturnella neglecta (86) [43]	14	0.594	[0.003 ; 1.185]
Sturnella neglecta (86) [43]	9	0.586	[−0.214 ; 1.386]
Sturnella neglecta (86) [43]	14	0.506	[−0.085 ; 1.097]
Sturnella neglecta (86) [44]	24	0.377	[−0.05 ; 0.805]
Mimus polyglottos (216) [30]	18	0.3	[−0.207 ; 0.806]
Acrocephalus palustris (241) [7]	23	0.17	[−0.268 ; 0.609]
Acrocephalus palustris (241) [7]	23	0.498	[0.06 ; 0.937]
Acrocephalus palustris (241) [8]	31	0.581	[0.21 ; 0.951]
Acrocephalus palustris (241) [8]	31	0.714	[0.344 ; 1.084]
Acrocephalus palustris (241) [8]	31	0.055	[−0.315 ; 0.425]
Sylvia communis (367.5) [47]	12	0.67	[0.016 ; 1.323]
Luscinia megarhynchos (1160) [23]	8	0.561	[−0.315 ; 1.438]

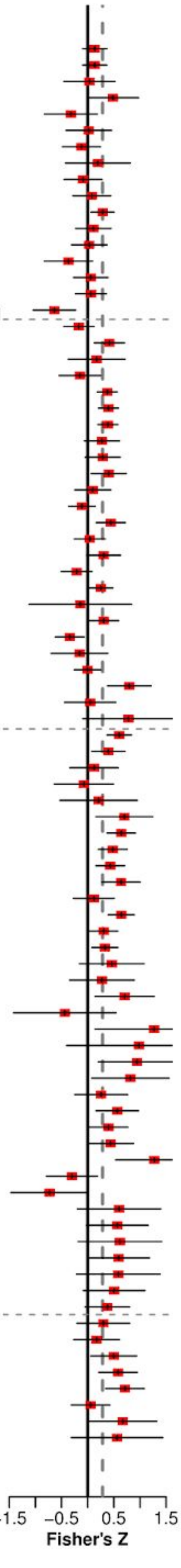

Supplemental Figure S6: Forest plot of the syllable repertoire dataset with territory-controlled measurements. This forest plot shows the individual studies and species studied, the number of birds used to generate a measurement, the Fisher's *Z* form of the estimate, and its 95% confidence intervals. Ticks in the boxes mark the Fisher's *Z* and black horizontal lines show the confidence interval. Grey, dashed vertical line shows the population mean. The grey, dashed horizontal lines show the thresholds used for subsequent analysis. When the same study is listed in more than one row on the plot, multiple different metrics of reproductive success were obtained from that study. Results of the Bayesian meta-analysis and BEST analysis are in Supplemental Tables 26 and 27 respectively.

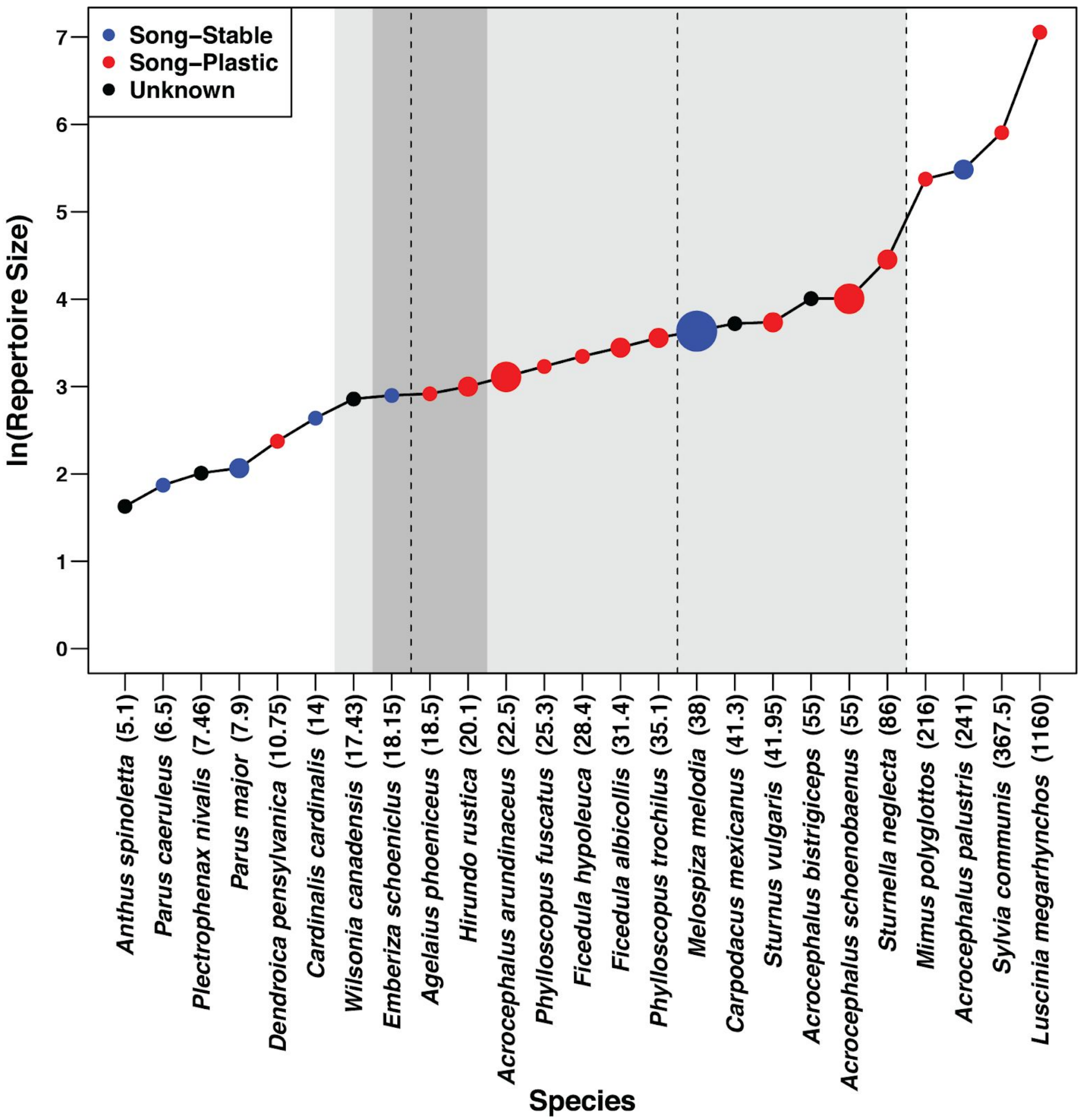

Supplemental Figure S7: Distribution of species average repertoire sizes. Species plotted in order of increasing average syllable repertoire size. Species average syllable repertoire size (without the log transformation) is included next to species name in parentheses. Red circles denote song-plastic species, and blue circles denote song-stable species. Black circles denote species for which no song stability information was available. Circle size increases as more studies were included for a single species. We tested each species average syllable repertoire size

between 7.46 and 367.5 as a threshold between smaller and larger species average syllable repertoires in the Bayesian meta-analyses. The darker grey region denotes species average syllable repertoire thresholds for which the meta-analytic mean for the smaller group was less than 0.05, while the lighter grey region denotes species average syllable repertoire thresholds for which p_{MCMC}<0.025 for the larger species average syllable repertoire group. Dashed lines show the thresholds used for all subsequent species average syllable repertoire size analyses (greater than or equal to 18.5, 38, and 216).

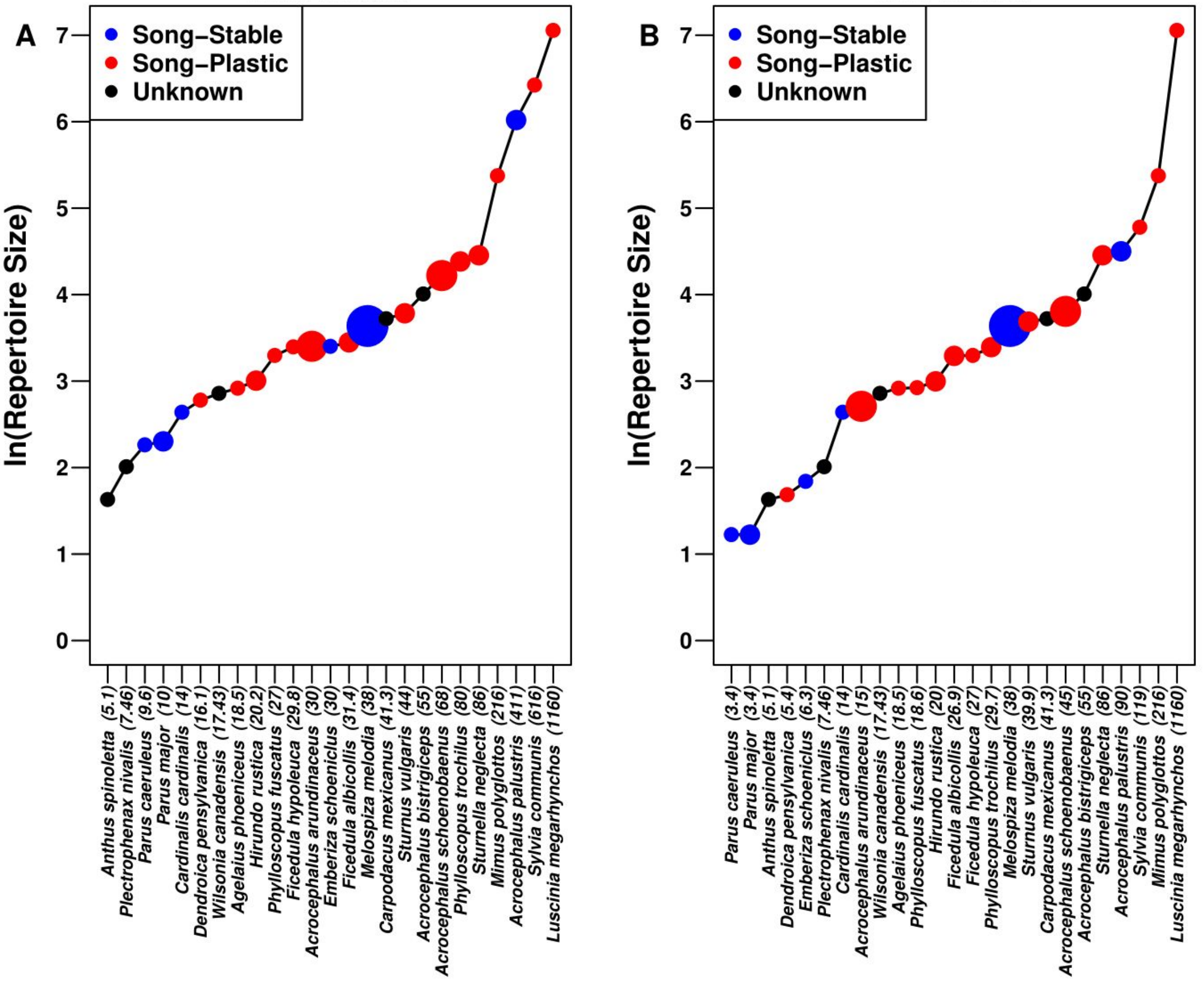

Supplemental Figure S8: Species plotted in order of increasing syllable repertoire size. Repertoire size (without the log transformation) is included next to species name in parentheses. Red circles denote song-plastic species, and blue circles denote song-stable species. Black circles denote species for which no song stability information was available. Circle size increases as more studies were included for a single species. (A) shows the order when the maximum estimate values in the literature were used to rank species, while (B) shows the order when the minimum values in the literature were used to rank species.

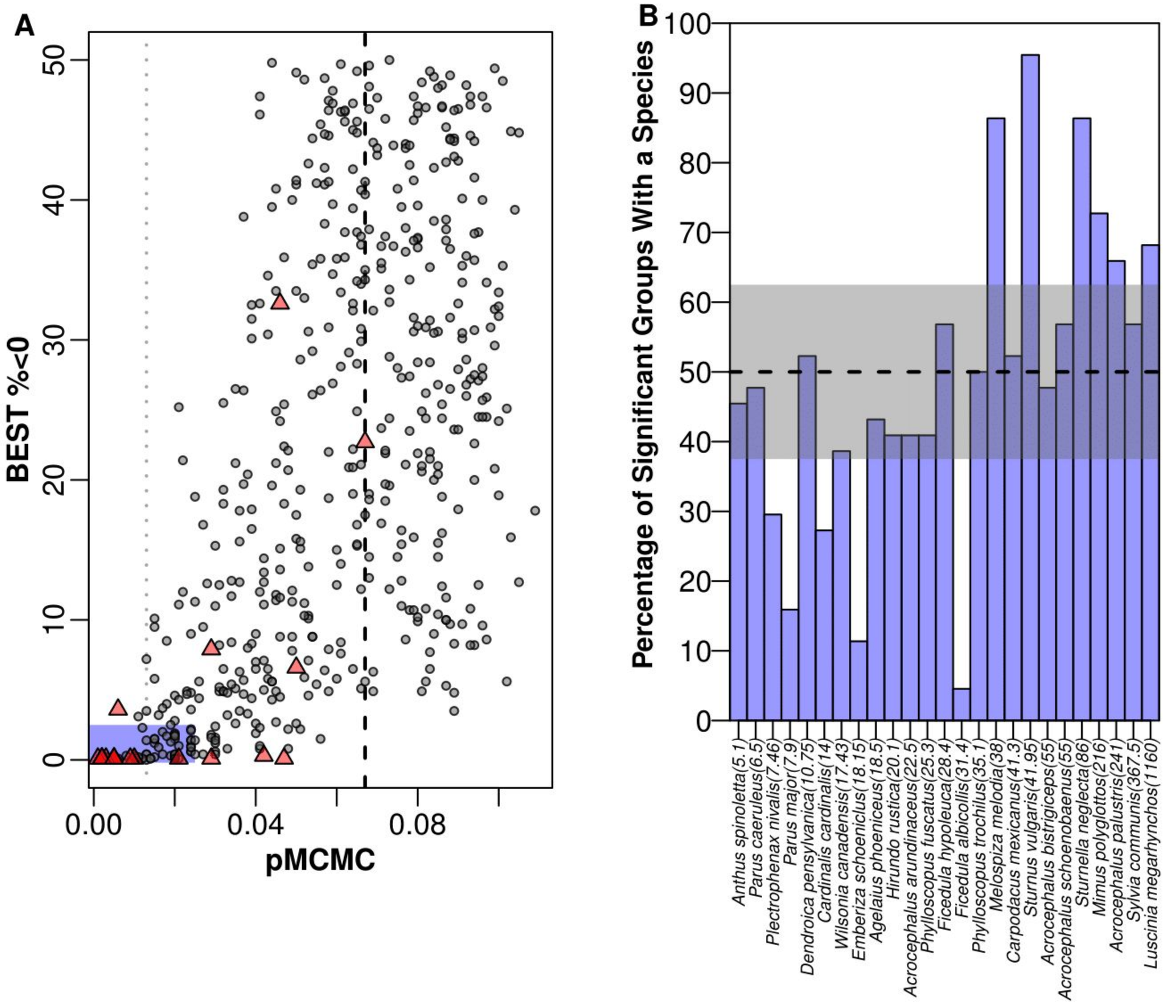

Supplemental Figure S9: Distribution of models when the two constituent groups are formed randomly using the syllable repertoire dataset. (A) Black dashed line denotes the meta-analytic mean for the full population in the syllable repertoire dataset. Each grey dot represents the group from each randomized model with the lowest p_{MCMC}. Each red triangle represents the p_{MCMC} for the larger syllable repertoire group at each of the thresholds tested in the syllable repertoire dataset for the main analysis. The light blue rectangle shows the region where a model must fall to have a group with significant evidence for a correlation between song elaboration and reproductive success (p_{MCMC}<0.025) that was also significantly different from the other group in that same model ($BEST_{\%<0}$<2.5%). In total, 4.4% of all randomized groups (8.8% of the 2-group models) were significant based on this criterion. In contrast, 14 out of 21 models from the real

data were significant (thresholds 18.15 to 216 syllables); the middle range of thresholds was significant while the largest and smallest thresholds were not. The grey dashed line indicates the p_{MCMC} threshold at which 2.5% percent of the randomized data would be considered significant (p_{MCMC}<=0.013). At this significance threshold, 13 out of 21 models from the real data were significant (thresholds 18.5 to 216 syllables). (B) Histogram of species in the group with a significant p_{MCMC} from the randomized models present in the blue region of (A) (44 models). Species are ordered from smallest to largest syllable repertoire size, with the literature reported value in parentheses after the species name. The black dashed line shows the percent chance a species had to be in randomized group 1 or group 2, and grey shaded rectangle shows where the data would be expected to fall due to chance 95% of the time. Significant groups contained several species with repertoire sizes less than 25 syllables less often than would be expected by random chance (*Parus caeruleus, Plectrophenax nivalis, Cardinalis cardinalis, and Emberiza schoeniclus*) and were enriched above chance levels in most species with repertoires greater than 55 syllables (*Sturnella neglecta, Mimus polyglottos, Acrocephalus palustris, Luscinia megarhynchos*).

Species (Rep) [citation]	nBirds	Fisher's Z	[95% CI]
Acrocephalus palustris (241) [7]	23	0.17	[−0.268 ; 0.609]
Acrocephalus palustris (241) [7]	23	0.498	[0.06 ; 0.937]
Acrocephalus palustris (241) [8]	31	0.581	[0.21 ; 0.951]
Acrocephalus palustris (241) [8]	31	0.714	[0.344 ; 1.084]
Acrocephalus palustris (241) [8]	31	0.055	[−0.315 ; 0.425]
Cardinalis cardinalis (14) [15]	21	−0.367	[−0.829 ; 0.095]
Emberiza schoeniclus (18.15) [18]	26	−0.635	[−1.044 ; −0.227]
Melospiza melodia (38) [24]	72	0.603	[0.367 ; 0.839]
Melospiza melodia (38) [24]	41	0.395	[0.077 ; 0.713]
Melospiza melodia (38) [25]	21	0.121	[−0.341 ; 0.583]
Melospiza melodia (38) [25]	15	−0.072	[−0.638 ; 0.494]
Melospiza melodia (38) [25]	10	0.209	[−0.532 ; 0.95]
Melospiza melodia (38) [26]	16	0.702	[0.158 ; 1.245]
Melospiza melodia (38) [22]	54	0.642	[0.368 ; 0.916]
Melospiza melodia (38) [22]	53	0.48	[0.203 ; 0.757]
Melospiza melodia (38) [22]	54	0.432	[0.157 ; 0.706]
Melospiza melodia (38) [22]	32	0.639	[0.275 ; 1.003]
Melospiza melodia (38) [28,20]	28	0.12	[−0.272 ; 0.512]
Melospiza melodia (38) [29]	64	0.643	[0.392 ; 0.894]
Melospiza melodia (38) [29]	56	0.307	[0.037 ; 0.576]
Melospiza melodia (38) [29]	64	0.329	[0.078 ; 0.58]
Parus caeruleus (6.5) [31]	19	0.034	[−0.456 ; 0.524]
Parus caeruleus (6.5) [31]	19	0.486	[−0.004 ; 0.975]
Parus major (7.9) [33]	32	−0.119	[−0.482 ; 0.245]
Parus major (7.9) [33]	13	0.193	[−0.427 ; 0.813]
Parus major (7.9) [33]	32	−0.089	[−0.453 ; 0.275]
Parus major (7.9) [33]	32	0.079	[−0.285 ; 0.443]
Parus major (7.9) [34]	81	0.286	[0.064 ; 0.508]
Acrocephalus arundinaceus (22.5) [1]	107	0.375	[0.183 ; 0.567]
Acrocephalus arundinaceus (22.5) [1]	107	0.398	[0.206 ; 0.59]
Acrocephalus arundinaceus (22.5) [1]	107	0.387	[0.194 ; 0.579]
Acrocephalus arundinaceus (22.5) [2]	37	0.273	[−0.063 ; 0.609]
Acrocephalus arundinaceus (22.5) [2]	37	0.288	[−0.048 ; 0.624]
Acrocephalus arundinaceus (22.5) [2]	37	0.404	[0.068 ; 0.74]
Acrocephalus arundinaceus (22.5) [3]	35	0.099	[−0.248 ; 0.445]
Acrocephalus arundinaceus (22.5) [3]	62	−0.11	[−0.365 ; 0.146]
Acrocephalus arundinaceus (22.5) [3]	52	0.443	[0.163 ; 0.723]
Acrocephalus arundinaceus (22.5) [3]	47	0.04	[−0.256 ; 0.335]
Acrocephalus arundinaceus (22.5) [3]	39	0.305	[−0.021 ; 0.632]
Acrocephalus arundinaceus (22.5) [3]	47	−0.211	[−0.506 ; 0.085]
Acrocephalus arundinaceus (22.5) [5]	77	0.254	[0.026 ; 0.482]
Acrocephalus schoenobaenus (55) [9]	26	0.566	[0.158 ; 0.975]
Acrocephalus schoenobaenus (55) [9]	31	0.399	[0.029 ; 0.769]
Acrocephalus schoenobaenus (55) [9]	23	0.438	[0 ; 0.877]
Acrocephalus schoenobaenus (55) [10]	10	1.274	[0.533 ; 2.015]
Acrocephalus schoenobaenus (55) [11]	19	−0.3	[−0.79 ; 0.19]
Acrocephalus schoenobaenus (55) [12]	10	−0.73	[−1.471 ; 0.011]
Agelaius phoeniceus (18.5) [13]	49	−0.169	[−0.458 ; 0.12]
Agelaius phoeniceus (18.5) [13]	49	0.415	[0.126 ; 0.704]
Dendroica pensylvanica (10.75) [17]	36	0.109	[−0.232 ; 0.45]
Dendroica pensylvanica (10.75) [17]	36	0.03	[−0.312 ; 0.371]
Ficedula albicollis (31.4) [19]	53	−0.343	[−0.62 ; −0.066]
Ficedula albicollis (31.4) [20]	16	−0.155	[−0.699 ; 0.388]
Ficedula hypoleuca (28.4) [21]	50	0.307	[0.022 ; 0.593]
Hirundo rustica (20.1) [22]	16	0.171	[−0.373 ; 0.715]
Hirundo rustica (20.1) [20]	27	−0.148	[−0.548 ; 0.252]
Luscinia megarhynchos (1160) [23]	8	0.561	[−0.315 ; 1.438]
Mimus polyglottos (216) [30]	18	0.3	[−0.207 ; 0.806]
Phylloscopus fuscatus (25.3) [35]	7	−0.141	[−1.121 ; 0.839]
Phylloscopus trochilus (35.1) [38]	62	−0.003	[−0.258 ; 0.252]
Phylloscopus trochilus (35.1) [38]	25	0.796	[0.378 ; 1.214]
Phylloscopus trochilus (35.1) [38]	19	0.048	[−0.442 ; 0.538]
Phylloscopus trochilus (35.1) [39]	8	0.782	[−0.095 ; 1.659]
Sturnella neglecta (86) [43]	9	0.601	[−0.199 ; 1.401]
Sturnella neglecta (86) [43]	14	0.566	[−0.025 ; 1.157]
Sturnella neglecta (86) [43]	9	0.615	[−0.185 ; 1.416]
Sturnella neglecta (86) [43]	14	0.594	[0.003 ; 1.185]
Sturnella neglecta (86) [43]	9	0.586	[−0.214 ; 1.386]
Sturnella neglecta (86) [43]	14	0.506	[−0.085 ; 1.097]
Sturnella neglecta (86) [44]	24	0.377	[−0.05 ; 0.805]
Sturnus vulgaris (41.95) [45]	15	0.708	[0.142 ; 1.273]
Sturnus vulgaris (41.95) [45]	7	−0.437	[−1.417 ; 0.543]
Sturnus vulgaris (41.95) [46]	6	1.272	[0.14 ; 2.403]
Sturnus vulgaris (41.95) [46]	5	0.982	[−0.404 ; 2.368]
Sturnus vulgaris (41.95) [46]	10	0.944	[0.203 ; 1.685]
Sturnus vulgaris (41.95) [46]	10	0.817	[0.076 ; 1.558]
Sylvia communis (367.5) [47]	12	0.67	[0.016 ; 1.323]

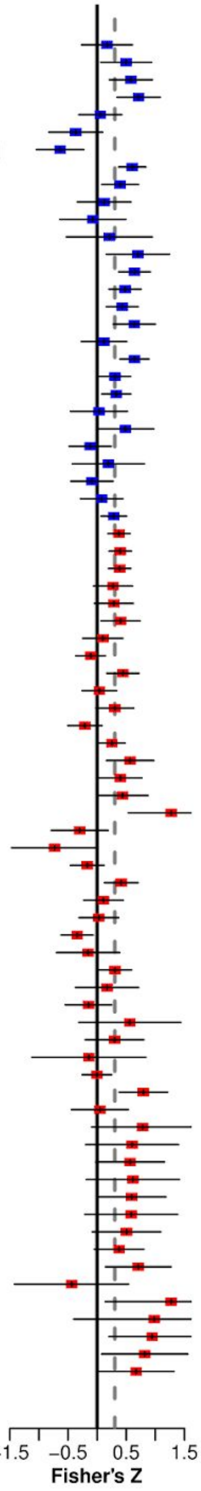

Supplemental Figure S10: Forest plot of the song stability dataset with territory-controlled measurements. This forest plot shows the individual studies and species studied, the number of birds used to generate a measurement, the Fisher's *Z* form of the estimate, and its 95% confidence intervals. Blue boxes mark measurements in the song-stable group, while red boxes mark measurements in the song-plastic group. Ticks in the boxes mark the Fisher's *Z* and black horizontal lines show the confidence interval. The grey, dashed vertical line shows the population mean. When the same study is listed in more than one row on the plot, multiple different metrics of reproductive success were obtained from that study. Results of the Bayesian meta-analysis and BEST analysis are in Supplemental Tables 45 and 46 respectively for the discrete analysis, and meta-analysis results for the continuous analysis are in Supplemental Table S36.

Tables Related to Methods

Supplemental Table S1: All measurements that we pulled from the literature. This large table is available as a separate Excel spreadsheet. Yellow colored cells were included in the main analysis. Orange colored cells were subsets of the data accounted for in the yellow cells. If a study measured the same population across different years, we either used the measurement that pooled all years or used the year where the most males were evaluated. Thus, orange cells were omitted. Purple cells were territory-controlled measures with a non-territory-controlled counterpart. These were included only in the supplemental territory datasets in place of the non-territory-controlled counterpart. Blue cells mark recruits and older offspring in the fledgling data. The red cell marks an instance where both day song and dawn song were analyzed. Both measurements were included. The green cell marks an instance where the possession of rare song types was correlated with individual reproductive success, a metric that we deemed did not relate to elaboration *per se* and omitted. Dark grey cells were omitted because no statistic was provided, or the provided statistic could not be converted into an r value. There are three notes about specific studies at the bottom of the table, which are related back to the row they refer to via one or more asterisks present at the end of the row. There is also a note about the ambiguous "repertoire size" notation in column 3.

Supplemental Table S2: Syllable repertoire estimates for House Finch (*Carpodacus mexicanus*). (top table) XC ID is the xeno-canto ID number for each recording. The first half of the table shows when a percentage of the discovered unique syllables was found in regards to the number of bouts analyzed, while the second half shows the percentage of unique syllables discovered over time in seconds. Analyzed recordings were all at least two minutes long. In most cases, the half of the syllable repertoire was revealed in the first few bouts with the majority revealed before bout 12. These estimates were similar to those in Tracy and Baker 1999 (bottom table), so we felt confident that they had defined "syllable types" per bird similarly to how we defined "unique syllables" per bird, and we combined the two data sets to get a final mean of 41.3 unique syllables per bird.

XC ID	bouts analyzed	50% rep	90% rep	complete rep	Length (s)	50% rep	90% rep	complete rep	syl rep
XC320727	17	1	7	11	124	4	48	73	29
XC268463	27	2	14	20	260	10	82	178	31
XC268458	24	2	6	14	190	9	36	95	33
XC353018	40	8	23	32	136	30	57	102	34
XC219331	20	3	11	12	167	15	69	78	35
XC179232	25	1	5	6	197	5	34	44	37
XC268457	18	2	11	11	139	9	82	84	44
XC268465	45	3	6	10	222	13	23	41	44
XC73494	19	3	10	11	178	12	71	74	49
XC268464	49	3	11	25	300	10	45	89	52

Average: 38.8 Unique syllables
Note: bout length varies widely within and between individuals

Syllable Repertoires from Tracy and Baker 1999 [77]:

31	30	31	72	64
45	38	41	34	35
58	43	40		

Average: 43.23 Unique syllables
Combined Average: 41.3 Unique syllables

Supplemental Table S3: Syllable repertoire estimates for Canada Warbler (*Wilsonia canadensis*). XC ID is the xeno-canto ID number for each recording. The first half of the table shows when a percentage of the discovered unique syllables was found in regards to the number of bouts analyzed, while the second half shows the percentage of unique syllables discovered over time in seconds. Analyzed recordings were all at least two minutes long. In most cases, half of the syllable repertoire was revealed in the first few bouts. The majority of the syllable repertoire was usually revealed by the fifth bout and well before the end of the recordings. The final mean used in this study was 17.43 unique syllables per bird.

XC ID	bouts analyzed	50% rep	90% rep	complete rep	Length (s)	50% rep	90% rep	complete rep	syl rep
XC51468	13	1	1	1	168	5	5	5	9
XC189300	27	1	3	3	324	2	27	27	11
XC179679	29	3	9	13	225	11	44	66	18
XC371402	22	1	5	10	174	3	34	72	14
XC294137	14	2	2	10	155	18	18	79	19
XC189302	39	2	4	9	239	8	19	36	25
XC370937	38	1	3	6	260	4	13	41	26

Average: 17.43 Unique syllables

Supplemental Table S4: Species present in each dataset.

Full	Repertoire	Stability	No Offspring	No Offspring or EPP
Acrocephalus arundinaceus	Acrocephalus arundinaceus	Acrocephalus arundinaceus	Acrocephalus arundinaceus	Acrocephalus arundinaceus
Acrocephalus bistrigiceps	Acrocephalus bistrigiceps		Acrocephalus bistrigiceps	Acrocephalus bistrigiceps
Acrocephalus palustris	Acrocephalus palustris	Acrocephalus palustris	Acrocephalus palustris	Acrocephalus palustris
Acrocephalus schoenobaenus	Acrocephalus schoenobaenus	Acrocephalus schoenobaenus	Acrocephalus schoenobaenus	Acrocephalus schoenobaenus
Agelaius phoeniceus	Agelaius phoeniceus	Agelaius phoeniceus	Agelaius phoeniceus	Agelaius phoeniceus
Anthus spinoletta	Anthus spinoletta		Anthus spinoletta	Anthus spinoletta
Cardinalis cardinalis	Cardinalis cardinalis	Cardinalis cardinalis		
Carpodacus mexicanus	Carpodacus mexicanus		Carpodacus mexicanus	Carpodacus mexicanus
Dendroica pensylvanica	Dendroica pensylvanica	Dendroica pensylvanica	Dendroica pensylvanica	
Emberiza schoeniclus	Emberiza schoeniclus	Emberiza schoeniclus	Emberiza schoeniclus	
Ficedula albicollis	Ficedula albicollis	Ficedula albicollis	Ficedula albicollis	Ficedula albicollis
Ficedula hypoleuca	Ficedula hypoleuca	Ficedula hypoleuca	Ficedula hypoleuca	Ficedula hypoleuca
Hirundo rustica	Hirundo rustica	Hirundo rustica	Hirundo rustica	Hirundo rustica
Luscinia megarhynchos	Luscinia megarhynchos	Luscinia megarhynchos	Luscinia megarhynchos	
Melospiza melodia	Melospiza melodia	Melospiza melodia	Melospiza melodia	Melospiza melodia
Mimus polyglottos	Mimus polyglottos	Mimus polyglottos	Mimus polyglottos	Mimus polyglottos
Parus caeruleus	Parus caeruleus	Parus caeruleus	Parus caeruleus	Parus caeruleus
Parus major	Parus major	Parus major	Parus major	Parus major
Phylloscopus fuscatus	Phylloscopus fuscatus	Phylloscopus fuscatus	Phylloscopus fuscatus	
Phylloscopus trochiloides				
Phylloscopus trochilus	Phylloscopus trochilus	Phylloscopus trochilus	Phylloscopus trochilus	Phylloscopus trochilus
Plectrophenax nivalis	Plectrophenax nivalis		Plectrophenax nivalis	Plectrophenax nivalis
Saxicola caprata				
Sturnella neglecta	Sturnella neglecta	Sturnella neglecta	Sturnella neglecta	Sturnella neglecta
Sturnus vulgaris	Sturnus vulgaris	Sturnus vulgaris	Sturnus vulgaris	Sturnus vulgaris
Sylvia communis	Sylvia communis	Sylvia communis	Sylvia communis	Sylvia communis
Wilsonia canadensis	Wilsonia canadensis			

Supplemental Table S5: Different metrics of reproductive success have different meta-analytic means. Model tested in the song stability dataset. Females refers to measurements that counted the number of social mates obtained. Fledge refers to measurements of the number of fledglings produced. Clutch refers to measurements of clutch size. Laying refers to measurements of the latency to laying date or latency to hatching date. These two measurements were combined, because there was only one measurement of latency to hatching date. Recruits refers to measurements of the number of recruits a male gained. EPP refers to measurements of extra-pair paternity.

Group	#Species	#Measure	Post Mean	95% CredInt	pMCMC
Females	5	10	0.404	[0.016;0.802]	0.045
Fledge	12	20	0.345	[-0.035;0.717]	0.059
Pairing	14	25	0.243	[-0.142;0.614]	0.147
Clutch	6	8	0.254	[-0.138;0.648]	0.151
Laying	7	11	0.219	[-0.161;0.633]	0.211
Recruits	3	6	0.224	[-0.187;0.627]	0.212
EPP	9	11	-0.182	[-0.596;0.228]	0.316

Variance Tables

Supplemental Table S6: Song stability variance in the song stability dataset. Different sources of variance and non-independence in the data were added to the model as random effects terms alone and in combination with the others. MType encodes the variance due to the metric of reproductive success used to generate each measurement. Study indicates variance accounted for by studies that reported multiple measurements. Phylo accounts for the effects of phylogeny, while Species encompasses all remaining species-related effects. Percent variance was calculated by dividing the mean estimated variance by the total variance in the data. DIC stands for deviance information criterion.

Random	I^2	DIC
Species	10.9%	15.7
MType	43.9%	8.9
Study	51.51%	-6.55
Species	6.38%	10.8
Phylo	13.55%	" "
MType	31.49%	-29.78
Study	23.24%	" "
MType	36.46%	-11.07
Species	3.56%	" "
Phylo	5.75%	" "
Study	32.81%	-23.9
Species	3.32%	" "
Phylo	7.12%	" "
MType	26.11%	-46.02
Species	2.22%	" "
Phylo	4.49%	" "
Study	17.13%	" "

Supplemental Table S7: Continuous repertoire size variance in the song stability dataset. Labels are the same as in **Supplemental Table S6**.

Random	I^2	DIC
Species	59.04%	14.3
MType	33.71%	36.21
Study	63.85%	-7.29
Species	12.56%	10.17
Phylo	18.64%	" "
MType	16.09%	-26.56
Study	48.51%	" "
MType	29.59%	-12.36
Species	7.61%	" "
Phylo	12.7%	" "
Study	35.16%	-24.83
Species	5.29%	" "
Phylo	13.61%	" "
MType	21.73%	-45.37
Species	4.52%	" "
Phylo	11.79%	" "
Study	17.12%	" "

Supplemental Table S8: Discrete repertoire size variance in the song stability dataset. Labels are the same as in **Supplemental Table S6**. Threshold ≥ 7.9.

Random	I^2	DIC
Species	56.56%	16.53
MType	37.01%	35.56
Study	63.61%	-7.63
Species	12.35%	12.02
Phylo	19.98%	" "
MType	15.15%	-27.29
Study	47.23%	" "
MType	24.92%	-9.88
Species	7.52%	" "
Phylo	14.86%	" "
Study	38.7%	-24.64
Species	5.28%	" "
Phylo	8.39%	" "
MType	20.31%	-44.55
Species	3.96%	" "
Phylo	9.67%	" "
Study	22.41%	" "

Supplemental Table S9: Discrete repertoire size variance in the song stability dataset. Labels are the same as in **Supplemental Table S6**. Threshold ≥ 10.75.

Random	I^2	DIC
Species	54.68%	17.57
MType	44.89%	27.26
Study	63.18%	-6.4
Species	9.47%	12.1
Phylo	29.3%	" "
MType	21.89%	-25.52
Study	40.7%	" "
MType	31.11%	-9.01
Species	4.81%	" "
Phylo	16.2%	" "
Study	38.51%	-24.26
Species	5.03%	" "
Phylo	10.55%	" "
MType	22.21%	-44.18
Species	2.89%	" "
Phylo	10.57%	" "
Study	19.97%	" "

Supplemental Table S10: Discrete repertoire size variance in the song stability dataset. Labels are the same as in **Supplemental Table S6**. Threshold ≥ 14.

Random	I^2	DIC
Species	41.33%	18.73
MType	31.76%	31.99
Study	61.79%	-5.67
Species	9.89%	13.18
Phylo	22.53%	" "
MType	14.26%	-25.71
Study	47.34%	" "
MType	26.31%	-9.04
Species	6.58%	" "
Phylo	15.67%	" "
Study	42.27%	-23.73
Species	4.17%	" "
Phylo	11.01%	" "
MType	23.96%	-43.95
Species	3.23%	" "
Phylo	7.77%	" "
Study	21.39%	" "

Supplemental Table S11: Discrete repertoire size variance in the song stability dataset. Labels are the same as in **Supplemental Table S6**. Threshold ≥ 18.15.

Random	I^2	DIC
Species	19.52%	19.54
MType	33.57%	28.03
Study	58.21%	-5.09
Species	9.25%	13.74
Phylo	19.47%	" "
MType	16.83%	-26.24
Study	44.02%	" "
MType	27.29%	-7.93
Species	7.54%	" "
Phylo	10.64%	" "
Study	35.67%	-23.11
Species	4.32%	" "
Phylo	12.34%	" "
MType	22.54%	-43.78
Species	2.52%	" "
Phylo	8.39%	" "
Study	23.4%	" "

Supplemental Table S12: Discrete repertoire size variance in the song stability dataset. Labels are the same as in **Supplemental Table S6**. Threshold ≥ 18.5.

Random	I^2	DIC
Species	11.12%	17.54
MType	29.87%	23.11
Study	53.18%	-6.13
Species	5.43%	11.32
Phylo	16.05%	" "
MType	15.43%	-26.07
Study	40.69%	" "
MType	22.82%	-7.69
Species	5.83%	" "
Phylo	11.05%	" "
Study	32.95%	-23.91
Species	3.3%	" "
Phylo	9.7%	" "
MType	16.15%	-43.17
Species	2.68%	" "
Phylo	5.91%	" "
Study	25.55%	" "

Supplemental Table S13: Discrete repertoire size variance in the song stability dataset. Labels are the same as in **Supplemental Table S6**. Threshold ≥ 20.1.

Random	I^2	DIC
Species	9.83%	18.15
MType	32.17%	20.19
Study	52.65%	-5.45
Species	6.34%	12.08
Phylo	17.04%	" "
MType	19.31%	-26.64
Study	36.83%	" "
MType	24.52%	-7.89
Species	4.71%	" "
Phylo	9.25%	" "
Study	36.09%	-23.2
Species	2.3%	" "
Phylo	8.07%	" "
MType	19.28%	-43.41
Species	2.77%	" "
Phylo	5.78%	" "
Study	21.03%	" "

Supplemental Table S14: Discrete repertoire size variance in the song stability dataset. Labels are the same as in **Supplemental Table S6**. Threshold $\geq$ 22.5.

Random	I^2	DIC
Species	8.24%	17.28
MType	26.64%	18.67
Study	48.03%	-6.1
Species	5.11%	11.56
Phylo	13.4%	“ ”
MType	17.9%	-26.24
Study	34.84%	“ ”
MType	23.35%	-6.81
Species	4.3%	“ ”
Phylo	10.31%	“ ”
Study	37.02%	-23.23
Species	2.13%	“ ”
Phylo	5.84%	“ ”
MType	15.98%	-42.86
Species	2.33%	“ ”
Phylo	5.55%	“ ”
Study	22.93%	“ ”

Supplemental Table S15: Discrete repertoire size variance in the song stability dataset. Labels are the same as in **Supplemental Table S6**. Threshold $\geq$ 25.3.

Random	I^2	DIC
Species	13.67%	16.84
MType	35.73%	13.62
Study	54.87%	-5.55
Species	8.02%	11.43
Phylo	15.55%	“ ”
MType	29.29%	-28.37
Study	27.11%	“ ”
MType	29.74%	-8.23
Species	3.17%	“ ”
Phylo	8.86%	“ ”
Study	28.46%	-22.9
Species	4.53%	“ ”
Phylo	10.07%	“ ”
MType	25.21%	-44.92
Species	2.93%	“ ”
Phylo	5%	“ ”
Study	19.16%	“ ”

Supplemental Table S16: Discrete repertoire size variance in the song stability dataset. Labels are the same as in **Supplemental Table S6**. Threshold $\geq$ 28.4.

Random	I^2	DIC
Species	14.45%	16.55
MType	35.13%	13.67
Study	55.36%	-5.67
Species	8.82%	11.24
Phylo	12.36%	“ ”
MType	28.55%	-28.02
Study	28.16%	“ ”
MType	33.1%	-7.81
Species	4.19%	“ ”
Phylo	6.16%	“ ”
Study	29.67%	-22.9
Species	3.76%	“ ”
Phylo	8.2%	“ ”
MType	24.48%	-44.38
Species	2.31%	“ ”
Phylo	5.48%	“ ”
Study	20.05%	“ ”

Supplemental Table S17: Discrete repertoire size variance in the song stability dataset. Labels are the same as in **Supplemental Table S6**. Threshold $\geq$ 31.4.

Random	I^2	DIC
Species	11.77%	17.84
MType	37.78%	16.05
Study	56.46%	-5.04
Species	10.38%	14.15
Phylo	10.83%	“ ”
MType	23.29%	-26.62
Study	30.68%	“ ”
MType	33.48%	-4.18
Species	4.98%	“ ”
Phylo	6.48%	“ ”
Study	36.61%	-22.07
Species	4.52%	“ ”
Phylo	8.71%	“ ”
MType	21.37%	-42.53
Species	2.29%	“ ”
Phylo	2.87%	“ ”
Study	23.54%	“ ”

Supplemental Table S18: Discrete repertoire size variance in the song stability dataset. Labels are the same as in **Supplemental Table S6**. Threshold $\geq$ 35.1.

Random	I^2	DIC
Species	7.01%	12.09
MType	35.99%	2.48
Study	45.1%	-7.37
Species	5.56%	8.51
Phylo	6.36%	" "
MType	26.36%	-32.08
Study	20.84%	" "
MType	31.24%	-10.85
Species	2.8%	" "
Phylo	4.84%	" "
Study	26.51%	-23.06
Species	3.03%	" "
Phylo	5.24%	" "
MType	22.38%	-45.6
Species	1.91%	" "
Phylo	3.97%	" "
Study	18.36%	" "

Supplemental Table S19: Discrete repertoire size variance in the song stability dataset. Labels are the same as in **Supplemental Table S6**. Threshold $\geq$ 38.

Random	I^2	DIC
Species	7.13%	9.78
MType	42.36%	-2.42
Study	40.08%	-7.54
Species	6.09%	6.37
Phylo	5.76%	" "
MType	32.87%	-33.21
Study	5.8%	" "
MType	29.05%	-15.85
Species	2.31%	" "
Phylo	4.66%	" "
Study	20.48%	-24.1
Species	3.84%	" "
Phylo	4.31%	" "
MType	29.1%	-47.5
Species	2.36%	" "
Phylo	3.01%	" "
Study	5.04%	" "

Supplemental Table S20: Discrete repertoire size variance in the song stability dataset. Labels are the same as in **Supplemental Table S6**. Threshold $\geq$ 41.95.

Random	I^2	DIC
Species	40.38%	9.94
MType	36.37%	21.17
Study	53.67%	-6.26
Species	34.83%	7.21
Phylo	8.06%	“ ”
MType	23.91%	-28.15
Study	34.73%	“ ”
MType	30.27%	-15.32
Species	5.6%	“ ”
Phylo	6.06%	“ ”
Study	27.48%	-24.28
Species	6.03%	“ ”
Phylo	8.75%	“ ”
MType	26.68%	-46.2
Species	4.65%	“ ”
Phylo	3.74%	“ ”
Study	11.62%	“ ”

Supplemental Table S21: Discrete repertoire size variance in the song stability dataset. Labels are the same as in **Supplemental Table S6**. Threshold $\geq$ 55.

Random	I^2	DIC
Species	49.1%	11.38
MType	35.94%	23.8
Study	59.85%	-6.89
Species	12.87%	8.17
Phylo	13.84%	“ ”
MType	21.78%	-28.49
Study	37.09%	“ ”
MType	35.78%	-15.01
Species	5.76%	“ ”
Phylo	9.42%	“ ”
Study	30.35%	-24.7
Species	6.5%	“ ”
Phylo	10.01%	“ ”
MType	26.05%	-46.5
Species	4.59%	“ ”
Phylo	8.44%	“ ”
Study	11.65%	“ ”

Supplemental Table S22: Discrete repertoire size variance in the song stability dataset. Labels are the same as in **Supplemental Table S6**. Threshold ≥ 86.

Random	I^2	DIC
Species	45.82%	12.59
MType	39.58%	26.27
Study	58.95%	-8.22
Species	10.5%	9.08
Phylo	14.6%	“ ”
MType	19.69%	-27.51
Study	40.33%	“ ”
MType	29.49%	-13.28
Species	6.83%	“ ”
Phylo	9.24%	“ ”
Study	32.88%	-25.63
Species	5.53%	“ ”
Phylo	10.61%	“ ”
MType	22.52%	-45.68
Species	4%	“ ”
Phylo	7.97%	“ ”
Study	16.6%	“ ”

Supplemental Table S23: Discrete repertoire size variance in the song stability dataset. Labels are the same as in **Supplemental Table S6**. Threshold ≥ 216.

Random	I^2	DIC
Species	53.87%	12.33
MType	38.28%	27.2
Study	61.66%	-9.37
Species	11.74%	9.2
Phylo	17.8%	“ ”
MType	19.14%	-31.37
Study	44.07%	“ ”
MType	31.82%	-14.92
Species	7.22%	“ ”
Phylo	8.43%	“ ”
Study	36.72%	-26.2
Species	4.48%	“ ”
Phylo	10.2%	“ ”
MType	28.73%	-48.5
Species	4.01%	“ ”
Phylo	5.7%	“ ”
Study	17.9%	“ ”

Supplemental Table S24: Discrete repertoire size variance in the song stability dataset. Labels are the same as in **Supplemental Table S6**. Threshold $\geq$ 241.

Random	I^2	DIC
Species	54.47%	14.92
MType	34.98%	30.66
Study	63.25%	-8.2
Species	12.11%	10.82
Phylo	20.79%	" "
MType	21.06%	-28.79
Study	42.73%	" "
MType	32.16%	-12.07
Species	6.45%	" "
Phylo	11.25%	" "
Study	40.31%	-25.12
Species	5.31%	" "
Phylo	8.23%	" "
MType	27.89%	-46.21
Species	4.16%	" "
Phylo	8.16%	" "
Study	16.82%	" "

Supplemental Table S25: Discrete repertoire size variance in the song stability dataset. Labels are the same as in **Supplemental Table S6**. Threshold $\geq$ 367.5.

Random	I^2	DIC
Species	50.27%	16.13
MType	41.78%	32.87
Study	62.75%	-7.48
Species	12.77%	11.81
Phylo	17.95%	" "
MType	18.52%	-28.75
Study	44.77%	" "
MType	32.5%	-11.03
Species	5.29%	" "
Phylo	13.69%	" "
Study	39.65%	-24.64
Species	4.5%	" "
Phylo	11.69%	" "
MType	24.82%	-46.27
Species	3.44%	" "
Phylo	8.83%	" "
Study	21.95%	" "

Tables Regarding the Full Population and Phylogeny

Supplemental Table S26: Population meta-analysis performed in all three datasets with territory-controlled measurements.

Group	#Species	#Measure	Post Mean	95% CredInt	pMCMC
Full Dataset	27	91	0.188	[-0.154;0.53]	0.197
Repertoire Dataset	25	86	0.214	[-0.041;0.458]	0.082
Song Stability dataset	20	77	0.24	[-0.007;0.476]	0.05

Supplemental Table S27: Using multiple predictive trees in place of a consensus tree did not significantly change any results. For each model below, 100 trees were randomly selected from the sample of 1000 used to generate the consensus tree. Each tree was run twice, so 200 MCMC chains were generated for each model. Reported below are values calculated using the quantile method. We obtained similar results using the hdr method, but it threw warnings because the hrd credibility intervals could not be calculated for the residual variance. p_{MCMC} not reported, because mulTree.summary does not provide this value.

Model Group	Post Mean	95% CredInt
Population	0.199	[-0.004;0.402]
Stable	0.147	[-0.206;0.465]
Plastic	0.317	[0.062;0.599]
Intercept	-0.355	[-0.751;0.018]
Slope	0.174	[0.079;0.273]
Smaller < 18.5	-0.022	[-0.279;0.229]
Larger ≥ 18.5	0.332	[0.131;0.542]
Smaller < 38	0.076	[-0.126;0.273]
Larger ≥ 38	0.463	[0.249;0.687]
Smaller < 216	0.174	[-0.04;0.385]
Larger ≥ 216	0.615	[0.225;1.032]

Tables Regarding Continuous Syllable Repertoire Size Models

Supplemental Table S28: Continuous syllable repertoire model meta-analysis with each species removed in turn. Removal of one species did not significantly affect the data. Asterisks (*) denote significant slopes.

Removed	Group	#Species	#Measure	Post Mean	95% CredInt	pMCMC
Acrocephalus arundinaceus	Intercept	24	73	-0.382	[-0.817;0.064]	0.079
Acrocephalus arundinaceus	Slope	24	73	0.179	[0.075;0.283]	0.001*
Acrocephalus bistrigiceps	Intercept	24	85	-0.358	[-0.777;0.053]	0.079
Acrocephalus bistrigiceps	Slope	24	85	0.176	[0.074;0.277]	0.001*
Acrocephalus palustris	Intercept	24	81	-0.424	[-0.86;0.006]	0.052
Acrocephalus palustris	Slope	24	81	0.198	[0.085;0.314]	0.002*
Acrocephalus schoenobaenus	Intercept	24	80	-0.365	[-0.784;0.028]	0.072
Acrocephalus schoenobaenus	Slope	24	80	0.177	[0.076;0.281]	0.002*
Agelaius phoeniceus	Intercept	24	84	-0.34	[-0.756;0.057]	0.093
Agelaius phoeniceus	Slope	24	84	0.168	[0.065;0.272]	0.002*
Anthus spinoletta	Intercept	24	84	-0.391	[-0.843;0.045]	0.074
Anthus spinoletta	Slope	24	84	0.183	[0.075;0.292]	0.001*
Cardinalis cardinalis	Intercept	24	85	-0.324	[-0.709;0.08]	0.094
Cardinalis cardinalis	Slope	24	85	0.169	[0.073;0.266]	0.001*
Carpodacus mexicanus	Intercept	24	84	-0.364	[-0.777;0.039]	0.072
Carpodacus mexicanus	Slope	24	84	0.176	[0.076;0.277]	0.002*
Dendroica pensylvanica	Intercept	24	84	-0.448	[-0.863;-0.017]	0.032
Dendroica pensylvanica	Slope	24	84	0.196	[0.1;0.298]	0*
Emberiza schoeniclus	Intercept	24	85	-0.31	[-0.698;0.06]	0.096
Emberiza schoeniclus	Slope	24	85	0.165	[0.07;0.261]	0.001*
Ficedula albicollis	Intercept	24	84	-0.306	[-0.699;0.072]	0.113
Ficedula albicollis	Slope	24	84	0.169	[0.075;0.262]	0.002*
Ficedula hypoleuca	Intercept	24	85	-0.38	[-0.814;0.036]	0.071
Ficedula hypoleuca	Slope	24	85	0.177	[0.077;0.28]	0.001*
Hirundo rustica	Intercept	24	84	-0.351	[-0.764;0.072]	0.089
Hirundo rustica	Slope	24	84	0.174	[0.074;0.279]	0.001*
Luscinia megarhynchos	Intercept	24	85	-0.362	[-0.805;0.053]	0.084
Luscinia megarhynchos	Slope	24	85	0.176	[0.073;0.286]	0.002*
Melospiza melodia	Intercept	24	72	-0.35	[-0.785;0.033]	0.083
Melospiza melodia	Slope	24	72	0.168	[0.065;0.271]	0.002*
Mimus polyglottos	Intercept	24	85	-0.322	[-0.714;0.1]	0.106
Mimus polyglottos	Slope	24	85	0.163	[0.064;0.264]	0.002*
Parus caeruleus	Intercept	24	84	-0.395	[-0.825;0.013]	0.061
Parus caeruleus	Slope	24	84	0.184	[0.082;0.287]	0.001*
Parus major	Intercept	24	81	-0.353	[-0.799;0.075]	0.106
Parus major	Slope	24	81	0.175	[0.07;0.283]	0.002*
Phylloscopus fuscatus	Intercept	24	85	-0.356	[-0.775;0.055]	0.081
Phylloscopus fuscatus	Slope	24	85	0.175	[0.075;0.275]	0.001*
Phylloscopus trochilus	Intercept	24	82	-0.344	[-0.761;0.059]	0.091
Phylloscopus trochilus	Slope	24	82	0.169	[0.07;0.27]	0.002*
Plectrophenax nivalis	Intercept	24	84	-0.33	[-0.749;0.072]	0.105
Plectrophenax nivalis	Slope	24	84	0.168	[0.065;0.268]	0.002*
Sturnella neglecta	Intercept	24	79	-0.371	[-0.802;0.037]	0.076
Sturnella neglecta	Slope	24	79	0.179	[0.073;0.287]	0.002*
Sturnus vulgaris	Intercept	24	80	-0.361	[-0.756;0.034]	0.064
Sturnus vulgaris	Slope	24	80	0.172	[0.078;0.271]	0.001*
Sylvia communis	Intercept	24	85	-0.357	[-0.79;0.049]	0.082
Sylvia communis	Slope	24	85	0.175	[0.075;0.28]	0.001*
Wilsonia canadensis	Intercept	24	84	-0.337	[-0.749;0.051]	0.093
Wilsonia canadensis	Slope	24	84	0.172	[0.074;0.272]	0.002*

Supplemental Table S29: Continuous syllable repertoire model meta-analysis where 3 to 9 species with the largest syllable repertoires were removed. Asterisks (*) denote significant slopes.

# Species Removed	Group	#Species	#Measure	Post Mean	95% CredInt	pMCMC
3	Intercept	21	78	-0.4	[-0.912;0.149]	0.13
3	Slope	21	78	0.189	[0.027;0.341]	0.024*
4	Intercept	20	71	-0.423	[-1.068;0.194]	0.17
4	Slope	20	71	0.196	[-0.002;0.387]	0.054
5	Intercept	19	65	-0.452	[-1.14;0.238]	0.172
5	Slope	19	65	0.204	[-0.008;0.428]	0.065
6	Intercept	18	64	-0.466	[-1.176;0.296]	0.188
6	Slope	18	64	0.21	[-0.021;0.446]	0.079
7	Intercept	17	58	-0.363	[-1.08;0.386]	0.301
7	Slope	17	58	0.167	[-0.079;0.408]	0.166
8	Intercept	16	56	-0.328	[-1.119;0.482]	0.377
8	Slope	16	56	0.152	[-0.119;0.425]	0.242
9	Intercept	15	42	-0.141	[-1.114;0.727]	0.742
9	Slope	15	42	0.077	[-0.25;0.394]	0.599

Supplemental Table S30: Continuous syllable repertoire model meta-analysis where 3 to 9 species with the smallest syllable repertoires were removed. Asterisks (*) denote significant slopes.

# Species Removed	Group	#Species	#Measure	Post Mean	95% CredInt	pMCMC
3	Intercept	22	80	-0.412	[-0.901;0.075]	0.088
3	Slope	22	80	0.187	[0.071;0.314]	0.003*
4	Intercept	21	75	-0.446	[-0.985;0.131]	0.108
4	Slope	21	75	0.194	[0.056;0.326]	0.006*
5	Intercept	20	73	-0.592	[-1.155;0]	0.041
5	Slope	20	73	0.229	[0.09;0.365]	0.002*
6	Intercept	19	72	-0.493	[-1.079;0.076]	0.082
6	Slope	19	72	0.207	[0.071;0.342]	0.005*
7	Intercept	18	70	-0.416	[-1.022;0.174]	0.158
7	Slope	18	70	0.192	[0.055;0.332]	0.007*
8	Intercept	17	69	-0.323	[-0.914;0.269]	0.268
8	Slope	17	69	0.171	[0.028;0.311]	0.017*
9	Intercept	16	67	-0.261	[-0.921;0.359]	0.41
9	Slope	16	67	0.155	[0.004;0.307]	0.044*

Supplemental Table S31: Continuous syllable repertoire model meta-analysis when the maximum or minimum values for syllable repertoire size reported in the literature were used. Asterisks (*) denote significant slopes.

Min or Max	Group	#Species	#Measure	Post Mean	95% CredInt	pMCMC
Max	Intercept	25	86	-0.356	[-0.78;0.067]	0.09
Max	Slope	25	86	0.165	[0.072;0.264]	0.001*
Min	Intercept	25	86	-0.282	[-0.659;0.078]	0.128
Min	Slope	25	86	0.166	[0.068;0.259]	0.002*

Supplemental Table S32: Continuous syllable repertoire model meta-analysis in the repertoire dataset with territory-controlled measurements. Asterisks (*) denote significant slopes.

Group	#Species	#Measure	Post Mean	95% CredInt	pMCMC
Intercept	25	86	-0.356	[-0.753;0.056]	0.081
Slope	25	86	0.175	[0.075;0.272]	0.001*

Tables Regarding Discrete Syllable Repertoire Size Models

Supplemental Table S33: Relatively larger syllable repertoires are predictive of a correlation between individual elaboration and reproductive success for most tested thresholds. There was significant evidence for this correlation in the larger repertoire group when the threshold 18.15 to 216. There was not significant evidence for this correlation in the smaller repertoire group for any threshold. Performed in the syllable repertoire dataset. Asterisks (*) denote significant groups.

Threshold	Group	#Species	#Measure	Post Mean	95% CredInt	pMCMC
<7.46	Smaller	2	4	0.161	[-0.378;0.695]	0.538
≥7.46	Larger	23	82	0.249	[-0.025;0.535]	0.067
<7.9	Smaller	3	6	0.054	[-0.382;0.526]	0.812
≥7.9	Larger	22	80	0.263	[0.007;0.551]	0.05
<10.75	Smaller	4	11	0.03	[-0.369;0.436]	0.88
≥10.75	Larger	21	75	0.284	[0.011;0.569]	0.042*
<14	Smaller	5	13	0.116	[-0.263;0.489]	0.515
≥14	Larger	20	73	0.271	[03.00;0.543]	0.047*
<17.43	Smaller	6	14	0.042	[-0.297;0.388]	0.804
≥17.43	Larger	19	72	0.294	[0.045;0.554]	0.029*
<18.15	Smaller	7	16	0.016	[-0.313;0.337]	0.914
≥18.15	Larger	18	70	0.311	[0.051;0.567]	0.021*
<18.5	Smaller	8	17	-0.034	[-0.338;0.267]	0.82
≥18.5	Larger	17	69	0.333	[0.093;0.574]	0.01*
<20.1	Smaller	9	19	-0.027	[-0.317;0.264]	0.857
≥20.1	Larger	16	67	0.344	[0.115;0.597]	0.009*
<22.5	Smaller	10	21	-0.01	[-0.268;0.258]	0.946
≥22.5	Larger	15	65	0.358	[0.134;0.594]	0.005*
<25.3	Smaller	11	34	0.053	[-0.215;0.309]	0.652
≥25.3	Larger	14	52	0.388	[0.121;0.629]	0.005*
<28.4	Smaller	12	35	0.056	[-0.199;0.316]	0.627
≥28.4	Larger	13	51	0.393	[0.152;0.651]	0.003*
<31.4	Smaller	13	36	0.085	[-0.164;0.329]	0.454
≥31.4	Larger	12	50	0.393	[0.143;0.632]	0.005*
<35.1	Smaller	14	38	0.071	[-0.146;0.3]	0.484
≥35.1	Larger	11	48	0.453	[0.225;0.688]	0.002*
<38	Smaller	15	42	0.098	[-0.121;0.324]	0.336
≥38	Larger	10	44	0.482	[0.244;0.732]	0.001*
<41.3	Smaller	16	56	0.124	[-0.125;0.369]	0.276
≥41.3	Larger	9	30	0.491	[0.204;0.775]	0.002*
<41.95	Smaller	17	58	0.133	[-0.118;0.373]	0.244
≥41.95	Larger	8	28	0.501	[0.208;0.802]	0.002*
<55	Smaller	18	64	0.165	[-0.097;0.434]	0.178
≥55	Larger	7	22	0.486	[0.155;0.824]	0.006*
<86	Smaller	20	71	0.175	[-0.072;0.44]	0.147
≥86	Larger	5	15	0.553	[0.197;0.928]	0.005*
<216	Smaller	21	78	0.185	[-0.068;0.459]	0.141
≥216	Larger	4	8	0.611	[0.194;1.051]	0.006*
<241	Smaller	22	79	0.206	[-0.072;0.478]	0.116
≥241	Larger	3	7	0.536	[0.068;1.049]	0.029*
<367.5	Smaller	23	84	0.22	[-0.049;0.497]	0.093
≥367.5	Larger	2	2	0.722	[0.014;1.44]	0.046*

Supplemental Table S34: Larger and smaller syllable repertoire size groups have significantly different correlations between individual song elaboration and reproductive success for most thresholds. BEST analysis predicts that the two groups had significantly different means from the thresholds 10.75 to 86. Performed in the syllable repertoire dataset. Asterisks (*) denote models with significantly different groups.

Threshold	BEST Mean	95% CredInt	%<0
7.46	0.125	[-0.337;0.587]	22.7
7.90	0.257	[-0.093;0.607]	6.6
10.75	0.26	[0.086;0.434]	0.3*
14	0.272	[0.117;0.427]	0.1*
17.43	0.307	[0.15;0.465]	0.1*
18.15	0.315	[0.169;0.461]	0.1*
18.50	0.358	[0.198;0.519]	0.1*
20.10	0.36	[0.201;0.518]	0.1*
22.50	0.373	[0.225;0.522]	0.1*
25.30	0.314	[0.165;0.463]	0.1*
28.40	0.337	[0.19;0.485]	0.1*
31.40	0.329	[0.179;0.479]	0.1*
35.10	0.376	[0.232;0.521]	0.1*
38	0.365	[0.216;0.515]	0.1*
41.30	0.324	[0.142;0.506]	0.1*
41.95	0.331	[0.138;0.525]	0.1*
55	0.238	[0.041;0.435]	1.3*
86	0.294	[0.144;0.444]	0.1*
216	0.246	[-0.028;0.52]	3.6*
241	0.189	[-0.085;0.462]	7.9
367.50	9.115	[-44.729;62.958]	32.6

Supplemental Table S35: Relatively larger syllable repertoires are predictive of a correlation between individual elaboration and reproductive success all tested thresholds. There was not significant evidence for this correlation in the smaller repertoire group for any tested threshold. Performed in the song stability dataset. Asterisks (*) denote significant groups.

Threshold	Group	#Species	#Measure	Post Mean	95% CredInt	pMCMC
<18.5	Smaller	5	11	-0.016	[-0.375;0.343]	0.937
≥18.5	Larger	15	66	0.336	[0.099;0.579]	0.009*
<38	Smaller	12	36	0.111	[-0.114;0.352]	0.31
≥38	Larger	8	41	0.486	[0.228;0.738]	0.002*
<216	Smaller	16	69	0.216	[-0.041;0.47]	0.091
≥216	Larger	4	8	0.637	[0.205;1.105]	0.006*

Supplemental Table S36: Removal of individual species does not significantly affect the syllable repertoire results. Performed in the syllable repertoire dataset. Threshold $\geq$ 18.5. Asterisks (*) denote significant groups.

Removed	Group	#Species	#Measure	Post Mean	95% CredInt	pMCMC
Acrocephalus arundinaceus	Smaller	8	17	-0.037	[-0.364;0.27]	0.813
Acrocephalus arundinaceus	Larger	16	56	0.353	[0.093;0.608]	0.013*
Acrocephalus bistrigiceps	Smaller	8	17	-0.038	[-0.345;0.257]	0.784
Acrocephalus bistrigiceps	Larger	16	68	0.33	[0.101;0.547]	0.007*
Acrocephalus palustris	Smaller	8	17	-0.045	[-0.359;0.241]	0.762
Acrocephalus palustris	Larger	16	64	0.32	[0.092;0.555]	0.01*
Acrocephalus schoenobaenus	Smaller	8	17	-0.055	[-0.363;0.219]	0.697
Acrocephalus schoenobaenus	Larger	16	63	0.335	[0.122;0.543]	0.005*
Agelaius phoeniceus	Smaller	8	17	-0.037	[-0.32;0.25]	0.798
Agelaius phoeniceus	Larger	16	67	0.341	[0.107;0.563]	0.008*
Anthus spinoletta	Smaller	7	15	-0.048	[-0.354;0.277]	0.757
Anthus spinoletta	Larger	17	69	0.333	[0.097;0.576]	0.012*
Cardinalis cardinalis	Smaller	7	16	0.001	[-0.28;0.289]	0.988
Cardinalis cardinalis	Larger	17	69	0.331	[0.105;0.561]	0.01*
Carpodacus mexicanus	Smaller	8	17	-0.034	[-0.327;0.258]	0.81
Carpodacus mexicanus	Larger	16	67	0.334	[0.088;0.568]	0.012*
Dendroica pensylvanica	Smaller	7	15	-0.098	[-0.407;0.208]	0.505
Dendroica pensylvanica	Larger	17	69	0.336	[0.087;0.589]	0.015*
Emberiza schoeniclus	Smaller	7	16	0.008	[-0.263;0.29]	0.931
Emberiza schoeniclus	Larger	17	69	0.329	[0.122;0.533]	0.008*
Ficedula albicollis	Smaller	8	17	-0.011	[-0.287;0.259]	0.93
Ficedula albicollis	Larger	16	67	0.37	[0.138;0.591]	0.005*
Ficedula hypoleuca	Smaller	8	17	-0.049	[-0.368;0.246]	0.761
Ficedula hypoleuca	Larger	16	68	0.315	[0.071;0.555]	0.023*
Hirundo rustica	Smaller	8	17	-0.032	[-0.327;0.259]	0.827
Hirundo rustica	Larger	16	67	0.35	[0.124;0.591]	0.008*
Luscinia megarhynchos	Smaller	8	17	-0.037	[-0.358;0.252]	0.807
Luscinia megarhynchos	Larger	16	68	0.32	[0.084;0.567]	0.017*
Melospiza melodia	Smaller	8	17	-0.026	[-0.333;0.258]	0.863
Melospiza melodia	Larger	16	55	0.317	[0.069;0.56]	0.017*
Mimus polyglottos	Smaller	8	17	-0.035	[-0.328;0.247]	0.807
Mimus polyglottos	Larger	16	68	0.318	[0.091;0.548]	0.012*
Parus caeruleus	Smaller	7	15	-0.058	[-0.36;0.234]	0.678
Parus caeruleus	Larger	17	69	0.334	[0.105;0.575]	0.012*
Parus major	Smaller	7	12	-0.037	[-0.361;0.276]	0.8
Parus major	Larger	17	69	0.339	[0.098;0.58]	0.011*
Phylloscopus fuscatus	Smaller	8	17	-0.033	[-0.336;0.255]	0.821
Phylloscopus fuscatus	Larger	16	68	0.336	[0.115;0.577]	0.011*
Phylloscopus trochilus	Smaller	8	17	-0.025	[-0.352;0.267]	0.879
Phylloscopus trochilus	Larger	16	65	0.328	[0.085;0.576]	0.015*
Plectrophenax nivalis	Smaller	7	15	-0.016	[-0.321;0.286]	0.919
Plectrophenax nivalis	Larger	17	69	0.332	[0.097;0.565]	0.011*
Sturnella neglecta	Smaller	8	17	-0.039	[-0.343;0.246]	0.793
Sturnella neglecta	Larger	16	62	0.326	[0.096;0.576]	0.013*
Sturnus vulgaris	Smaller	8	17	-0.034	[-0.315;0.246]	0.815
Sturnus vulgaris	Larger	16	63	0.316	[0.092;0.544]	0.011*
Sylvia communis	Smaller	8	17	-0.036	[-0.328;0.263]	0.805
Sylvia communis	Larger	16	68	0.326	[0.096;0.557]	0.011*
Wilsonia canadensis	Smaller	7	15	-0.024	[-0.308;0.273]	0.864
Wilsonia canadensis	Larger	17	69	0.338	[0.117;0.574]	0.01*

Supplemental Table S37: Removal of individual species does not significantly affect the syllable repertoire results. Performed in the syllable repertoire dataset. Threshold ≥ 38. Asterisks (*) denote significant groups.

Removed	Group	#Species	#Measure	Post Mean	95% CredInt	pMCMC
Acrocephalus arundinaceus	Smaller	14	29	0.046	[-0.187;0.272]	0.651
Acrocephalus arundinaceus	Larger	10	44	0.468	[0.242;0.718]	0.002*
Acrocephalus bistrigiceps	Smaller	15	42	0.097	[-0.108;0.317]	0.327
Acrocephalus bistrigiceps	Larger	9	43	0.49	[0.252;0.723]	0.001*
Acrocephalus palustris	Smaller	15	42	0.093	[-0.118;0.312]	0.352
Acrocephalus palustris	Larger	9	39	0.487	[0.239;0.743]	0.001*
Acrocephalus schoenobaenus	Smaller	15	42	0.102	[-0.089;0.291]	0.256
Acrocephalus schoenobaenus	Larger	9	38	0.531	[0.307;0.759]	0*
Agelaius phoeniceus	Smaller	14	40	0.078	[-0.136;0.289]	0.424
Agelaius phoeniceus	Larger	10	44	0.454	[0.224;0.682]	0.001*
Anthus spinoletta	Smaller	14	40	0.094	[-0.138;0.319]	0.366
Anthus spinoletta	Larger	10	44	0.474	[0.233;0.722]	0.002*
Cardinalis cardinalis	Smaller	14	41	0.101	[-0.108;0.31]	0.295
Cardinalis cardinalis	Larger	10	44	0.465	[0.239;0.699]	0.001*
Carpodacus mexicanus	Smaller	15	42	0.088	[-0.129;0.311]	0.37
Carpodacus mexicanus	Larger	9	42	0.478	[0.24;0.735]	0.001*
Dendroica pensylvanica	Smaller	14	40	0.06	[-0.182;0.296]	0.564
Dendroica pensylvanica	Larger	10	44	0.466	[0.218;0.731]	0.003*
Emberiza schoeniclus	Smaller	14	41	0.102	[-0.098;0.282]	0.254
Emberiza schoeniclus	Larger	10	44	0.459	[0.256;0.669]	0.001*
Ficedula albicollis	Smaller	14	40	0.137	[-0.09;0.373]	0.199
Ficedula albicollis	Larger	10	44	0.491	[0.258;0.759]	0.001*
Ficedula hypoleuca	Smaller	14	41	0.072	[-0.15;0.295]	0.476
Ficedula hypoleuca	Larger	10	44	0.475	[0.246;0.726]	0.001*
Hirundo rustica	Smaller	14	40	0.097	[-0.136;0.32]	0.35
Hirundo rustica	Larger	10	44	0.483	[0.238;0.735]	0.001*
Luscinia megarhynchos	Smaller	15	42	0.094	[-0.129;0.324]	0.361
Luscinia megarhynchos	Larger	9	43	0.47	[0.224;0.72]	0.001*
Melospiza melodia	Smaller	15	42	0.087	[-0.142;0.304]	0.399
Melospiza melodia	Larger	9	30	0.473	[0.195;0.728]	0.003*
Mimus polyglottos	Smaller	15	42	0.094	[-0.12;0.312]	0.338
Mimus polyglottos	Larger	9	43	0.459	[0.219;0.693]	0.002*
Parus caeruleus	Smaller	14	40	0.086	[-0.135;0.313]	0.397
Parus caeruleus	Larger	10	44	0.477	[0.22;0.717]	0.002*
Parus major	Smaller	14	37	0.106	[-0.123;0.349]	0.333
Parus major	Larger	10	44	0.486	[0.232;0.744]	0.002*
Phylloscopus fuscatus	Smaller	14	41	0.1	[-0.116;0.328]	0.331
Phylloscopus fuscatus	Larger	10	44	0.483	[0.243;0.729]	0.001*
Phylloscopus trochilus	Smaller	14	38	0.077	[-0.149;0.293]	0.462
Phylloscopus trochilus	Larger	10	44	0.47	[0.234;0.712]	0.002*
Plectrophenax nivalis	Smaller	14	40	0.097	[-0.119;0.319]	0.329
Plectrophenax nivalis	Larger	10	44	0.466	[0.225;0.695]	0.002*
Sturnella neglecta	Smaller	15	42	0.083	[-0.15;0.298]	0.414
Sturnella neglecta	Larger	9	37	0.473	[0.213;0.726]	0.002*
Sturnus vulgaris	Smaller	15	42	0.096	[-0.113;0.316]	0.342
Sturnus vulgaris	Larger	9	38	0.466	[0.222;0.72]	0.002*
Sylvia communis	Smaller	15	42	0.097	[-0.119;0.321]	0.351
Sylvia communis	Larger	9	43	0.475	[0.236;0.721]	0.002*
Wilsonia canadensis	Smaller	14	40	0.093	[-0.124;0.309]	0.348
Wilsonia canadensis	Larger	10	44	0.47	[0.232;0.707]	0.001*

Supplemental Table S38: Removal of individual species does not significantly affect the syllable repertoire results. Performed in the syllable repertoire dataset. Threshold ≥ 216. Asterisks (*) denote significant groups.

Removed	Group	#Species	#Measure	Post Mean	95% CredInt	pMCMC
Acrocephalus arundinaceus	Smaller	20	65	0.181	[-0.091;0.462]	0.162
Acrocephalus arundinaceus	Larger	4	8	0.627	[0.16;1.067]	0.007*
Acrocephalus bistrigiceps	Smaller	20	77	0.192	[-0.054;0.436]	0.103
Acrocephalus bistrigiceps	Larger	4	8	0.62	[0.187;1.034]	0.004*
Acrocephalus palustris	Smaller	21	78	0.18	[-0.075;0.415]	0.122
Acrocephalus palustris	Larger	3	3	0.782	[0.247;1.338]	0.006*
Acrocephalus schoenobaenus	Smaller	20	72	0.177	[-0.058;0.409]	0.114
Acrocephalus schoenobaenus	Larger	4	8	0.627	[0.221;1.062]	0.004*
Agelaius phoeniceus	Smaller	20	76	0.181	[-0.058;0.42]	0.124
Agelaius phoeniceus	Larger	4	8	0.59	[0.161;1.017]	0.008*
Anthus spinoletta	Smaller	20	76	0.19	[-0.072;0.45]	0.134
Anthus spinoletta	Larger	4	8	0.615	[0.163;1.037]	0.007*
Cardinalis cardinalis	Smaller	20	77	0.195	[-0.046;0.433]	0.099
Cardinalis cardinalis	Larger	4	8	0.606	[0.19;1.012]	0.004*
Carpodacus mexicanus	Smaller	20	76	0.172	[-0.085;0.428]	0.168
Carpodacus mexicanus	Larger	4	8	0.611	[0.174;1.053]	0.009*
Dendroica pensylvanica	Smaller	20	76	0.162	[-0.101;0.424]	0.191
Dendroica pensylvanica	Larger	4	8	0.608	[0.171;1.052]	0.01*
Emberiza schoeniclus	Smaller	20	77	0.201	[-0.022;0.412]	0.07
Emberiza schoeniclus	Larger	4	8	0.594	[0.206;1.002]	0.004*
Ficedula albicollis	Smaller	20	76	0.227	[-0.033;0.494]	0.073
Ficedula albicollis	Larger	4	8	0.61	[0.194;1.037]	0.006*
Ficedula hypoleuca	Smaller	20	77	0.157	[-0.113;0.412]	0.216
Ficedula hypoleuca	Larger	4	8	0.598	[0.164;1.028]	0.008*
Hirundo rustica	Smaller	20	76	0.192	[-0.085;0.451]	0.134
Hirundo rustica	Larger	4	8	0.617	[0.194;1.059]	0.006*
Luscinia megarhynchos	Smaller	21	78	0.183	[-0.085;0.436]	0.144
Luscinia megarhynchos	Larger	3	7	0.579	[0.123;1.032]	0.015*
Melospiza melodia	Smaller	20	64	0.159	[-0.076;0.416]	0.169
Melospiza melodia	Larger	4	8	0.592	[0.178;1.031]	0.008*
Mimus polyglottos	Smaller	21	78	0.189	[-0.061;0.443]	0.12
Mimus polyglottos	Larger	3	7	0.537	[0.068;0.999]	0.021*
Parus caeruleus	Smaller	20	76	0.177	[-0.076;0.448]	0.155
Parus caeruleus	Larger	4	8	0.605	[0.181;1.051]	0.008*
Parus major	Smaller	20	73	0.196	[-0.067;0.469]	0.129
Parus major	Larger	4	8	0.614	[0.193;1.065]	0.006*
Phylloscopus fuscatus	Smaller	20	77	0.188	[-0.06;0.448]	0.129
Phylloscopus fuscatus	Larger	4	8	0.613	[0.194;1.055]	0.005*
Phylloscopus trochilus	Smaller	20	74	0.172	[-0.092;0.446]	0.167
Phylloscopus trochilus	Larger	4	8	0.597	[0.174;1.024]	0.009*
Plectrophenax nivalis	Smaller	20	76	0.192	[-0.063;0.437]	0.111
Plectrophenax nivalis	Larger	4	8	0.609	[0.186;1.04]	0.008*
Sturnella neglecta	Smaller	20	71	0.164	[-0.096;0.404]	0.168
Sturnella neglecta	Larger	4	8	0.608	[0.189;1.067]	0.007*
Sturnus vulgaris	Smaller	20	72	0.154	[-0.092;0.396]	0.189
Sturnus vulgaris	Larger	4	8	0.61	[0.185;1.008]	0.005*
Sylvia communis	Smaller	21	78	0.185	[-0.069;0.444]	0.136
Sylvia communis	Larger	3	7	0.604	[0.161;1.066]	0.01*
Wilsonia canadensis	Smaller	20	76	0.191	[-0.06;0.443]	0.115
Wilsonia canadensis	Larger	4	8	0.614	[0.174;1.045]	0.006*

Supplemental Table S39: Using the maximum values in the literature for syllable repertoire size did not significantly affect the results. Performed in the syllable repertoire dataset. Asterisks (*) denote significant groups.

Threshold	Group	#Species	#Measure	Post Mean	95% CredInt	pMCMC
<7.46	Smaller	1	2	0.067	[-0.599;0.784]	0.848
≥7.46	Larger	24	84	0.25	[-0.024;0.534]	0.064
<9.6	Smaller	2	4	-0.044	[-0.574;0.466]	0.853
≥9.6	Larger	23	82	0.261	[-0.015;0.528]	0.052
<10	Smaller	3	6	0.054	[-0.382;0.526]	0.812
≥10	Larger	22	80	0.263	[0.007;0.551]	0.05
<14	Smaller	4	11	0.03	[-0.369;0.436]	0.88
≥14	Larger	21	75	0.284	[0.011;0.569]	0.042*
<16.1	Smaller	5	12	-0.051	[-0.409;0.329]	0.779
≥16.1	Larger	20	74	0.311	[0.039;0.584]	0.028*
<17.43	Smaller	6	14	0.042	[-0.297;0.388]	0.804
≥17.43	Larger	19	72	0.294	[0.045;0.554]	0.029*
<18.5	Smaller	7	16	0.016	[-0.313;0.337]	0.914
≥18.5	Larger	18	70	0.311	[0.051;0.567]	0.021*
<20.2	Smaller	8	18	0.018	[-0.296;0.318]	0.894
≥20.2	Larger	17	68	0.322	[0.062;0.574]	0.017*
<27	Smaller	9	20	0.03	[-0.253;0.311]	0.816
≥27	Larger	16	66	0.333	[0.084;0.58]	0.013*
<29.8	Smaller	10	21	0.034	[-0.241;0.316]	0.794
≥29.8	Larger	15	65	0.337	[0.1;0.589]	0.011*
<30	Smaller	11	22	0.068	[-0.201;0.337]	0.598
≥30	Larger	14	64	0.337	[0.1;0.586]	0.012*
<31.4	Smaller	13	36	0.085	[-0.164;0.329]	0.454
≥31.4	Larger	12	50	0.393	[0.143;0.632]	0.005*
<38	Smaller	14	38	0.071	[-0.146;0.3]	0.484
≥38	Larger	11	48	0.453	[0.225;0.688]	0.002*
<41.3	Smaller	15	52	0.102	[-0.144;0.357]	0.379
≥41.3	Larger	10	34	0.457	[0.184;0.728]	0.003*
<44	Smaller	16	54	0.112	[-0.134;0.364]	0.335
≥44	Larger	9	32	0.463	[0.181;0.741]	0.003*
<55	Smaller	17	60	0.148	[-0.121;0.415]	0.232
≥55	Larger	8	26	0.446	[0.125;0.755]	0.007*
<68	Smaller	18	61	0.15	[-0.103;0.428]	0.224
≥68	Larger	7	25	0.454	[0.14;0.778]	0.007*
<80	Smaller	19	67	0.157	[-0.117;0.411]	0.202
≥80	Larger	6	19	0.487	[0.154;0.825]	0.006*
<86	Smaller	20	71	0.175	[-0.072;0.44]	0.147
≥86	Larger	5	15	0.553	[0.197;0.928]	0.005*
<216	Smaller	21	78	0.185	[-0.068;0.459]	0.141
≥216	Larger	4	8	0.611	[0.194;1.051]	0.006*
<411	Smaller	22	79	0.206	[-0.072;0.478]	0.116
≥411	Larger	3	7	0.536	[0.068;1.049]	0.029*
<616	Smaller	23	84	0.22	[-0.049;0.497]	0.093
≥616	Larger	2	2	0.722	[0.014;1.44]	0.046*

Supplemental Table S40: Using the minimum values in the literature for syllable repertoire size did not significantly affect the results. Performed in the syllable repertoire dataset. Asterisks (*) denote significant groups.

Threshold	Group	#Species	#Measure	Post Mean	95% CredInt	pMCMC
<5.1	Smaller	2	7	0.117	[-0.454;0.695]	0.65
≥5.1	Larger	23	79	0.256	[-0.026;0.553]	0.068
<5.4	Smaller	3	9	0.098	[-0.355;0.56]	0.65
≥5.4	Larger	22	77	0.266	[-0.01;0.566]	0.056
<6.3	Smaller	4	11	0.185	[-0.222;0.612]	0.356
≥6.3	Larger	21	75	0.254	[-0.02;0.542]	0.063
<7.46	Smaller	5	12	0.082	[-0.278;0.475]	0.646
≥7.46	Larger	20	74	0.276	[0.017;0.545]	0.039*
<14	Smaller	6	14	0.039	[-0.306;0.373]	0.818
≥14	Larger	19	72	0.294	[0.057;0.557]	0.023*
<15	Smaller	7	15	-0.015	[-0.322;0.303]	0.915
≥15	Larger	18	71	0.316	[0.077;0.552]	0.013*
<17.43	Smaller	8	28	0.059	[-0.238;0.368]	0.674
≥17.43	Larger	17	58	0.329	[0.064;0.584]	0.017*
<18.5	Smaller	9	30	0.041	[-0.25;0.341]	0.747
≥18.5	Larger	16	56	0.354	[0.094;0.611]	0.013*
<18.6	Smaller	10	32	0.043	[-0.243;0.324]	0.722
≥18.6	Larger	15	54	0.371	[0.119;0.641]	0.011*
<20	Smaller	11	33	0.047	[-0.24;0.316]	0.694
≥20	Larger	14	53	0.376	[0.123;0.635]	0.008*
<26.9	Smaller	12	35	0.056	[-0.199;0.316]	0.627
≥26.9	Larger	13	51	0.393	[0.152;0.651]	0.003*
<27	Smaller	13	37	0.049	[-0.186;0.273]	0.629
≥27	Larger	12	49	0.447	[0.213;0.677]	0.002*
<29.7	Smaller	14	38	0.071	[-0.146;0.3]	0.484
≥29.7	Larger	11	48	0.453	[0.225;0.688]	0.002*
<38	Smaller	15	42	0.098	[-0.121;0.324]	0.336
≥38	Larger	10	44	0.482	[0.244;0.732]	0.001*
<39.9	Smaller	16	56	0.124	[-0.125;0.369]	0.276
≥39.9	Larger	9	30	0.491	[0.204;0.775]	0.002*
<41.3	Smaller	17	62	0.159	[-0.113;0.422]	0.196
≥41.3	Larger	8	24	0.479	[0.167;0.82]	0.005*
<45	Smaller	18	64	0.165	[-0.097;0.434]	0.178
≥45	Larger	7	22	0.486	[0.155;0.824]	0.006*
<55	Smaller	19	70	0.176	[-0.079;0.429]	0.14
≥55	Larger	6	16	0.514	[0.176;0.871]	0.006*
<86	Smaller	20	71	0.175	[-0.072;0.44]	0.147
≥86	Larger	5	15	0.553	[0.197;0.928]	0.005*
<90	Smaller	21	78	0.185	[-0.068;0.459]	0.141
≥90	Larger	4	8	0.611	[0.194;1.051]	0.006*
<119	Smaller	22	83	0.2	[-0.072;0.444]	0.114
≥119	Larger	3	3	0.801	[0.234;1.334]	0.006*
<216	Smaller	23	84	0.206	[-0.049;0.476]	0.108
≥216	Larger	2	2	0.874	[0.209;1.539]	0.011*

Supplemental Table S41: Groups of species with relatively larger and smaller syllable repertoire sizes were significantly different. Performed in the syllable repertoire dataset, using the maximum repertoire size for each species. Asterisks (*) denote significant groups.

Threshold	BEST Mean	95% CredInt	%<0
9.60	0.326	[-0.245;0.897]	6.8
10	0.257	[-0.093;0.607]	6.6
14	0.26	[0.086;0.434]	0.3*
16.10	0.301	[0.118;0.484]	0.1*
17.43	0.307	[0.15;0.465]	0.1*
18.50	0.315	[0.169;0.461]	0.1*
20.20	0.319	[0.173;0.464]	0.1*
27	0.333	[0.192;0.475]	0.1*
29.80	0.356	[0.218;0.495]	0.1*
30	0.339	[0.198;0.481]	0.1*
31.40	0.329	[0.179;0.479]	0.1*
38	0.376	[0.232;0.521]	0.1*
41.30	0.325	[0.155;0.495]	0.1*
44	0.317	[0.14;0.495]	0.1*
55	0.231	[0.045;0.417]	0.7*
68	0.237	[0.048;0.426]	0.9*
80	0.273	[0.108;0.438]	0.1*
86	0.294	[0.144;0.444]	0.1*
216	0.246	[-0.028;0.52]	3.6*
411	0.189	[-0.085;0.462]	7.9
616	9.115	[-44.729;62.958]	32.6

Supplemental Table S42: Groups of species with relatively larger and smaller syllable repertoire sizes were significantly different. Performed in the syllable repertoire dataset, using the minimum repertoire size for each species. Asterisks (*) denote significant groups.

Threshold	BEST Mean	95% CredInt	%<0
5.40	0.196	[0.021;0.371]	1.4*
6.30	0.221	[0.072;0.37]	0.4*
7.46	0.279	[0.087;0.471]	0.3*
14	0.325	[0.149;0.501]	0.1*
15	0.362	[0.183;0.542]	0.1*
17.43	0.26	[0.107;0.413]	0.1*
18.50	0.285	[0.136;0.433]	0.1*
18.60	0.292	[0.142;0.441]	0.1*
20	0.313	[0.161;0.464]	0.1*
26.90	0.337	[0.19;0.485]	0.1*
27	0.384	[0.24;0.528]	0.1*
29.70	0.376	[0.232;0.521]	0.1*
38	0.365	[0.216;0.515]	0.1*
39.90	0.324	[0.142;0.506]	0.1*
41.30	0.225	[0.045;0.404]	0.7*
45	0.238	[0.041;0.435]	1.3*
55	0.273	[0.122;0.425]	0.1*
86	0.294	[0.144;0.444]	0.1*
90	0.246	[-0.028;0.52]	3.6*
119	0.357	[-1.669;2.382]	12.2
216	-12.852	[-109.569;83.864]	38.5

Supplemental Table S43: Relatively larger syllable repertoires are predictive of a correlation between individual elaboration and reproductive success for all tested thresholds. There was not significant evidence for this correlation in the smaller repertoire group for any threshold. Performed in the syllable repertoire dataset with territory-controlled measurements. Asterisks (*) denote significant groups.

Threshold	Group	#Species	#Measure	Post Mean	95% CredInt	pMCMC
<18.5	Smaller	8	17	-0.039	[-0.316;0.25]	0.778
≥18.5	Larger	17	69	0.307	[0.088;0.542]	0.013*
<38	Smaller	15	42	0.084	[-0.136;0.283]	0.374
≥38	Larger	10	44	0.448	[0.212;0.667]	0.002*
<216	Smaller	21	78	0.176	[-0.076;0.424]	0.134
≥216	Larger	4	8	0.476	[0.086;0.902]	0.023*

Supplemental Table S44: Larger and smaller syllable repertoire size groups have significantly different correlations between individual elaboration reproductive success for most thresholds. Performed in syllable repertoire dataset with territory-controlled measurements. Asterisks (*) denote models with significantly different groups.

Threshold	BEST Mean	95% CredInt	%<0
18.50	0.345	[0.187;0.504]	0.1*
38	0.361	[0.22;0.502]	0.1*
216	0.183	[-0.054;0.42]	6.2

Tables Regarding Song Stability Models

Supplemental Table S45: Increasing repertoire size with age was not a better predictor of the correlation between reproductive success and individual repertoire size than song stability. Performed in the song stability dataset. Three species that were labeled as song plastic (*Dendroica pensylvanica*, *Mimus polyglottos*, and *Phylloscopus fuscatus*) were put in the Not Increasing group based on information available in the literature.

Group	#Species	#Measure	Post Mean	95% CredInt	pMCMC
Increasing	11	45	0.278	[-0.008;0.571]	0.056
Not Increasing	9	32	0.238	[-0.092;0.564]	0.14

Supplemental Table S46: Increasing repertoire with age BEST Results in the song stability dataset. There was a reasonable probability that there was no difference between species with repertoires that increased with age and those whose repertoires do not. Three species that were labeled as song plastic (*Dendroica pensylvanica*, *Mimus polyglottos*, and *Phylloscopus fuscatus*) were put in the Not Increasing group based on information available in the literature.

BEST Mean	95% CredInt	%<0
0.096	[-0.087;0.278]	15.6

Supplemental Table S47: Switching the song stability category of most species did not significantly the song stability results. Performed in the song stability dataset. Asterisks (*) denote significant groups.

Switched	Group	#Species	#Measure	Post Mean	95% CredInt	pMCMC
Acrocephalus arundinaceus	Plastic	13	36	0.331	[0.036;0.633]	0.028*
Acrocephalus arundinaceus	Stable	7	41	0.168	[-0.167;0.495]	0.286
Acrocephalus palustris	Plastic	15	54	0.31	[0.034;0.594]	0.028*
Acrocephalus palustris	Stable	5	23	0.149	[-0.226;0.511]	0.39
Acrocephalus schoenobaenus	Plastic	13	43	0.305	[0.011;0.583]	0.035*
Acrocephalus schoenobaenus	Stable	7	34	0.194	[-0.151;0.514]	0.233
Agelaius phoeniceus	Plastic	13	47	0.332	[0.056;0.62]	0.021*
Agelaius phoeniceus	Stable	7	30	0.13	[-0.223;0.471]	0.423
Cardinalis cardinalis	Plastic	15	50	0.31	[0.034;0.594]	0.028*
Cardinalis cardinalis	Stable	5	27	0.149	[-0.226;0.511]	0.39
Dendroica pensylvanica	Plastic	13	47	0.305	[0.026;0.598]	0.034*
Dendroica pensylvanica	Stable	7	30	0.182	[-0.174;0.509]	0.276
Emberiza schoeniclus	Plastic	15	50	0.31	[0.034;0.594]	0.028*
Emberiza schoeniclus	Stable	5	27	0.149	[-0.226;0.511]	0.39
Ficedula albicollis	Plastic	13	47	0.35	[0.079;0.632]	0.016*
Ficedula albicollis	Stable	7	30	0.092	[-0.248;0.428]	0.557
Ficedula hypoleuca	Plastic	13	48	0.303	[0.027;0.588]	0.034*
Ficedula hypoleuca	Stable	7	29	0.187	[-0.162;0.525]	0.255
Hirundo rustica	Plastic	13	47	0.327	[0.058;0.62]	0.022*
Hirundo rustica	Stable	7	30	0.137	[-0.207;0.485]	0.408
Luscinia megarhynchos	Plastic	13	48	0.294	[0.023;0.577]	0.036*
Luscinia megarhynchos	Stable	7	29	0.194	[-0.162;0.539]	0.252
Melospiza melodia	Plastic	15	63	0.31	[0.034;0.594]	0.028*
Melospiza melodia	Stable	5	14	0.149	[-0.226;0.511]	0.39
Mimus polyglottos	Plastic	13	48	0.282	[0.01;0.57]	0.044*
Mimus polyglottos	Stable	7	29	0.22	[-0.127;0.57]	0.194
Parus caeruleus	Plastic	15	51	0.31	[0.034;0.594]	0.028*
Parus caeruleus	Stable	5	26	0.149	[-0.226;0.511]	0.39
Parus major	Plastic	15	54	0.31	[0.034;0.594]	0.028*
Parus major	Stable	5	23	0.149	[-0.226;0.511]	0.39
Phylloscopus fuscatus	Plastic	13	48	0.313	[0.036;0.594]	0.027*
Phylloscopus fuscatus	Stable	7	29	0.148	[-0.216;0.505]	0.383
Phylloscopus trochilus	Plastic	13	45	0.313	[0.033;0.606]	0.03*
Phylloscopus trochilus	Stable	7	32	0.174	[-0.173;0.518]	0.293
Sturnella neglecta	Plastic	13	42	0.298	[0.016;0.595]	0.038*
Sturnella neglecta	Stable	7	35	0.2	[-0.146;0.547]	0.235
Sturnus vulgaris	Plastic	13	43	0.287	[-0.005;0.568]	0.043*
Sturnus vulgaris	Stable	7	34	0.211	[-0.148;0.556]	0.216
Sylvia communis	Plastic	13	48	0.296	[0.021;0.579]	0.036*
Sylvia communis	Stable	7	29	0.189	[-0.172;0.526]	0.269

Supplemental Table S48: The strength of the colletarion between individual song elaboration and reproductive success for song plasticity was separated from zero with significant by weak evidence. Performed in the song stability dataset meta-analysis with territory-controlled measurements. Asterisks (*) denote significant groups.

Group	#Species	#Measure	Post Mean	95% CredInt	pMCMC
Stable	14	49	0.144	[-0.201;0.487]	0.378
Plastic	6	28	0.279	[0.014;0.544]	0.036*

Supplemental Table S49: Song stable and song plastic species did not form two significantly different groups. Performed in the song stability dataset meta-analysis with territory-controlled measurements.

BEST Mean	95% CredInt	%<0
0.053	[-0.125;0.23]	28.6

Combined Model and Disentangling Mate Choice from Fecundity

Supplemental Table S50: Combined meta-analysis of syllable repertoire and song stability. Performed in the song stability dataset. Asterisks (*) denote significant groups.

Threshold	Group	#Species	#Measure	Post Mean	95% CredInt	pMCMC
<18.5	SmallerStable	4	9	-0.137	[-0.575;0.283]	0.52
≥18.5	LargerStable	2	19	0.435	[0.011;0.827]	0.036*
<18.5	SmallerPlastic	1	2	0.385	[-0.283;1.042]	0.24
≥18.5	LargerPlastic	13	47	0.308	[0.039;0.583]	0.028*
<38	SmallerStable	4	9	-0.113	[-0.5;0.223]	0.523
≥38	LargerStable	2	19	0.436	[0.106;0.784]	0.015*
<38	SmallerPlastic	8	27	0.187	[-0.065;0.448]	0.137
≥38	LargerPlastic	6	22	0.535	[0.243;0.841]	0.002*
<216	SmallerStable	5	23	0.077	[-0.325;0.468]	0.657
≥216	LargerStable	1	5	0.452	[-0.208;1.109]	0.158
<216	SmallerPlastic	11	46	0.265	[-0.014;0.557]	0.062
≥216	LargerPlastic	3	3	0.843	[0.249;1.442]	0.008*

Supplemental Table S51: Relatively larger syllable repertoire size was not predictive of mating success. Performed in the dataset without offspring measurements.

Threshold	Group	#Species	#Measure	Post Mean	95% CredInt	pMCMC
<18.5	Smaller	6	7	0.071	[-0.524;0.635]	0.753
≥18.5	Larger	17	46	0.272	[-0.254;0.831]	0.195
<38	Smaller	13	21	0.078	[-0.49;0.657]	0.654
≥38	Larger	10	32	0.407	[-0.21;0.972]	0.104
<216	Smaller	19	47	0.18	[-0.401;0.803]	0.366
≥216	Larger	4	6	0.54	[-0.125;1.243]	0.087

Supplemental Table S52: Relatively larger repertoire size was not predictive of social mating success. Performed in the dataset without offspring or extra-pair paternity measurements.

Threshold	Group	#Species	#Measure	Post Mean	95% CredInt	pMCMC
<18.5	Smaller	4	4	0.14	[-0.531;0.824]	0.536
≥18.5	Larger	15	38	0.355	[-0.195;0.971]	0.097
<38	Smaller	10	15	0.151	[-0.557;0.834]	0.35
≥38	Larger	9	27	0.522	[-0.171;1.221]	0.072
<216	Smaller	16	38	0.285	[-0.378;0.949]	0.148
≥216	Larger	3	4	0.605	[-0.146;1.389]	0.076

Supplemental Table S53: Larger and smaller syllable repertoire size groups have significantly different correlations between individual elaboration and mating success only at the threshold of 38. BEST analysis performed in the no offspring dataset. Asterisks (*) denotes the model with significantly different groups.

Threshold	BEST Mean	95% CredInt	%<0
18.50	0.293	[-0.083;0.669]	4.3*
38	0.322	[0.108;0.537]	0.2*
216	0.227	[-0.196;0.651]	11.9

Supplemental Table S54: Larger and smaller syllable repertoire size groups have significantly different correlations between individual elaboration and social mating success only at the threshold of 38. BEST analysis performed in the dataset without offspring or extra-pair paternity measurements. Asterisk (*) denotes the model with significantly different groups.

Threshold	BEST Mean	95% CredInt	%<0
18.50	0.287	[-0.307;0.881]	9.8
38	0.294	[0.071;0.518]	0.5*
216	0.174	[-0.567;0.915]	20.9

References for studies included in meta-analyses and tables

[1] D. Hasselquist, “Polygyny in great reed warblers: A long-term study of factors contributing to male fitness,” *Ecology*, vol. 79, no. 7, pp. 2376–2390, 1998.

[2] C. K. Catchpole, “Song repertoires and reproductive success in the great reed warbler Acrocephalus arundinaceus,” *Behav. Ecol. Sociobiol.*, vol. 19, pp. 439–445, 1986.

[3] W. Forstmeier and B. Leisler, “Repertoire size, sexual selection, and offspring viability in the great reed warbler: Changing patterns in space and time,” *Behav. Ecol.*, vol. 15, no. 4, pp. 555–563, 2004.

[4] B. Leisler, J. Beier, G. Heine, and K.-H. Siebenrock, “Age and other factors influencing mating status in German great reed warblers (*Acrocephalus arundinaceus*),” *Japanese J. Ornithol.*, vol. 44, no. 3, pp. 215–216, 1995.

[5] D. Hasselquist, S. Bensch, and T. von Schantz, “Correlation between male song repertoire, extra-pair paternity and offspring survival in the great reed warbler,” *Nature*, vol. 381, no. 6579, pp. 229–232, 1996.

[6] S. Hamao and H. Eda-Fujiwara, “Vocal mimicry by the black-browed reed warbler *Acrocephalus bistrigiceps*: Objective identification of mimetic sounds,” *Ibis (Lond. 1859).*, vol. 146, no. 1, pp. 61–68, 2004.

[7] B. D. Bell, M. Borowiec, J. Lontkowski, and S. Pledger, “Short records of marsh warbler (*Acrocephalus palustris*) song provide indices that correlate with nesting success,” *J. Ornithol.*, vol. 145, no. 1, pp. 8–15, 2004.

[8] A. Darolová, J. Krištofík, H. Hoi, and M. Wink, “Song complexity in male marsh warblers: Does it reflect male quality?,” *J. Ornithol.*, vol. 153, no. 2, pp. 431–439, 2012.

[9] K. L. Buchanan and C. K. Catchpole, “Female choice in the sedge warbler *Acrocephalus schoenobaenus*: multiple cues from song and territory quality,” *Proc. R. Soc. B Biol. Sci.*, vol. 264, no. 1381, pp. 521–526, 1997.

[10] C. K. Catchpole, “Sexual selection and the evolution of complex songs among European warblers of the genus *Acrocephalus*,” *Behaviour*, 1980.

[11] K. L. Buchanan and C. K. Catchpole, “Extra-pair paternity in the socially monogamous sedge warbler *Acrocephalus schoenobaenus* as revealed by multilocus DNA fingerprinting,” *Ibis (Lond. 1859).*, vol. 142, no. 1, pp. 12–20, 2000.

[12] R. C. Marshall, K. L. Buchanan, and C. K. Catchpole, “Song and female choice for extrapair copulations in the sedge warbler, *Acrocephalus schoenobaenus*,” *Anim. Behav.*, vol. 73, no. 4, pp. 629–635, 2007.

[13] K. Yasukawa, J. L. Blank, and C. B. Patterson, “Song repertoires and sexual

selection in the red-winged blackbird," *Behav. Ecol. Sociobiol.*, vol. 7, no. 3, pp. 233–238, 1980.

[14] U. Rehsteiner, H. Geisser, and H. Reyer, "Singing and mating success in water pipits: one specific song element makes all the difference," *Anim. Behav.*, vol. 55, no. 6, pp. 1471–81, 1998.

[15] R. N. Conner, M. E. Anderson, and J. G. Dickson, "Relationships among territory size, habitat, song, and nesting success of Northern cardinals," *Auk*, vol. 103, no. January, pp. 23–31, 1986.

[16] D. J. Mennill, A. V. Badyaev, L. M. Jonart, and G. E. Hill, "Male house finches with elaborate songs have higher reproductive performance," *Ethology*, vol. 112, no. 2, pp. 174–180, 2006.

[17] B. E. Byers, "Extrapair paternity in chestnut-sided warblers is correlated with consistent vocal performance," *Behav. Ecol.*, vol. 18, no. 1, pp. 130–136, 2007.

[18] K. M. Bouwman, R. E. van Dijk, J. J. Wijmenga, and J. Komdeur, "Older male reed buntings are more successful at gaining extrapair fertilizations," *Anim. Behav.*, vol. 73, no. 1, pp. 15–27, 2007.

[19] G. Hegyi, E. Szöllosi, S. Jenni-Eiermann, J. Török, M. Eens, and L. Z. Garamszegi, "Nutritional correlates and mate acquisition role of multiple sexual traits in male collared flycatchers," *Naturwissenschaften*, vol. 97, no. 6, pp. 567–576, 2010.

[20] L. Z. Garamszegi and A. P. Møller, "Extrapair paternity and the evolution of bird song," *Behav. Ecol.*, vol. 15, no. 3, pp. 508–519, 2004.

[21] H. M. Lampe and Y. O. Espmark, "Mate choice in pied flycatchers *Ficedula hypoleuca*: can females use song to find high-quality males and territories?," *Ibis (Lond. 1859).*, vol. 145, no. 1, pp. E24–E33, 2003.

[22] L. Z. Garamszegi *et al.*, "The design of complex sexual traits in male barn swallows: Associations between signal attributes," *J. Evol. Biol.*, vol. 19, no. 6, pp. 2052–2066, 2006.

[23] C. Landgraf, K. Wilhelm, J. Wirth, M. Weiss, and S. Kipper, "Affairs happen-To whom? A study on extrapair paternity in common nightingales," *Curr. Zool.*, vol. 63, no. 4, pp. 421–431, 2017.

[24] J. M. Reid *et al.*, "Song repertoire size predicts initial mating success in male song sparrows, *Melospiza melodia*," *Anim. Behav.*, vol. 68, no. 5, pp. 1055–1063, 2004.

[25] W. A. Searcy, "Song repertore size and female preferences in song sparrows," *Behav. Ecol. Sociobiol.*, vol. 14, pp. 281–286, 1984.

[26] S. M. Hiebert, P. K. Stoddard, and P. Arcese, "Repertoire size, territory acquisition, and reproductive success in the song sparrow," *Anim. Behav.*, vol. 37, no. PART 2, pp. 266–273, 1989.

[27] J. M. Reid *et al.*, "Fitness correlates of song repertoire size in free-living song sparrows (*Melospiza melodia*)," *Am. Nat.*, vol. 165, no. 3, pp. 299–310, 2005.

[28] C. E. Hill, Ç. Akçay, S. E. Campbell, and M. D. Beecher, "Extrapair paternity, song, and genetic quality in song sparrows," *Behav. Ecol.*, vol. 22, no. 1, pp. 73–81, 2011.
[29] D. A. Potvin, P. W. Crawford, S. A. MacDougall-Shackleton, and E. A. MacDougall-Shackleton, "Song repertoire size, not territory location, predicts reproductive success and territory tenure in a migratory songbird," *Can. J. Zool.*, vol. 93, no. 8, pp. 627–633, 2015.
[30] R. D. Howard, "The influence of sexual selection and interspecific competition on mockingbird song (*Mimus polyglottus*)," *Evolution (N. Y).*, vol. 28, no. 3, pp. 428–438, 1974.
[31] A. Poesel, K. Foerster, and B. Kempenaers, "The dawn song of the blue tit *Parus caeruleus* and its role in sexual selection," *Ethology*, vol. 107, no. 6, pp. 521–531, 2001.
[32] J. Krebs, R. Ashcroft, and M. Webber, "Song repertoires and territory defence in the great tit," *Nature*, vol. 271, no. 5645, pp. 539–542, 1978.
[33] M. Lambrechts and A. A. Dhondt, "Male quality, reproduction, and survival in the great tit (*Parus major*)," *Behav. Ecol. Sociobiol.*, vol. 19, no. 1, pp. 57–63, 1986.
[34] C. McGregor, P., Krebs, J., Perrins, "Song repertoires and lifetime reproductive success in the great tit (*Parus major*)," *Am. Soc. Nat.*, vol. 118, no. 2, pp. 149–159, 1981.
[35] W. Forstmeier, B. Kempenaers, A. Meyer, and B. Leisler, "A novel song parameter correlates with extra-pair paternity and reflects male longevity," *Proc. R. Soc. B Biol. Sci.*, vol. 269, no. 1499, pp. 1479–1485, 2002.
[36] E. S. C. Scordato, "Geographical and temporal variation in sexually selected traits : Environmental variation, multiple signals, and consequences for population divergence," THE UNIVERSITY OF CHICAGO, 2012.
[37] D. Gil, P. J. B. Slater, and J. A. Graves, "Extra-pair paternity and song characteristics in the willow warbler *Phylloscopus trochilus*," *J. Avian Biol.*, vol. 38, no. 3, pp. 291–297, 2007.
[38] D. Gil and P. J. B. Slater, "Multiple song repertoire characteristics in the willow warbler (*Phylloscopus trochilus*): Correlations with female choice and offspring viability," *Behav. Ecol. Sociobiol.*, vol. 47, no. 5, pp. 319–326, 2000.
[39] T. Järvi, "The evolution of song versatility in the willow warbler *Phylloscopus trochilus*: A case of evolution by intersexual selection explained by the 'female's choice of the best mate,'" *Ornis Scand.*, 1983.
[40] E. Hofstad, Y. Espmark, A. Moksnes, T. Haugan, and M. Ingebrigtsen, "The relationship between song performance and male quality in snow buntings (*Plectrophenax nivalis*)," *Can. J. Zool.*, vol. 80, no. 3, pp. 524–531, 2002.
[41] N. Dadwal and D. Bhatt, "Relationship between song repertoire size with nesting

success, territory size, and territorial conflict in pied bush chat (*Saxicola caprata*)," *Wilson J. Ornithol.*, vol. 129, no. 4, pp. 701–712, 2017.

[42] R. E. Lemon, D. M. Weary, and K. J. Norris, "Male morphology and behavior correlate with reproductive success in the American redstart (*Setophaga ruticilla*)," *Behav. Ecol. Sociobiol.*, pp. 399–403, 1992.

[43] a. G. Horn, T. E. Dickinson, and J. B. Falls, "Male quality and song repertoires in Western meadowlarks (*Sturnella-neglecta*)," *Can. J. Zool. Can. Zool.*, vol. 71, pp. 1059–1061, 1993.

[44] M. K. Aweida, "Repertoires, territory size and mate attraction in Western meadowlarks," *Condor*, vol. 97, no. 4, pp. 1080–1083, 1995.

[45] D. J. Mountjoy and R. E. Lemon, "Female choice for complex song in the European starling: a field experiment," *Behav. Ecol. Sociobiol.*, vol. 38, no. 1, pp. 65–71, 1996.

[46] M. Eens, R. Pinxten, and R. F. Verheyen, "Male song as a cue for mate choice in the European starling," *Behaviour*, vol. 116, no. 3–4, pp. 210–238, 1991.

[47] T. J. S. Balsby, "Song activity and variability in relation to male quality and female choice in whitethroats *Sylvia communis*," *J. Avian Biol.*, vol. 31, pp. 56–62, 2000.

[48] A. Demko, "Temporal and individual song variation in the canada warbler (*Cardellina canadensis*)," no. March, pp. 1–114, 2012.

[49] S. Nowicki, D. Hasselquist, S. Bensch, and S. Peters, "Nestling growth and song repertoire size in great reed warblers: evidence for song learning as an indicator mechanism in mate choice.," *Proc. Biol. Sci.*, vol. 267, no. 1460, pp. 2419–24, 2000.

[50] F. Dowsett-Lemaire, "The imitative range of the song of the marsh warbler *Acrocephalus palustris*, with special reference to imitations of african birds," *Ibis (Lond. 1859).*, vol. 121, no. 4, pp. 453–468, 1979.

[51] J. S. Nicholson, K. L. Buchanan, R. C. Marshall, and C. K. Catchpole, "Song sharing and repertoire size in the sedge warbler, *Acrocephalus schoenobaenus*: Changes within and between years," *Anim. Behav.*, 2007.

[52] B. Y. P. Marler, P. Mundinger, M. S. U. E. Waser, and A. N. N. Lutjen, "Song development in red-winged blackbirds (*Agelaius phoeniceus*)," *Anim. Behav.*, pp. 586–606, 1972.

[53] R. E. Lemon, "Geographic variation in the song of cardinals," *Can. J. Zool.*, 1966.

[54] B. E. Byers, "Geographic variation of song form within and among chestnut-sided warbler populations," *Auk*, vol. 113, no. 2, pp. 288–299, 1996.

[55] M. Bessert-Nettelbeck, S. Kipper, C. Bartsch, and S. L. Voigt-Heucke, "Similar, yet different: Male reed buntings (*Emberiza schoeniclus*) show high individual differences in song composition, rates of syllable sharing and use," *J. Ornithol.*, 2014.

[56] L. Z. Garamszegi, J. Török, G. Hegyi, E. Szöllõsi, B. Rosivall, and M. Eens, "Age-dependent expression of song in the collared flycatcher, *Ficedula albicollis*," *Ethology*, vol. 113, no. 3, pp. 246–256, 2007.

[57] A. Eriksen, T. Slagsvold, and H. M. Lampe, "Vocal plasticity - are pied flycatchers, *Ficedula Hypoleuca*, open-ended learners?," *Ethology*, 2011.

[58] L. Z. Garamszegi, D. Heylen, A. P. Møller, M. Eens, and F. De Lope, "Age-dependent health status and song characteristics in the barn swallow," *Behav. Ecol.*, 2005.

[59] P. Galeotti, N. Saino, E. Perani, R. Sacchi, and A. R. Møller, "Age-related song variation in male barn swallows," *Ital. J. Zool.*, vol. 68, no. 4, pp. 305–310, 2001.

[60] S. Kiefer *et al.*, "First-year common nightingales (Luscinia megarhynchos) have smaller song-type repertoire sizes than older males," *Ethology*, vol. 112, no. 12, pp. 1217–1224, 2006.

[61] J. C. Nordby, S. E. Campbell, and M. D. Beecher, "Adult song sparrows do not alter their song repertoires," *Ethology*, 2002.

[62] A. Poesel, H. P. Kunc, K. Foerster, A. Johnsen, and B. Kempenaers, "Early birds are sexy: male age, dawn song and extrapair paternity in blue tits, *Cyanistes* (formerly *Parus*) *caeruleus*," *Anim. Behav.*, 2006.

[63] H. F. Rivera-Gutierrez, R. Pinxten, and M. Eens, "Difficulties when assessing birdsong learning programmes under field conditions: A re-evaluation of song repertoire flexibility in the great tit," *PLoS One*, 2011.

[64] W. Forstmeier and T. J. S. Balsby, "Why mated dusky warblers sing so much: Territory guarding and male quality announcement," *Behaviour*, 2002.

[65] D. Gil, J. L. S. Cobb, and P. J. B. Slater, "Song characteristics are age dependent in the willow warbler, Phylloscopus trochilus," *Anim. Behav.*, 2001.

[66] A. Horn, "Structure of western meadowlark (Sturnella neglecta) song repertoires," *Can. J. Zool.*, vol. 66, no. 2, pp. 284–288, 1988.

[67] D. J. Mountjoy and R. E. Lemon, "Extended song learning in wild European starlings," *Anim. Behav.*, vol. 49, no. 2, pp. 357–366, 1995.

[68] T. J. S. Balsby and P. Hansen, "Element repertoire: Change and development with age in whitethroat *Sylvia communis* song," *J. Ornithol.*, 2010.

[69] J. M. Moore, T. Szekely, J. Buki, and T. J. DeVoogd, "Motor pathway convergence predicts syllable repertoire size in oscine birds," *Proc. Natl. Acad. Sci.*, 2011.

[70] W. Forstmeier, D. Hasselquist, S. Bensch, and B. Leisler, "Does song reflect age and viability? A comparison between two populations of the great reed warbler *Acrocephalus arundinaceus*," *Behav. Ecol. Sociobiol.*, 2006.

[71] T. Szekely, C. K. Catchpole, A. Devoogd, Z. Marchl, and T. J. Devoogd, "Evolutionary changes in a song control area of the brain (HVC) are associated with evolutionary changes in song repertoire among European warblers

(*Sylviidae*).," *Proceedings of the Royal Society B: Biological Sciences*. 1996.

[72] S. Hamao, "Syntactical complexity of songs in the black-browed reed warbler *Acrocephalus bistrigiceps*," *Ornithol. Sci.*, vol. 7, no. 2, pp. 173–177, 2008.

[73] L. Z. Garamszegi, "Bird song and parasites," *Behav. Ecol. Sociobiol.*, vol. 59, no. 2, pp. 167–180, 2005.

[74] R. E. Irwin, "Directional sexual selection cannot explain variation in song repertoire size in the new world blackbirds (*Icterinae*)," *Ethology*, 1990.

[75] M. Soma and L. Z. Garamszegi, "Rethinking birdsong evolution: Meta-analysis of the relationship between song complexity and reproductive success," *Behav. Ecol.*, vol. 22, no. 2, pp. 363–371, 2011.

[76] M. E. Anderson and R. N. Conner, "Northern cardinal song in three forest habitats in EasternTexas.," *Wilson Bull.*, 1985.

[77] T. T. Tracy and M. C. Baker, "Geographic variation of syllables of house finch songs," *Auk*, vol. 116, no. 3, pp. 666–676, 1999.

[78] L. Z. Garamszegi *et al.*, "Estimating the complexity of bird song by using capture-recapture approaches from community ecology," *Behavioral Ecology and Sociobiology*. 2005.

[79] L. Z. Garamszegi, T. Boulinier, A. P. Møller, J. Török, G. Michl, and J. D. Nichols, "The estimation of size and change in composition of avian song repertoires," *Anim. Behav.*, 2002.

[80] Y. O. Espmark and H. M. Lampe, "Variations in the song of the pied flycatcher within and between breeding seasons," *Bioacoustics*, 1993.

[81] H. M. Lampe and Y. O. Espmark, "Singing activity and song pattern of the redwing *Turdus iliacus* during the breeding season," *Ornis Scand.*, 1987.

[82] P. Galeotti, N. Saino, R. Sacchi, and A. P. Moller, "Song correlates with social context, testosterone and body condition in male barn swallows," *Anim. Behav.*, 1997.

[83] D. J. Borror, "Song variation in Maine song-sparrows," *Wilson Bull.*, vol. 77, no. 1, pp. 5–37, 1965.

[84] J. L. Wildenthal, "Structure in primary song of the mockingbird (*Mimus polyglottos*)," *Auk*, 1965.

[85] W. Forstmeier, "Individual reproductive strategies in the dusky warbler (*Phylloscopus fuscatus*): Female and male perspectives," *Thesis*, p. 112, 2001.

[86] T. Järvi, T. Radesäter, and S. Jakobsson, "The song of the willow warbler *phylloscopus trochilus* with special reference to singing behaviour in agonistic situations," *Ornis Scand.*, 1980.

[87] M. Eens, "Understanding the complex song of the European starling: an integrated ethological approach," *Adv. Study Behav.*, 1997.

[88] T. J. S. Balsby and T. Dabelsteen, "The meaning of song repertoire size and song

length to male whitethroats *Sylvia communis*," *Behav. Processes*, 2001.

[89] S. A. MacDougall-Shackleton, "Sexual Selection and the Evolution of Song Repertoires," in *Current Ornithology*, V. Nolan, E. D. Ketterson, and C. F. Thompson, Eds. Boston; MA: Springer US, 1997, pp. 81–124.

[90] T. J. Devoogd, J. R. Krebs, S. D. Healy, and A. Purvis, "Relations between song repertoire size and the volume of brain nuclei related to song: comparative evolutionary analyses amongst oscine birds.," *Proc. Biol. Sci.*, 1993.

[91] C. W. Dobson and R. E. Lemon, "Re-examination of monotony threshold hypothesis in bird song," *Nature*, 1975.

[92] C. K. Catchpole and P. K. McGregor, "Sexual selection, song complexity and plumage dimorphism in European buntings of the genus Emberiza," *Anim. Behav.*, 1985.

[93] E. del Hoyo; J., Elliott, A.; Sargatal, J.; Christie, D.A.; de Juana, Ed., *Handbook of the Birds of the World Alive*. Barcelona: Lynx Edicions, 2017.

References for xeno-canto files used for quantifying syllable repertoire size

Carpodacus mexicanus

Ian Cruickshank, XC73494. Accessible at www.xeno-canto.org/73494.

Nick Komar, XC320727. Accessible at www.xeno-canto.org/320727.

Paul Marvin, XC219331. Accessible at www.xeno-canto.org/219331.

Richard E. Webster, XC268457. Accessible at www.xeno-canto.org/268457.

Richard E. Webster, XC268458. Accessible at www.xeno-canto.org/268458.

Richard E. Webster, XC268463. Accessible at www.xeno-canto.org/268463.

Richard E. Webster, XC268464. Accessible at www.xeno-canto.org/268464.

Richard E. Webster, XC268465. Accessible at www.xeno-canto.org/268465.

Richard E. Webster, XC353018. Accessible at www.xeno-canto.org/353018.

Thomas G. Graves, XC179232. Accessible at www.xeno-canto.org/179232.

Wilsonia canadensis

Andrew Spencer, XC51468. Accessible at www.xeno-canto.org/51468.

Martin St-Michel, XC294137. Accessible at www.xeno-canto.org/294137.

Matt Wistrand, XC370937. Accessible at www.xeno-canto.org/370937.

Matt Wistrand, XC371402. Accessible at www.xeno-canto.org/371402.

Paul J. Hurtado, XC179679. Accessible at www.xeno-canto.org/179679.

Richard E. Webster, XC189300. Accessible at www.xeno-canto.org/189300.

Richard E. Webster, XC189302. Accessible at www.xeno-canto.org/189302.

Saxicola caprata

Allen T. Chartier, XC31442. Accessible at www.xeno-canto.org/31442.

chiefredearth, XC265266. Accessible at www.xeno-canto.org/265266.

David Edwards, XC24586. Accessible at www.xeno-canto.org/24586.

Frank Lambert, XC88897. Accessible at www.xeno-canto.org/88897.

Mike Nelson, XC204612. Accessible at www.xeno-canto.org/204612.

Mike Nelson, XC240349. Accessible at www.xeno-canto.org/240349.

Peter Boesman, XC311490. Accessible at www.xeno-canto.org/311490.

Stuart Fisher, XC19405. Accessible at www.xeno-canto.org/19405.

Phylloscopus trochiloides

Allen T. Chartier, XC31572. Accessible at www.xeno-canto.org/31572.

Frank Lambert, XC88897. Accessible at www.xeno-canto.org/88897.

Lars Edenius, XC376516. Accessible at www.xeno-canto.org/376516.

Manuel Schweizer, XC329369. Accessible at www.xeno-canto.org/329369.

Timo Janhonen, XC118604. Accessible at www.xeno-canto.org/118604.
